# Genome-wide identification of the Dicer-like family in cotton and analysis of the DCL expression modulation in response to biotic stress in two contrasting commercial cultivars

**DOI:** 10.1186/s12870-019-2112-4

**Published:** 2019-11-15

**Authors:** Marianna O. Moura, Anna Karoline S. Fausto, Amanda Fanelli, Fernanda A. de F. Guedes, Tatiane da F. Silva, Elisson Romanel, Maite F. S. Vaslin

**Affiliations:** 1 Departamento de Virologia, Instituto de Microbiologia, Universidade Federal do Rio de Janeiro (UFRJ), Rio de Janeiro, RJ 21941-590 Brazil; 2 Departamento de Biotecnologia, Escola de Engenharia de Lorena/Universidade de São Paulo (EEL/USP), Lorena, SP 12602-810 Brazil; 3 Programa de Pós-graduação em Biotecnologia Vegetal, Universidade Federal do Rio de Janeiro (UFRJ), Rio de Janeiro, RJ 21941-590 Brazil

**Keywords:** Cotton, *G. hirsutum*, DCL, Dicer-like, CLRDV, Biotic stress

## Abstract

**Background:**

Dicer-like proteins (DCLs) are essential players in RNA-silencing mechanisms, acting in gene regulation via miRNAs and in antiviral protection in plants and have also been associated to other biotic and abiotic stresses. To the best of our knowledge, despite being identified in some crops, cotton DCLs haven’t been characterized until now. In this work, we characterized the *DCLs* of three cotton species and analyzed their expression profiles during biotic stress.

**Results:**

As main results, 11 *DCLs* in the allotetraploid cotton *Gossypium hirsutum*, 7 and 6 in the diploid *G. arboreum* and *G. raimondii*, were identified, respectively. Among some *DCLs* duplications observed in these genomes, the presence of an extra *DCL3* in the three cotton species were detected, which haven’t been found in others eudicots. All the *DCL* types identified by in silico analysis in the allotetraploid cotton genome were able to generate transcripts, as observed by gene expression analysis in distinct tissues. Based on the importance of DCLs for plant defense against virus, responses of cotton DCLs to virus infection and/or herbivore attack using two commercial cotton cultivars (cv.), one susceptible (FM966) and another resistant (DO) to polerovirus CLRDV infection, were analyzed. Both cvs. Responded differently to virus infection. At the inoculation site, the resistant cv. showed strong induction of *DCL2a* and *b*, while the susceptible cv. showed a down-regulation of these genes, wherever *DCL4* expression was highly induced. A time course of *DCL* expression in aerial parts far from inoculation site along infection showed that *DCL2b* and *DCL4* were repressed 24 h after infection in the susceptible cotton. As CLRDV is aphid-transmitted, herbivore attack was also checked. Opposite expression pattern of *DCL2a* and *b* and *DCL4* was observed for R and S cottons, showing that aphid feeding alone may induce DCL modulation.

**Conclusions:**

Almost all the DCLs of the allotetraploide *G. hirsutum* cotton were found in their relative diploids. Duplications of *DCL2* and *DCL3* were found in the three species. All four classes of DCL responded to aphid attack and virus infection in *G. hirsutum. DCLs* initial responses against the virus itself and/or herbivore attack may be contributing towards virus resistance.

## Background

DICER proteins represent an ubiquitous class of RNAse III enzymes that are vital for the establishment of RNA interference (RNAi). DCLs recognize and cut a long ds-RNA substrate to release 21–24-nt small RNA duplexes, which have 2-nt 3′ overhangs at each end [1–3]. These small RNAs are important riboregulators in fungi, plants and animals, negatively regulating the expression of specific target genes by base-pairing. The function of small RNAs is largely described in plants associated with both development and responses to abiotic and biotic stress [4–6]. Plant small RNAs are classically separated into short-interfering RNAs (siRNAs) and microRNAs (miRNAs). Different classes of siRNAs have been previously described as natural-antisense siRNAs (natsiRNA) and trans-acting siRNAs (tasiRNAS), which, as miRNAs, are involved in post-transcriptional gene regulation by degrading their mRNA targets or inhibiting their translation. Heterochromatin-associated siRNAs (hcsiRNA) are associated with chromatin modifications and transcriptional repression of their target DNA *loci*. The long non-translated RNA (lsiRNA) and the viral small RNA (vsiRNA) were generated by DICER cutting of intermediate viral genome structures during viruses infection. Once produced, small RNAs are incorporated into ARGONAUTE (AGO)-containing RNA-induced silencing complexes (RISCs) to confer sequence specificity in the silencing of RNA information [3, 5, 7, 8]. In plants and fungi, cellular RNA-dependent RNA polymerase (RDR) acts to convert aberrant RNAs to dsRNA, leading to small RNA amplification and more intensive RNA silencing [3, 9, 10].

DICERs, or DICER-like (DCLs), as these proteins are called in plants, present six domains: DEAD, Helicase-C and DUF283 domains at the N-terminus, a Piwi/Argonaute/Zwille (PAZ) domain in the middle and a dual RNAse III domain followed by one or two dsRNA-binding domains in the C-terminal half [11, 12]. In lower eukaryotes, one or more of these domains may be absent [4]. The helicase domain serves to recruit co-factor regulatory proteins [13–15]. ATP hydrolysis is used to achieve progressive cleavage of the long dsRNA substrate. The DEAD domain acts as an ATP-binding domain [16]. The PAZ domain in turn mediates the recognition of the dsRNA substrate terminus, and the distance between PAZ and the RNAse III catalytic center determines the size of the small RNAs produced [17, 18]. Each of the two RNase III domains cuts one of the dsRNA strands, leaving a characteristic 2-nt overhang at the 3′-end of the product [19–21], and the C-terminal dsRNA-binding domains (dsRBD or DSRM) act as a protein–protein interaction interface and nuclear localization signals, in addition to having dsRNA-binding functions. dsRBD mediates the discrimination of different RNA substrates and subsequent incorporation of effector complexes [22–24]. Nevertheless, multiple dsRBD may be able to act in combination by recognizing secondary structures of specific RNAs [13].

In Arabidopsis, 4 genes (*DCL1–4*) encoding DCL proteins have been found [12, 25]. Each of the four AtDCLs is involved in the biogenesis of specific small RNA species. However, they may play redundant and hierarchical roles in the production of distinct sRNAs [26]. AtDCL1 is responsible for miRNA biogenesis. AtDCL2 generates 22-nt siRNAs and viral siRNAs (vsiRNAs) from endogenous inverted-repeats, integrated viruses, transgenes and RDR-amplified virus dsRNAs [26–28]. AtDCL3 is associated with transcriptional silencing, producing 24-nt length siRNAs involved in the establishment and maintenance of the heterochromatin state through RNA-dependent DNA methylation and histone modification [29, 30]. These 24 siRNAs are mostly generated from repetitive DNA *loci* and transposons, as well as retrotransposon insertion of DNA regions. AtDCL4 responds to the biogenesis of primary vsiRNAs that has 21-nt and are generated by dicing of viral genomes replicative intermediates. This DCL is also responsible for the production of phased siRNAs, ta-siRNAs and virus-activated siRNAS (vasiRNAs, a new class of endogenous RDR1-dependent siRNAs induced by virus infection) [31–33]. DCL4 is very important in viral resistance, initiating virus silencing in primary infected cells [33]. Primary vsiRNAs may further initiate secondary siRNA production, under the action of AtDCL2 and AtDCL4, and other genes [25, 34, 35]. So, DCL2 and 4 are key control virus replication levels even in susceptible plants. The production of the secondary vsiRNAs enables the increase of the amplitude of viral defense, making RNAi crucial for anti-virus defense in plants.

In contrast to the number of reports about DCL characterization and functions in Arabidopsis, only a few reports have characterized them in other plant species. In general, these reports show the presence of 4 classes of DCLs, with more than one member for each class. Seven *DCLs* have been identified in the tomato *Solanum lycopersicum* [36], eight in rice [37], five in maize [38], five in poplar [12, 39], four in grapevine [40], eight in *Brassica napus* [41], three in *Archis duranensis*, and four in chickpea, pigeonpea, *A. ipaensis* [42] and four in pepper [43]. Thus far, cotton DCLs have only been identified in the *G*. *raimondii* genome [44], but they have not been investigated by comparative evolutionary and expression analyses in cotton species.

Cotton crop is important to the economies of more than 30 countries and represent the principal source of natural fiber in the textile industry worldly. Besides cotton fiber, cotton oil is widely used for human consumption. The evolution of the *Gossypium* genus is marked by two important events. First, a divergence occurred 5–10 million years ago (MYA), when A and D diploid genomes form two separated branches. Subsequently, around 1–2 MYA, allopolyploidization events occurred by interspecific hybridization between A and B-genome ancestors resembling *G. arboreum* and *G. raimondii,* respectively, generating new *Gossypium* species, as the cultivated *G. hirsutum* (AADD) between others [44, 45]. As a consequence of the allopolyploidization, thousands of genes were duplicated and showed different expression levels, explaining the drastically phytomorphology changes observed in the allopolyploid cotton, compared to the diploid species *G. arboreum* and *G. raimondii* [44].

Here, we identify and characterize the DCL family of the allotetraploid *G. hirsutum* and two diploids, *G. arboreum* and *G. raimondii,* cotton species. As observed earlier in *G. raimondii* [44], an extra *DCL3* is also present in *G. arboreum* and *G. hirsutum,* where it is currently expressed. Due to the importance of DCL in viral defense, we studied the expression patterns of *G. hirsutum* DCLs in two commercial cotton cultivars (cv.) during biotic stress induced by CLRDV (*Cotton leafroll dwarf virus*) infection and/or herbivore attack by *Aphis gossypii*. Comparison of a CLRDV-resistant and susceptible cotton accession or cv. during infection showed that the modulation of *DCL* expression, especially *DCL2a*, *2b*, and *DCL4*, might explain the contrasting viral responses exhibited by each accession. Interestingly, both cotton *DCL3* were also differentially modulated during viral infection.

## Results

### Identification of cotton Dicer-like gene family and chromosomal localization

Using *Arabidopsis* and rice *DCLs* as query sequences, we identified the *Dicer-like* genes of two diploid cottons, *G. raimondii* and *G. arboreum,* and of the allotetraploid *G. hirsutum acc*. TM-1 were identified by searching the cotton genome database [46–48]. Based on these queries and Blast tool analyses, we identified 6 genes encoding DCL proteins in *G. raimondii*, 7 in *G. arboreum* and 11 in the allotetraploid *G. hirsutum*, respectively (Table 1).

**Table 1:**
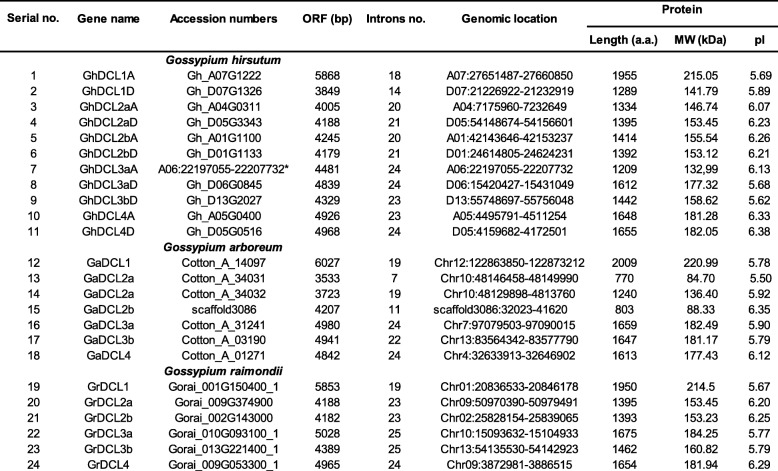
The cotton DCL genes and properties of the deduced proteins

*gene not annotated. The letters A and D after DCL name in *G. hirsutum* indicate the sub-genome A and D where they were identified, respectively. Cotton DCLs parologs were coded as “a” and “b” according to their chromosome position. *ORF* Open reading frame length, *aa* Amino acid, *MW* Molecular weight, *pI* Theoretical isoelectric point

*DCL* genes were named according to the closest orthologs in *A. thaliana*. Among 4 *DCL* genes in *A. thaliana*, *AtDCL1*, *AtDCL2*, *AtDCL3*, and *AtDCL4* have orthologs in cotton. *AtDCL1* has only one ortholog in *G. raimondii* and *G. arboreum,* and two in *G. hirsutum. AtDCL2* has 2 orthologs in *G. raimondii,* 3 in *G. arboreum*, and 4 in *G. hirsutum. AtDCL3* has 2 orthologs in the two diploid genomes and 3 in *G. hirsutum*. All three cotton species present two DCL3. It seems that this duplication could be relevant for cotton evolution as it is present in the three species studied here; however, we cannot exclude the possibility that it is neutral. *AtDCL4* has 2 orthologs in tetraploid cotton, but only 1 ortholog in diploid cottons*.* Cotton DCLs distinct paralogs were coded as a and b, according to their order on the homologous chromosomes.

Detailed information for these genes is listed in Table 1, including the chromosome location, ORF and protein lengths, molecular weight and theoretical isoelectric point. The newly identified *Dicer-like* loci showed coding potentials of 1209 to 2009 amino acid polypeptides, with predicted molecular weights (MW) of 132.99 to 220.99 kDa, respectively. A very small DCL putative polyprotein was identified in *G. arboreum* for DCL2a (Cotton_A_34031), which were 770 amino acids in length (84.7 KDa). It represents an extra DCL2a, that is present only in the *G. arboreum* genome.

**Fig. 1:**
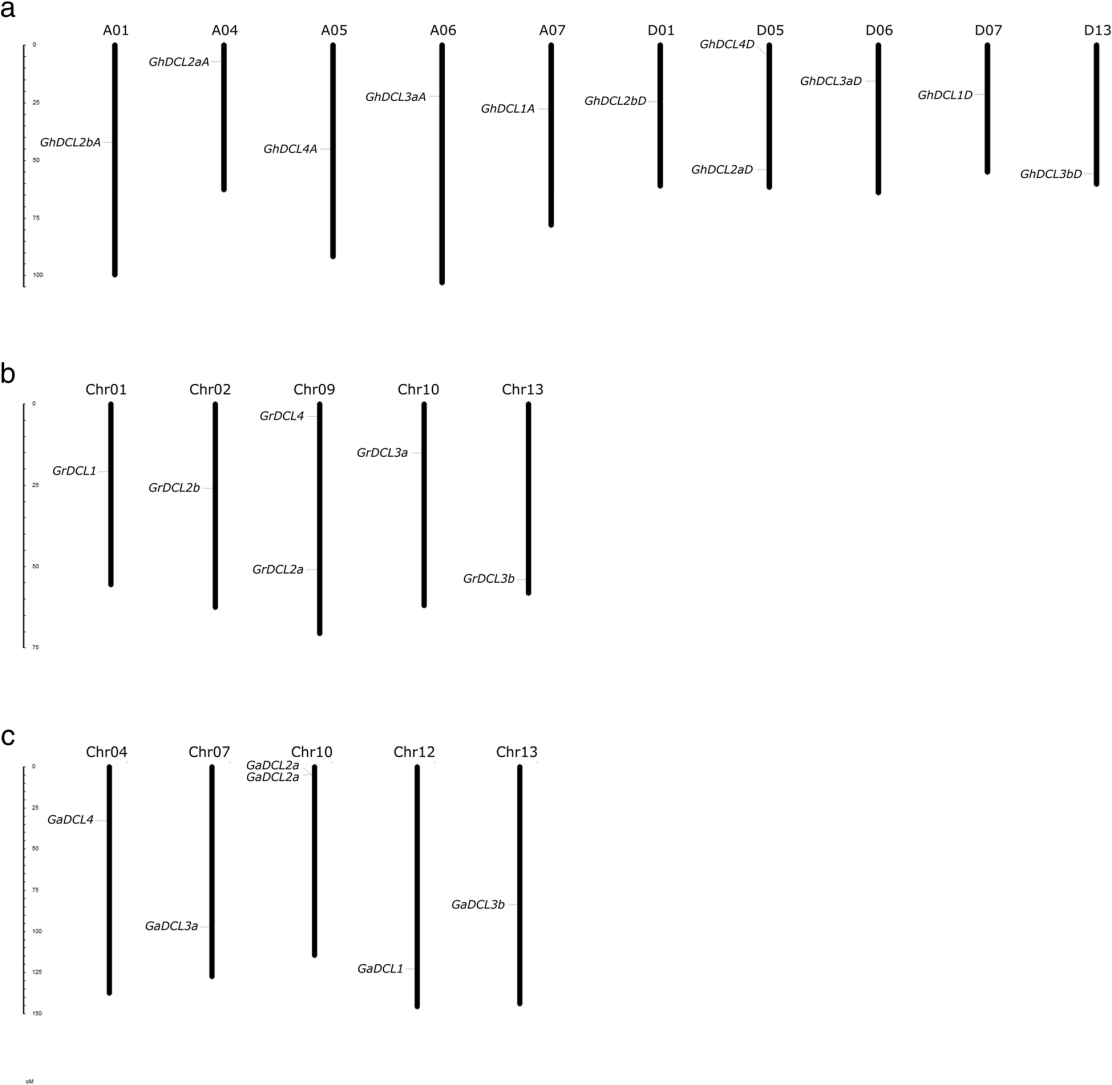
Chromosomal distributions of DCL genes in *Gossypium*. *G. hirsutum* DCLs (*GhDCLs*) are distribute between chromosomes (Chr) A01, A04–07, D01, D05–07 and D13 (**a**); *G. raimondii* DCLs (*GrDCLs*) between chromosomes 01, 02, 09, 10 and 13 (**b**), and *G. arboreum* DCLs (*GaDCLs*) between chromosomes 04, 07, 10, 12 and 13 (**c**), respectively

The physical location of the three cotton species *DCL* genes is shown in Fig. 1. A total of eleven *GhDCL* genes were distributed on 10 *G. hirsutum* chromosomes. All the chromosomes (A01, A04, A05, A06, A07, D01, D06, D07, and D13) contained a single representative of *GhDCL*, with the exception of chromosome D05, which contained *GhDCL2a* and *GhDCL4*. Curiously, almost all the *GhDCLs* were correlated with chromosomes inherited from each parental-related species*. G. raimondii* presented 6 *DCL* genes distributed in 5 chromosomes, and *G. arboreum* 7 had *DCLs* distributed in 5 chromosomes and in a scaffold region (scaffold3086), that had not yet been incorporated into the physical map of chromosomes. *G. arboreum* presented two *DCL2a* located in very close proximity on chromosome 10, separated by approximately 9 kb. These two *DCL2as* (named herein as *GaDCL2a31* and *32*) were highly similar, sharing 93.4% identity at the amino acid level. The predicted *GaDCL2a32* was shorter than its orthologs and seemed to have lost nucleotides/amino acids at the 5′ extremity/N-terminus.

**Fig. 2:**
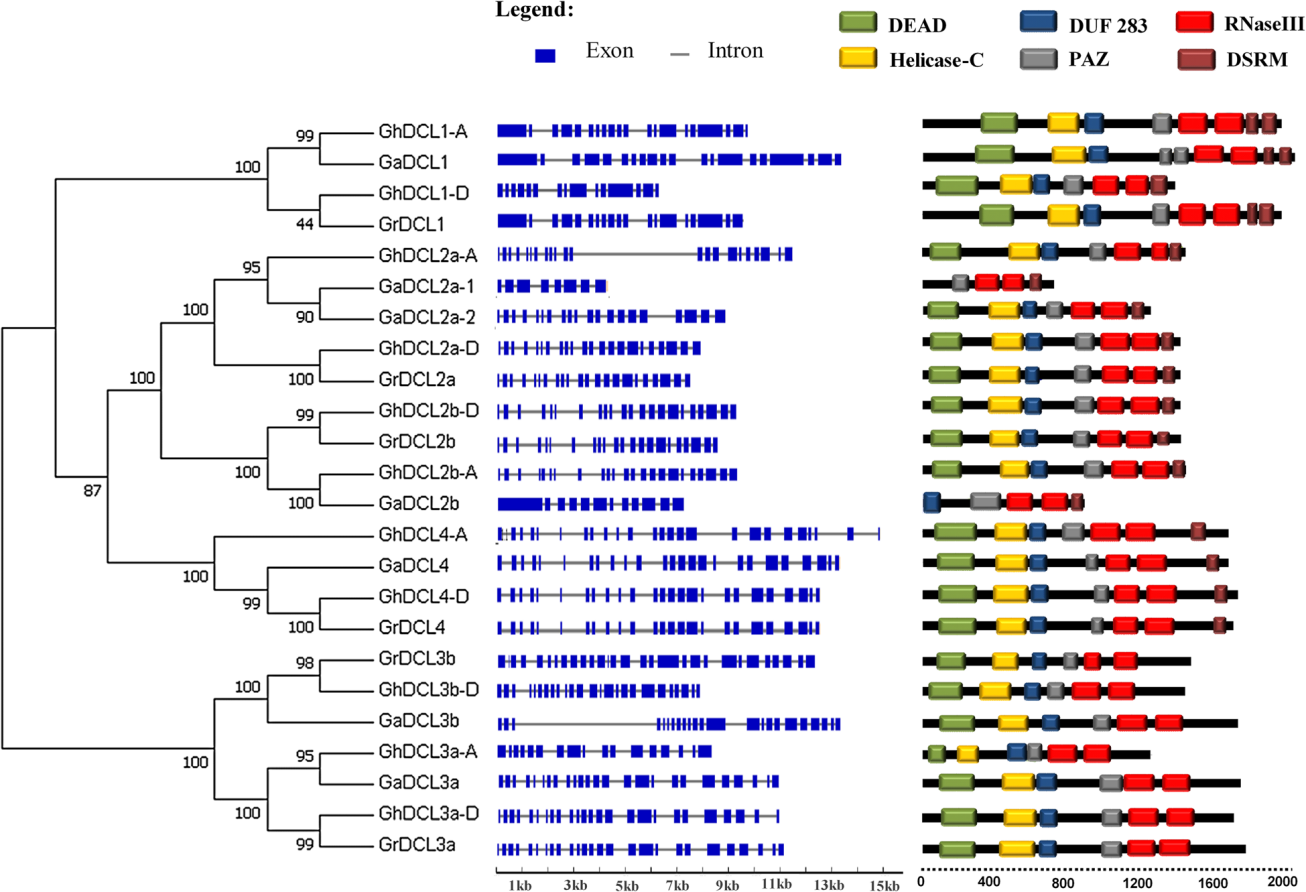
Phylogenetic tree, gene structure and domain compositions of cotton DCLs. The phylogenetic tree was constructed using MEGA 7.0. Exon/intron structures of *DCL* genes are represented by blue boxes and gray lines, respectively. Protein domain analysis are represented by different colors. Sequence used are listed at Additional file 1

The intron/exon (I/E) distribution as well as intron numbers, are shown in Table 1 and Fig. 2. GaDCL1 (Cotton_A_14097) showed the longest ORF of 6027 bp and coding potential for a polypeptide of 2009 amino acids. The maximum number of introns, 25, was found in *DCL3a* and *b* of *G. raimondii*. *DCL4* of all three distinct species showed a very similar intron/exon distribution. The same could be observed for *DCL3a* of *G. arboreum*, *G. raimondii*, and *G. hirsutum* from subgenome D. *GhDCL3a* from subgenome A, however, showed a distinct pattern at the 5′ end of the gene. It seemed to have lost part of the nucleotides from this region. A very similar distribution of introns and exons was also observed for *DCL2b* of *G. raimondii* and *G. hirsutum* subgenomes A and D. *GaDCL2b*, on the other hand, showed a very different distribution of I/E.

### Cotton DCL phylogenetic and domain composition analysis

The phylogenetic relationships of the amino acid sequence of the three cotton species (*G. raimondii, G. arboreum*, and *G. hirsutum)* were used to construct a neighbor-joining (N-J) phylogenetic tree, using MEGA 7.0 software (Fig. 2). These analyses indicated that the *DCL* genes clustered in two separate clades, one composed of *DCL1*, *2*, and *4* and the other of *DCL3s*. The two *GhDCL* subfamilies were further divided into many subclades that clearly grouped the *DCLs* from the parental/ancestral diploid species with each corresponding subgenome in the allotetraploid cotton. The extra *DCL3*, named herein as *DCL3b*, was present in all three species, but the allotetraploid cotton presented only one member of this gene on the genome, showing the closest relationship to *GrDCL3b*. We can hypothesize that the *DCL3b* gene, that was acquired from *G. arboreum* during the *G. hirsutum* evolution, had been lost. Alternately, it may have been acquired independently by the three species after allotetraploid hybridization. The two *DCL2a* from *G. arboreum* formed a group together with *GhDCL2a* from subgenome A, showing that they probably duplicated after tetraploid hybridization. The constructed tree suggested a high level of sequence conservation for *DCL* sequences in the diploid species and the allotetraploid *G. hirsutum* during evolution.

SMART was used to identify the DCL domains in all Dicer-like genes from the three species (Fig. 2). All DCLs contained a DEAD, helicase-C, DUF283 and PAZ domain, excluding GaDCL2a31 and GaDCL2b, which did not present any DEAD or helicase-C domains. In fact, GaDCL2a31 was a truncate DCL because it also did not present a DUF283 domain. Two RNase III domains were present in all DCLs, and at least one DSRM domain was present in 15 out of the 22 cotton DCL proteins. All 7 cotton DCL3s had a dsRB domain, which is a characteristic of DCL3 plant proteins. Interestingly, GhDCL1 from subgenome D seemed to have lost a DSRM domain, while DCL1 of subgenome A maintained 2 DSRB domains.

**Fig. 3:**
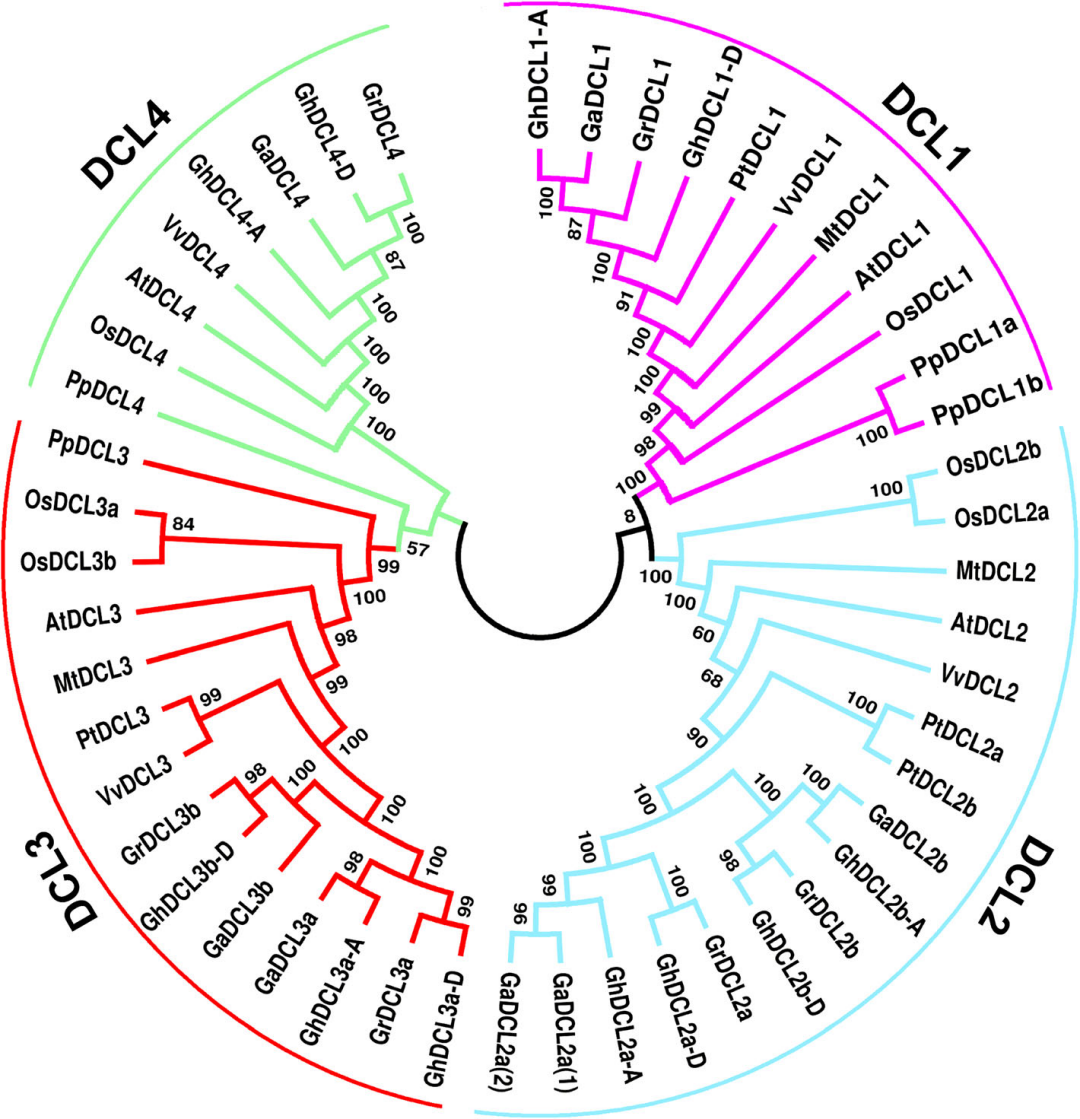
Phylogenetic relationships of DCL proteins from cotton *G. arboreum*, *G. raimondii* and *G. hirsutum*, *A. thaliana (At)*, *O. sativa (Os)*, *V. vinífera* (Vv), *Medicago truncatula* (Mt), *Populus sp. (Po)* and *Physcomitrella patens* (*Pp)*. The unrooted NJ tree was constructed using MEGA 7, and the bootstrap test was performed with 1000 replicates. Sequence used are listed at Additional file 1 and Additional file 2: Table S1

A comparison of cotton DCL proteins with those from Arabidopsis, rice, poplar, grapevine and *Medicago* sp. revealed that cotton DCL1s shared a common ancestor with grapevine and poplar DCL1s (Fig. 3). DCL2a and 2b from cotton, however, were more related to poplar DCL2s. Grapevine and poplar DCL3s showed the closest relationship to cotton DCL3 from all species analyzed. DCL3 duplication in rice seemed to occur over a long time before cotton DCL3 duplication, but after dicot/monocot divergence. The duplication of DCL3 in cotton probably occurred before cotton allotetraploid hybridization and therefore more than 1.2 MYA. DCL4 seemed to be the most conserved DCL among the eudicots analyzed herein.

### DCL gene expression profiles in different *G. hirsutum* organs

To evaluate if the in silico-identified *DCLs* in cotton were able to generate transcripts, we collected tissue samples from root, leaf, stem and flower from greenhouse-grown cotton of two distinct commercial *G. hirsutum* cv., Fibermax 966 (FM966) and Delta Opal (DO), at 60 days post germination (60 dpg) and analyzed gene expression by quantitative real-time PCR (RT-qPCR). These two cultivars were especially selected because they show contrasting phenotypes against an important worldwide distributed cotton virus disease, the cotton blue disease (CBD). Fibermax is susceptible to CBD, while Delta Opal is resistant.

**Fig. 4:**
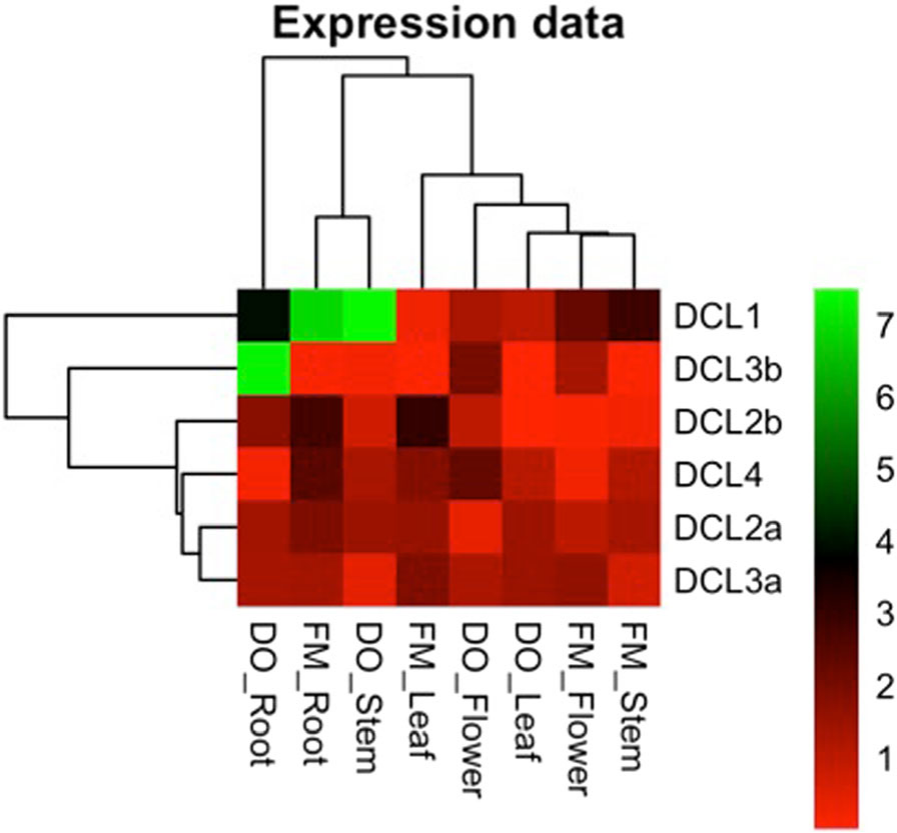
Real-time PCR expression profiling of *Dicer-like* genes in different cotton tissues. *DCL* expressions levels of all cotton *DCLs* were analyzed in stem, flower, leaf and root of two commercial cotton cv. Fibermax 966 (FM) and Delta Opal (DO) at 60 dag by qRT-PCR. The heat map was generated by a log transformation of the real-time PCR data presented as ΔCt (Ct_*DCL*_ – Ct_*GhmiR390*/*GhPP2A*_). The expression values are detailed at Additional file 2: Table S3

As shown in Fig. 4, transcripts of all 6 *DCLs* types were identified in *G. hirsutum* plants. *DCL1* was expressed in all analyzed tissues from both cv. FM and DO, as well as *DCL2a*, *DCL3a*, and *DCL4*. *DCL2b* was not detected in leaves from the DO cv. and flowers of cv. FM, while *DCL3b* was almost undetectable in stems from FM and leaves from both cultivars. *DCL4*, which is essential for intracellular antiviral silencing, was expressed at the same levels in FM and DO plants, as well as *DCL2a*. However, *DCL2b* showed slightly contrasting basal expression levels between them. The extra *DCL3* identified in cotton seemed to be important, especially in flower and root tissues from healthy plants.

### DCL gene expression is modulated in response to herbivore attack and virus infection

Plant DCLs initiate the RNAi innate defense system against invading viruses because they recognize and process incoming viral and transposon nucleic acids into small siRNAs of 21, 22, and 24 nts. Thus, we were interested in shedding some light on how cotton *DCL* expression is modulated during RNA viral infection using virus-resistant and virus-susceptible contrasting cotton cvs.: Delta Opal and FM966, respectively. *Cotton leafroll dwarf virus* (CLRDV) (genus, *Polerovirus*; family, *Luteoviridae*), which is transmitted only by an aphid vector (*Aphis gossypii*), is the causal agent of cotton blue disease [49]. CLRDV is phloem-restricted, and its genome consists of a single-strand, positive-sense, non-polyadenylated RNA (5.8 kb) containing six open reading frames (ORFs) [50].

As CLRDV is only transmitted by its aphid vector, another important point is to understand the aphid vector component in the DCL modulation. Consequently, we evaluated cotton DCL expression patterns during aphid herbivore attack, mediated by *Aphis gossypii* and/or CLRDV infection.

**Fig. 5:**
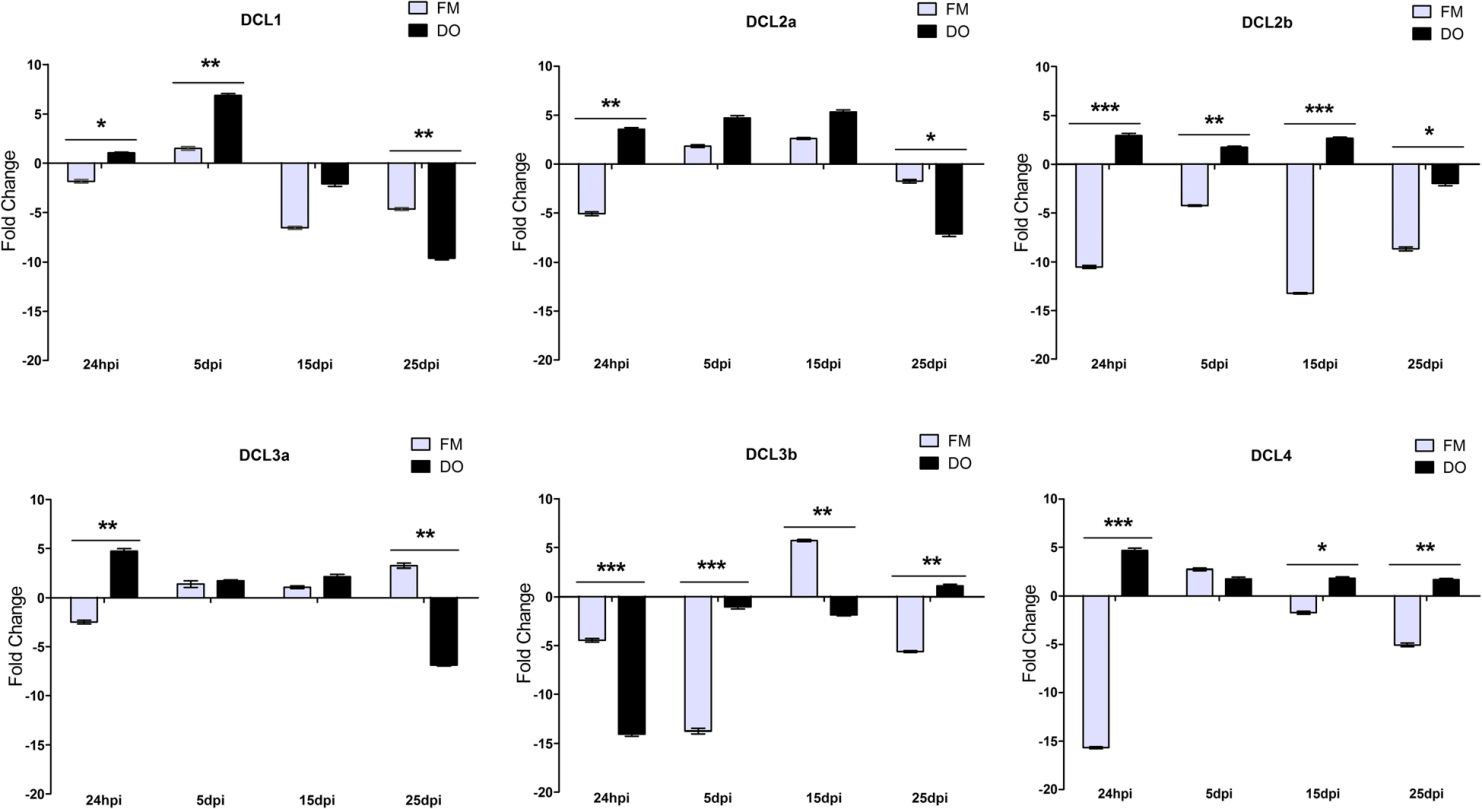
Expression profile of cotton *DCL* analysis by RT-qPCR during herbivore attack. Fibermax 966 and Delta Opal cotton plants were submitted to contact with 10 aphids for 24 h. Systemic leaves were collected at 24 hpi and 5, 15 and 25 dpi for evaluation of each *DCL* level over time. The fold change was calculated using the 2^-ΔΔCt^ method as described by [51]. The standard deviation is indicated by the error bars, and “*” indicates significant differences, calculated using the GraphPad Prism program. * *P* < 0.1 ** *P* < 0.01 *** *P* < 0.001. hpi - hours post-aphid inoculation, dpi - days post-aphid inoculation

To analyze the influence of herbivore attack on DCL expression, 30 dag cotton plants were inoculated in the greenhouse with virus-free aphids. Aphid were restricted to one basal leave (inoculated leave) per plant and 24 h after inoculation, the aphids were eliminated by insecticide application. The expression levels of all cotton DCLs were evaluated in young systemic leaves (3–4 leaves above the inoculated leaves) at 24 hpi, 5, 15, and 25 dpi after contact with the aphids (Fig. 5). For CLRDV infection, a similar biological approach was applied using viruliferous aphids harboring CLRDV.

**Fig. 6:**
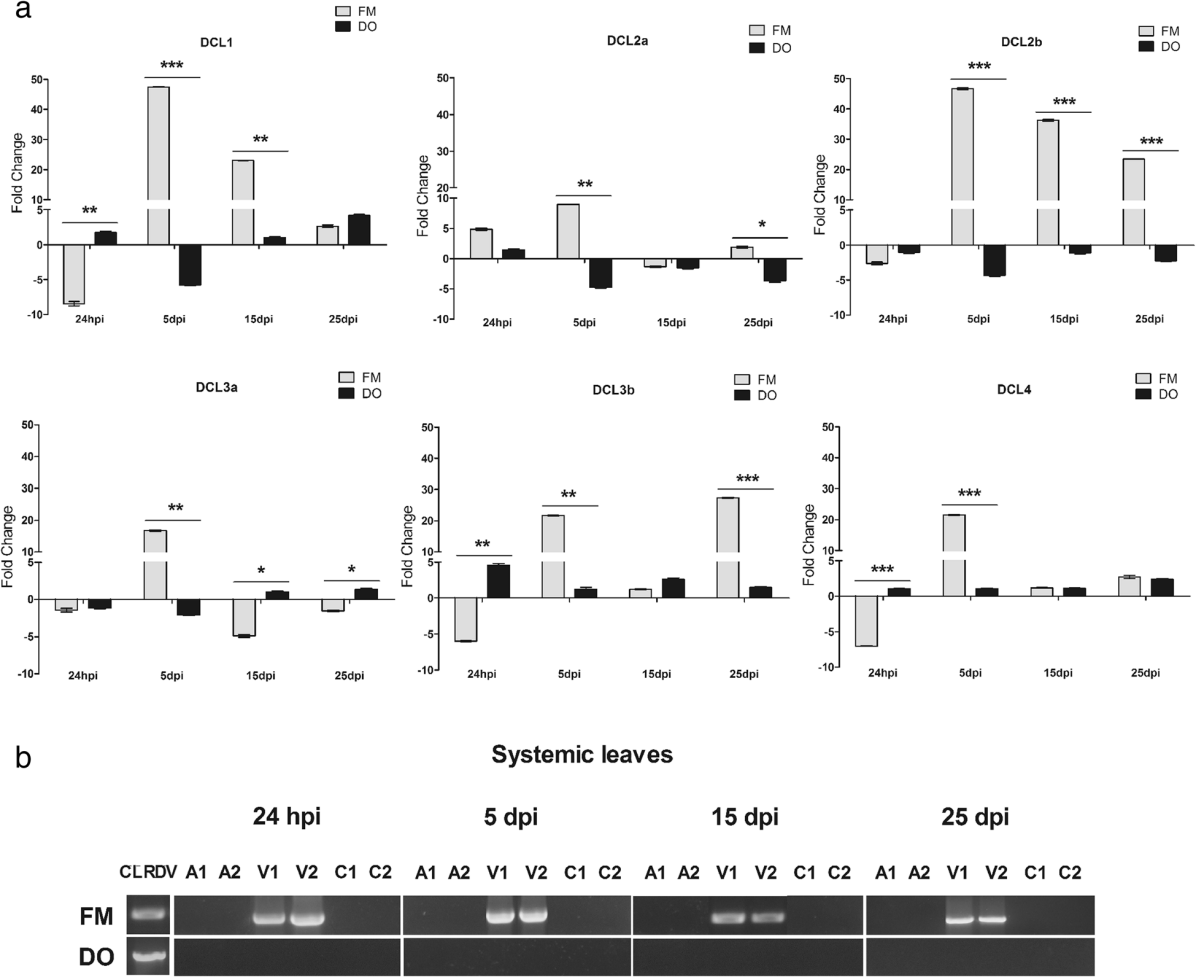
Expression profiles of cotton *DCLs* analyzed by RT-qPCR after CLRDV virus infection. Cotton plants from the cv. Fibermax that is susceptible to CLRDV infection and Delta Opal that is resistant to CLRDV infection, were inoculated with CLRDV vectors. Vectors were eliminated 24 h after inoculation. **a** Systemic leaves of Fibermax 966 and Delta Opal plants were collected at 24 hpi and 5, 15 and 25 dpi for the evaluation of each *DCL* level over time. The fold change was calculated using the 2^-ΔΔCt^ method as described by [52]. The standard deviation is indicated by the error bars, and “*” indicates significant differences, calculated using the GraphPad Prism program. * *P* < 0.1 ** *P* < 0.01 *** *P* < 0.001. hpi - hours post-virus infection, dpi - days post-virus infection. **b** A Nested RT-PCR for CLRDV capsid protein (CP) gene amplification was performed in all systemic leaves to check virus presence in virus-free aphid inoculated (A), CLRDV infected (V) and uninoculated plants (C). The number 1 and 2 represent independent pools of treated plants. FM – CLRDV susceptible plant and DO – CLRDV resistant plant

In general, all *DCLs* showed an induction of their expression levels in the virus-resistant DO plants after aphid contact (Fig. 5). When these plants were subjected to the aphid and virus simultaneously, in the case of viruliferous aphid contact, DO *DCL* levels showed an otherwise repression pattern. In the virus-susceptible cv, the inverse was observed (Fig. 6).

Interestingly, it was observed that after the first 24 hpi a systemic modulation of all *DCLs* mRNAs occurred in both cotton cvs., meaning that aphid feed in inoculated leaves is inducing a systemic response. For FM plants, this systemic response is more pronounced than for DO plants. At 24 hpi, FM plants showed strong down regulation of the three cotton DCLs involved in virus defense, *DCL2a*, *2b* and *4*, with reductions of approximately 5, 10 and 15-fold, respectively. DO plants, in contrast, showed a systemic upregulation of these DCLs. This contrasting modulation of DCLs by aphid feeding may predispose the CBD susceptible FM cv. to be more vulnerable against virus infection, as they have less *DCL2* and *4* accumulation in the incoming virus tissues. Whereas for DO plants, when the virus is trying to spread from the local infection site, it faces a strong antiviral silencing pre-activated in systemic tissues that present 3–5 higher levels of *DCL2a*, *2b* and *4* than healthy plant tissues. The presence of previously systemic accumulation of these DCL induced distally by aphid feeding can help these plants to block the virus infection cycle establishment in new cells far from local infection site.

*DCL1* mRNA expression was also modulated, been induced in systemic leaves of DO cv. 24 hpi and 5 days after aphid contact. At 15 and 25 dpi, *DCL1* expression was reduced in both FM and DO cvs. in comparison to healthy control plants (Fig. 5).

*DCL3a* expression was induced by aphid feeding in both DO and FM cvs. at all time points analyzed. Important exceptions were noted for FM at 24 hpi and for DO at 25 dpi, at which time this DCL was downregulated. In contrast, the cotton extra *DCL3* (*DCL3b*) showed strong repression during the first 24 hpi to 5 dpi in both cvs.

When DO and FM plants were infected with viruliferous aphids (Fig. 6), cotton *DCLs* showed a distinct expression pattern in systemic leaves, compared with those inoculated with virus-free aphids, showing that the presence of virus also modulate *DCL* expression (Fig. 6 and Additional file 2: Figure S2). The fold change analysis between FM CLRDV-infected and FM mock plants (virus-free aphid inoculum) showed that the presence of virus induced an additional systemically down-regulation of almost all their *DCLs* during the initial stages of virus infection (24 hpi), with the exception of *DCL2a*. Five days later, however, the levels of all FM *DCLs* markedly increased, showing almost 60, 10, 47, 17, 28 and 28-fold change variations for *DCL1*, *DCL2a*, *DCL2b*, *DCL3a*, *DCL3b*, and *DCL4*, respectively. In contrast, the *DCL* transcript levels from DO plants at all time points were lightly induced or repressed, maintaining levels that were very similar to uninfected mock plants. Even with the strong DCL modulation observed in the susceptible plant FM, CLRDV could be easily detected in systemic leaves from 24 hpi, showing that even with such extensive efforts of the antiviral machinery, the virus was replicating and spreading throughout the plant (Fig. 6b). This strong modulation of *DCL* expression was not observed in DO plants, in which *DCLs* were only slightly induced or reduced up to 25 dpi, and the virus was not detected in any systemic leaves between 24 hpi and 25 dpi (Fig. 6b). Absence of virus accumulation in DO plants may explain why DO DCLs were almost no induced at these leaves.

Deep sequencing of viral small RNA from CLRDV FM-infected plants, previously performed by our group at this same time point on systemic leaves, showed that the most abundant viral siRNAs were 22 nt [53], highlighting the importance of DCL2 in combating the spread and/or replication of the virus in aerial parts far from inoculated leaves. These efforts are insufficient for the blockade of virus infection. Both DCL2s probably participate in the processing/dicing of viral dsRNA for the generation of these second viral siRNAs of 22 nts. However, as *DCL2b* levels are more than 4 times higher than *DCL2a* levels at the systemic infected leaf cells 5 dpi, we can hypothesize that *DCL2b* is most important for 22-nt viral siRNA generation.

### Modulation of DCL2 and DCL4 expression by virus/aphid infection at local infection sites

**Fig. 7:**
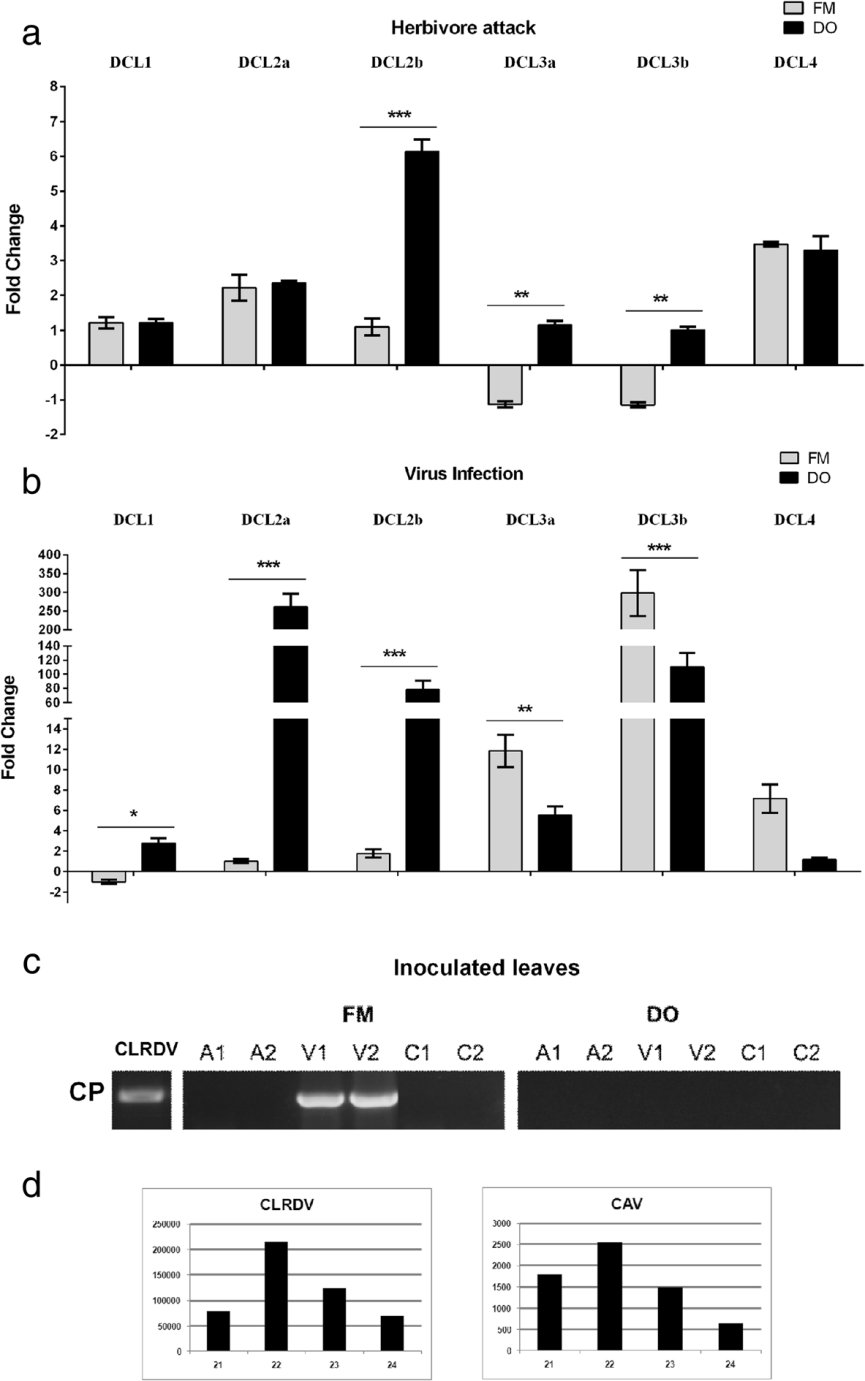
Profiles of cotton *DCL* expression at inoculation sites. **a** *DCL* expression profiles in aphid-inoculated leaves at 24 hpi. **b** Cotton *DCL* expression profiles at 24 hpi with CLRDV. **c** Identification of CLRDV replication in inoculated leaves. Virus replication was assayed by CLRDV coat protein detection using nested RT-PCR in aphid inoculated (A), CLRDV infected and uninoculated plants (C). **d** Profiles of viral small RNAs from cotton plants infected with distinct species of polerovirus. CLRDV sviRNA profile was obtained previously by [54] and is shown here just for illustrative purpose. Numbers 1 and 2 represent independent pools of treated plants. FM – CLRDV susceptible plant and DO – CLRDV resistant plant. CAV – Cotton anthocyanosis virus

It has been already shown for Arabidopsis that DCL4 is an essential component of intracellular antiviral silencing, whereas both DCL4 and DCL2 are necessary for the inhibition of systemic infection. Our results indicated that *DCL4* was almost not modulated by CLRDV infection in aerial parts of virus-resistant cotton during infection, while *DCL2a* and *2b* were downregulated (Fig. 6). In susceptible plants, however, *DCL4* was strongly downregulated systemically at the beginning of infection. Thus, the next step was to examine how these *DCLs* were expressed at the infection site. Consequently, we collected samples from inoculated leaves 24 h after virus infection and analyzed *DCL* mRNA expression profiles by RT-qPCR (Fig. 7).

Aphid contact induced *DCL2a* and *DCL4* expression at similar levels in both FM and DO cvs. (Fig. 7a). *DCL2b* in turn was 6 times more induced in DO than in FM plants. Local aphid attack modulation responses seems to be the same in the resistance/susceptibility phenotype for *DCL4* as both plants showed similar level of induction of this DCL at the inoculated leaves.

The presence of the virus, in contrast, induces stronger modulations of almost all DCLs. At virus infection sites, *DCL2a*, *DCL2b*, and *DCL3b* were strongly induced (approximately 250, 70, and 100-fold change, respectively) in the resistant cv. at 24 hpi (Fig. 7b). The strong *DCL2a* and *2b* induction seemed to be relevant for virus resistance because systemic spread of the virus was completely inhibited in DO plants (Fig. 6b). Virus replication is inhibit even in the virus inoculated leaves, as observed by high sensitivity nested RT_PCR for CLRDV detection (Fig. 7c). The susceptible cv. FM did not respond to virus presence in the same way as the DO cv., as only a very slight induction of *DCL2a* and *b* was observed at 24 hpi in inoculated leaves. In these plants virus accumulation is observed in systemic and inoculated leaves since 24 hpi (Figs. 6b and 7c, respectively).

An induction of FM *DCL4* 6x higher than that of DO *DCL4* was also observed. This finding indicated that both susceptible and resistant plants produced more *DCL4* to combat virus invasion; however, this overexpression alone did not seem to be sufficient to inhibit the spread of infection, since FM plants that produce more *DCL4* than DO are completely susceptible. So, the two cotton DCL2s seem to be very important to avoid virus dissemination and accumulation at inoculation sites while DCL4 may have a secondary paper. However, we cannot say with these results that the strong induction of DCL2 is the responsible for DO CLRDV resistance phenotype as unrelated resistance mechanism may be acting also. Curiously, *DCL3a* and *3b* were highly induced in the FM cv. (11 and 260-fold greater expression than in mock-infected plants and more than 2-fold in DO plants, respectively), while only *DCL3b* was induced in DO cv. (approximately 100-fold greater expression than in the mock). These results showed that *DCL3b* seemed to be important during the initial virus defense activation.

Taken together, our results suggested that the contrasting CLRDV susceptibility phenotype demonstrated by FM and DO plants might be related to their distinct DCL modulation mediated both by aphid feed and virus infection.

Corroborating the importance of cotton *DCL2* in the polerovirus infection, a profile of the vsiRNAs produced by DCL dicing was obtained for CLRDV and another polerovirus infected cotton plants by deep sequencing. As observed in Fig. 7d, in the plants infected with both CLRDV and Cotton anthocyanosis virus (CAV), 22–nt sviRNAs accumulated in major levels than 21-nt sviRNAs showing the relevant paper of cotton DCL2s in the virus defense.

### Biotic and abiotic stress-responsive *cis*-acting regulatory elements are present in the promoters of upland cotton DCL genes

**Fig. 8:**
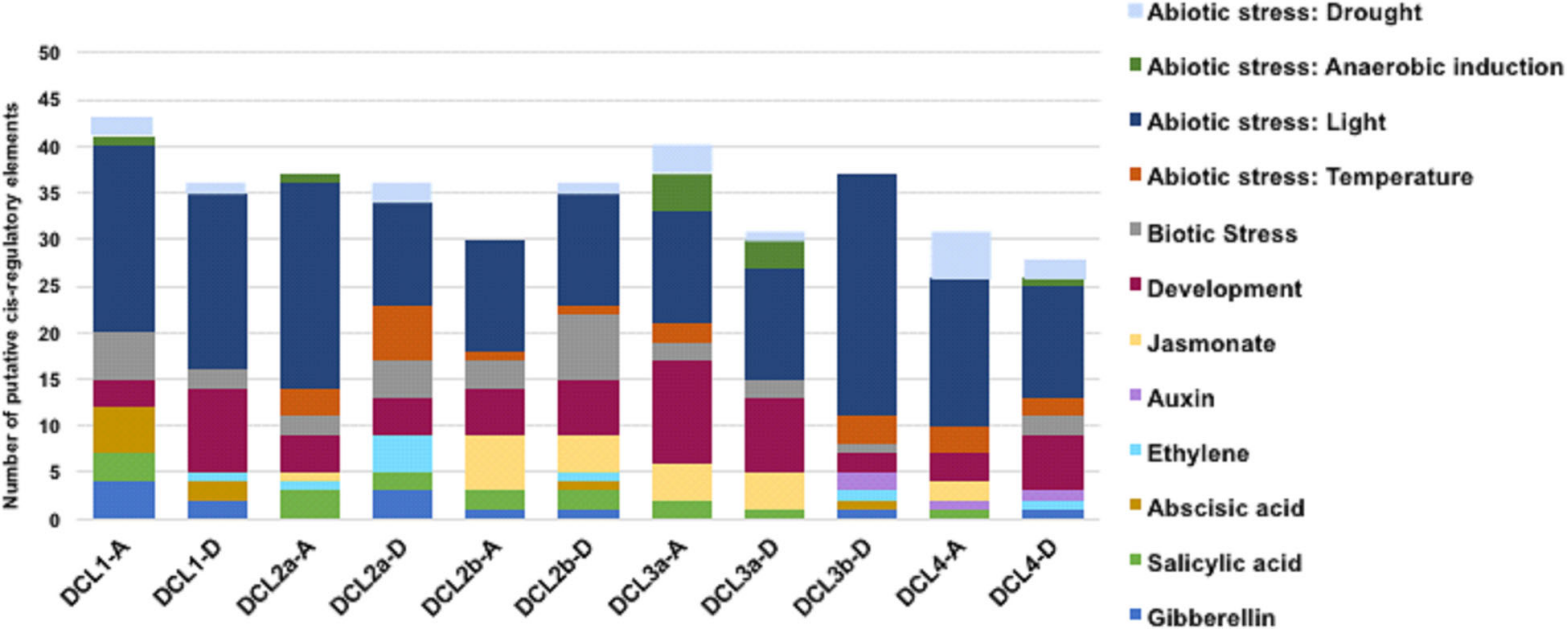
Analysis of *G. hirsutum DCL* promoter sequences. The 1.5-kb upstream promoter sequence of the transcription start site of each *DCL* gene was retrieved and analyzed for the presence of putative *cis*-elements. Different *cis*-elements with the same or similar functions are shown in the same color. Enhancer *cis* elements are shown in Additional file 3: Figure S3

Promoter sequences 1.5 kb upstream of the translation start of all *G. hirsutum DCL* genes were obtained from the cotton genome project to attempt to understand how cotton *DCLs* are modulated by both virus infection and herbivore attack. Transcriptional responsive *cis*-elements of *DCL* gene promoters were analyzed using PlantCare. Analysis of the promoter region of all 11 upland cotton *DCL* genes revealed the presence of various biotic and abiotic stress-responsive cis-acting regulatory elements, including the TCA-element, ERE and ABRE. Light stress-responsive elements were relatively the most abundant in the promoters of the upland cotton *DCL* genes, specifically Box 1, Box 4 and GT1-motif (Fig. 8 and Additional file 3: Figure S3, and Additional file 4), indicating that all DCL proteins might have an important functional role in light stress responses. All *DCL* promoters displayed the development of *cis* elements, especially HD-Zip1 and 2, and almost all the elements that are responsive to drought (especially MBS), salicylic acid (TCA) and other biotic stresses (as WUN, box-W1, ELI-box 3, TC-rich repeats, Box S, JERE, and GCC boxes), revealing possible mechanisms mediated by almost all *DCLs* in drought tolerance and biotic stress responses in the upland cotton *G. hirsutum*. Surprisingly, *GhDCL4A* did not show typical biotic stress or ethylene-responsive cis elements, but they were present in *GhDCL4D*. In general, there were significant differences in the average proportions of the promoter elements detected within the different DCL gene families, as well as between the same *DCLs* originating from a distinct parental diploid cotton (Fig. 8). However, abiotic stress elements presented the highest average proportions in all *DCLs*. Phytohormone-responsive elements, especially those associated with ethylene and gibberellin, were found in *DCL1*, *DCL2a* and *2b*, *DCL3b*, and *DCL4*, while those correlated to Me-jasmonate were predominant in *DCL2b*, *DCL3*, and *DCL4-A*. A large number of enhancer elements were found in all DCLs (Additional file 3: Figure S3), suggesting that all the *DCLs* from the two subgenomes were able to generate transcripts.

## Discussion

In the present study, we first characterized the *DCLs* of 3 cotton species (the commercial allotetraploid *G. hirsutum* and its parental-like *G. arboreum* and *G. raimondii*) at the genomic level. Our results showed that cotton carried extra copies of *DCL2* and *DCL3*. The extra *DCL2* has been previously reported in poplar, rice and maize between other plants. Our phylogenic analysis corroborated other findings, showing that *DCL2* duplication seemed to have evolved independently in mono- and eudicots, after their genetic separation. The *DCL3* duplication shown here is a unique event in eudicots [12]. The extra *DCL3*, denoted herein as *DCL3b*, showed a complete *DCL3* structure in the allotetraploid *G. hirsutum* and in the diploids *G. arboreum* and *G. raimondii.* Furthermore, quantitative RT-PCR showed that it was expressed in distinct cotton tissues/organs such as flowers and roots. The importance of this extra *DCL3* for cotton must be studied, but we can hypothesize that it may be associated with the large number of transposons detected in the cotton genome. *G. raimondii*, *G. arboreum* and *G. hirsutum* genome account approximately to 53, 67 and 62% of retrotransposons, respectively [44]. Curiously, in allotetraploid cotton, we found only one set of *DCL3b*, which probably derived from its *G. raimondii*-like parental species. *DCL3b* from the parental *G. arboreum* seemed to have been missed during evolution.

Analyzing expression of these cotton extra DCL transcripts during virus infection and herbivore attack, we could observe that they seem to be functional and are modulated in both stresses. However, a further characterization of all the cotton DCL2s and DCL3s in heterologous system will be necessary to understand the paper of these DCLs extra copies in RNA silencing machinery.

Previous studies from our group have shown that CLRDV infection in *G. hirsutum* impacts small RNA accumulation both quantitatively and qualitatively [54, 55]. Our deep sequencing identified several cotton miRNAs that were down- or upregulated during CLRDV infection of the susceptible FiberMax 966 *G. hirsutum* cv [56]. All these miRNA families participate in distinct and overlapping networks, such as plant disease resistance (miR393, 403 and 472), leaf development (miR159, 164, 319 and 393), flowering time (miR172, 156 and 157), stress nutrition (miR395, 397, 398 and 408), and miRNA pathways (miR403) [55]. In addition to the global alteration of Gh-miRNAs during CLRDV infection, we observed a decrease in the frequency of 24-nt sRNA in the infected plant with an enrichment of *gypsy-* and *copia-like* retrotransposon transcripts [56]. In contrast, the CLRDV viral siRNA profile during infection revealed a greater abundance of the 22-nt vsiRNAs [54]. Thus, cotton DCLs are anticipated to play an important role in the mediation of CLRDV:cotton interactions during this compatible infection.

Here, the analysis of the expression of the 6 cotton *DCLs* in two contrasting virus-susceptible commercial cotton cvs., during two types of biotic stress, revealed that almost all DCLs were modulated during these stresses. The CLRDV-susceptible cv. FM966 showed a strong downregulation of *DCL2a*, *2b*, *3a*, *3b*, and *4* in the aerial part of the plant during the initial hours after aphid contact. This downregulation was maintained until 25 dpi for *DCL2b*, *DCL2b*, and *DCL4* at the majority of the analyzed time points. For the CLRDV-resistant cv. Delta Opal, this downregulation was not observed, except for *DCL3b,* which was repressed almost 15-fold compared with the mock-infected control plants at 24 hpi (Fig. 5). So, sensing of aphid sucking is able to distinctly modulate *DCL* expression in aerial parts of these two cvs. That were not in contact with the aphid. However, at the inoculated leaves (Fig. 7) *DCL2a* and *DCL4* modulation by herbivore attack was very similar in both cotton cvs. Thus, virus resistance and/or susceptibility did not seem to be a consequence of differential responses to aphid attack at the initial site of aphid contact but was more likely a consequence of the virus versus cv. interaction. Bozorov et al. [57] studied the effects of *N. attenuata DCL* silencing on the performance of *Manduca sexta* larvae. They found that *NaDCL2*, *NaDCL3*, and *NaDCL4* are involved in the regulation of a number of different genes, leading to signaling and defense metabolite activation in response to herbivore attack. Thus, like *N. attenuata DCLs*, cotton *DCLs* are apparently important in anti-herbivore defense activation, as they are clearly modulated locally and especially systemically by aphid sucking.

Antiviral activity of DCLs was already intensively reported ([58–61], among others). Although DCL4 is the major producer of 21-nt antiviral siRNA, DCL2 is essential for secondary 22-nt siRNA-mediated transitive virus silencing and can compensate the loss of DCL4 in *dcl4* mutants [62–65]. During potyvirus *Zucchini yellow mosaic virus* (ZYMV) infection of *N. benthamiana*, for example, DCL4 has been observed to restrict the systemic movement of the virus [66]. The authors observed an enhancement of systemic virus accumulation in double dcl2 and 4 and, particularly, in the triple knockdown mutant dcl2/3/4. Examination of the modulation of *DCL* expression during virus infection by CLRDV showed that the virus induced strong systemic repression of *DCL1*, *2b*, *3b*, and *4* in the susceptible cv. FM966 during the initial hours after infection. This drastic repression was not observed in the resistant cv., in which the expression of all these *DCLs* remained almost similar to that of mock infected plants. Investigation of *DCL* expression modulation at infection sites showed that even FM *DCL4* was expressed 6x higher than DO *DCL4*, and this high induction of *DCL4* was insufficient for virus restriction to the initially infected cells. In contrast, both FM *DCL2a* and *b* were almost not induced in the inoculated leaves. Others authors have reported previously that DCL2 and DCL4 may have different antiviral activities in inoculated and systemic leaves [33, 58, 61]. In these studies it was observed that DCL4 alone responded to the antiviral silencing in inoculated leaves. In systemic leaves, however, both DCL2 and DCL4 are necessary to trigger virus silencing. It has been also previously demonstrated that *DCL4* can downregulate *DCL2* expression [58]. FM *DCL4* overexpression may impair *DCL2a* and *b* expression at the inoculation site in the susceptible cotton cv., allowing the cell-to-cell spread of the virus and establishment of systemic infection due to the noneffective intercellular antiviral activity of DCL2 at these sites. In DO-resistant plants, the high levels of *DCL2a* and *b* induced by the presence of virus may efficiently activate intercellular antiviral silencing, resulting in restriction of the virus to the first cell-autonomous or inoculated cells during the initial hours after infection and dramatically reducing virus replication and aborting infection spread completely.

*DCL2* acts at intercellular VIGS, generating the second front line against viral infection in virus-neighboring recipient cells [58]. CLRDV, similar to all poleroviruses, is able to replicate only in companion cells of the phloem, and the presence of *DCL2* at high levels in the neighborhood of companion cells may impose strong antiviral silencing mediated by the 22-nt virus small RNAs, blocking the spread of the virus in subsequent tissues in resistant DO plants.

Donaire et al. [67] analyzed the vsiRNAs derived from nine viruses belonging to eight different genera. The predominant class of vsiRNAs were 21 nt for most viruses, with the exception of tombusvirus *Cymbidium ring spot virus-*infected plants that accumulated higher levels of 22-nt vsiRNAs. Recently, *Brassica yellows virus* (BrYV) vsiRNA accumulation was analyzed in single-infected *N*. *benthamiana* plants, and the 22-nt class represented the dominant class of BrYV vsiRNAs, followed by the 21-nt class [68]. These results are in agreement with our previous findings [53, 54] and the present studies in which 22-nt vsiRNAs were the most abundant class of vsiRNAs in two other virus species in the polerovirus genus. The accumulation of 22-nt vsiRNAs appears to be characteristic of polerovirus infections, highlighting the important role of DCL2 in antiviral defense against these viruses, which may exceed that of DCL4.

In addition to classical DCL4 and DCL2 antiviral functions, several authors have reported the importance of DCL3 in virus defense via the production of high levels of 24-nt siRNAs from invading viruses [26, 69, 70], guiding AGO family members to cleave viral RNA or recruit cellular machinery to methylate viral DNA. In *A. thaliana*, DCL3 are also responsible for the directed cleavage of positive-sense RNA viruses, including *Cucumber mosaic virus* (a cucumovirus), *Oilseed rape mosaic virus* (a tobamovirus) and *Turnip crinkle virus* (a carmovirus) [26, 71]. Thus, DCL3 also plays an important role in virus silencing. In CLRDV-infected cotton, *GhDCL3a* and *b* were induced on infected leaves (with high levels of expression), as well as systemic leaves, suggesting a possible contribution of these DCLs in viral defense.

It should take in account, however, that ours results to not exclude that other CLRDV-resistance mechanisms not directly related to DCL2/4 modulation should be active in DO plants. These resistance mechanisms may turn down virus replication consequentially decreasing virus imposed stress levels in these plants. One way to deeply check the importance of the DCL modulation observed here by itself in the CLRDV resistance in DO plants would be induce the silencing of each cotton DCL genes individually and/or in combinations in DO cotton plants by VIGS and CLRDV-infect plants the plants further.

## Conclusions

We identified *G. raimondii*, *G. arboreum* and *G. hirsutum* cotton DCLs and found duplication of DCL2 and DCL3. Additionally, we found that all *G. hirsutum* DCLs are involved in polerovirus infection responses, especially the DCL2 and DCL3 paralogs and DCL4, and that modulation of the expression of these DCLs by viruses may be crucial for virus susceptibility and/or resistance response.

## Methods

### Identification of the DCL family in *Gossypium*

The genome sequences of three cotton species, *G. arboreum* (BGI-CGB v2.0 assembly genome), *G. raimondii* (JGI assembly v2.0 data) and *G. hirsutum* acc. TM-1 (NAU-NBI v1.1 assembly genome), were downloaded from the Phytozone (http://www.phytozone.net/) and CottonGen (https://www.cottongen.org/) databases. Dicer-like protein sequence data were obtained for *A. thaliana* and *O. sativa* from the General Feature Format (GFF) le *Arabidopsis* Information Resource (TAIR release 10, http://www.arabidopsis.org) and from the Rice Genome Annotation Project Database (RGAP release 7, http://rice.plantbiology.msu.edu/index.shtml). Gene names and IDs are listed in Additional file 1 and in Additional file 2: Table S1).

The physico-chemical properties of cotton DCL proteins were predicted using the ExPASy Compute pI/Mw tool (http://au.expasy.org/tools/pi_tool.html; Bjellqvistetal Bjellqvistetal, 1993).

### Chromosomal location analysis and phylogenetic tree construction

The locations of the DCLs in chromosomes were assessed using Mapinspect software (http://www.softsea.com/review/MapInspect.html) using start and end position of each open read frame obtained from the genome database. .

A phylogenetic tree was constructed using MUSCLE (Multiple Sequence Comparison by Log-Expectation) alignment and the neighbor-Joining (NJ) method in MEGA 7.0 software [72], with the 1000-replicate bootstrap test. A keyword search of the Phytozome v12.1 database (https://phytozome.jgi.doe.gov/pz) and National Center for Biotechnology Information database - NCBI (https://www.ncbi.nlm.nih.gov) was further performed to obtain the DCL genes in different plant organisms.

### Intron–exon and domain analysis of the DCL family

The Gene Structure Display Server (http://gsds.cbi.pku.edu.cn/index.php) was used to analyze the intron–exon structure by comparing the CDS of cotton *DCL* genes with their corresponding genomic sequences [45, 73]. The conserved domains in the DCLs were identified by SMART (http://smart.embl-heidelberg.de).

### Plant materials

Two upland cotton *G. hirsutum* cultivars were used for the *DCL* expression assays and viral infection: acc. FiberMax966 (FM966) (Aventis Crop Science, Australia) and acc. Delta Opal (DO) (Delta and Pine Land Co., United States). Seeds were kindly provided by IMA, Instituto Matogrossense do Algodão, Primavera do Leste, Mato Grosso state, Brazil. Plantlets were grown under greenhouse conditions at 28 +/− 2 °C as previously described [49].

For cotton organ *DCL* expression, samples of leaves, shoots, flowers, and roots from independent plants at 30 days after germination (dag) were collected from each cotton acc. FM and DO. Samples were immediately frozen in liquid N_2_ and stored at − 80 °C until RNA extraction.

### Plant aphid inoculation and virus infection

Cotton (*Gossypium hirsutum*) plants of cultivars acc. FM966 (susceptible to Cotton blue disease) and acc. DO (resistant to Cotton blue disease) grown in the greenhouse and at the 30 dag stage were infected with *Cotton leafroll dwarf virus* (CLRDV, polerovirus, *Luteoviridae* family) by viruliferous aphids (*Aphis gossypii* Glover) as described previously [56]. Full-developed leaves of plants with the same age from both cultivars were inoculated with non-viruliferous aphids (virus free aphids). Aphids were restricted at the inoculation site by double-side adhesive tapes (3MM Co.) and killed 24 h after inoculation with insecticide. Aphids were restricted to the inoculation sites at the inoculated leaves by surrounding the inoculation site with a double face tap.

Systemic leaves, localized 3–4 leaves above the inoculated leaf, were collected at 24 h post-infection (hpi) and 5, 15 and 25 days post-infection (dpi) with CLRDV aphids or non-viruliferous aphids. Leaves from the same position of uninfected plants were used as uninoculated controls. Leaves from 3 to 5 independent inoculated plants composed each biological replicate. Samples were stored at − 80 °C until RNA isolation and expression analysis.

All the samples recovered from aphid-free inoculated, CLRDV infected and uninoculated were assayed for CLRDV detection by a Nested RT-PCR assay that amplifies viral coat protein sequence following [53]. CLRDV susceptible infected plants were used as control.

### Real-time quantitative RT-PCR (RT-qPCR)

Total RNA was isolated using the Invisorb Spin Plant RNA Kit (Invitek Molecular Co., Germany). The quality and concentration of each RNA samples was determined by agarose gel electrophoresis and using a NanoDrop 2000 (Thermo Fisher Scientific Co.) spectrophotometer. Only RNA samples with a 260/280 nm ratio between 1.8–2.1 and 260/230 ratio ≥ 2.0 were used. The c-DNA first-strand reverse transcription was conducted with the Revertaid First Strand cDNA, Synthesis Kit (Thermo Fisher Scientific Co.) in accordance with the manufacturer’s instructions. One microgram of total RNA from each sample was treated with DNAse I (Fermentas Co.), and cDNAs were synthetized by adding 100 μM of oligo dT24V primer. *G. hirsutum DCL* reverse and forward primers were designed using NCBI primer-BLAST (Additional file 2: Table S2). Maxima SYBR Green/ROX qPCR Master Mix (Thermo Fisher Scientific Co.) was used to perform RT-qPCR in a 7500 Fast Real-Time PCR System (Applied Biosystems), in accordance with the manufacturer’s instructions. The cycling conditions were 10 min at 95 °C for initial denaturation, followed by 40 cycles of denaturation at 95 °C for 15 s and annealing/extension at 60 °C for 30 s. Cotton *GhmiR390* and *GhPP2A* (catalytic subunit of phosphatase 2A) were used as reference genes for the analysis of *DCL* expression in different organs, the results were obtained by △CT method and the 2^−△△CT^ method was used to analyze *DCL* expression during viral infection and/or aphid inoculation [51, 52]. Cotton *GhmiR390* and *GhPP2A* (PROTEIN PHOSPHATASE 2), which were previously identified as the best reference genes for CLRDV infected cotton studies [74], were used to normalize cDNA expression levels. Reactions were prepared in a total volume of 20 μL, which contained 10 μL of SYBR green master mix, 2 μL of cDNA template, 6 μL of ddH_2_O, and 2 μL of each primer to make a final concentration of 10 μM. Three biological replicates were performed, and three technical replicates were assayed per cDNA sample.

In order to show DCL expression in each organ, a heat-map of the *DCLs* expression pattern was established by complete clustering method analysis using Euclidean distance and calculated in the R software environment. The DCL Cts results are shown at Additional file 2: Table S3.

### Sequencing of CAV sviRNAs by deep sequencing

Leaves of cotton infected with Cotton anthocyanosis virus (CAV) obtained in Mato Grosso state, Brazil, were used for total RNA extraction and small RNA purification and sequencing by Illumina plataform following procedures described by [54].

### Statistical analysis

*DCL* relative expression levels determined for the different samples under herbivore attack and/or virus infection were compared with these controls: untreated plants (control) x aphid inoculated plants for herbivore expression analysis, and mock (virus-free aphids inoculated plants) x CLRDV-aphid inoculated plants for viral infection using the parametric one-way ANOVA test at *P* ≤ 0.05 and *P* ≤ 0.01. For checking if the different relative expression levels of DCLs, from FM and DO, are statistical significant T-student test were used.

### Promoter region *cis*-acting element analysis

For the identification of cis-regulatory elements present in *G. hirsutum DCLs,* upstream sequences of 1500 nucleotides from ATG star codon were downloaded from the CottonGen website (https://www.cottongen.org) and cis elements predicted with PlantCARE software (http://bioinformatics.psb.ugent.be/webtools/plantcare/html/).

## Supplementary information

**Additional file 1.** Cotton DCL c-DNA and protein sequences.

**Additional file 2: Table S1.** Gene name and gene ID of DCLs in *Arabidopsis*, *Medicago*, rice, *Populus*, *Physcomitrella* and grapevine. **Table S2.** List of primers and amplicon characteristics of *DCL* genes. **Table S3.** DCL expression in organs. **Figure S1.** DCL amplification melting curves. **Figure S2.** Expression pattern of *G. hirsutum* acc. FM DCL genes based on transcriptome sequencing data.

**Additional file 3: Figure S3.** *Cis*-elements distribution in DCL promoters with enhancer elements. **Figure S4.** Promoter sequences of *Gossypium hirsutum* DCLs.

**Additional file 4. **Positions of all *cis* elements found in *G. hirustum* DCLs promoters.

## Data Availability

All data generated or analysed during this study are included in this published article and in its supplementary information files.

## Abbreviations

CLRDV: *Cotton leafroll dwarf virus*
DCL: Dicer-like protein
DO: Delta Opal cotton cultivar
FM: Fibermax966 cotton cultivar
GaDCL: Dicer-like gene or protein from *Gossypium arboreum*
GhDCL: Dicer-like gene or protein from *Gossypium hirsutum*
GrDCL: Dicer-like gene or protein from *Gossypium raimondii*
RNAi: RNA interference
vsiRNA: Viral small interfering RNAs
