# Supplementary material for: Genome-wide identification of the Dicer-like family in cotton and analysis of the DCL expression modulation in response to biotic stress in two contrasting commercial cultivars
Source: BMC Plant Biol. 2019 Nov 15;19:503. doi: 10.1186/s12870-019-2112-4 (PMC6858778; doi:10.1186/s12870-019-2112-4)
Supplement: Supplementary file 1 — Additional file 1. Cotton DCL c-DNA and protein sequences. [file 12870_2019_2112_MOESM1_ESM.docx]

**Additional file 1**

**Cotton DCL c-DNA and protein sequences**

***Gossypium hirsutum***

**>Gh_A07G1222_NBI-AD1_v1.1 ID=Gh_A07G1222_ *Gossypium hirsutum*|mRNA| length=5868bp_DCL1**

ATGGAGGAAGAAGGTAGGGTTTCTGGTGGCAATGGGTCATCTTACTGGCTGGATGCTTGTGAGGACATATCATGCGACTTGATTAGCGATTTTGTTGATTTCGATGCTCCTATAGTTCAAGACTCCGTTGACAATACTCCAATCAAGATTTCTTTGGAGGAATTGATCACATTCTCGATAGCTTCAAGAACGGCGGGGGACTTCCTCCGGTGGGAAGTAACGGTGATAGCTCTGCCGTCAATGGAAATGGAATTTACGACCCCGTTGCTGGAGATGGATGGTCTCCCAATGAGCTCTCTGGGGTCTCCAAGGATTTACCTGACAACTCAGTTCCGCCATCTAATGGAGTTGAAAAGAAGATTGGAAGCAAAGGGCAGGAAAAAAGCTGCGATGATAGTAATTCCAGTTTATTTGATTATTCTAATAAGGATAATGGAGTTCATCGAGACGATAAAAGGTCATCCGAGTCGAGAGATAGGGGTTTAGATAGTGAAGAGAGGTGTAGAAAGAGGGCTCGTGCGAATGGCTGCAAGAGTGATAGGCAGTATTCTAGTAGAGGTCAATATTATCCCCGGGATAGGGAGAGATGTTCTTCTAGGAAAAGGGTTCGAGATTGGGATGAAATTGATCGGAGAGATAGAGAGCATGTTAGGAGAAGAGAACATTATTATGGTGGCAATAGGAGGGATGGGAGAGAGAGAGAACCAAGGGGTTATTGGGAGAGGGATCGGTCAGGGTCCAATGAGATGGTTTTTAGGTTGGGTACTTGGGAAGCAGATCGGCAAAGAGAAGGGAAGGTGGCTTATGACAAAACTCCCGAGTGCAATGGAAAGATGGAGAAGAAAGTTGAGCAGCCGAAGGAAAAACTCTTGGAGGAGCAAGCTCGTCAATATCAATTGGATGTTCTTGAGCAGGCAAAGAGGAAAAACACAATTGCATTTCTTGAAACCGGGGCAGGGAAGACCCTCATTGCTGTTCTCCTCATGAAAAGTATTTCTGATGATTTACAAAAGCACACCAGAAAAATGCTCTCTGTCTTTTTGGTTCCTAAAGTTCCACTAGTTTATCAGCAAGCTGAAGTTATTCGCGAGAGAACTGGTTTCCAAGTAGGTCATTATTGTGGGGAGATGGGTCAAGATTTTTGGGATGCTCGTAGGTGGCAGCGCGAGTTTGAATCAAAGCAGGTTTTGGTTATGACAGCTCAAATTCTGCTGAATATTTTGAGACACAGCATAATTAAAATGGAATCAATCAATCTCCTTATCCTGGACGAGTGTCATCATGCTGTGAAGAAACATCCGTATTCACTGGTTATGTCTGAATTCTATCATACAACACCAAAGGAGAAGGACCTTCTGTTTTTGGAATGACCGCTTCTCCTGTTAACTTGAAGGGTGTTTCAAGTCAAGTTGATTGTGCTATAAAAATTCGTAATCTAGAAAGCAAACTGGATTCTGTAGTCTGTACCATCAAAGACCGCAAGGAACTCGAGAAACATGTGCCAATGCCTTCAGAAGTTGTGGTAGAGTATGACAAAGCAGCCAGTTTATGGTCCCTCCATGAACAAATAAAACAAATGGAAGCAACAGTTGAAGAAGCTGCACAATCAAGCTCTAGAAGAAGTAAATGGCAGTTCATGGGAGCTAGAGATGCAGGAGCCAAGGAAGAGCTTCACCAAGTTTATGGTGTATCTGAAAGAACAGAAAGTGATGGAGCTGCTAATTTGATACAAAAGTTGAGGGCTGTTAATTATGCACTGGGTGAACTGGGCCAATGGTGTGCTTATAAGGTTGCACAATCTTTTCTGACGGCTTTGCAAAATGATGAGAGGGCAAACTACCAGCTTGATGTCAAGTTTCAAGAATCTTACCTAAACAAAGTTGTTTCTCTCTTACAATGCCAATTATCAGAGGGAGCTGTTACTGAAAAAGACATGAATAATGCAGAAGCAGAGAACTGTAATGCTCAAGATGGGACCAATACTGATGAGATTGAGGAAGGAGAGCTCCCTGACAGTCATGTTGTCTCTGGTGGAGAGCATGTGGATGTGATAATTGGAGCGGCTGTAGCAGATGGAAAAGTGACCCCGAAAGTACAGTCATTGATTAAAATACTTCTGAAGTATCAGCACACAGAGGATTTTCGAGCAATCATCTTTGTTGAGCGAGTTGTGGCTGCTTTAGTTCTTCCTAAGGTTTTTGCAGAGCTTCCGTCTCTGAGTTTCATCAGGTGTGCAAGTTTAATTGGGCACAACAATAGTCAAGAAATGCGGACTGGACAAATGCAGGATACAATTGCTAAATTCCGTGATGGTCGTGTGACATTGTTAGTTGCAACTAGTGTTGCTGAGGAAGGATTGGATATTCGGCAATGCAATGTTGTCATTCGTTTTGATCTTGCAAAAACTGTTTTGGCATACATTCAGTCTAGAGGTCGTGCAAGGAAGCCTGGGTCAGATTACATCTTGATGGTTGAGAGAGGAAATTTATCGCATGCTACATTCCTAAGGAATGCTAGGAATAGTGAGGAGACCTTGCGGAAAGAAGCAATTGAGAGAACTGACCTTAGTCATTTGAAGGATACTTCGAGGTTGATTTCGGTGGATATGGTACCAGGTACGGTGTACCAGGTTGAATCAACTGGTGCCATTGTTAGCTTGAATTCTGCTGTTGGACTCGTCCATTTTTACTGCTCTCAGCTTCCTAGTGACAGATATTCAATACTTCGTCCCGAGTTTATTATGAAGAAGCATGAGAAGCCAGGGGGTCCAACTGAATATTCTTGCAAGCTTCAGCTCCCCTGTAATGCACCATTTGAAGAGCTCGAGGGTCCCATGTGCAGTTCTATGCGTCTTGCCCAGCAGGCATGCTATACTGCTGTATGTTTGGCTGCTTGCAAGAAGCTCCATGAGATGGGAGCATTTACTGATATGCTCTTGCCCGACAAAGGAAGCGGGGAAGAAGCAGAGAAGGTTGACCAGAATGATGAAGGAGACCCACTTCCTGGAACTGCTAGGCATAGAGAATTCTATCCAGAAGGTGTAGCAGATATACTCCAGGGAGAATGGATTTTATCTGGAAGAGATTGTGTTGGTGACTCCAAAATACATCGTCTGTACATGTATACTATCAAATGTGTAAATAATGGCTCTTCAAAAGATCCATTCTTAACTAAAGTCTCAGATTTTGCAGTACTTTTTGGCAAAGAGCTGGATGCAGAGGTGTTATCGATGTCGGTGGATCTATTTATCGTTCGAGCCATGATAACAAAGGCATCTCTTGTCTTCAGGGGATCAATAGATATAACTGAAAGTCAGATGGCATCCCTTAAAAATTTTCACGTAAGATTGATGAGCATTGTATTGGATGTGGATGTTGATCCTGCCACTACTCCTTGGGATCCTGCCAAGGCATATTTGTTTGTCCCTGTGGTTGGCGATAAGTTTGTAGATCCTATAAAAGAAGTTGATTGGGATTTGGTAGATAATATAATCACTACAAATGCGTGGAGCAATCCCCTTCAGAGAGCTAGGCCAGATGTTTTCCTTGGGACAAATGAGAGGACACTTGGTGGTGACAGAAGGGAGTATGGATTTGGGAAATTGCGTCATGGCCTGGCTTTTGGGCACAAACCTCATCCTACTTATGGTATCAGAGGAGCCGTAGCCCCATTTGATGTTGTTAAAGCTACCGGGGTGGTTCCTAGTCGTGATACGATTGAGGTACAAGGGGATTGGACCAAAGGCAATTTGATAATGGCTGATGGTGTTGCACGTGCAGAAGATCTTGTTGGAAGAATAATAACAGCCGCTCATTCGGGGAAGAGGTTTTATGTAGATACAATATGCTATGACATGTCAGCAGAGACCTCCTTTCCAAGGAAAGAGGGCTATCTTGGTCCTGTCGAGTACAGCTCATATGCCGATTACTATAAGCTGAAGTATGGTGTTGAGTTGAGCTGCAAGCAACAAGCTTTGATAAGAGGTCGTGGTGTTTCATACTGCAAGAATCTCTTATCTCCTCGATTTGAGCACTCAGAAGGTGAATCGGAGGAGGCACTTGACAAAACATACTACGTGTTTCTTCCGCCTGAGCTATGTTTTGTTCATCCACTTCCTGGATCACTTGTTAGAGGTGCCCAGAGGTTGCCCTCTATTATGAGGAGGGTTGAGAGCATGCTTCTTGCAATTCAACTTAAGCACATAATACAATTTCCGGTCCCTGCTTCAAAGATTTTGGAAGCTTTGACTGCTGCTTCGTGTCAGGAGACATTTTGCTATGAAAGGGCTGAGCTTCTTGGGGATGCTTACTTGAAATGGGTAGTCAGTCGTTTTCTGTTTCTTAAATATCCCCAGAAACATGAAGGTCAACTGACCAGGATGAGACAACAAATGGTGAGTAACATGGTATTGTATCAATATGCATTAAATAAGGGACTTCAATCATACATCCAAGCAGATCGCTTTGCACCATCTAGATGGGCTGCTCCTGGGGTGTTGCCAGTCTTTGATGAGGATACAAAAGATGGTGACATGTCCTTATTTGATCAAGAACATGCAACTGCTGATGTTTTACCAGTAAAAGTACTTGGTGATGGGTTTGAAGATGAGGATATGGAAGATGGTGAGATTGAGAGTGACTCAAGTTCTTATAGAGTCCTCTCTAGCAAGACCTTAGCGGATGTGGTTGAAGCACTGATTGGAGTTTATTATGTTGAAGGTGGCAAGCATGCAGCTAACCACCTCATGAAATGGATTGGGATCCAGGTGGAGTCTGATCCTGATGATATGGACTCTATAGTGAAGCCATCGAATGTTCCAGAAAGCATACTCAGGAGTGTAAATTTTGAGGCCTTAGAAGGTGCATTGAACATCGAGTTTAAAAATAGGGCCTTGTTGGTAGAAGCTATTACTCATGCTTCACGCCCATCATCGGGAGTATCCTGCTACCAGCGTTTGGAGTTTGTTGGTGATGCAGTCTTGGATCATCTTATCACAAGACATTTGTTTTTTACATACACCAATTTGCCTCCAGGTCGCTTGACTGATTTGCGTGCTGCTGCCGTAAACAATGAAAACTTTGCACGTGTTGCTGTAAAGCATCAGTTGCACGTGCATCTTCGGCATGGATCAAGTGCCCTTGAGAAACAGATTCGGGACTTCGTGAAGGAAGTTCAGGATGAGTTATTAAAGCCAGGTTTCAACTCTTTTGGATTGGGAGATTGCAAAGCACCGAAAGTTCTTGGAGATATTGTTGAATCCATTGCTGGTGCCATTTTTCTTGACAGTGGACGTGACACTGCAGTTGTCTGGAGGGTTTTTCAACCTCTGTTGCATCCCATGGTGACTCCAGAGACATTGCCGATGCATCCTGTCCGGGAACTACAAGAACGATGCCAGCAACAAGCTGAAGGCTTGGAATACAAAGCCAGTCGTAGTGGCAATTTGGCCACTGTGGAGGTTTTCATTGATGGGGTCCAGGTTGGAGTTGCTCAGAATCCCCAAAAGAAGATGGCACAGAAACTAGCTGCAAGGAATGCACTTGCTGTTCTGAAGGAGAAAGAAACAGCTGAAGCTAAGGAGAACTGTGAGGAGAATGGGAAAAAGAAGAAGAATGGCAACCAGACATTTACCAGGCAAACATTGAATGATATCTGCCTGCGTCGAAACTGGCCTATGCCCTTCTATCGGTGTGTGAATGAAGGGGGCCCTGCCCATGCAAAGAGATTTACTTTTGCTGTCAAGGTTAACACCACTGACAGGGGGTGGACCGATGAATGCATAGGTGAGCCAATGCCTAGTGTTAAGAAGGCCAAGGACTCGGCCGCTGTGCTTCTCTTGGAACTTTTAAACAAATGGTATTCATGA

**>Gh_A07G1222_DCL1_protein sequence length=1955aa**

MEEEGRVSGGNGSSYWLDACEDISCDLISDFVDFDAPIVQDSVDNTSNQDFFGGIDHILDSFKNGGGLPPVGSNGDSSAVNGNGIYDPVAGDGWSPNELSGVSKDLPDNSVPPSNGVEKKIGSKGQEKSCDDSNSSLFDYSNKDNGVHRDDKRSSESRDRGLDSEERCRKRARANGCKSDRQYSSRGQYYPRDRERCSSRKRVRDWDEIDRRDREHVRRREHYYGGNRRDGREREPRGYWERDRSGSNEMVFRLGTWEADRQREGKVAYDKTPECNGKMEKKVEQPKEKLLEEQARQYQLDVLEQAKRKNTIAFLETGAGKTLIAVLLMKSISDDLQKHTRKMLSVFLVPKVPLVYQQAEVIRERTGFQVGHYCGEMGQDFWDARRWQREFESKQVLVMTAQILLNILRHSIIKMESINLLILDECHHAVKKHPYSLVMSEFYHTTPKEKRPSVFGMTASPVNLKGVSSQVDCAIKIRNLESKLDSVVCTIKDRKELEKHVPMPSEVVVEYDKAASLWSLHEQIKQMEATVEEAAQSSSRRSKWQFMGARDAGAKEELHQVYGVSERTESDGAANLIQKLRAVNYALGELGQWCAYKVAQSFLTALQNDERANYQLDVKFQESYLNKVVSLLQCQLSEGAVTEKDMNNAEAENCNAQDGTNTDEIEEGELPDSHVVSGGEHVDVIIGAAVADGKVTPKVQSLIKILLKYQHTEDFRAIIFVERVVAALVLPKVFAELPSLSFIRCASLIGHNNSQEMRTGQMQDTIAKFRDGRVTLLVATSVAEEGLDIRQCNVVIRFDLAKTVLAYIQSRGRARKPGSDYILMVERGNLSHATFLRNARNSEETLRKEAIERTDLSHLKDTSRLISVDMVPGTVYQVESTGAIVSLNSAVGLVHFYCSQLPSDRYSILRPEFIMKKHEKPGGPTEYSCKLQLPCNAPFEELEGPMCSSMRLAQQACYTAVCLAACKKLHEMGAFTDMLLPDKGSGEEAEKVDQNDEGDPLPGTARHREFYPEGVADILQGEWILSGRDCVGDSKIHRLYMYTIKCVNNGSSKDPFLTKVSDFAVLFGKELDAEVLSMSVDLFIVRAMITKASLVFRGSIDITESQMASLKNFHVRLMSIVLDVDVDPATTPWDPAKAYLFVPVVGDKFVDPIKEVDWDLVDNIITTNAWSNPLQRARPDVFLGTNERTLGGDRREYGFGKLRHGLAFGHKPHPTYGIRGAVAPFDVVKATGVVPSRDTIEVQGDWTKGNLIMADGVARAEDLVGRIITAAHSGKRFYVDTICYDMSAETSFPRKEGYLGPVEYSSYADYYKLKYGVELSCKQQALIRGRGVSYCKNLLSPRFEHSEGESEEALDKTYYVFLPPELCFVHPLPGSLVRGAQRLPSIMRRVESMLLAIQLKHIIQFPVPASKILEALTAASCQETFCYERAELLGDAYLKWVVSRFLFLKYPQKHEGQLTRMRQQMVSNMVLYQYALNKGLQSYIQADRFAPSRWAAPGVLPVFDEDTKDGDMSLFDQEHATADVLPVKVLGDGFEDEDMEDGEIESDSSSYRVLSSKTLADVVEALIGVYYVEGGKHAANHLMKWIGIQVESDPDDMDSIVKPSNVPESILRSVNFEALEGALNIEFKNRALLVEAITHASRPSSGVSCYQRLEFVGDAVLDHLITRHLFFTYTNLPPGRLTDLRAAAVNNENFARVAVKHQLHVHLRHGSSALEKQIRDFVKEVQDELLKPGFNSFGLGDCKAPKVLGDIVESIAGAIFLDSGRDTAVVWRVFQPLLHPMVTPETLPMHPVRELQERCQQQAEGLEYKASRSGNLATVEVFIDGVQVGVAQNPQKKMAQKLAARNALAVLKEKETAEAKENCEENGKKKKNGNQTFTRQTLNDICLRRNWPMPFYRCVNEGGPAHAKRFTFAVKVNTTDRGWTDECIGEPMPSVKKAKDSAAVLLLELLNKWYS

>**Gh_D07G1326_NBI-AD1_v1.1 ID=Gh_D07G1326_NBI-AD1_v1.1_ *Gossypium hirsutum*| mRNA|length=3849bp_DCL1**

ATGTGCCGTTTTGTCATAGTTGTCTCTGGTGGAGAGCATGTGGATGTGATCATTGGAGCGGCTGTAGCAGATGGAAAAGTGACCCCGAAAGTACAGTCATTGATTAAAATACTTCTGAAGTATCAGCACACAGAGGATTTTCGAGCAATCATCTTTGTTGAGCGAGTTGTGGCTGCTTTAGTTCTTCCTAAGGTTTTTGCAGAGCTTCCGTCTCTGAGTTTCATCAGGTGTGCAAGTTTAATTGGGCACAACAATAGTCAAGAAATGCGGACCGGACAAATGCAGGATACAATTGCTAAATTCCGTGATGGTCGTGTGACATTGTTAGTTGCAACTAGTGTTGCTGAGGAAGGATTGGATATTCGGCAATGCAATGTTGTCATTCGTTTTGATCTTGCAAAAACTGTTTTGGCATACATTCAGTCTAGAGGTCGTGCAAGGAAGCCTGGGTCAGATTACATCTTGATGGTTGAGAGAGGAAATTTATCGCATGCTACATTCCTAAGGAATGCGAGGAATAGTGAGGAGACCTTGCGGAAAGAAGCAATTGAGAGAACCGACCTTAGTCATTTGAAGGATACTTCGAGGTTGATTTCGGTGGATATGGTACCAGGTACTGTGTACCAGGTTGAATCAACTGGTGCCATTGTTAGCTTGAATTCTGCTGTTGGACTCGTCCATTTTTACTGCTCTCAGCTTCCTAGTGACAGATATTCAATACTTCGTCCCGAGTTTATTATGAAGAAGCATGAGAAGCCAGGGGGTCCAACTGAATATTCTTGCAAGCTTCAGCTTCCCTGTAATGCACCATTTGAAGAGCTCGAGGGTCCCATGTGCAGTTCTATGCGTCTTGCCCAGCAGGCTGTATGTTTGGCTGCTTGCAAGAAGCTCCATGAGATGGGAGCATTTACTGATATGCTCTTGCCCGACAAAGGAAGCGGGGAAGAAGCAGAGAAGGTTGACCAGAATGATGAAGGAGACCCACTTCCTGGAACTGCTAGGCATAGAGAATTCTATCCAGAAGGTGTAGCAGATATACTCCAGGGAGAATGGATTTTATCTGGAAGAGATGGTGTTGATGACTCCAAAATACATCGTCTGTACATGTATACTATCAAATGTGTAAATAATGGCTCTTCAAAAGATCCATTCTTAACTAAAGTCTCAGATTTTGCAGTACTATTTGGCAAAGAGCTGGATGCAGAGGTGTTATCGATGTCGGTGGATCTATTTATCGTTCGAGCCATGATAACAAAGGCATCTCTTGTCTTCAGGGGATCAATAGATATAACTGAAAGTCAGATTTCATCCCTTAAAAATTTTCACGTAAGATTGATGAGCATTGTATTGGATGTGGATGTTGATCCTGCCACTACTCCTTGGGATCCTGCCAAGGCATATTTGTTTGTCCCTGTGGTTGGCGATAAGTTTGTAGATCCTATAAAAGAAGTTGATTGGGATTTGGTAGATAATATAATCACTACAAATGCGTGGAGCAATCCCCTTCAGAGAGCTAGGCCAGATGTTTTCCTTGGGACAAATGAGAGGACACTTGGTGGTGACAGAAGGGAGTACGGATTTGGGAAATTGCGTCATGGCCTGGCTTTTGGGCACAAACCTCATCCTACTTATGGTATCAGAGGAGCTGTAGCCCCATTTGATGTTGTTAAAGCTACCGGGGTGGTTCCTAGCCGTGATACGAACGAGGTACAAGGGGATTGGACCAAAGGCAATTTGATAATGGCTGATGGTGTTGCACGTGCAGAAGATCTTGTTGGAAGAATAATAACAGCCGCTCATTCGGGGAAGAGGTTTTATGTAGATACAATATGCTATGACATGTCAGCAGAGACCTCCTTTCCGAGGAAAGAGGGCTATCTTGGTCCTGTCGAGTACAGCTCATATGCCGATTACTATAAGCTGAAGTATGGTGTTGAGTTGAGCTGCAAGCAACAAGCTTTGATAAGAGGTCGTGGTGTTTCATACTGCAAGAATCTCTTATCTCCTCGATTTGAGCACTCAGAAGGTGAATCGGAGGAGGCACTTGACAAAACATACTACGTGTTTCTTCCGCCTGAGCTATGTTTTGTTCATCCACTTCCTGGATCACTTGTTAGAGGTGCCCAGAGGTTGCCCTCTATTATGAGGAGGGTTGAGAGCATGCTTCTTTCAATTCAACTTAAGCACATAATACAATTTCCGGTCCCTGCTTCAAAGATTTTGGAAGCTTTGACTGCTGCTTCGTGTCAGGAGACATTTTGCTATGAAAGGGCTGAGCTTCTTGGGGATGCTTACTTGAAATGGGTAGTCAGTCGTTTTCTGTTTCTTAAATATCCCCAGAAACATGAAGGTCAACTGACCAGGATGAGACAACAAATGGTGAGTAACATGGTATTGTATCAATATGCATTAAATAAGGGACTTCAATCATACATCCAAGCAGATCGCTTTGCACCATCTAGATGGGCTGCTCCTGGGGTGTTGCCAGTCTTTGACGAGGATACAAAAGATGGTGACACGTCCTTATTTGATCAAGAACATGCAACTGCTGATGTTTTACCAGTAAAAGTACATGGTGATGGGTTTGAAGATGAAGATATGGAAGATGGTGAGATTGAGAGTGACTCAAGTTCTTATAGAGTCCTCTCTAGCAAGACCTTAGCAGATGTGGTTGAAGCACTGATTGGAGTTTATTATGTTGAAGGTGGCAAGCATGCAGCTAACCACCTCATGAAATGGATTGGGATCCAGGTGGAGTCTGATCCTGATGAGATGGACTCTATAGTGAAGCCATCTAATGTTCCAGAAAGCATACTCAGGAGTGTAAATTTTGAGGCCTTAGAAGGTGCATTGAAAATCGAGTTTAAAAATAGGGCCGTGTTGGTAGAAGCTATTACTCATGCTTCACGCCCATCATCGGGAGTATCCTGCTACCAGCGGTTGGAGTTTGTTGGTGATGCAGTCTTGGATCATCTTATCACAAGACATTTGTTTTTTACATACACCAATTTGCCTCCAGGTCGCTTGACTGATTTGCGTGCTGCTGCCGTAAACAATGAAAACTTTGCACGTGTTGCAGTAAAGCACCTGTTGCATGTGCACCTTCGGCATGGATCAAGTGCCCTTGAGAAACAGATTCGGGACTTCGTGAAGGAAGTTCAGGATGAGTTATTAAAGCCGGGTTTCAACTCTTTTGGATTGGGAGATTGCAAAGCACCAAAAGTTCTTGGAGATATTGTTGAATCCATTGCTGGTGCCATTTTTCTCGACAGTGGACGTGACACTGCAGTTGTCTGGAGGGTTTTTCAACCTCTGTTGCATCCCATGGTGACTCCAGAGACATTGCCGATGCATCCTGTCCGGGAACTACAAGAACGATGCCAGCAACAAGCTGAAGGCTTGGAATACAAAGCCAGTCGTAGTGGCAATTTGGCCACTGTGGAGGTTTTCATTGATGGGGTCCAGGTTGGAGTTGCTCAGAATCCCCAAAAGAAGATGGCACAGAAACTAGCTGCAAGGAATGCACTTGCTGTTCTGAAGGAGAAAGAAACAGCTGAAGCTAAGGAGAACTGTGAGGAGAATGGGAAAAAGAAGAATGGCAACCAGACATTTACCAGACAAACATTGAATGATATCTGCCTGCGTCGAAACTGGCCTATGCCCTTCTATCGGTGTGTGAATGAAGGGGGCCCTGCCCATGCAAAGAGATTTACTTTTGCTGTCAAGGTTAACACCACTGACAGGGGGTGGACCGATGAATGCATAGGTGAGCCAATGCCTAGTGTTAAGAAGGCCAAGGACTCAGCCGCTGTGCTTCTCTTGGAACTTTTAAACAAATGGTATTCATGA

**>Gh_D07G1326_ DCL1_protein sequence length=1289aa**

MCRFVIVVSGGEHVDVIIGAAVADGKVTPKVQSLIKILLKYQHTEDFRAIIFVERVVAALVLPKVFAELPSLSFIRCASLIGHNNSQEMRTGQMQDTIAKFRDGRVTLLVATSVAEEGLDIRQCNVVIRFDLAKTVLAYIQSRGRARKPGSDYILMVERGNLSHATFLRNARNSEETLRKEAIERTDLSHLKDTSRLISVDMVPGTVYQVESTGAIVSLNSAVGLVHFYCSQLPSDRYSILRPEFIMKKHEKPGGPTEYSCKLQLPCNAPFEELEGPMCSSMRLAQQAVCLAACKKLHEMGAFTDMLLPDKGSGEEAEKVDQNDEGDPLPGTARHREFYPEGVADILQGEWILSGRDGVDDSKIHRLYMYTIKCVNNGSSKDPFLTKVSDFAVLFGKELDAEVLSMSVDLFIVRAMITKASLVFRGSIDITESQISSLKNFHVRLMSIVLDVDVDPATTPWDPAKAYLFVPVVGDKFVDPIKEVDWDLVDNIITTNAWSNPLQRARPDVFLGTNERTLGGDRREYGFGKLRHGLAFGHKPHPTYGIRGAVAPFDVVKATGVVPSRDTNEVQGDWTKGNLIMADGVARAEDLVGRIITAAHSGKRFYVDTICYDMSAETSFPRKEGYLGPVEYSSYADYYKLKYGVELSCKQQALIRGRGVSYCKNLLSPRFEHSEGESEEALDKTYYVFLPPELCFVHPLPGSLVRGAQRLPSIMRRVESMLLSIQLKHIIQFPVPASKILEALTAASCQETFCYERAELLGDAYLKWVVSRFLFLKYPQKHEGQLTRMRQQMVSNMVLYQYALNKGLQSYIQADRFAPSRWAAPGVLPVFDEDTKDGDTSLFDQEHATADVLPVKVHGDGFEDEDMEDGEIESDSSSYRVLSSKTLADVVEALIGVYYVEGGKHAANHLMKWIGIQVESDPDEMDSIVKPSNVPESILRSVNFEALEGALKIEFKNRAVLVEAITHASRPSSGVSCYQRLEFVGDAVLDHLITRHLFFTYTNLPPGRLTDLRAAAVNNENFARVAVKHLLHVHLRHGSSALEKQIRDFVKEVQDELLKPGFNSFGLGDCKAPKVLGDIVESIAGAIFLDSGRDTAVVWRVFQPLLHPMVTPETLPMHPVRELQERCQQQAEGLEYKASRSGNLATVEVFIDGVQVGVAQNPQKKMAQKLAARNALAVLKEKETAEAKENCEENGKKKNGNQTFTRQTLNDICLRRNWPMPFYRCVNEGGPAHAKRFTFAVKVNTTDRGWTDECIGEPMPSVKKAKDSAAVLLLELLNKWYS

**>Gh_A04G0311_NBI-AD1_v1.1_ID=Gh_A04G0311_NBI-AD1_v1.1_*Gossypium hirsutum* | mRNA|length=4005bp_DCL2a**

ATGGAGCCAGTTGACATGGAGGAGGATATTTCTCAACTACTCCCTGCTGATCCTCTTCCTTTTGCCAGAAGCTACCAGCTTGAAGCATTAGAGAAAGCTATCAAGCAAAACACAATAACTTACTTGGAAACTGGATCTGGCAAGACAATGATCGCCATCATGCTTCTCCGCAGCTATGCCCATCTTATTCGCAAGCCCTCACCTTTTTTTGCTGTCTTCTTGGTTCCCAAAGTTGTCCTGGTTAAACAACAAGCTGATGCTGTGGAAATGCATACGGACTTGAATGTTGGAAAGTATTGGGGACATATGCAGGTCGACTTTTGGGATGGGGAGAAGTGGAAACAAGAAATAGATAAATATGAGGTGCTTGTGATGACACCTCAAATTTTACTTGATGGATTGAGGCATCGCTTCTTCAATATAAACATGATAAAGGTTTTGATAATTGATGAATGCCATCATGCCCGAGGAAAGCATCCTTATGCCTCTATTATGAGAGAATTCTATCATCGCCCGTTAGAAGCTGGTGCATCTAATCTTCCTAGGATTTTTGGGATGACTGCTTCTCCTATAAATTCAAAAGGGGCAAACTCTGCTGATAGCTATTGGCAGAAGATCCATGAATTGGAGACTATTATGAACTCAAAGGTGTATACGTGTGTAAGTGCATCAGTGCTTGCTCAGTTTGTTCCATTTGCGACTCCAAAGTTCAAGTTTTACCAACATATGGCAATTCCAAATGTTTTATATTCACACTTGGTAGAGGAATTGACTGTTTTGAAAGTAAAGCATGAATGTTCGTTGGATAATTTGGATCTTGAAGCTTCTGCAGCAGAATCTACAAGAAAGAAACTATCAAAGATACATTCAGCTTTAATACATTGTTTATATGAGCTTGGTGCTTGGTTGGCTCTGAAGGCTGCAGAGTGCTTTTCATGTTATGAAAGTGAGCTTCTTATGTGGGGGAAATTGGATGTCTTTGGTGAGAAAATTATTAGGAGCTACAGCGTGGATGCTTTTCGTGCAATTGAAACATGCATGCCATCTGGTCTGGATTGGACCATAGCTAATGATGTTAAAGGCAGTGTGGCTGCTGGGTTTCTTACCACAAAAGTTTTATGCCTTATTGAATCTTTATTCGAATACAGGGTGTTGAAGGACATAAGATGTATAATTTTTGTTGAGAGGGTTATAACAGCTGTTGTGCTTCAATCGCTATTCAGAGAATTGCTTCCCAGGTACAGTAATTGGAAGACTAAGTACATTGCGGGAAATAACTCTGGATTGCAGAATCAGACAAGGAAAAAACAAAATGAAATTGTAGAAGAATTCCGCAAAGGCATGGTTAACATAATCGTTGCAACTTCAATTCTTGAAGAAGGCTTGGATGTTCAATCATGCAACCTAATTATCAGATTTGATCCTTCACCAACAATTTGTAGTTTCATACAGTCTCGAGGACGTGCTAGAATGCAGAACTCAGATTATCTATTAATGCTGAAGAGCGGGGATTTTTCTACACATTCTCGACTGAAGAACTATCTTACTAGTGGAGATGTAATGAGAAAGGAATCTTTATGCCATGCATCATATCCTTGTTCTCCTCTTAGTGAGGGCTTAGATGATGAAGAGTTTTACCATGTTGTGAGTACAGGGGCAAGTATGACTCTTAGTTCTAGTGTTGGTCTAATGTACTTCTATTGCTCACGTCTCCCTGCAGATGGGTATTTTAAACCTATTCCGAGGTGTGTTATAGACAAGCAAATGGGGCTTTGCAACCTCCTTCTACCCAAGAGTTGCCCTATACAAACTGTTTGTGTTCAGGGTGATATAAAAAACATAAAGAAAAAAGCATGCTTTGAAGCATGCAAGAAACTCCATCAAATTGGTGCTTTAACAGACAATCTTGTTCCTGATATTGTTTTTGAAGAACATGATGTGGAAGAAATTGAAAAGGAGCCCTATAATGATGATCAACCTATTTTCTTTCCAACTGAACTAGTGAATAAGGGTTCACTGGACTCCATGACAAAGTACTACTGCTACTTAATGGAGTTGAAGCAGAACTTTGATTATGAGGTTCCTGTTCATAACATCATGCTTCTTGTCAGGAATCAGTTTGACATGGATGAAAAAAGTGTGAATATTGAGTTAGAAGTTGACAGGGGCACATTAACAGTTAGCATGAAATATATTGGACTAATACGTCTTAATTCTGACCAGGTTATCTTATGTAGAAGGTTTCAGCTTGCAGTTTTTCAAGTACTTATGGATCGTAAAGCTGAAAAGTTGGCAGAGGTGTTATGTGACCATACCTTGGGGAATAATTCTGAAATTGATTATCTACTCCTCCCATCAAATTATGTGGGCCAGAGTCCTCTGATTGATTGGTTGTCAGTTACTTCTGTCACATTTTCTTATGAAAAGGCCTGCAAGAATCATGTGAACTGCAATGCTGATATGATACAGATCAAAAGCGGTCTGGTGTGCACTTGCATGATCCAAAATTCGTTGGTCTCTACTCCTCATAATGGTCATGCTTATATTATCAGTGGTCTTTTAACTAATATAAATGCAAATTCACTTTTGAGGTTGAGTGATGGACGCTTAATGACTTACAAGGAGTATTATGAACAACGCCACGGTATCAATTTATGTTATAGTCAAGTCTCTTTCCTTGCTGGGCAACATATTTTTCCCGTGCAGAATCACATTCAAAGGTGCAGAAAACAGAAAGAGAAAGAATCAAGTAACGCATTGGTAGAATTGCCTCCTGAGCTTTGTTGTGTAGTAATGTCTCCCATATCGATTAGCACATTTTATTCTTTCACATTTCTTCCATCAATCATGCATCGACTCGAGTCTTTGCTCCTTGCTACCAGCTTGAAAAAGATGCATTTGGATCATTGCGTGCAAAATGTTGCCATTCCAACTATGAAGGTTTTGGAGGCAATTACTACCAAAAAGTGCCTAGAAAACTTTCATTTGGAATCACTAGAGACTCTTGGTGACTCTTTCTTGAAGTATGCTGTTTGTCAACAGCTTTTCAAAAAATATCCAAATCATCACGAGGGCCTTCTTAGTATTAGGAAGGAAAAAATTATTTCAAATACAGCTCTGTCCATGCTAGGATGTGACAAGAAACTTCCGGGGTTTATCCGTGATGAGCCTTTCGATCCCAAAGACTGGATGATTCCTGGTTATAATTGTGGAAGTTACTCATTAAATGAGGAAACACTGTGTAATGCAAAAAAAATATATGTTAGAGGAAGAAGAAAGCTGAAGTTTAAGAAGGTTGCTGATGTTGTTGAGGCACTTATTGGTGCATACCTTAGCACGGGAGGCGAAGCAGCTGGATTACTATTTTTGGATTGGATCGGTATAAGTATTAATTTCACAAATATACCATATGAAAGACATTTTAAAGTGCGGGCCGAGAAGTTTGTTAATGTCCAGCACTTTGAGTCCCTTCTACACTATTCATTCCAAGACCCTTCTTTGTTGGTGGAAGCATTAACCCATGGTTCTTATATGCTTGCTGAAATTCCAGGATGTTATCAGAAGCTTTACAAGGATATAAAAGAAACCGTCGAAAGTTTTCAGGAATTATCATTGGAATACACTTTCGGCTGGGAATCTGAAAAATCTTTCCCCCAGGTACTTGCTGACGTGATGGAGTCACTTGCTGGAGCTATTTTTGTTGATTCAGGATACAATAAGGAAATCGTATTCCAGAGTATAAGGCCACTGTTGGAGCCCATGATTACTCTGGAGACTATGACGGTTCAACCTGTCAAAGAGCTATATGAGCTCTGTCAAAAGGAGCATTATGACCTAAGAAAACCCATTGTTTCACATGAGGATGGCATTTCTTCTATTACGATAGAGGTTGAAGCAAATGGGAAGGTATTCAAGCACACATCCACAGCTTGTGATAAGGAGATGGCCAAGAAACTGGCTTCCAAAGAAGTTTTGAAGTCTCTAAAGGGAGCCAATTTTAGCTAG

**>Gh_A04G0311_DCL2a_protein sequence length= 1334aa**

MEPVDMEEDISQLLPADPLPFARSYQLEALEKAIKQNTITYLETGSGKTMIAIMLLRSYAHLIRKPSPFFAVFLVPKVVLVKQQADAVEMHTDLNVGKYWGHMQVDFWDGEKWKQEIDKYEVLVMTPQILLDGLRHRFFNINMIKVLIIDECHHARGKHPYASIMREFYHRPLEAGASNLPRIFGMTASPINSKGANSADSYWQKIHELETIMNSKVYTCVSASVLAQFVPFATPKFKFYQHMAIPNVLYSHLVEELTVLKVKHECSLDNLDLEASAAESTRKKLSKIHSALIHCLYELGAWLALKAAECFSCYESELLMWGKLDVFGEKIIRSYSVDAFRAIETCMPSGLDWTIANDVKGSVAAGFLTTKVLCLIESLFEYRVLKDIRCIIFVERVITAVVLQSLFRELLPRYSNWKTKYIAGNNSGLQNQTRKKQNEIVEEFRKGMVNIIVATSILEEGLDVQSCNLIIRFDPSPTICSFIQSRGRARMQNSDYLLMLKSGDFSTHSRLKNYLTSGDVMRKESLCHASYPCSPLSEGLDDEEFYHVVSTGASMTLSSSVGLMYFYCSRLPADGYFKPIPRCVIDKQMGLCNLLLPKSCPIQTVCVQGDIKNIKKKACFEACKKLHQIGALTDNLVPDIVFEEHDVEEIEKEPYNDDQPIFFPTELVNKGSLDSMTKYYCYLMELKQNFDYEVPVHNIMLLVRNQFDMDEKSVNIELEVDRGTLTVSMKYIGLIRLNSDQVILCRRFQLAVFQVLMDRKAEKLAEVLCDHTLGNNSEIDYLLLPSNYVGQSPLIDWLSVTSVTFSYEKACKNHVNCNADMIQIKSGLVCTCMIQNSLVSTPHNGHAYIISGLLTNINANSLLRLSDGRLMTYKEYYEQRHGINLCYSQVSFLAGQHIFPVQNHIQRCRKQKEKESSNALVELPPELCCVVMSPISISTFYSFTFLPSIMHRLESLLLATSLKKMHLDHCVQNVAIPTMKVLEAITTKKCLENFHLESLETLGDSFLKYAVCQQLFKKYPNHHEGLLSIRKEKIISNTALSMLGCDKKLPGFIRDEPFDPKDWMIPGYNCGSYSLNEETLCNAKKIYVRGRRKLKFKKVADVVEALIGAYLSTGGEAAGLLFLDWIGISINFTNIPYERHFKVRAEKFVNVQHFESLLHYSFQDPSLLVEALTHGSYMLAEIPGCYQKLYKDIKETVESFQELSLEYTFGWESEKSFPQVLADVMESLAGAIFVDSGYNKEIVFQSIRPLLEPMITLETMTVQPVKELYELCQKEHYDLRKPIVSHEDGISSITIEVEANGKVFKHTSTACDKEMAKKLASKEVLKSLKGANFS

**>Gh_D05G3343_NBI-AD1_v1.1_ID=Gh_D05G3343_NBI-AD1_v1.1_*Gossypium hirsutum* |mRNA| length=4188bp_DCL2a**

ATGGAGCCAGTTGACATGGAGAAGGACATTTCTCAACTACACCCTGCTGATCCTCTCCCTTTTGCCAGAAGCTATCAGCTTGAAGCATTAGAGAAAGCTATCAAGCAAAACACAATAACTTACTTGGAAACTGGATCTGGCAAGACAATGATCGCCATCATGCTTCTCCGCAGCTATGCCCATCTTATTCGCAAGCCCTCACCTTTTTTTGCTGTCTTCTTGGTTCCCAAAGTTGTCCTGGTTAAACAACAAGCTGATGCTGTGGAAATGCATACGGACTTGAATGTTGGAAAGTATTGGGGAGATATGCAGGTCGACTTTTGGGACGGGGAGAAGTGGAAGCAAGAAATAGATAAATATGAGGTGCTTGTGATGACACCTCAGATTTTACTTGATGGATTGAGGCATAGCTTCTTCAAGATAAACATGATAAAGGTTTTGATAATTGATGAATGCCACCATGCCCGAGGAAATCACCCTTATGCCTCTATTATGAGAGAATTCTATCATCGCCATTTAGAAGCTGGTGCATCTAATCTTCCTAGGATTTTTGGGATGACTGCTTCTCCTATAAATTCAAAAGGGGCAAACTCTGCTGATAGCTATTGGCAGAAGATCCATGAATTGGAGACGATTATGAACTCAAAGGTGTATACATGTGAAAGTGAATCAGTGCTTGCTCAGTTTGTTCCATTTTCTACTCCAAAGTTCAAGTTTTACCAACATATGGAAATTCCAAATGTTCTATATGCACACTTGGTAGAGGAATTGACTGTTTTGAAAGTAAAGCATGAATGTTCGTTGGATAATTTGGATCTTGAAGCTTCTGCAGCAGAATCTACAAGAAAGAAACTATCAAAGATACATTCAGCTTTAATACATTGTCTACATGAGCTTGGTGTTTGGTTGGCTTTAAAGGCTGCAGAGTGCTTTTCATGTTATGAAAGTGAGCATCTTATGTGGGGGAAATTGGATGTCTTTGGCGAGAAAATTATTAGGAGCTACAGTGTGGATGCTTTTCATGCAATTGAAACATGCATGCCATCTGGTCTGGATTGGACCATAGCTAATGATGTTAAAGGCAGTGTGGCTGCTGGGTTTCTTACCACAAAAGTTTTATGCCTTATTGAATCTCTATTTGAATACAGGGTGTTGAAGGACATAAGATGTATAATTTTTGTTGAGAGGGTTATAACAGCTGTTGTGCTTCAATCGCTATTTAGTGAATTACTTCCCAGGTACAGTAATTGGAAGACTAATTACATTGCGGGAAATAACTCTGGATTGCAGAATCAGACAAGGAAAAAACAAAACGAAATTGTAGAAGAATTCCGCAAAGGCATGGTTAACATAATCGTTGCAACTTCAATTCTTGAAGAAGGCTTGGATGTTCAATCATGCAACCTAATTATCAGATTTGATCCTTCACCAACAATTTGTAGTTTCATACAGTCTCGAGGACGTGCTAGAATGCAGAACTCAGATTATCTATTAATGCTGAAGAGTGGGGACTTTTCTACACATTCTCGACTGAAGAACTATCTTACTAGTGGAGATGTAATGAGAAAGGAATCTTTACGCCATGCATCCAATCCTTGTTCTCCTCTTAGTAAGGGCTTAGATGATGAAGAGTTTTACCAAGTTGCGAGTACAGGGGCATGTATGACTCTTAGTTCTAGTGTTGGTCTAATGTACTTCTATTGCTCACGTCTCCCTGCAGATGGGTATTTTAAACCTATTCCGAGGTGTGTTATTGACAAGCAAATGGGGCTTTGCACCCTCCTCCTACCCAAGAGTTGCCCTATACAAACTGTTTGTGTTCAGGGTAATATAAAAAACCTAAAGAAAATAGCTTGCTTTGAAGCATGCAAGAAACTCCATCAAATTGGTGCTTTAACAGACAATCTTGTTCCTGATATTGTTTTTGAAGAAAATGATGTGGAAGAATTTGAAAAGGAGCCCTATAATGATGATCAACCTATTTTCTTTCCACCTGAACTAGTGAATAAGGGTTCACTGGACTCCATGACAAAGTACTACTGCTATTTAATGGAGTTGAAGCAGAACTTTGATTATGAGGTTCCTGTTCATAACATCATGCTTCTTGTCAGGAATCAGTTTGACATGGATGAAAAAAGTGTGAATATTGAGTTAGAAGTTGACAGGGGCACATTAACAGTTAACATGAAATATATTGGACTAATACGTCTTAATTCTGACCAGGTTATCTTATGTAGAAGGTTTCAGCTTGCAGTTTTTCAAGTACTTATGGATCGTAAAGCTGAAAAGTTGGCAGAGGTGTTATGTGACCATACCTTGGGGAATAATTCTGAAATTGATTATCTACTCCTCCCATCAAATTATGTGGGCCAGAGTCCTCTGATTGATTGGTTGTCAGTTACTTCTGTCACATTTTCTTATGAAAAGGCCTGGAAGAATCATGTGAACTGCAATGCTGGTATGATACAGACCAAAAGCAGTCTGGTGTGCACTTGCATGGTCCAAAATTCGTTGGTCTTTACTCCTCATAATGGTCATGCTTATATTATCAGTGGTCTTTTAACTAATATAAATGCAAATTCACTTTTGAGGTTGAGTGATGGACGCTTAATGACTTACAAGGAGTATTATGAACAACGCCACAGTATCAATTTCTGTTATAGTGAAGTCTCTTTCCTTGCTGGGCGACATATTTTCCCTGTGCAAAATCACATTCAAAGGTGCAGAAAACAAAAAGAGAAAGGCATGTTTAATGCATTGGTAGAATTGCCTCCTGAGCTTTGTTGTGTAGTAATGTCTCCCATATCGGTTAGCACATTTTATTCATTCACATTTCTTCCGTCAATCATGCATCGACTCGAGTCTTTGCTCCTTGCTACCAGCTTGAAAAAGATGCATTTGGATCATTGCGTGCAAAATATTGCGATTCCAACCATGAAGGTTTTGGAGGCAATTACAACCAAAAAATGCCTAGAAAACTTTCATTTGGAATCACTAGAGACTCTTGGTGACTCTTTTTTGAAGTATGCTGTTTGTCAGCAGCTTTTCAAAAAATATCAAAATCATCATGAGGGCCTTCTTAGTATTAGGAAGGACAAAATTATTTCGAATACGGCTCTGTCCATGCTAGGATGTGACAAGAAACTTCCGGGGTTTATCCGTGATGAGCCTTTTGATACCAAAGATTGGATGATTCCTGGTTATAATTGTGGAAATTACTCATTAAATGAGGAAACACTGTGTAATGCAAAAAAAATATATGTTAGAGGGAGAAGAAAGCTAAAGTGTAAGAAGGTTGCTGATGTTGTTGAGGCACTTATTGGTGCATACCTTAGCACGGGAGGCGAAGCAGCTGGATTACTATTTTTGGATTGGATCGGTATAAGTATTAATTTCACAAATATACCATATGAAAGACATTTTAAAGTGCGGGCCGAGAAGTTTGTTAATGTCCAGCACTTTGAGTCCCTTCTACACTATTCATTCCAAGACCCTTCTTTGTTGGTGGAAGCATTAACCCATGGTTCTTATATGCTTGCTGAAATTCCAGGTTGTTATCAGCGGCTGGAATTTCTAGGGGACTCCGTTTTAGATTATCTTATTACTCTGCATTTATATAATAAATATCCTGGGATCACGCCAGGATTATTAACGGATCTTAGGTCAGCATCTGTTAACAACAATTGCTATGCATTATCAGCTGTAAAGGCTGGATTTCATAAGCACATTCTCCAAAGTTCACAGAAGCTTTACAAGGATATAAAAGAAACCGTCGAAAGTTTTCAGGAATTATCATTGGAATACACTTTCGGCTGGGAATCTGAAAAATCTTTCCCCCAGGTACTCGGTGACGTGGTGGAGTCACTTGCTGGAGCTATTTTTGTTGATTCAGGATACAAGAAGGAAATCGTATTCCAGAGTATAAGGCCACTGTTGGAGCCCATGATTACTCCAGAGACTATGACGGTTCACCCTGTCAAAGAGCTATATGAGCTCTGTCAAAAGGAGCATTATGAACTAAGAAAAGCCATTGTTTCACAAGAGGACGGCATTTCTTCTATTACGATAGAGGTTGAAGCAAATGGGAAGGTATTCAAGCACACATCCACAGCTTGTGATAAGAAGATGGCCAAGAAACTGGCTTCCAAAGAAGTTTTGAAGTCTCTAAAGGGAGCCAATTTTAGCTAG

**>Gh_D05G3343_DCL2a_DCL2a_protein sequence length= 1395aa**

MEPVDMEKDISQLHPADPLPFARSYQLEALEKAIKQNTITYLETGSGKTMIAIMLLRSYAHLIRKPSPFFAVFLVPKVVLVKQQADAVEMHTDLNVGKYWGDMQVDFWDGEKWKQEIDKYEVLVMTPQILLDGLRHSFFKINMIKVLIIDECHHARGNHPYASIMREFYHRHLEAGASNLPRIFGMTASPINSKGANSADSYWQKIHELETIMNSKVYTCESESVLAQFVPFSTPKFKFYQHMEIPNVLYAHLVEELTVLKVKHECSLDNLDLEASAAESTRKKLSKIHSALIHCLHELGVWLALKAAECFSCYESEHLMWGKLDVFGEKIIRSYSVDAFHAIETCMPSGLDWTIANDVKGSVAAGFLTTKVLCLIESLFEYRVLKDIRCIIFVERVITAVVLQSLFSELLPRYSNWKTNYIAGNNSGLQNQTRKKQNEIVEEFRKGMVNIIVATSILEEGLDVQSCNLIIRFDPSPTICSFIQSRGRARMQNSDYLLMLKSGDFSTHSRLKNYLTSGDVMRKESLRHASNPCSPLSKGLDDEEFYQVASTGACMTLSSSVGLMYFYCSRLPADGYFKPIPRCVIDKQMGLCTLLLPKSCPIQTVCVQGNIKNLKKIACFEACKKLHQIGALTDNLVPDIVFEENDVEEFEKEPYNDDQPIFFPPELVNKGSLDSMTKYYCYLMELKQNFDYEVPVHNIMLLVRNQFDMDEKSVNIELEVDRGTLTVNMKYIGLIRLNSDQVILCRRFQLAVFQVLMDRKAEKLAEVLCDHTLGNNSEIDYLLLPSNYVGQSPLIDWLSVTSVTFSYEKAWKNHVNCNAGMIQTKSSLVCTCMVQNSLVFTPHNGHAYIISGLLTNINANSLLRLSDGRLMTYKEYYEQRHSINFCYSEVSFLAGRHIFPVQNHIQRCRKQKEKGMFNALVELPPELCCVVMSPISVSTFYSFTFLPSIMHRLESLLLATSLKKMHLDHCVQNIAIPTMKVLEAITTKKCLENFHLESLETLGDSFLKYAVCQQLFKKYQNHHEGLLSIRKDKIISNTALSMLGCDKKLPGFIRDEPFDTKDWMIPGYNCGNYSLNEETLCNAKKIYVRGRRKLKCKKVADVVEALIGAYLSTGGEAAGLLFLDWIGISINFTNIPYERHFKVRAEKFVNVQHFESLLHYSFQDPSLLVEALTHGSYMLAEIPGCYQRLEFLGDSVLDYLITLHLYNKYPGITPGLLTDLRSASVNNNCYALSAVKAGFHKHILQSSQKLYKDIKETVESFQELSLEYTFGWESEKSFPQVLGDVVESLAGAIFVDSGYKKEIVFQSIRPLLEPMITPETMTVHPVKELYELCQKEHYELRKAIVSQEDGISSITIEVEANGKVFKHTSTACDKKMAKKLASKEVLKSLKGANFS

**>Gh_A01G1100_NBI-AD1_v1.1_ID=Gh_A01G1100_NBI-AD1_v1.1_ *Gossypium hirsutum*| mRNA|length=4245bp_DCL2b**

ATGGAGCCAGTTGGCATGGAGATGAACATTTCCCAACAACACTCTTCTGATCCTCTCCCATTTGCCAGAAGCTATCAGCTTGAAGCACTAGAGAAAGCTATCAAGCAAAACACAATAGCTTACTTGGAAACTGGCTCTGGCAAGACACTGATTGCCATCATGCTTCTTCGCAGATATGCCTACCTTGTTCGCAAGCCTTCACCTTTTAATGCTGTCTTCTTGGTTCCCCAAGTTGTCCTGGTTGAACAACAAGCTGATGCTGTGGAAATGCATACGGACTTGAATGTGGGCAAGTATTGGGGAGATATGGAGGTTGACTTTTGGGATGATGCAAATGGAAGCAGGAAATTGATAAATATGAGGCTTCTTTTGTTGACTGGTACTATAGTTTCAAGTCAAATAACTGTGTTTCTTTCTTCCTTCTTAGATATGGTGCTTGTGATGACACCTCAAATTTTACTTAATGGATTGAGGCATGGCTTCTTCAAGATAAACATGATTAAGGTTTTGATAATTGATGAATGCCATCATGCCCGAAGAAAGCACCCTTATGCTTCCATTATGACAGAATTCTATCATCGGCAATTAGAAGCTGGTGTATCTGATCTTCCTAGGATTTTCGGGATGACTGCTTCCCCTATAAACACAAAAGGGGCAAACTCAGCTGATGGCTATTGGCAGAAGATCCATGAACTAGAGACGATTATGAATTCAAAGGTGTATACATGTGTAACTGAATCAGTGCTTGCTCAGTTCGTTCCATTTTCGACTCCAAAGTTCAAGTTTTACCAAGACATGGAAATTCCATATGTTTTATATGCACGCATGGTACAGGAATTGAATGTTTTGAAAGTAAAGCATGAATGTTCGTTGGACAATTTGGATCTTGAAGCTTCTGCAGCAGAATCTACGAGAAGGAAAATATCAAAGATGCATTCAGCTTTAATACATTGTTTGGACGAGCTTGGTGTCTGGTTGGCTTTAAAGGCTGCAGAGTACTTGTCATGTTATGAAAGTGAGTTTCTTATGTGGGGCAAATTGGATGTCTTTGGCGAGAAAATTGTTAGGAGTTACACTGTGGATGCTTTCCATGCCATTAGAACATGCATACCATCTGATCCAGATTGGACCATCGTTAATGATGTTAAAGCCAGTGTGGATGCTGGGTTTCTTACCACAAAAGTTTTATGCCTTATTGAATCTCTTTCTGAATACAGGGTGTTGAAAGACATAAGGTGTATAATTTTTGTGGAGAGAATTATAACAGCAGTTGTGCTTCAATCACTATTCAGTGAATTGCTTCCGAAGTACGGTAACTGGAAGACTAAATACATTGCAGGAAATAACTCTGGATTGCAGAACCAGACTAGGAAAAAACAAAATGAAATTGTAGAAGAATTCCGAAAAGGCATTGTAAACGTAATTGTTGCAACTTCGATTCTTGAGGAAGGCTTGGATGTTCAAGCATGCAACTTAATTATCAGATTTGATCCTTCGCCGACAGTTTGTAGTTTTATACAGTCTCGAGGACGTGCTAGAATGCAGAATTCCGATTATCTATTAATGGTGAAGACCGGGGATTTTTCTACTCATTCTCAACTGAAGAACTATCTTACTAGTGGAGATGTAATGAGAAAGGAATCTCTGTGCCATGCATCTGATCCTTGTTCTCCTCTAAGCAATGACTTATGTGATGAAGAGTTCTACCATGTTGCAAGTACAGGGGCATTTATGACTCCTAGTTCAAGTGTTGGTTTGATATACTTCTATTGCTCACGCCTCCCTACAGACAGATATTTTAAACCTACTCTGAGGTGTATCATAGACAAGCAAAAGGGGATTTGCACCCTCCATCTACCCAAGAGTTCCCCTATACAAACTGTTTGTGTTAAGGGAAACTTCAAAAGCCTAAAGCAGAAGGCATGCTTTGAAGCATGCAAGCAACTCCATCAATTTGGTGCTTTGACAGATAATCTTGTGCCTGATATTGTTGTGGAAGAAAATGATGCTGAAGAAATTGGAAAGGAATCCTATAATGATGATCAACCGATTTTTTTTCCTCCTGAACTGGTGAATCAAGATTCACAGGAGTCCATGACAAAATACTACTGCTACTTAATGGAGTTGAAGCAGAACTTTGGTTATGAGATTCCTGTTCATAACATCATACTCCTTGTCAGGAGTCAGCTTGAAATGGAAGCAAAAAGTATTGAGTTAGAAGTTGATAGGGGCACTCTGACAGTTAACTTGAAATATATTGGGCTGATACGTCTTAGTTCTGACCAGGTTATCTTGTGTAGAAGGTTTCAGATTGCTCTTTTTCGAGTTCTTATGGATCATAAAGCAGAAAAGTTGACAGAGTTGTTATCTGACCTTACGTCAGGGAACAATTCTGAAATCGATTATCTTCTTCTCCCATCAAATTACATGGGCCAACCCGTGATTGATTGGTTGTCAATTTCTTCCATCTTATTTTCTTATGAGAAGGTCTGGAAGAATCATGTGAACTGCAATGCTGGCATGATACAGACCAAAAGTGGTCTGGTGTGCACTTGCATGATAAAAAATTCGTTGGTCTCTACTCCTCATAATGGTCACACATATATTATTGACGGTCTTTTAAATAATATAAATGCAAATTCACTTTTGACACTGAGTGATGGAGGAGTAATGACCTACAAGGAATATTATGAACAACAACATGGTATCCATTTATGTTTTAGTCGAGTCTCTTTCCTTGCTGGGCGACACATTTTCCCTGTGCAAAATCACATTCAAAGGTGCAAAAAACAGAAAGAGAAAGAATCCAGTAACGCATTTGTAGAATTGCCTCCTGAGCTTTGTGATGTAATAATGTCTCCCATATCGATTAGCACATTTTATTCTTTCACATTTCTTCCATCAATCATGCATCGACTCGAGTCTTTACTCCTTGCTACCAGCTTGAAAAAGATGCTTCTCGATCATTGTGTCCATAATGTTGCTATTCCAACCATGAAGGTTTTGGAGGCAATTACCACAAAAAAGTGCCTGGAAAGTTTTCATTTGGAATCACTGGAGACTCTTGGTGATTCTTTTTTGAAGTATGCTGTTTGTCAACAGCTATTCAAAAAACATCAGAATCATCATGAGGGCCTTCTTAGTATTAGGAAGGACAAAATGATTTCAAATACAACCCTTTGCATGCTGGGATGTGACAAGAAACTTCCGGGATTTATCCGTGATGAGCCTTTCGATCCCAAAGGTTGGATGGTTCCTGGTTATAATTGTGGAAGATATACATTAGATGAGAAGACATTGAATACAAGAAAAATATATGTTAGTGGGAGAAGGAAGCTGAAGAGTAAGAAGGTTGCTGATGTTGTTGAGGCTCTTATCGGTGCATATCTTAGCATGGGAGGTGAAGCTGCTGCATTATTATTTTTGAATTGGATTGGTATAACTATTGATTTTAGAAATATACCGTACGAAAGACATTTCGAAGTGCAGGCTGAAAAGTTTGTTAATGTCCAACATTTAGAGTCTCTTCTTAACTATTCATTTCAAGACCCTTCTTTTTTAGTGGAAGCACTAACACATGGTTCTTACATGCTTGCTGAAATTCCAGGATGTTATCAGCGGCTGGAATTTCTAGGAGACTCCGTTTTAGATTATCTCATCACTCGGCATTTATATAATAATTATCCTGGGATCTCACCAGGATTATTAACGGATCTGAGGTCAGCATCTGTTAACAACAATTGCTATGCATTATCAGCTGTTAAGGCTGGATTGCATAAGCACATTCTCCAAAGTTCACAGAAGCTTTACAAGCATATAAAAGAAACTGTTGAAAGTTTTCAGGAATTATCGTTGGATTGCACTTTCGGTTGGGAATCTGAAAAATCTTTCCCCAAGGTACTGGGTGATGTGATGGAGTCACTTGCTGGGGCTATATTTGTTGATTCAGGATACAACAAGGAAACTGTATTCCGGAGTATCAGGCCACTGTTGGAGCCCCTGATTACTCTAGAGACTATGACGGTCCACCCCGTGCAGGAGTTGAATGAACTTTGTCAAAAGAAGCATTATGAACAAAGAAAACCCATTGTTTCACATGACAATGGGGTTTCTTCTGTTACCATAGAGGTTGAAGCAAATGGCGAGGTATTAAAGCACACATCAACAGCTTCTGATAGGAAGATGGCCAAGAAACTGGCTTCCAAAGAAATTTTGAAGTCCCTAAAGGGAGCCAATTTTTGCTAG

**>Gh_A01G1100_DCL2b _protein sequence length= 1414aa**

MEPVGMEMNISQQHSSDPLPFARSYQLEALEKAIKQNTIAYLETGSGKTLIAIMLLRRYAYLVRKPSPFNAVFLVPQVVLVEQQADAVEMHTDLNVGKYWGDMEVDFWDDANGSRKLINMRLLLLTGTIVSSQITVFLSSFLDMVLVMTPQILLNGLRHGFFKINMIKVLIIDECHHARRKHPYASIMTEFYHRQLEAGVSDLPRIFGMTASPINTKGANSADGYWQKIHELETIMNSKVYTCVTESVLAQFVPFSTPKFKFYQDMEIPYVLYARMVQELNVLKVKHECSLDNLDLEASAAESTRRKISKMHSALIHCLDELGVWLALKAAEYLSCYESEFLMWGKLDVFGEKIVRSYTVDAFHAIRTCIPSDPDWTIVNDVKASVDAGFLTTKVLCLIESLSEYRVLKDIRCIIFVERIITAVVLQSLFSELLPKYGNWKTKYIAGNNSGLQNQTRKKQNEIVEEFRKGIVNVIVATSILEEGLDVQACNLIIRFDPSPTVCSFIQSRGRARMQNSDYLLMVKTGDFSTHSQLKNYLTSGDVMRKESLCHASDPCSPLSNDLCDEEFYHVASTGAFMTPSSSVGLIYFYCSRLPTDRYFKPTLRCIIDKQKGICTLHLPKSSPIQTVCVKGNFKSLKQKACFEACKQLHQFGALTDNLVPDIVVEENDAEEIGKESYNDDQPIFFPPELVNQDSQESMTKYYCYLMELKQNFGYEIPVHNIILLVRSQLEMEAKSIELEVDRGTLTVNLKYIGLIRLSSDQVILCRRFQIALFRVLMDHKAEKLTELLSDLTSGNNSEIDYLLLPSNYMGQPVIDWLSISSILFSYEKVWKNHVNCNAGMIQTKSGLVCTCMIKNSLVSTPHNGHTYIIDGLLNNINANSLLTLSDGGVMTYKEYYEQQHGIHLCFSRVSFLAGRHIFPVQNHIQRCKKQKEKESSNAFVELPPELCDVIMSPISISTFYSFTFLPSIMHRLESLLLATSLKKMLLDHCVHNVAIPTMKVLEAITTKKCLESFHLESLETLGDSFLKYAVCQQLFKKHQNHHEGLLSIRKDKMISNTTLCMLGCDKKLPGFIRDEPFDPKGWMVPGYNCGRYTLDEKTLNTRKIYVSGRRKLKSKKVADVVEALIGAYLSMGGEAAALLFLNWIGITIDFRNIPYERHFEVQAEKFVNVQHLESLLNYSFQDPSFLVEALTHGSYMLAEIPGCYQRLEFLGDSVLDYLITRHLYNNYPGISPGLLTDLRSASVNNNCYALSAVKAGLHKHILQSSQKLYKHIKETVESFQELSLDCTFGWESEKSFPKVLGDVMESLAGAIFVDSGYNKETVFRSIRPLLEPLITLETMTVHPVQELNELCQKKHYEQRKPIVSHDNGVSSVTIEVEANGEVLKHTSTASDRKMAKKLASKEILKSLKGANFC

**>Gh_D01G1133_NBI-AD1_v1.1_ID=Gh_D01G1133_NBI-AD1_v1.1_*Gossypium hirsutum*| mRNA|length=4179bp_DCL2b**

ATGGAGCCAGTTGGCATGGAGATGAACATTTCCCAACAACACTCTTCTGATCCTCTCCCATTTGCCAGAAGCTATCAGCTTGAAGCACTAGAGAAAGCTATCAAGCAAAACACAATAGCTTACTTGGAAACTGGCTCTGGCAAGACACTGATTGCCATCATGCTTCTCCGTAGATATGCCTACCTTATTCGGAAGCCTTCACCTTTTAATGCTGTCTTCTTGGTTCCCCAAGTTGTCCTGGTTGAACAACAAGCTGATGCTGTGGAAATGCATACGGACTTGAATGTGGGAAAGTATTGGGGAGATATGGAGGTTGACTTTTGGGATGATGCGAAATGGAAGCAAGAAATTGATAAATATGAGGTGCTTGTGATGACACCTCAAATTTTACTCAATGGATTGAGGCATGGCTTCTTCAAGATAAACATGATTAAGGTTTTGATAATTGATGAATGCCATCATGCCCGAGGAAAGCACCCTTATGCTTCCATTATGACAGAATTCTATCATCGGCAATTAGAAGCTGGTGTATCTGATCTTCCTAGGATTTTCGGGATGACTGCTTCCCCTATAAACACAAAAGGGGCAAACTCGGCTGATAGCTATTGGCAGAAGATCCATGAACTAGAGACGATTATGAATTCAAAGGTGTATACATGTGTAAGCGAATCAGTGCTTGCTCAGTTCGTTCCATTTTCGACTCCAAAGTTCAAGTTTTACCAAGACATGGAAATTCCATATGTTTTATATGCACGCTTGGTACAGGAATTGAATGTTTTGAAAGTAAAGCATGAATGTTCGTTGGACAATTTGGATCTTGAAGCTTCTGCAGCAGAATCTACGAGACGGAAAATATCGAAGATACATTCAGCTTTAATACATTGTTTAGATGAGCTTGGTGTCTGGTTGGCTTTAAAGGCTGCAGAGTACTTGTCATGTTATGAAAGTGAGTTTCTTATGTGGGGGAAATTGGATGTCTTTGGCGACAAAATTGTTAGGAGTTACAGTGTGGATGCTTTCCATGCCATTGGAACATGCATACCATCTGATCCAGATTGGACCATCGTTAATGATGTTAAAGCCAGTGTGGATGATGGGTTTCTTACCACAAAAGTTTTATGCCTTATTGAATCTCTTTCTGAATACAGGGTGTTGAGAGACATAAGGTGTATAATTTTTGTGGAGAGAATTATAACAGCAGTTGTGCTTCAATCACTATTCAGTGAATTGCTTCCGAAGTACGGTAACTGGAAGACTAAATACATTGCAGGAAATAACTCTGGATTGCAGAACCAGACTAGGAAAAAACAAAATGAAATTGTAGAAGAATTCCGAAAAGGCATGGTAAACATAATTGTTGCAACTTCGATTCTTGAGGAAGGCTTGGATGTTCAAGCATGCAACTTAATTATCAGATTTGATCCTTCACCAACAGTTTGTAGTTTTATACAGTCTCGAGGACGTGCTAGAATGCAGAATTCGGATTATCTATTAATGGTGAAGAGTGGGGATTTTTCTACACATTCTCGACTGAAGAACTATCTTACTAGTGGAGATGTAATGAGAAAGGAATCTTTGTGCCATGCATCTGATCCTTGTTCTCCTCTAAGCAATGACTTATGTGATGAAGAGTTCTACCATGTTGCAAGTACAGGGGCATTTATGACTCTTAGTTCTAGTGTTGGTTTGATATACTTCTATTGCTCACGCCTCCCTGCAGACGGGTATTTTAAACCTACTCTGAGGTGTATCATAGACAAGCAAATGGGGGTTTGCACCCTCCATCTACCCAAGAGTTCCCCTATACGAACTGTTTGTGTTAAGGGTAACTTTAAAAGCCTAAAGCAGAAGGCATGCTTTGAAGCATGCAAGCAACTCCATCAAATTGGTGCTTTGACAGATAATCTTGTGCCTGATATTGTTGTGGAAGAAAATGATGTTGAAGAAATTGGAAAGGAATCCTATAATGATGATCAACCGATTTTTTTTCCACCTGAACTGGTGAATCAAGATTCACAGGAGTCCATGACAAAATACTACTGCTACTTAATGGAGTTGAAGCAGAACTTTGGTTATGAGTTTCCTGTTCAAAACATCATACTCCTTGTCAGGAGTCAGCTTGAAATGGAAGCAAAAAGTGTGGGTATTGAGTTAGAAGTTGATAGGGGCACTCTGACAGTTAACTTGAAATATATTGGACTGATACGTCTTAGTTCTGACCAGGTTATCTTGTGTAGAAGGTTTCAGATTGCTCTTTTTCGAGTTCTTATGGATCATAAAGCAGAAAAGTTGACAGAGTTATCTGACCTTACGTCAGGGAACAATTCTGAAATTGATTATCTTCTTCTCCCATCAAATTACATGGGCCAAAATCCCGTGATTGATTGGTTGTCAATTTCTTCCATCTTATTTTCTTATGAGAAGGTCTGGAAGAATCATGTGAACTGCAATGCTGGCATGATACAGACCAAAAGTGGTCTGGTGTGCACTTGCATGATAAAAAATTCGTTGGTCTCTACTCATAATGGTCGCACATATATTATTGACGGTCTTTTAAATAATATAAATGCAAATTCACTTTTGACACTGAGTGATGGAGGAGTAATGACCTACAAGGAATATTATGAACAACAACATGGTATCCATTTATGTTTTAGTCGAGTCTCTTTCCTTGCTGGGCGACACATTTTCCCCGTGCAAAATCACATTCAAAGGTGCAAAAAACAGAAAGAGAAAGAATCCAGTAACGCATTTGTAGAATTGCCTCCTGAGCTTTGTGATGTAATAATGTCTCCTATATCGATTAGCACATTTTATTCTTTCACATTTCTTCCATCAATCATGCATCGACTCGAATCTTTACTCCTTGCTACCATCTTGAAAAAGATGCTTCTCGATCATTGTGTGCATAATGTTGCTATTCCAACCATGAAGGTTTTGGAGGCAATTACCACAAAAAAGTGCCTGGAAAGTTTTCATTTGGAATCACTGGAGACTCTTGGTGATTCTTTTTTGAAGTATGCTGTTTGTCAACAGCTATTCAAAAAACATCAGAATCATCATGAGGGCCTTCTTAGTATTAGGAAGGACAAAATGATTTCAAATACAACCCTTTGCATGCTGGGATGTGACAAGAAACTTCCGGGATTTATCTGTGATGAGCCTTTCGATCCCAAAGGTTGGATGGTTCCTGGTTATAACTGTGGAAGACATACATTAAATGAGGAGAAATTGAATACAAGAAAAATATACGTTAGTGGGAGAAGGAAGCTGAAGAGTAAGAAGGTTGCTGATGTTGTTGAGGCTCTTATTGGTGCATACCTTAGCACGGGAGGTGAAGTAGCTGCATTATTATTTATGAATTGGATTGGTATAACTATTGATTTTAGAAATATACCATACGAAAGACATTTCGAAGTGCAGGCTGAAAAGATTGTTAATGTCCAACATTTAGAGTCTCTTCTTAACTATTCATTCCAGGACCCTTCTTTGTTAGTGGAAGCACTAACACATGGTTCTTACATGCTTGCTGAAATTCCAGGATGTTATCAGCGGCTGGAATTTCTAGGGGACTCCGTTTTAGATTATCTTATCACTCGGCATTTATATAATAAATATCCTGGGATCTCACCAGGATTATTAACGGATCTGAGGTCAGCATCTGTTAACAACAATTGCTATGCATTATCAGCTGTCAAGGCTGGATTGCATAAGCACATTCTCCAAAGTTCACAGAAGCTTTACAAGCATATAAAAGAAACTGTTGAAAGTTTTCAGGAATTATCATTGGATTGCACTTTCGGTTGGGAATCTGAAAAATCTTTCCCCAAGGTACTGGGTGATGTGATGGAGTCACTTGCTGGGGCTATATTTGTTGATTCAGGATACAACAAGGAAACTGTATTCCGGAGTATCAGGCCACTGTTGGAGCCCCTGATTACTCTAGAGACTATGACGGTTCACCCCGTGAAGGAGTTGAATGAACTCTGTCAAAAGAAGCATTATGAACGAAGAAAACCCATTGTTTCACATGGCAATGGGGTTTCTTCTGTTACCATAGAGGTTGAAGCAAATGGCGAGGTATTAAAGCACACATCAACAGCTTGTGATAAGAAGATGGCCAAGAAACTGGCTTCCAAAGAAATTTTGAAGTCCCTAAAGCTAGCCAATTTTTGCTAG

**>Gh_D01G1133_DCL2b_protein sequence length= 1392aa**

MEPVGMEMNISQQHSSDPLPFARSYQLEALEKAIKQNTIAYLETGSGKTLIAIMLLRRYAYLIRKPSPFNAVFLVPQVVLVEQQADAVEMHTDLNVGKYWGDMEVDFWDDAKWKQEIDKYEVLVMTPQILLNGLRHGFFKINMIKVLIIDECHHARGKHPYASIMTEFYHRQLEAGVSDLPRIFGMTASPINTKGANSADSYWQKIHELETIMNSKVYTCVSESVLAQFVPFSTPKFKFYQDMEIPYVLYARLVQELNVLKVKHECSLDNLDLEASAAESTRRKISKIHSALIHCLDELGVWLALKAAEYLSCYESEFLMWGKLDVFGDKIVRSYSVDAFHAIGTCIPSDPDWTIVNDVKASVDDGFLTTKVLCLIESLSEYRVLRDIRCIIFVERIITAVVLQSLFSELLPKYGNWKTKYIAGNNSGLQNQTRKKQNEIVEEFRKGMVNIIVATSILEEGLDVQACNLIIRFDPSPTVCSFIQSRGRARMQNSDYLLMVKSGDFSTHSRLKNYLTSGDVMRKESLCHASDPCSPLSNDLCDEEFYHVASTGAFMTLSSSVGLIYFYCSRLPADGYFKPTLRCIIDKQMGVCTLHLPKSSPIRTVCVKGNFKSLKQKACFEACKQLHQIGALTDNLVPDIVVEENDVEEIGKESYNDDQPIFFPPELVNQDSQESMTKYYCYLMELKQNFGYEFPVQNIILLVRSQLEMEAKSVGIELEVDRGTLTVNLKYIGLIRLSSDQVILCRRFQIALFRVLMDHKAEKLTELSDLTSGNNSEIDYLLLPSNYMGQNPVIDWLSISSILFSYEKVWKNHVNCNAGMIQTKSGLVCTCMIKNSLVSTHNGRTYIIDGLLNNINANSLLTLSDGGVMTYKEYYEQQHGIHLCFSRVSFLAGRHIFPVQNHIQRCKKQKEKESSNAFVELPPELCDVIMSPISISTFYSFTFLPSIMHRLESLLLATILKKMLLDHCVHNVAIPTMKVLEAITTKKCLESFHLESLETLGDSFLKYAVCQQLFKKHQNHHEGLLSIRKDKMISNTTLCMLGCDKKLPGFICDEPFDPKGWMVPGYNCGRHTLNEEKLNTRKIYVSGRRKLKSKKVADVVEALIGAYLSTGGEVAALLFMNWIGITIDFRNIPYERHFEVQAEKIVNVQHLESLLNYSFQDPSLLVEALTHGSYMLAEIPGCYQRLEFLGDSVLDYLITRHLYNKYPGISPGLLTDLRSASVNNNCYALSAVKAGLHKHILQSSQKLYKHIKETVESFQELSLDCTFGWESEKSFPKVLGDVMESLAGAIFVDSGYNKETVFRSIRPLLEPLITLETMTVHPVKELNELCQKKHYERRKPIVSHGNGVSSVTIEVEANGEVLKHTSTACDKKMAKKLASKEILKSLKLANFC

**>A06:22197055-22207732_NBI-AD1_v1.1 _NBI-AD1_v1.1_*Gossypium hirsutum*| Possible mRNA|length=3630bp_DCL3a**

ATGGTTTCTTATATGACTGGATCTAATACTTCTGTTGATTCTCTTGCTCCTAAGATTCAAAAGGAAACTCTTGAATCTTTTAGATCTGGAAAGGTTAATCTTCTTTTTACTACTGATGTTGTTGAAGAAGGAATTCATGTTCCTAATTGTTGTTATGTTATTAGATTTGATCTTCCTAAGACTGTTAGATCTTATGTTCAATCTAGAGGAAGAGCTAGACAAAATAATTCTGAATTTATTATGATGCTTGAAAGAGGAAATATGAAGCAAAGAAATCAACTTTATGATATTATTAGATCTGAATATTCTATGACTAATCTTGCTATTAAGAGAGATCCTGATTCTGATCCTTGTCTTCTTAAGGATCATACTTTTGAAGAAACTAATGTTTTTATTGTTGATGCTACTGGAGCTTCTGTTACTGCTGATTCTGCTGTTTCTCTTATTCATAAGTATTGTGGAAAGCTTCCTGGAGATAAGTATTATACTCCTAAGCCTAATTTTCAATTTACTTCTTCTGAAGGACTTTATAAGTGTAAGCTTACTCTTCCTGTTAATGCTGCTGTTCAAACTATTGTTGGACCTCCTTCTAGAAATTCTCATCTTGCTAAGCAACTTGTTTGTCTTGAAGCTTGTAAGCAACTTCATCAAATGGGAGCTCTTGATGATCATCTTACTCCTTCTATTGAAGAACCTTCTGAAAATGCTTGTGTTTCTAAGGGAAAGGATTCTGGAGCTGGAGCTGGAACTACTAAGAGAAAGGAACTTCATGGAACTACTTGTATTCAAGCTCTTTGTGGATCTTGGGGAGAAAAGTCTGATGATGCTGTTTTTTTTGCTTATAAGTTTGATTTTAAGTGTAATATTATTACTGTTGTTTATTCTGGATTTGTTCTTCTTATTGAATCTAAGCTTGCTGATGATGTTGGAAATACTGAAATGGATCTTTTTCTTATTGGAAAGATGGTTAAGGCTTCTGTTTCTTCTTGTGGACAAGTTCATCTTAATGCTGAACAAATGATGAAGGCTAAGAGATTTCAAGAATTTTTTTTTAATGGACTTTTTGGAAAGCTTTTTGTTGGATCTAAGTCTTCTGGAGCTCCTAGAGAATTTCTTCTTAGAGATAAGTCTTCTTCTCTTTGGTCTCCTTCTAGAATGTATCTTCTTCTTCCTCTTGAAGATAATTCTACTGATGAACTTAGAATTCATTGGCCTGGAATTACTGCTTGTACTCTTGCTGCTGAATTTCTTAATAAGAATTCTCTTCTTGGAACTGAACAATCTGATGATGGAGGATCTAATCCTTCTCTTAATTCTACTGGATCTCCTGTTACTGATTGTAAGGAAACTAATATTATTAGATTTGCTAATTCTTCTGTTGATGCTAATTCTCTTAGAAATACTGTTGTTCTTGCTATTCATACTGGAAGAATTTATTGTATTATTGAAGCTGTTTCTGATAAGACTGCTGAATCTTCTTTTGCTGAAACTGTTGATACTGTTTCTTCTGAATTTGCTAATTTTTATGAATATTTTTATAAGAAGTATAATATTGTTCTTAAGCATCCTGGACAACCTCTTATGCTTCTTAAGCAATCTCATAATCCTCATAATCTTCTTGTTAATTTTAATGATGAAGGAGTTTCTGCTAAGGCTTCTCAAGCTGGAGTTGTTAATGAAAAGCCTAGATTTCATGTTCATATGCCTCCTGAACTTCTTCTTGTTCTTGATGTTCCTGTTTCTGTTCTTAAGTCTCTTTATCTTCTTCCTTCTCTTATGCATAGACTTGAATCTCTTATGCTTGCTAATCAACTTAGAGAAGAAATTAATTTTTGTTCTTCTAATATTGATATTCCTTCTTCTATGATTCTTGAAGCTCTTACTACTCTTAGATGTTGTGAATCTTTTTCTATGGAAAGACTTGAACTTCTTGGAGATTCTGTTCTTAAGTATGCTGTTTCTTGTCATCTTTTTCTTAGATATCCTAATAAGCATGAAGGACAACTTTCTGCTAGAAGATCTCTTGCTGTTTGTAATTCTACTCTTCATAAGCTTGGAACTGATCATAAGATTCAAGGATATATTAGAGATTCTGCTTTTGATCCTAGAAGATGGGTTGCTCCTGGACAAAGAGTTCTTAGACCTGTTCCTTGTAAGTGTGGAGTTGATTCTCTTGAAGTTCCTCTTGATAAGAAGTTTCAAACTGAAGATCCTAAGGTTAAGGTTGGAAAGTCTTGTGATAGAGGACATAGATGGTTTTGTTCTAAGACTATTTCTGATTGTGTTGAAGCTCTTATTGCTGCTTATTATCTTTCTGGAGGACTTGTTGCTGCTCTTCATGTTATGAAGTGGCTTGGAATTGATGCTGAAGTTGATCCTTCTGTTGTTGCTGAAGTTATTAATCAAGCTTCTCTTAGAACTTATGTTCCTAATTATAAGATTCATATGATTGAATCTAAGGTTGGATATAATTTTTCTGTTAAGTTTTTTCTTCAAGAAGCTCTTACTCATGAATCTGTTCATGAATCTTATTGTTATCAAAGACTTGAATTTCTTGGAGATTCTGTTCTTGATCTTCTTATTACTCAATATCTTTATAATCATCATACTGATATTGATCCTGGAGAACTTACTGATCTTAGATCTGCTTCTGTTAATAATGAAAATTTTGGACAAGTTGCTGTTAGACATGATCTTCATAAGCATCTTCAACATTGTTCTACTCTTCTTTCTAATCAAATTTCTGAATATGTTCAATCTTTTACTGAATCTGATAATACTACTAGACTTGATCCTTCTATTAAGGGACCTAAGGCTCTTGGAGATCTTGTTGAATCTATTGTTGGAGCTATTCTTATTGATACTAATCTTAATCTTGGAAAGGTTTGGAGAATTGTTGAACCTCTTCTTTCTCCTATTGTTACTCCTGATAAGCTTGAACTTCCTCCTTTTAGAGAACTTAATGAACTTTGTGATTCTCTTGGATATTTTATTAAGGAAAAGTGTATTAATAAGGGAGAAGTTGTTCATGCTGAACTTCTTCTTCAACTTGATCATGATCTTCTTGTTGGAGAAGGATTTGATAGATCTAGAAAGGTTGCTAAGGGAAAGGCTGCTTCTTGTCTTCTTAAGGATCTTGAAAATAGAGGAATTTCTAGAAAGAAGAGAAAGCATGATTATGTTGATTCTTCTCAAACTATGGATGATGATTCTCTTGAACCTACTATTCCTAAGATGCAAAGAAGAGCTGAAATTCAACTTCTTGATGAATCTAAGAAGGCTTGTTCTGCTACTCCTGCTACTCCTGTTATTGTTACTGTTAAGACTAAGAAGGGAGGACCTAGAACTACTCTTTTTGAACTTTGTAAGAAGCTTCTTTGGCCTATGCCTTCTATTAAGGCTACTGAACATAAGTCTTCTGCTCCTATGGAAATTGGAGAAGGACCTGAAAGAAAGAAGGGATTTATTTCTTTTGTTTCTAAGATTATTCTTAATGTTCCTGGATATGATATTATTGAATGTACTGGAGATGCTAAGGCTGATAAGAAGTCTTCTTCTGATTCTGCTGCTCTTTTTATGCTTTATGAACTTGAACAAAGAGGAAAGCTTATTATTGAAGAAACTCTTTGA

**>A06:22197055-22207732_GhDCL3a_ protein sequence length= 1209aa**

MVSYMTGSNTSVDSLAPKIQKETLESFRSGKVNLLFTTDVVEEGIHVPNCCYVIRFDLPKTVRSYVQSRGRARQNNSEFIMMLERGNMKQRNQLYDIIRSEYSMTNLAIKRDPDSDPCLLKDHTFEETNVFIVDATGASVTADSAVSLIHKYCGKLPGDKYYTPKPNFQFTSSEGLYKCKLTLPVNAAVQTIVGPPSRNSHLAKQLVCLEACKQLHQMGALDDHLTPSIEEPSENACVSKGKDSGAGAGTTKRKELHGTTCIQALCGSWGEKSDDAVFFAYKFDFKCNIITVVYSGFVLLIESKLADDVGNTEMDLFLIGKMVKASVSSCGQVHLNAEQMMKAKRFQEFFFNGLFGKLFVGSKSSGAPREFLLRDKSSSLWSPSRMYLLLPLEDNSTDELRIHWPGITACTLAAEFLNKNSLLGTEQSDDGGSNPSLNSTGSPVTDCKETNIIRFANSSVDANSLRNTVVLAIHTGRIYCIIEAVSDKTAESSFAETVDTVSSEFANFYEYFYKKYNIVLKHPGQPLMLLKQSHNPHNLLVNFNDEGVSAKASQAGVVNEKPRFHVHMPPELLLVLDVPVSVLKSLYLLPSLMHRLESLMLANQLREEINFCSSNIDIPSSMILEALTTLRCCESFSMERLELLGDSVLKYAVSCHLFLRYPNKHEGQLSARRSLAVCNSTLHKLGTDHKIQGYIRDSAFDPRRWVAPGQRVLRPVPCKCGVDSLEVPLDKKFQTEDPKVKVGKSCDRGHRWFCSKTISDCVEALIAAYYLSGGLVAALHVMKWLGIDAEVDPSVVAEVINQASLRTYVPNYKIHMIESKVGYNFSVKFFLQEALTHESVHESYCYQRLEFLGDSVLDLLITQYLYNHHTDIDPGELTDLRSASVNNENFGQVAVRHDLHKHLQHCSTLLSNQISEYVQSFTESDNTTRLDPSIKGPKALGDLVESIVGAILIDTNLNLGKVWRIVEPLLSPIVTPDKLELPPFRELNELCDSLGYFIKEKCINKGEVVHAELLLQLDHDLLVGEGFDRSRKVAKGKAASCLLKDLENRGISRKKRKHDYVDSSQTMDDDSLEPTIPKMQRRAEIQLLDESKKACSATPATPVIVTVKTKKGGPRTTLFELCKKLLWPMPSIKATEHKSSAPMEIGEGPERKKGFISFVSKIILNVPGYDIIECTGDAKADKKSSSDSAALFMLYELEQRGKLIIEETL

**>Gh_D06G0845_NBI-AD1_v1.1_ID=Gh_D06G0845_NBI-AD1_v1.1_ *Gossypium hirsutum*| mRNA|length=4839bp_DCL3a**

ATGTCTTTGCTGAAGTCCCATTCAACACCCCAATTTTCTCCGGTTATCTTAACCAAACTAATCGAGGAACAGGGTGAGCCTCTAAAACGAAGTTTCAGTGAGGCGAAGTCAGACCCACCTGATTGGATGGTTCTAGACGACAATGAAACTGCTAAGGAAGAAGACCCTTCTTCATCTTCAAAACCTAAGGACTTTAACCCCAGAGGGTATCAATTGCAGGTATATGAGGTGGCAAAGAGAAGAAACATAATAGCATTGTTGGATACAGGTGTAGGGAAGACAATGATAGCTGTGATGCTCATAAAAGATTTTGGTCAAGCTATCGAGTCTACTGAAAGTAAAAAATTGATTATTTTCTTGGCTCCCACTGTTCATCTAGTCAATCAGCAATTTGAATATGTAAAAGATCATACTAGTTTGGAAGTTGAACAGTATTATGGAGCTAAGGGGGTTGATGAATGGACCTTGGATTGTTGGGAGAAAGAAACAAAGGAGCATGATGTTCTGGTTATGACACCCCAAATTCTGTTGGATGCCTTAAGGAAGGCATTCTTGAGTCTGGAGATGGTGTCCTTAATGATAATAGACGAGTGCCACCGTGCTACTGGCAACCATCCATATGCCAAAATAATGAAGGAATTTTATCACAAATCTAATAACAAGCCAAAGATTTTTGGAATGACAGCATCACCTGTAGTTAGCAAAGGTGTGTTGTCCAGCAATAATTGCGATGGTCAAATGTCAGAACTTGAATGTGTTTTGGATTCCTTGATATACACTATTGAAGACAGGACAGAGATGGAAGCGTGTGTTCCTTCTGCTAAAGAAAGTTGTAGATTTTTTGATCCAGCACAATTTTCTAGCTTGGATTTGAAAGCAAAGGTAGAAGCCTCTTGGTTAAAGACAGATGGGTCATTGTCAAATTTGCAAAGCTCACTGCAAACTTCTTATAAAGACATGGATGATAAACTTAAGAATCTACGGAAGCGATTATCAAATGACCATGCTAAGGTTCTGCATTGCCTTGATAATCTTGGTCTCATATGTGCTTATGAGGCTGTTAACATTTGTCTAGAGAACATCCTTGACACCACAGAGGAATCTAAAGCATATAGAGAAAGTGTGTTGCAGTATAAAAATTTCCTTGAGGAAGTCCAATGTAGAATTGGGGAGTCTCTTCCCCTCGGTGATAAAAATTTTCTGAATTCTGGATTTGACTATTTGAAGGCAGTAGATCTTGGCTATATTTCTCCAAAACTGCATGAACTACTTCAACTGTTCCAATCATTTGGAGAAACTAGACAAGTATTGTGCCTGATTTTTGTTGAAAGAATTATTACTGCTAAAGTAATTGAAAGATTTGCAAAGAAAGTAAGCTGGTTATCACATTTCATGGTTTCATATATGACTGGAAGTAATACATCTGTTGATTCCCTGGCACCAAAAATGCAAAAGGAAACCTTGGAGTCATTTCGATCTGGGAAGGTGAATCTATTGTTTACCACTGATGTGGTTGAGGAGGGAATTCATGTACCAAATTGCTGTTATGTGATACGTTTCGACCTGCCAAAGACAGTCCGGAGTTATGTACAATCTCGAGGAAGAGCTAGGCAGAACAATTCTGAATTCATTATGATGCTTGAGAGGGGAAATGTGAAACAAAGAAATCAACTTTATGATATAATCAGGAGTGAGTATTCAATGACAAATTCGGCAATTAAAAGAGATCCCGATTCTGATCCATGCCTTCTTAAAGATCATACATTTGAAGAAACAAATGTTTTTATTGTGGATGCTACTGGAGCTTCAGTTACTGCAGATTCTGCTGTTAGCCTCATCCATAAATATTGTGGGAAGCTCCCCGGTGATAAGTATTACACACCAAAGCCAAATTTCCAGTTCACGTCTTCTGAAGGATTATATAAGTGTAAATTAACATTACCTGTTAATGCAGCTGTTCAAACAATAGTTGGTCCACCATCTAGGAATTCTCATTTAGCAAAGCAGCTTGTATGCTTAGAAGCATGTAAGCAGCTTCATCAAATGGGTGCCTTGGATGATCATCTCACTCCATCCATTGAAGAGCCTTCAGAAAATGCTTGTATTTCTAAAGGAAAAGATTCAGGTGCAGGTGCAGGTGCAGGTGCAGGAACTACAAAACGGAAGGAGCTTCATGGAACAACTTGCATACAGGCATTATGTGGAAGCTGGGGAGAAAAATCTGATGATGCTGTTTTCTTTGCCTATAAATTTGACTTCAAATGCAATATCATTACTGTGGTTTATTCTGGATTTGTTCTTCTAATTGAATCAAAGCTCGCGGATGATGTGGGGAATACTGAAATGGATCTTTTCTTGATTGGTAAGATGGTTAAGGCTAGCGTTTCTTCCTGTGGGCAAGTGCGTTTGAATGCAGAACAGATGATGAAAGCGAAGCGCTTTCAGGAATTTTTCTTTAATGGCTTGTTTGGGAAGTTATTTGTTGGATCAAAATCATCTGGAGCACCAAGAGAATTTCTACTTCGGGATAAAACAAGCTCCTTGTGGAGTCCATCCCACATGTATTTGCTTTTGCCCCTCGAGGATAATTCAACTGATGAATTGAGAATACACTGGCCAGGGATAACAGCTTGCACATTAGCTGTGGAATTTTTAAAAAAAAATTCTTTGTTGGGCACTGAGCAATCTGATGATGATGGAAGCAATCCATCATTGAACAGTACTGGTTCACCTGTGACATACTGCAAAGAAACCAATATAATCCGCTTTGCTAACAGTTCAGTTGATGCTAATAGTCTTAGAAATATGGTAGTATTGGCTATTCACACTGGAAGAATCTACTGCATCATTGAAGCTGTGAGTGATAAAACTGCTGAAAGTTCTTTCGCTGAAACTGTTGATACGGTCTCATCAGAGTTTGCTACCTTCTATGAATACTTCTACAAAAAGTACAATATTGTGCTGAAACATCCAGGACAGCCTTTGATGCTGTTAAAGCAGAGCCATAACCCGCACAACTTGCTTGTGAATTTTAATGATGAAGGTGTATCAGCTAAGGCATCACAAGCTGGCGTGGTTAATGAAAAGCCTCGGTTTCATGTCCACATGCCCCCTGAGCTTTTACTTGTCCTTGATGTCCCAGTGAGTGTTCTAAAATCATTGTACTTACTGCCATCATTGATGCATCGGCTGGAGTCCTTAATGTTGGCCAACCAACTCAGAGAAGAGATAAACTTTTGTTCTAGCAACATTGATATTCCAAGCTCAATGATCCTGGAAGCATTAACAACACTTAGATGCTGTGAAAGTTTTTCAATGGAAAGGCTGGAATTGCTTGGGGATTCAGTTCTGAAGTATGCTGTTAGCTGCCACCTCTTTCTTAGATATCCCAACAAACATGAGGGACAATTATCTGCCAGGCGTTCGTTGGCAGTTTGTAATTCAACCCTGCATAAGTTGGGAACTGACCACAAAATACAGGGATACATTAGGGACAGTGCTTTTGATCCCCGTCGTTGGGTAGCTCCAGGACAACGAGTGCTTCGCCCTGTTCCTTGCAAGTGTGGTGTGGACTCTCTAGAAGTTCCTTTAGATAAAAAATTTCAGACAGAAGACCCTAAAGTTAAGGTTGGTAAATCCTGTGATAGAGGCCACCGATGGTTGTGTTCAAAAACCATATCAGATTGTGTTGAAGCTCTCATAGGAGCATACTACTTAAGTGGTGGACTTGTTGCTGCCCTTCATGTGATGAAGTGGCTCGGGATTGATGCCGAACTTGATCCCTTGGTGGTAGCTGAAGTCATTAATCAGGCATCCTTAAGAACTTATGTCCCCAATTACGAAATCCACATGATAGAGTCAAAAGTTGGCTACAATTTTTCTGTCAAGTTTTTTTTGCAGGAGGCATTGACACATGAATCTTTGCACGAGTCCTACTGTTACCAGAGGCTTGAATTTCTTGGTGATTCTGTGTTGGACTTGCTGATCACCCAGTATCTCTACAACCATCACACTAATATAGATCCGGGTGAGTTGACTGACCTGCGCTCCGCTTCGGTTAATAATGAAAATTTTGCTCAAGTTGCCGTGCGCCATGACCTTCATAAGCATCTTCAACATTGTTCAACTTTACTATCAAACCAAATAAGTGAATATGTGCAGTCCTTCACTGAATCTGATAACACCACCAGATTAGATCCCAGCATAAAAGGTCCCAAGGCTCTTGGAGACCTGGTTGAAAGTATTGTTGGGGCAATTCTGATTGATACAAACCTGAATCTTGACAAAGTCTGGAGAATTGTTGAACCGTTGTTGTCTCCAATTGTAACCCCAGATAAGCTTGAGCTGCCTCCATTCCGGGAACTGAATGAATTATGCGACTCTCTTGGATATTTTATTAAGGAAAAATGTAAAAATAAGGGGGAAGTGGTGCACGCCGAGCTTCTATTGCAGCTAGATCATGATTTGTTGGTAGGAGAGGGGTTTGATAGAAGTAGAAAAGTAGCAAAAGGAAAAGCCGCTTCTTGTCTGTTGAAGGACCTTGAGAACAGAGGAATCTCCAGGAAGAAAAGGAAACATGACTGTGTGGATTCAAGTCAAACCATGGAAGATGACTCGTTAGAACCAACAATCCCTAAGAGGCAAAGGAGAGCCGAAATTCAGTTGCTTGATGAATCAAAGAAAGCATGCAGTGCTACGCCTGCCACCCCAGGATACGACATTATTGAATGCACTGGAGATGCAAAAGCTGACAAAAAAAGTTCTTCAGACTCAGCAGCACTGTTTATGCTTTATGAGCTTGAACAGCGTGGGAAGCTCATCATCGAGGAAACCTTATAA

**>Gh_D06G0845_DCL3a_ protein sequence length= 1612aa**

MSLLKSHSTPQFSPVILTKLIEEQGEPLKRSFSEAKSDPPDWMVLDDNETAKEEDPSSSSKPKDFNPRGYQLQVYEVAKRRNIIALLDTGVGKTMIAVMLIKDFGQAIESTESKKLIIFLAPTVHLVNQQFEYVKDHTSLEVEQYYGAKGVDEWTLDCWEKETKEHDVLVMTPQILLDALRKAFLSLEMVSLMIIDECHRATGNHPYAKIMKEFYHKSNNKPKIFGMTASPVVSKGVLSSNNCDGQMSELECVLDSLIYTIEDRTEMEACVPSAKESCRFFDPAQFSSLDLKAKVEASWLKTDGSLSNLQSSLQTSYKDMDDKLKNLRKRLSNDHAKVLHCLDNLGLICAYEAVNICLENILDTTEESKAYRESVLQYKNFLEEVQCRIGESLPLGDKNFLNSGFDYLKAVDLGYISPKLHELLQLFQSFGETRQVLCLIFVERIITAKVIERFAKKVSWLSHFMVSYMTGSNTSVDSLAPKMQKETLESFRSGKVNLLFTTDVVEEGIHVPNCCYVIRFDLPKTVRSYVQSRGRARQNNSEFIMMLERGNVKQRNQLYDIIRSEYSMTNSAIKRDPDSDPCLLKDHTFEETNVFIVDATGASVTADSAVSLIHKYCGKLPGDKYYTPKPNFQFTSSEGLYKCKLTLPVNAAVQTIVGPPSRNSHLAKQLVCLEACKQLHQMGALDDHLTPSIEEPSENACISKGKDSGAGAGAGAGTTKRKELHGTTCIQALCGSWGEKSDDAVFFAYKFDFKCNIITVVYSGFVLLIESKLADDVGNTEMDLFLIGKMVKASVSSCGQVRLNAEQMMKAKRFQEFFFNGLFGKLFVGSKSSGAPREFLLRDKTSSLWSPSHMYLLLPLEDNSTDELRIHWPGITACTLAVEFLKKNSLLGTEQSDDDGSNPSLNSTGSPVTYCKETNIIRFANSSVDANSLRNMVVLAIHTGRIYCIIEAVSDKTAESSFAETVDTVSSEFATFYEYFYKKYNIVLKHPGQPLMLLKQSHNPHNLLVNFNDEGVSAKASQAGVVNEKPRFHVHMPPELLLVLDVPVSVLKSLYLLPSLMHRLESLMLANQLREEINFCSSNIDIPSSMILEALTTLRCCESFSMERLELLGDSVLKYAVSCHLFLRYPNKHEGQLSARRSLAVCNSTLHKLGTDHKIQGYIRDSAFDPRRWVAPGQRVLRPVPCKCGVDSLEVPLDKKFQTEDPKVKVGKSCDRGHRWLCSKTISDCVEALIGAYYLSGGLVAALHVMKWLGIDAELDPLVVAEVINQASLRTYVPNYEIHMIESKVGYNFSVKFFLQEALTHESLHESYCYQRLEFLGDSVLDLLITQYLYNHHTNIDPGELTDLRSASVNNENFAQVAVRHDLHKHLQHCSTLLSNQISEYVQSFTESDNTTRLDPSIKGPKALGDLVESIVGAILIDTNLNLDKVWRIVEPLLSPIVTPDKLELPPFRELNELCDSLGYFIKEKCKNKGEVVHAELLLQLDHDLLVGEGFDRSRKVAKGKAASCLLKDLENRGISRKKRKHDCVDSSQTMEDDSLEPTIPKRQRRAEIQLLDESKKACSATPATPGYDIIECTGDAKADKKSSSDSAALFMLYELEQRGKLIIEETL

**>Gh_D13G2027_NBI-AD1_v1.1_ID=Gh_D13G2027_NBI-AD1_v1.1_*Gossypium hirsutum*| mRNA|length=4329bp_DCL3b**

ATGGAAGAAGAGCAGTGTTACCCTCTAAAAACAACCTTCAGTGAAACACACTTACACCCATCTCAGATTGTGGTTTCAATCAATGACGATCATGCTAAGAACCACCACCCTTCTTCATCTTTAAATCCTGATCACTCTATCCCTAGAGGGCATCAGCTGCAGGTGTATGAAGTTGCAAAGAGAAGAAATATCATAGCAGTGTTAGATACAAGTGGAGGGAAGACAATGATAGCTGTAATGCTTATCAAAGATTTTGTTCAACCTATCAACTCCATTGATAAAAAAAAGTTGATTATTTTCTTGGCCCCCACTGTTCATCTTGTTAATCAGCAATTTGAATATATAAAGTTCCATACAAGTTTGGATGTTGAACAATATTATGGAGATAAAGGGGTTAATGAATGGAACTCAGGGTATTGGGAGAAAGACATAAAGGAGCATGATGAATTCTATCACAAATCGAACAACAAACCGAAGATTTTCGGGATGACGGCATCGCCCGTAGTTAGCAAAGGTGCATTATCCAACAATGATTGTGAGGATCAAATTTCAGAACTTGAAAATGTTATGGATTGCATGATATATGCAATTGAAGACATGACAGAGATGGAAACATATGTTCCTACAGCAAAAGAAAGTTGTAGGTTTTTCGATCCAACACGATTTTGTAGTTCGGGTTTGAAAGCAATGATCGAAGCCTCGTGGTTAAAGATAGATGCTTCATTATCAACGTTGCAAGGCTCGATTCAAACTTCTTATACAGACATGGATGATAAGTTTAAGGCCCTCCATAAACGGTCATCCAATGACCATGCTAAGATTCTGCATTGTCTCGACAATCTTGGTCTCATATGTGCTTATGAGGCTGTTAAAGTCTGTCTTGAGAACATCCCTGATACAAAAGAGGAGTGTGAAGCATATAAAGAAGGTGTATTGCAGTGCAAAAGGTTCCTCAACGAAGTGCTACAGATAATCGGGGAATCCCTTCCCCTGGGTGATGAAAATTTTCTGAATTCCGGGTTTGACTACTTAAAGGCGGTAGATTCGGGCTATATATCTCCGAAACTGCATGAACTAATTGAATTTTTCTTATCACTCGGTAATACGTCAGTTGATTCCATGGCACTAAAGATTCAAAAGGAAACTTTGGAGTCATGTCAATCCGGGAAGGTGAATCTATTTTTTGCTACCGATGTGGTCGAGGAGGGAATTCATATACCAAACTGTTCATGTGTGATACGTTTTGACTTGCCGAAAACAGTGTTGAGTTATGTACAATCTCGAGGAAGAGCTAGGCAGAGTGGTTCCCAATTCATCATGATGCTAGAGAGGGGAAAAGAGAAACAAAGAGATCAACTATACGATCTAATTCGGAGTGAACATTCAATGACGAATATAGCTATGAATAGAGATCCTGATCTATCTCTTCCGGAAAACCGTACATTGGAAGAAACAAATGTTTCTATCATGGCTGCTACTGGAGCTTCCATTGAAGAAACTTCCAAAAATCGTTGTGCTTCTAAAGGAAAAGATCCCATTTTCGATTCAGGTGCAGGAACTACAAGATGGAAGGAGCTGCACGGAACAACTCAGATTCGTGCATTTTCCGGGAGCTGGGGAGAAAAAGCCGATGCTTCAGTTTTCTTTGCCTATAAAATTGACTTTTCATGCAATGTTGTTAGCGTTGTTTACTCTGGATTTGTACTTCTAATTGAATCTAAGCTTGCTGATGATGTGGGGAACATCGAAGTAGATCTTTACTTGATCGGAAAGATAGCTAAGGCTCGTGTTTCTTTTTATGGGAAAGTACATTTGGATGCTGTACAGATTACCGCAGCAAAGCGTTTTCAGGAAATTTTCTTTAATGGCTTGTTCGGGAGGCTAGATAGTTTGAGTAATGAACTGAGAATAAACTGGCCTGGGATAACAGCTTGTACATTAGCTGTAGAGTTTTTAACTGCTAACGGTAACAGAGGTAATCCGTCATTGAACCAAACGGATTCATCCGTGACAGAACACAAGGAGACCAATGTAATCCACTTTGCTAACAGATCTATAGATGTTAACAATATCAGTAACATCGTAGTATTGGCAATTCATACCGGAAGAATCTATTCCACCATTGAATTAGTTCATGATACATCCGCCAAAAGTTCTTTCAATGACATTGTTGATATGAACTCATCGAAGTTCGCGACCTTCTCCGAATACTGCAACAGAAAGTACTGCATCGTGCTGAAACATCCAGGACAACCTTTATTGCTGTTAAAGCAGAGTCATAACCCGCACAACTTGCTGGTTGATTTTAACGATGAAGATTTTTCAGCACAAGCATTACAAGCAAGCTCGGTTAATGAAAAGTCATGGAATAATATCCACATGCCACCCAAGCTTTTGCTCGTCCTTGATGTCCCGGTCTATGTTCTAAAAGCCTTTTACTTACTACCATCACTGATGCACCGGCTCGAGTCCTTAATGTTGGCTAGCCAACTTAGGGAAGAGATCGATTTTCGTTCAAGCAACTTCGGTATACCGAGCTCAATGATCCTGGAAGCGTTAACAACGCTTAGATGTTGTGAAAGCTTTTCGATGGAGAGGCTGGAATTTCTCGGTGATTCAGTTCTGAAGTATGCTGTGAGCTACCACCTCTTTCTTAGAGATCCCGAGAAACACGAAGGAGAATTATCAATGATACGTTCATTGGCACTGAGAAACTCGACGCTGCATAAGCTGGGAACTGACTGCAAATTACAGGGTTACATTAGAGACAGTGCTTTCGACCCCCGACGTTGGGTAGCTCCAGGACAACTACGTCTTCGGTCTTTTCCTTGTAACTGCAGGGTGGACTCTCGAGAAGTTCCTTTAGATAAAAAGTTTCAGACTGAAGACCTGGAAATTAAGGTCAGTATATCTTGTGATAGAGGCCACCGATGGATGTGTTCGAAAACCATATCGGATTGTGTTGAAGCTCTCATAGAAGCATACTATGTAAGTGGTGGACTCATTGCTGCTCTTCATATGATGAAATGGCTTGGGATCGATGCCGAACTTGATCCCTCTTTGGTAGCTGAAGCCATTTATCGTGCATCCCTACAATCTTATGTCCCTAATGATGAAATCCAGATTATAGAATCGAAAATTGGATACAAATTTTCTATGAAGTTTTTCTTACAGGAGGCCTTGACACATGCATCGGCAAACGAGTTTTACTGTTACCAGAGGCTTGAATTTCTTGGTGATTCCGTGTTGGACTTGCTGATAACCCGATATCTCTACTGCAATCATACCGATATAGATTCAGGAGAACTGACTGACCTCCGATCGGCTTCAGTTAGTAATGAAACTTTTGCTCAAGTTGCTGTCCGTAATGACCTTCATAAGCATCTTCGACATTGTTCAACTTTACTATTGGACCAAATAAGTGAATATGCACAGTCCTTCCCTGAATCTTGGGACACTACACGATCAGTACCCGGCATAAAAGGTCCAAAGGCTCTTGGAGACCTTATTGAAAGTATCGTCGCGGCAATCTTGATCGATACAAACCTGAACCTTGACGAAGTTTGGAGAATTGTTGAGTCATTGTTATCTCCGATCGTGACTCCCGATAAGCTCGAGGAAAAGTGCGTTAATACGGGAGAAATGGTGCATGCCAAGCTTTGGTTACAGCTGGATGATATTTTGTTGGTCGGAGAGGCATTCAATCGAAGCAAAAAGGTAGCAAAAGGAAAAGCAGCTTTATATCTATTGAAGGAACTCGAGGATAGAGGGATCTCCCGAAAGAGAACGAAACAGGATCATGCCGGTTCGAGTGGAACCACTGATGATGATTTGTTAGAGCCAAAAATGTGCAAGAAGCAGAGGATAGCTGAGATTCCTTCTCCGACTAACGACTCTTCAAGGACAGCATATGGAACTGAGATTCCTTCTCCGGCTAACGCTTCTTCAAGGACAGCATATGGTTTCAGCTCCGACGCCCTAGGGATGCAACATTCAGTTCAGAATTTGGTATTGAAACCGATAAACACAAATAAAGGACGACCGAGGGCCACTCTTTACGAGATTTGTCAGAAACAGTTATGGCCTAGGCCTACATTCGAAACAACAGAAGGAAGATCACGTTCTCCTATGGAAATCGGCAAAGGTGCAGACCGAAAAATCGGGTTTAACAGCTTTGTAACGAAGATCACCTTGAATGTACCGAGTTCAGGCATTATTGAATGCAGCGGAGATGTAAGAGCCGACAAAAAGAGCTCTTGTGACTCTGCAGCAATCCTTATGCTTTACAGGCTTAAAGAGCTAGGGAAGCTCATCATCCGCGAATCCTAG

**>Gh_D13G2027_DCL3b_ protein sequence length= 1442aa**

MEEEQCYPLKTTFSETHLHPSQIVVSINDDHAKNHHPSSSLNPDHSIPRGHQLQVYEVAKRRNIIAVLDTSGGKTMIAVMLIKDFVQPINSIDKKKLIIFLAPTVHLVNQQFEYIKFHTSLDVEQYYGDKGVNEWNSGYWEKDIKEHDEFYHKSNNKPKIFGMTASPVVSKGALSNNDCEDQISELENVMDCMIYAIEDMTEMETYVPTAKESCRFFDPTRFCSSGLKAMIEASWLKIDASLSTLQGSIQTSYTDMDDKFKALHKRSSNDHAKILHCLDNLGLICAYEAVKVCLENIPDTKEECEAYKEGVLQCKRFLNEVLQIIGESLPLGDENFLNSGFDYLKAVDSGYISPKLHELIEFFLSLGNTSVDSMALKIQKETLESCQSGKVNLFFATDVVEEGIHIPNCSCVIRFDLPKTVLSYVQSRGRARQSGSQFIMMLERGKEKQRDQLYDLIRSEHSMTNIAMNRDPDLSLPENRTLEETNVSIMAATGASIEETSKNRCASKGKDPIFDSGAGTTRWKELHGTTQIRAFSGSWGEKADASVFFAYKIDFSCNVVSVVYSGFVLLIESKLADDVGNIEVDLYLIGKIAKARVSFYGKVHLDAVQITAAKRFQEIFFNGLFGRLDSLSNELRINWPGITACTLAVEFLTANGNRGNPSLNQTDSSVTEHKETNVIHFANRSIDVNNISNIVVLAIHTGRIYSTIELVHDTSAKSSFNDIVDMNSSKFATFSEYCNRKYCIVLKHPGQPLLLLKQSHNPHNLLVDFNDEDFSAQALQASSVNEKSWNNIHMPPKLLLVLDVPVYVLKAFYLLPSLMHRLESLMLASQLREEIDFRSSNFGIPSSMILEALTTLRCCESFSMERLEFLGDSVLKYAVSYHLFLRDPEKHEGELSMIRSLALRNSTLHKLGTDCKLQGYIRDSAFDPRRWVAPGQLRLRSFPCNCRVDSREVPLDKKFQTEDLEIKVSISCDRGHRWMCSKTISDCVEALIEAYYVSGGLIAALHMMKWLGIDAELDPSLVAEAIYRASLQSYVPNDEIQIIESKIGYKFSMKFFLQEALTHASANEFYCYQRLEFLGDSVLDLLITRYLYCNHTDIDSGELTDLRSASVSNETFAQVAVRNDLHKHLRHCSTLLLDQISEYAQSFPESWDTTRSVPGIKGPKALGDLIESIVAAILIDTNLNLDEVWRIVESLLSPIVTPDKLEEKCVNTGEMVHAKLWLQLDDILLVGEAFNRSKKVAKGKAALYLLKELEDRGISRKRTKQDHAGSSGTTDDDLLEPKMCKKQRIAEIPSPTNDSSRTAYGTEIPSPANASSRTAYGFSSDALGMQHSVQNLVLKPINTNKGRPRATLYEICQKQLWPRPTFETTEGRSRSPMEIGKGADRKIGFNSFVTKITLNVPSSGIIECSGDVRADKKSSCDSAAILMLYRLKELGKLIIRES

**>Gh_A05G0400_NBI-AD1_v1.1_ID=Gh_A05G0400_NBI-AD1_v1.1_*Gossypium hirsutum*| mRNA|length=4926bp_DCL4**

ATGCCGGACGGCGAATTGTCCACCGACGGGACCGAGCTGTCCATGGCCTCTAAAGTTAAAGCCTTTTCTACTCCGTCGCCGATCGTTGAAATCAGCCGAAAAGATGAACCTATAATGGAAAAGAAAGAGAAGGATCCACGAAAAATTGCTAGAAAGTATCAACTGGAGTTATGCAAGAAAGCCATGGAAGAGAACATAATTGTTTATTTGGAGACTGGTTGTGGGAAGACCCACATTGCAGTTCTGCTTATTTATGAGCTTGGCCATTTGATACGGAAACCTCAGAATAGGGTATGCATTTTTCTTGCTCCCACAGTGGCTCTTGTTCAGCAGCAAGCCCGGGTTATAGAAGATTCTCTTGATTTCAAGGTTGGGACTTATTGTGGTAACTGCAGACACTTGAAGAACCATCATGACTGGGAAATAGAGATCAAAGAATACGAGGTTCTTGTTATGACTCCCCAAATACTACTTCGTAGCTTGTATCACTGCTTCATCAGGGTGGACTTAATCGCTCTTCTGATATTTGATGAGTGTCATCATGCTCAAATTAAAAGCAACCATCCTTATGCAGAAATCATGAAAGTTTTTTATGATAAAGCTACTGCATCAATGCTTCCTCGTATATTTGGAATGACAGCGTCTCCCGTTGTTGGTAAAGATGCTTCCAGTCAAGTAAATTTACCTAAAAGCATCAACAGCCTCGAAAACTTGCTTGATGCTAAGGTATATTCAGTTGGAGATAAGGAGGAATTGGAAAGCTTTGTAGCATCTCCTGTGGTTAGAGTATATGATTATGGTCCTGTCAATTTTGGTTCTTCTTGTTCCACCATGATCTATTGTAGTAAACTTGAGGAGATTAAGCGCCAGTGCATACCTCTTGTCAGGAAGAATGGTGATATTCAATCTGCCAGGAACACAAAAAAGCTGCTCAACAGAATGCATGATAATATAATCTTTTGCTTGGAAAATCTGGGCATTTGGGGAGCATTGCAAGCTTGCCGACTTCTTTTAACTGGTAATAACTCTGAGAGGAATGAATTGATAGAAGATGAAGGGATTTTAAGCGACGACTCTGTATGTGATCGATATCTTGTTCATGCTGCTGATGTTTTTGCTTCAGATTGTACGAGAGATGGACCTGCAAACGATTTATCTGATGTAGAGATTTTGAAGGAACCATTTTTCTCGAAAAAGCTGCTACGTCTTGTTGGAATTCTTTCCACCTTCAGGTTACAGCCAAATATGAAATGCATAATTTTTGTGAATAGGATTGTTACTGCAAGATCCTTGTCATACATACTGCAAAACCTTAAGTTCCTATTATTTTGGAAGTGCCATTTTCTTGTGGGAGTCCACTCTGGACTTAAAAGTATGTCAAGGAAGACAATGAAAAACATTCTTGAGAAGTTCAGAACAGGAGAGTTGAATCTTTTGATTGCAACTAAAGTCGGTGAAGAAGGACTTGACATTCAGACATGCTGCCTTGTGATACGTTTTGACCTTCCAGAAACTGTTGCGAGCTTTATCCAATCAAGAGGACGTGCAAGAATGCCTCTCTCTGAGTATGCATTTCTGGTGAACAGTGGGAACGAGAAGGAACTAGATTTGATTAAAAATTTTAAGAAAGATGAAGATCGTATGAATGTGGAAATTTCTTTCAGAACATCTACTGAGGTTTCTATTGGTCTTGAGGAGAGAATCTATACGGTTGATTCATCTGGTGCTTCCATCAGTTCAGGATATAGTATCTCTCTACTTCATCATTACTGCTCAAAACTGCCCCATGATGAGTACTTTTTCCCCAAGCCAAGCTTTTATTATTTTGAAGATTCAGGAGGAACAATCTGCAATATAATATTACCTTCTAATGCTCCAATAAATCAAATTGCCAGTACACCTCAATCTTCAGTTAATGCTGCCAAAAAGGATGCTTGTCTAAAAGCAATTGAAGAATTGCATAAATTGGGAGCCTTGACTGACCATCTCTTGCCACTGCAAAACAGTGTTCTTGAGGAGGAAACATTGCTGGTGTCTTCTGATTCTGGGAGCTCTGAAGCAGATGAGGATTCACGAGGTGAACTACATGAAATGCTTGTTCCTGCTGTGCTTAAAGAATCATGGACTAACTTAGAGAACTGTGTCCTCCTTTATGCTTACTATATAAAATTTATTCCGAATCCTGAGGACAGGAGCTATAAAGAGTTTGCTCTCTTTGTCAAGTCTCCTCTCCCTAAGGAGGCTGAGAGAATGGAGCTGGATCTTCACCTATCTCGCCGTAGGTCGGTGATGACAAAGCTTACCCCATCAGGAGTTGCAGAATTTAAGAGAGAAGAGATCATGCAGGCACAGCATTTTCAAGAAATGTTCTTCAAGGTCATCCTTGATAGATCAGAACTCCTTTCTGAATTTGTTACTTTAGGAAATGTCTTTCTTTCATCAAGCTCCTCAACATTCTATTTACTGCTTCCTGTTATTCTCTCCAATTGTGAGAATAAAGTGACAGTGGATTGGGGGATTGTGCAAAGATGTTTGTCGTCTCCACTTTTTAAGTCTCCTGTTGCAGCTGCAAAGATTGAAAATTTCCCTTCAGATGTTTGCTTGCACCTTGTAAATGGTTGTAGGAGTATAAGAGACATTGAGAATAGTTTGGTGTATGCCACACACAAGAGGGCCTTTTACTTTATTACTAGCATCGTTGGTGAAAAAAATGGTTACAGCCCATATAGAGATTCAGGCACTTTAAATCACTTGGAGCACTTAAACATGTTGGTTCTAGAAGCTCTTACTACAGAGAAGTGTCAAGAGCGTTTTTCTCTTGAAAGGCTTGAATCACTTGGTGATTCCTTCCTCAAGTTTGCTGTGGCCCGCCATCTTTTTCTTTTACATGATGCACTCGATGAAGGGGAATTGACTAGGAGAAGGTCAAATGTTGTAAATAATTCTAATTTATTCAAGCTGGCAACAAGGCGAAATTTACAGGTTTACATACGTGATCAACCTTTTGATCCTTATCAGTTCTTTCCCTTGGGCCATCCCTGCCCAGTAATTTGTACAAAAGAAACTAAAGGAACTGTTCATCCACAATCTAGTTGTCAAGTAGATCATGCAAAGAGTGAAGTCAGATGTAGTAGAAATCACCATTGGCTACAAAAGAAAACAATTTCTGATGTGGTTGAGGCTCTTGTAGGAGCATTTATAGTTGATAGGGGCTTCCAAGCGGCAACAGCATTTCTTAGATGGATAGGCATACGAGTGGACTTCCAAGGTTCTCAACTTAATAGTATTTGCGCTGCAAGCAAGGGATTTATGCCACTTTCCTCCCTATTGGATATTGGAGATCTTGAAAATTTGTTGGGTTATCAGTTTCTTCATAAGGGTCTCCTCCTTCAGGCGATTGTGCATCCTTCTTTTAATAGGCATGGTGGAGGCTGCTACCAGAGATTGGAGTTTCTTGGAGATGCTGTCTTGGATTATTTAATTACATCATATCTGTTTTCACTGTATCCAAAGTTGAAACCTGGTCAGTTGACTGATTTGAGATCAGTGTCAGTGAACAACAAGTCCTTTGCCAATGTTGCAGTGGACAGACGCTTACACAAATTTCTTATGTGTGACTCTTGTCACCTCAATGAGGCCATAGAAAAATATGTTGACTTCATCACATTATCACCAGACAGGGGATTATTTGAAGGGCCAAAATGTCCGAAGGCTCTTGGTGACTTGGTAGAGTCTTGTTTTGGTGCTATTCTCCTTGATACAGGGTTCAACTTGAACCGTGTTTGGAAGATAATGTTATCCATTCTGGATCCAATCAAGAGCCTTTCAAGTGTGCAGCTCAATCCTATCAGGGAAGTACAGGAACTTTCCCAGTGTTATAACTGGGATTTGCAGTTTCTAGTTGCAAAAGTTGGTAGAAATTTTTCAGTGGATGCAAAGGTAAATGCAGGAGATGTTCCTCTATGTGTTTGTTCCAGCAACATTAATAGAAAAGAAGCTATCAGAACTACTGCACATCAACTATATGTAAAGTTAAAGGCTCTAGGATATGCACCCAAATCAAAGTCCTTGGACGAAGTTTTGAAGGGAAGCCCCAAGAATGAAGCCAAGCTTATTGGATACGATGAAACACCCATTGATGTCACTGTTCCTGACATAATTGGATTTGAAAACATGAAATTGCAAGAATCTCTTGTGAATGATTTCAACCCCAAAACCCGTTCTAGTAAAAAGACAACTTCTAGCGGTGTATCTTGCATCTCGCCTGGTAGCAGACCTTTTGAAGTAAAAGCAGCAAGGGGTTCAGCCTCTGGTATTGAACCCAAAGGCACACCCCCCAATTGCTCCATTGTGGATCCTAGTTGTGGGATTGACTCGCCATCCAAAGGTGAATCACATAGTCGTACTGCTAGATCACAGTTATACGAAATCTGTGCTATCAATTGCTGGAAACCCCCTTTGTTTGAATGTTGCAAAGAGGAAGGACCAAGTCATTTGAGATCATTCACTTACAGGGTAATAGTTGAAATCGAAGAAGCTCCAGATATGATATTGGAGTGCTTCAGCAGTCCTCGGACCACAAAGAAGGCAGCAGCCGAGCATGCAGCGGAAGGCGCAGTTCACAGATGCCTCCTCGGAAGCGGAAGTGCCACCGCATCGGCCACTTCGAGTCGCCATTCCGTGCTCCTTAAATTCAAGGAGTTCATCAGAAACTTCGAGAAGGAGAAGAACGTGTTCCCTCACAGAGAAAGCCTCGTGGATAACCCTAAATTCCTAGTGGTCAATCTTGAAGACCTTCTCTCCTTCTATTCCGATCTCCCTTCCCTCCTTCGATCCTCTCCCTCCGATTATCTGCCTCTGGTCCGCATCATCTGCAACCGCAACACCGGAATCGCAATCGCGACAGACATTGCAATCGCAATTTAG

**>Gh_A05G0400_DCL4_ protein sequence length= 1648aa**

MPDGELSTDGTELSMASKVKAFSTPSPIVEISRKDEPIMEKKEKDPRKIARKYQLELCKKAMEENIIVYLETGCGKTHIAVLLIYELGHLIRKPQNRVCIFLAPTVALVQQQARVIEDSLDFKVGTYCGNCRHLKNHHDWEIEIKEYEVLVMTPQILLRSLYHCFIRVDLIALLIFDECHHAQIKSNHPYAEIMKVFYDKATASMLPRIFGMTASPVVGKDASSQVNLPKSINSLENLLDAKVYSVGDKEELESFVASPVVRVYDYGPVNFGSSCSTMIYCSKLEEIKRQCIPLVRKNGDIQSARNTKKLLNRMHDNIIFCLENLGIWGALQACRLLLTGNNSERNELIEDEGILSDDSVCDRYLVHAADVFASDCTRDGPANDLSDVEILKEPFFSKKLLRLVGILSTFRLQPNMKCIIFVNRIVTARSLSYILQNLKFLLFWKCHFLVGVHSGLKSMSRKTMKNILEKFRTGELNLLIATKVGEEGLDIQTCCLVIRFDLPETVASFIQSRGRARMPLSEYAFLVNSGNEKELDLIKNFKKDEDRMNVEISFRTSTEVSIGLEERIYTVDSSGASISSGYSISLLHHYCSKLPHDEYFFPKPSFYYFEDSGGTICNIILPSNAPINQIASTPQSSVNAAKKDACLKAIEELHKLGALTDHLLPLQNSVLEEETLLVSSDSGSSEADEDSRGELHEMLVPAVLKESWTNLENCVLLYAYYIKFIPNPEDRSYKEFALFVKSPLPKEAERMELDLHLSRRRSVMTKLTPSGVAEFKREEIMQAQHFQEMFFKVILDRSELLSEFVTLGNVFLSSSSSTFYLLLPVILSNCENKVTVDWGIVQRCLSSPLFKSPVAAAKIENFPSDVCLHLVNGCRSIRDIENSLVYATHKRAFYFITSIVGEKNGYSPYRDSGTLNHLEHLNMLVLEALTTEKCQERFSLERLESLGDSFLKFAVARHLFLLHDALDEGELTRRRSNVVNNSNLFKLATRRNLQVYIRDQPFDPYQFFPLGHPCPVICTKETKGTVHPQSSCQVDHAKSEVRCSRNHHWLQKKTISDVVEALVGAFIVDRGFQAATAFLRWIGIRVDFQGSQLNSICAASKGFMPLSSLLDIGDLENLLGYQFLHKGLLLQAIVHPSFNRHGGGCYQRLEFLGDAVLDYLITSYLFSLYPKLKPGQLTDLRSVSVNNKSFANVAVDRRLHKFLMCDSCHLNEAIEKYVDFITLSPDRGLFEGPKCPKALGDLVESCFGAILLDTGFNLNRVWKIMLSILDPIKSLSSVQLNPIREVQELSQCYNWDLQFLVAKVGRNFSVDAKVNAGDVPLCVCSSNINRKEAIRTTAHQLYVKLKALGYAPKSKSLDEVLKGSPKNEAKLIGYDETPIDVTVPDIIGFENMKLQESLVNDFNPKTRSSKKTTSSGVSCISPGSRPFEVKAARGSASGIEPKGTPPNCSIVDPSCGIDSPSKGESHSRTARSQLYEICAINCWKPPLFECCKEEGPSHLRSFTYRVIVEIEEAPDMILECFSSPRTTKKAAAEHAAEGAVHRCLLGSGSATASATSSRHSVLLKFKEFIRNFEKEKNVFPHRESLVDNPKFLVVNLEDLLSFYSDLPSLLRSSPSDYLPLVRIICNRNTGIAIATDIAIAI

**>Gh_D05G0516_NBI-AD1_v1.1_ID=Gh_D05G0516_NBI-AD1_v1.1_*Gossypium hirsutum*| mRNA|length=4968bp_DCL4**

ATGCCGGGCGGCGAATTGTCCACCGACGGGACCGAGCCGTTCATGGCCTCTAAAGTTAAAGCCTTTTCTACTCCGTCGCCGATCGTTGAAATCAGCCGAAAAGATGAACCTATAATGGAAAAGAAAGAGAAGGATCCACGAAAAATTGCTAGAAAGTATCAACTGGAGTTATGCAAGAAAGCCATGGAAGAGAACATAATTGTTTATTTGGAGACTGGTTGTGGGAAGACCCACATCGCAGTTCTGCTTATTTATGAGCTTGGCCATTTGATACGGAAACCTCAGAATAGGGTATGCATTTTTCTTGCTCCCACAGTGGCTCTTGTTCAGCAGCAAGCCCGGGTTATAGAAGATTCTCTTGATTTCAAGGTTGGGACTTATTGTGGTAACTGCAGACACTTGAAGAACCATCATGACTGGGAAATAGAGATCAAAGAATATGAGGTTCTTGTTATGACTCCCCAAATACTACTTCGTAGCTTGTATCACTGCTTCATCAGGGTGGACTTAATCGCTCTTCTGATATTTGATGAGTGTCATCATGCTCAAATTAAAAGCAACCATCCTTATGCAGAAATCATGAAAGTTTTTTATGATAAAGCTACTGCATCAATGCTTCCTCGTATATTTGGAATGACAGCATCTCCCGTTGTTGGTAAAGATGCTTCCAGTCAAGTAAATTTACCTAAAAGCATCAACAGCCTCGAAAACTTGCTTGATGCTAAGGTATATTCAGTTGGAGATAAGGAGGAATTGGAAAGCTTTGTAGCATCTCCTGTGGTTAGAGTATATGATTATGGTCCTGTCAATTTTGGTTCTTCTTGTTCCACCATTATCTATTGTAGTAAACTTGAGGAGATTAAGCGCCAGTGCATACCTTTTGTCAGGAAGAATGGTGATATTCAATCTGCCAGGAACACAAAAAAGCTGCTCAACAGAATGCATGATAATATAATATTTTGCTTAGAAAATCTGGGCATTTGGGGAGCATTGCAAGCTTGCCGACTTCTTTTAACTGGTAATAACTCTGAGAGGAATGAATTGATAGAAGATGAAGGGATTTTAAGCGACGACTCTGTATGTGATCGATATCTTGTTCATGCTGCTGATGTTTTTGCTTCAGATTGTACGAGAGATGGATCTGCAAACGATTTATCTGATGTAGAGATTTTGAAGGAGCCATTTTTCTCGAAAAAGCTGCTACGTCTTGTTGGAATTCTTTCCACCTTCAGGTTACAGCCAAATATGAAATGCATAATTTTTGTGAATAGGATTGTTACTGCAAGATCCTTGTCATACATACTGCAAAACCTTAAGTTCCTATTATTTTGGAAGTGCCATTTTCTTGTGGGAGTCCACTCTGGACTTAAAAGTATGTCAAGAAAGACAATGAAAAACATTCTTGAGAAGTTCAGAACAGGAGAGTTGAATCTTTTGATTGCAACTAAAGTCGGTGAAGAAGGACTTGACATTCAGACATGCTGCCTTGTGATACGTTTTGACCTTCCAGAAACTGTTGCGAGCTTTATCCAATCAAGAGGACGTGCAAGAATGCCTCTCTCTGAGTATGCATTTCTGGTGAACAGTGGGAACGAGAAGGAACTAGATTTGATTAAAAATTTTAAGAAAGATGAAGATCGTATGAATGTGGAAATTTCTTTCAGAACATCTACTGAGGTTTCTATCGGTCTTGAGGAGAGAATCTATATGGTTGATTCATCTGGTGCTTCCATCAGTTCAGGATATAGTATCTCTCTACTTCATCATTACTGCTCAAAACTGCCCCATGATGAGTACTTTTTCCCCAAGCCAAGCTTTTATTATTTTGAAGATTCAGGAGGAACAATCTGCAATATAATATTACCTTCGAATGCTCCAATAAATCAAATTGCCAGTACACCTCAATCTTCAGTTAATGCTGCCAAAAAAGATGCTTGTCTAAAAGCAATTGAAGAATTGCATAAATTGGGAGCCTTGACTGACCATCTCTTGCCACTGCAGAACAGTGTTCTTGAGGAGGAAACATTGCTGGTGTCTTCTGATTCTGGGAGCTCTGAAGCAGATGAGGATTCACGAGGTGAACTACATGAGATGCTTGTTCCTGCTGTGCTTAAAGAATCATGGACTAACTTAGAGAACTGTGTCCTCCTTTATGCTTACTATATAAAATTTAATCCGAATCCTAAGGACAGGAGCTATAAAGAGTTTGCTCTCTTTGTCAAGTCGCCTCTCCCTAAGGAGGCTGAGAGAATGGAGCTGGATCTTCACCTATCTCGCCGTAGGTCGGTGATGACAAAGCTTACCCCATCTGGAGTTGCAGAATTTAAGAGAGAAGAGATCATGCAGGCACAGCATTTTCAAGAAATGTTCTTCAAGGTCATCCTTGATAGATCAGAACTCCTTTCTGAATTTATTACTTTAGGAAATGTCTTTCTTTCATCAAGCTCCTCAACATTCTATTTATTGCTTCCTGTTATTCTCTCCAATTGTGAGAATAAAGTGACAGTGGATTGGGGGATTGTGCAAAGATGTTTGTCGTCTCCACTTTTTAAGCCTCCTGTTGCAGCTGCAAAGATTGAAAATTTCCCTTCAGATGTTTGCTTGCACCTTGTAAATGGTTGTAGGAGTATAAGAGACATTGAGAATAGTTTGGTGTATGCCACACACAAGAGGGCCTTTTACTTTATTACTAGCATTGTTGGTGAAAAAAATGGTTACAGCCCATATAGAGATTCAGGCACTTTAAATCACGTGGAGCACTTAAACATGTCTGGCATTCATCTTAAGTACCCTGAACAACCTCTTCTGCATGCAAAACCACTTTTTAAGTTGCACAATTTGCTCCACAACCGAAAGCCGGAGGATTCAGAAGCAAATGAACTGGAAGAATACTTCATTGATTTGCCTCCTGAGCTTTGTCAGCTGAAAATAATAGGCTTCTCCAAAGAGATAGGGAGTTCGCTTTCTTTGTTACCATCAATTATGCATCGCCTGGAGAACTTGCTTGTGGCTATTGAACTGAAGCATGTTTTTTCTGCTTCATTTGCCGAGGGAGCTGAAGTTACAGCCCTTAGGGTCCTAGAAGCACTTACTACAGAGAAGTGTCAAGAGCGTTTTTCTCTTGAAAGGCTTGAGACACTTGGTGATTCCTTCCTCAAGTTTGCTGTGGCCCGCCATCTTTTTCTTTTACATGATGCACTTGATGAAGGGGAATTGACTAGGAGAAGGATTAATGTTGTAAATAATTCTAATTTATTCAAGCTGGCAACAAGGCGAAATTTACAGGTTTACATACGTGATCAACCTTTTGATCCTTATCAGTTCTTTCCCTTGGGCCATCCCTGCCCAGTAATTTGTACAAAAGAAACTAAAGGAACTGTTCACCCACAATCTAGTTGTCAAGTAGATCATACAAAGAGTGAAGTCAGATGTAGTAGAAATCACCATTGGCTACATAAGGACACAATTTCTGATGTGGTTGAGGCTCTTGTAGGAGCATTTATAGTTGATAGGGGCTTCCAAGCGGCAACAGCATTTCTTAGATGGATAGGCATACGAGTGGACTTCCAAGGTTCTCAACTTAATAGTATTTGCGCTGCAAGCAAGAGATTTATGCCACTTTCCTCCCTATTGGATATTGGAGATCTTGAAAATTTGTTGGGTTACCAGTTTCTTCATAAGGGTCTCCTCCTTCAGGCGATTGTGCATCCTTCTTTTAATAGGCATGGGGGAGGCTGCTACCAGAGATTGGAGTTTCTTGGAGATGCTGTCTTAGATTATTTAATTACATCATATCTGTTTTCACTGTATCCAAAGTTGAAACCTGGTCAGTTGACTGATTTGAGATCAGTGTCAGTGAACAACAAGTCCTTTGCCAATGTTGCCGTGGACAGACGCTTACACAAATTTCTTATGTGTGACTCTTGTCACCTCAACGAGGCCATAGAAAAATATGTTGACTTCATCACATCATCATCACCAGACAGGGGATTGTTTGAAGGGCCAAAATATCCGAAGGCTCTTGGTGACTTGGTAGAGTCTTGTTTCGGTGCTATTCTCCTTGATACAGGGTTCAACTTGAACCGTGTTTGGAAGATAATGCTATCCATTCTGGATCCAATCAAGAGCCTTTCAAGTGTGCAGCTCAATCCTATCAGGGAAGTACAGGAGCTTTCCCAGCGTTATAACTGGGATTTGCAGTTTCTAGTTGCAAAAGTTGGTAGAAAATTTTCAGTGGATGCAAAGGTAAATGCAGGAGATGTACCTCTATGTGTTTCTTCCAGCAACATTAATAGAAAAGAAGCTATCAGAACTACTGCACATCAACTATATGTAAAGTTAAAGGCTCTAGGATATGCACCCAAATCAAAGTCCTTGGAAGAAGTTTTGAAGGGAAGCCCCAAGAATGAAGCCAAGCTGATTGGATACGATGAAACATCCATTGATGTCAGTGTTACTGACATAGTTGGATTTGAAAACATGAAATTGCAAGAATCTCTTGTGAATGATTTCAACCCCAAAACCCGTTCTAGTAAAAGGACAACTTCTAGCGGTGTATCTTGCATCTCACCTGGTAGCAGACCTCCACCTTCTTTTGAAGTAAAAGCAGCAAGGGGTTCAGCCTCTGGTATTGAAGCCAAAGGCAAATCCCCCAATTGCTCCATTGTGGATCCTAGTTGTGGGATTGACTCGCCATCCAAAGGTGAATCACATGGTCGTACTGCTAGATCACAGTTATACGAAATCTGTGCTATCAATTGCTGGAAACCCCCTTTGTTTGAATGTTGCAAAGAGGAAGGACCAAGTCATTTGAGATCATTCACTTACAGGGTAATAGTTGAAATCGAAGAAGCTCCAGATATGATATTGGAGTGCTTCAGCAGTCCTCGGACCACAAAGAAGGCAGCAGCCGAGCATGCAGCGGAAGGTGCACTCTGGTATTTAAAACATGGAGGATACTTACAATAG

**>Gh_D05G0516_DCL4_ protein sequence length= 1655aa**

MPGGELSTDGTEPFMASKVKAFSTPSPIVEISRKDEPIMEKKEKDPRKIARKYQLELCKKAMEENIIVYLETGCGKTHIAVLLIYELGHLIRKPQNRVCIFLAPTVALVQQQARVIEDSLDFKVGTYCGNCRHLKNHHDWEIEIKEYEVLVMTPQILLRSLYHCFIRVDLIALLIFDECHHAQIKSNHPYAEIMKVFYDKATASMLPRIFGMTASPVVGKDASSQVNLPKSINSLENLLDAKVYSVGDKEELESFVASPVVRVYDYGPVNFGSSCSTIIYCSKLEEIKRQCIPFVRKNGDIQSARNTKKLLNRMHDNIIFCLENLGIWGALQACRLLLTGNNSERNELIEDEGILSDDSVCDRYLVHAADVFASDCTRDGSANDLSDVEILKEPFFSKKLLRLVGILSTFRLQPNMKCIIFVNRIVTARSLSYILQNLKFLLFWKCHFLVGVHSGLKSMSRKTMKNILEKFRTGELNLLIATKVGEEGLDIQTCCLVIRFDLPETVASFIQSRGRARMPLSEYAFLVNSGNEKELDLIKNFKKDEDRMNVEISFRTSTEVSIGLEERIYMVDSSGASISSGYSISLLHHYCSKLPHDEYFFPKPSFYYFEDSGGTICNIILPSNAPINQIASTPQSSVNAAKKDACLKAIEELHKLGALTDHLLPLQNSVLEEETLLVSSDSGSSEADEDSRGELHEMLVPAVLKESWTNLENCVLLYAYYIKFNPNPKDRSYKEFALFVKSPLPKEAERMELDLHLSRRRSVMTKLTPSGVAEFKREEIMQAQHFQEMFFKVILDRSELLSEFITLGNVFLSSSSSTFYLLLPVILSNCENKVTVDWGIVQRCLSSPLFKPPVAAAKIENFPSDVCLHLVNGCRSIRDIENSLVYATHKRAFYFITSIVGEKNGYSPYRDSGTLNHVEHLNMSGIHLKYPEQPLLHAKPLFKLHNLLHNRKPEDSEANELEEYFIDLPPELCQLKIIGFSKEIGSSLSLLPSIMHRLENLLVAIELKHVFSASFAEGAEVTALRVLEALTTEKCQERFSLERLETLGDSFLKFAVARHLFLLHDALDEGELTRRRINVVNNSNLFKLATRRNLQVYIRDQPFDPYQFFPLGHPCPVICTKETKGTVHPQSSCQVDHTKSEVRCSRNHHWLHKDTISDVVEALVGAFIVDRGFQAATAFLRWIGIRVDFQGSQLNSICAASKRFMPLSSLLDIGDLENLLGYQFLHKGLLLQAIVHPSFNRHGGGCYQRLEFLGDAVLDYLITSYLFSLYPKLKPGQLTDLRSVSVNNKSFANVAVDRRLHKFLMCDSCHLNEAIEKYVDFITSSSPDRGLFEGPKYPKALGDLVESCFGAILLDTGFNLNRVWKIMLSILDPIKSLSSVQLNPIREVQELSQRYNWDLQFLVAKVGRKFSVDAKVNAGDVPLCVSSSNINRKEAIRTTAHQLYVKLKALGYAPKSKSLEEVLKGSPKNEAKLIGYDETSIDVSVTDIVGFENMKLQESLVNDFNPKTRSSKRTTSSGVSCISPGSRPPPSFEVKAARGSASGIEAKGKSPNCSIVDPSCGIDSPSKGESHGRTARSQLYEICAINCWKPPLFECCKEEGPSHLRSFTYRVIVEIEEAPDMILECFSSPRTTKKAAAEHAAEGALWYLKHGGYLQ

***Gossypium arboreum***

**>Cotton_A_14097_BGI-A2_v1.0_ID=Cotton_A_14097_BGI A2_v1.0_*Gossypium arboreum*| mRNA|length=6027bp_DCL1**

ATGGAGGAAGAAGGTAGGGTTTCTGGTGGCAATGGGTCATCTTACTGGCTGGATGCTTGTGAGGACATATCATGCGACTTGATTAGCGATTTTGTTGATTTCGATGCTCCTATAGTTCAAGACTCCGTTGACAATACTTCCAATCAAGATTTCTTTGGAGGAATTGATCACATTCTCGATAGCTTCAAGAACGGCGGGGGACTTCCTCCGGTGGGAAGTAACGGTGATAGCTCTGCCGTCAATGGAAATGGAATTCACGACCCCGTTGCTGGAGATGGATGGTCTCCCAATGAGCTCTCTGGGGTCTCCAAGGATTTACCTGACAACTCAGTTCCGCCATCTAATGGAGTTGAAAAGAAGATTGGAAGCAAAGGGCAGGAAAAAAGCTGCGATGATAGTAATTCCAGTTTATTTGATTATTCTAATAAGGATAATGGAGTTCATCGAGACGATAAAAGGTCATCCGAGTCGAGAGATAGGGGTTTAGATAGTGAAGAGAGGTGTAGAAAGAGGGCTCGTGCGAATGGCTGCAAGAGTGATAGGCAGTATTCTAGTAGAGGTCAATATTATCCCCGGGATAGGGAGAGATGTTCTTCTAGGAAAAGGGTTCGAGATTGGGATGAAATTGATCGGAGAGATAGAGAGCATGTTAGGAGAAGAGAACATTATTATGGTGGCAATAGGAGGGATGGGAGAGAGAGAGAACCAAGGGGTTATTGGGAGAGGGATCGGTCAGGGTCCAATGAGATGGTTTTTAGGTTGGGTACTTGGGAAGCAGATCGGCAAAGAGAAGGGAAGGTGGCTTATGACAAAACTCCCGAGTGCAATGGAAAGATGGAGAAGAAAGTTGAGCAGCCGAAGGAAAAACTCTTGGAGGAGCAAGCTCGTCAATATCAATTGGATGTTCTTGAGCAGGCAAAGAGGAAAAACACAATTGCATTTCTTGAAACCGGGGCAGGGAAGACCCTCATTGCTGTTCTCCTCATGAAAAGTATTTCTGATGATTTACAAAAGCACACCAGAAAAATGCTCTCTGTCTTTTTGGTTCCTAAAGTTCCACTAGTTTATCAGCAAGCTGAAGTTATTCGCGAGAGAACTGGTTTCCAAGTAGGTCATTATTGTGGGGAGATGGGTCAAGATTTTTGGGATGCTCGTAGGTGGCAGCGCGAGTTTGAATCAAAGCAGGTTTTGGTTATGACAGCTCAAATTCTGCTGAATATTTTGAGACACAGCATAATTAAAATGGAATCAATCAATCTCCTTATCCTGGACGAGTGTCATCATGCTGTGAAGAAACATCCGTATTCACTGGTTATGTCTGAATTCTATCATACAACACCAAAGGAGAAGAGACCTTCTGTTTTTGGAATGACCGCTTCTCCTGTTAACTTGAAGGGTGTTTCAAGTCAAGTTGATTGTGCTATAAAAATTCGTAATCTAGAAAGCAAACTGGATTCTGTAGTCTGTACCATCAAAGACCGCAAGGAACTCGAGAAACATGTGCCAATGCCTTCAGAAGTTGTGGTAGAGTATGACAAAGCAGCCAGTTTATGGTCCCTCCATGAACAAATAAAACAAATGGAAGCAACAGTTGAAGAAGCTGCACAGTCAAGCTCTAGAAGAAGTAAATGGCAGTTCATGGGAGCTAGAGATGCAGGAGCCAAGGAAGAGCTTCACCAAGTTTATGGTGTATCTGAAAGAACAGAAAGTGATGGAGCTGCTAATTTGATACAAAAGTTGAGGGCTGTTAATTATGCACTGGGTGAACTGGGCCAATGGTGTGCTTATAAGGTATCATATGTTGCACAATCTTTTCTGACGGCTTTGCAAAATGATGAGAGGGCAAACTACCAGCTTGATGTCAAGTTTCAAGAATCTTACCTAAACAAAGTTGTTTCTCTCTTACAATGCCAATTATCAGAGGGAGCTGTTACTGAAAAAGACATGAATAATGCAGAAGCAGAGAACTGTAATGCTCAAGATGGGACCAATACTGATGAGATTGAGGAAGGAGAGCTCCCTGACAGTCATGTTGTCTCTGGTGGAGAGCATGTGGATGTGATAATTGGAGCGGCTGTAGCAGATGGAAAAGTGACCCCGAAAGTACAGTCATTGATTAAAATACTTCTGAAGTATCAGCACACAGAGGATTTTCGAGCAATCATCTTTGTTGAGCGAGTTGTGGCTGCTTTAGTTCTTCCTAAGGTTTTTGCAGAGCTTCCGTCTCTGAGTTTCATCAGGTGTGCAAGTTTAATTGGGCACAACAATAGTCAAGAAATGCGGACTGGACAAATGCAGGATACAATTGCTAAATTCCGTGATGGTCGTGTGACATTGTTAGTTGCAACTAGTGTTGCTGAGGAAGGATTGGATATTCGGCAATGCAATGTTGTCATTCGATTTGATCTTGCAAAAACTGTTTTGGCATACATTCAGTCTAGAGGTCGTGCAAGGAAGCCTGGGTCAGATTACATCTTGATGGTTGAGAGGTACTTACAAGCTAACTTTCATGTTGTTTACAGAGGAAATTTATCGCATGCTACATTCTTAAGGAATGCTAGGAATAGTGAGGAGACCTTGCGGAAAGAAGCAATTGAGAGAACTGACCTTAGTCATTTGAAGGATACTTCGAGGTTGATTTCGGTGGATATGGTACCAGGTACGGTGTACCAGGTTGAATCAACTGGTGCCATTGTTAGCTTGAATTCTGCTGTTGGACTCGTCCATTTTTACTGCTCTCAGCTTCCTAGTGACAGATATTCAATACTTCGTCCCGAGTTTATTATGAAGAAGCATGAGAAGCCAGGGGGTCCAACTGAATATTCTTGCAAGCTTCAGCTCCCCTGTAATGCACCATTTGAAGAGCTCGAGGGTCCCATGTGCAGTTCTATGCGTCTTGCCCAGCAGGCATGCTATACTGCTGTATGTTTGGCTGCTTGCAAGAAGCTCCATGAGATGGGAGCATTTACTGATATGCTCTTGCCCGATAAAGGAAGCGGGGAAGAAGCAGAGAAGGTTGACCAGAATGATGAAGGAGACCCACTTCCTGGAACTGCTAGGCATAGAGAATTCTATCCAGAAGGTGTAGCAGATATACTCCAGGGAGAATGGATTTTATCTGGAAGAGATTGTGTTGGTGACTCCAAAATACATCGTCTGTACATGTATACTATCAAATGTGTAAATAATGGCTCTTCAAAAGATCCATTCTTAACTAAAGTCTCAGATTTTGCAGTACTTTTTGGCAAAGAGCTGGATGCAGAGGTGTTATCGATGTCGGTGGATCTATTTATCGTTCGAGCCATGATAACAAAGGCATCTCTTGTCTTCAGGGGATCAATAGATATAACTGAAAGTCAGATGGCATCCCTTAAAAATTTTCACGTAAGATTGATGAGCATTGTATTGGATGTGGATGTTGATCCTGCCACTACTCCTTGGGATCCTGCCAAGGCATATTTGTTTGTCCCTGTGGTTGGCGATAAGTTTGTAGATCCTATAAAAGAAGTTGATTGGGATTTGGTAGATAATATAATCACTACAAATGCGTGGAGCAATCCCCTTCAAAGAGCTAGGCCAGATGTTTTCCTTGGGACAAATGAGAGGACACTTGGTGGTGACAGAAGGGAGTATGGATTTGGGAAATTGCGTCATGGCCTGGCTTTTGGGCACAAACCTCATCCTACTTATGGTATCAGAGGAGCCGTAGCCCCATTTGATGTTGTTAAAGCTACCGGGGTGGTTCCTAGTCGTGATACGATTGAGGTACAAGGGGATTGGACCAAAGGCAATTTGATAATGGCTGATGGTGTTGCACGTGCAGAAGATCTTGTTGGAAGAATAATAACAGCCGCTCATTCGGGGAAGAGGTTTTATGTAGATACAATATGCTATGACATGTCAGCAGAGACCTCCTTTCCGAGGAAAGAGGGCTATCTTGGTCCTGTCGAGTACAGCTCATATGCCGATTACTATAAGCTGAAGTATGAATTTGATATCTCTTACTTAAATTCCTATTTTCTTCCCTACATCAAATATGCTTTCTGTTTCTTCCCGCACAACAAATGCATTAGGCTATTGAGTCCTCTATATATTGTTTCCAGGTATGGTGTTGAGTTGAGCTACAAGCAACAAGCTTTGATAAGAGGTCGTGGTGTTTCATACTGCAAGAATCTCTTATCTCCTCGATTTGAGCACTCAGAAGGTGAATCGGAGGAGGCACTTGACAAAACATACTACGTGTTTCTTCCTCCTGAGCTATGTTTTGTTCATCCACTTCCTGGATCACTTGTTAGAGGTGCCCAGAGGTTGCCCTCTATTATGAGGAGGGTTGAGAGCATGCTTCTTGCAATTCAACTTAAGCACATAATACAATTTCCGGTCCCTGCTTCAAAGATTTTGGAAGCTTTGACTGCTGCTTCGTGTCAGGAGACATTTTGCTATGAAAGGGCTGAGCTTCTTGGGGATGCTTACTTGAAATGGGTAGTCAGTCGTTTTCTGTTTCTTAAATATCCACAGAAACATGAAGGTCAACTGACCAGGATGAGACAACAAATGGTGAGTAACATGGTATTGTATCAATATGCATTAAATAAGGGACTTCAATCATACATCCAAGCAGATCGCTTTGCACCATCTAGATGGGCTGCTCCTGGGGTGTTGCCAGTCTTTGATGAGGATACAAAAGATGGTGACATGTCCTTATTTGATCAAGAACATGCAACTGCTGATGTTTTACCAGTAAAAGTACTTGGTGATGGGTTTGAAGATGAGGATATGGAAGATGGTGAGATTGAGAGTGACTCAAGTTCTTATAGAGTCCTCTCTAGCAAGACCTTAGCGGATGTGGTTGAAGCACTGATTGGAGTTTATTATGTTGAAGGTGGCAAGCATGCAGCTAACCACCTCATGAAATGGATTGGGATCCAGGTGGAGTCTGATCCTGATGATATGGACTCTATAGTGAAGCCATCGAATGTTCCAGAAAGCATACTCAGGAGTGTAAATTTTGAGGCCTTAGAAGGTGCATTGAACATCGAGTTTAAAAATAGGGCCTTGTTGGTAGAAGCTATTACTCATGCTTCACGCCCATCATCGGGAGTATCCTGCTACCAGCGTTTGGAGTTTGTTGGTGATGCAGTCTTGGATCATCTTATCACAAGACATTTGTTTTTTACATACACCAATTTGCCTCCAGGTCGCTTGACTGATTTGCGTGCTGCTGCCGTAAACAATGAAAACTTTGCACGTGTTGCTGTAAAGCATCAGTTGCACGTGCATCTTCGGCATGGATCAAGTGCCCTTGAGAAACAGATTCGGGACTTCGTGAAGGAAGTTCAGGATGAGTTATTAAAGCCGGGTTTCAACTCTTTTGGATTGGGAGATTGCAAAGCACCGAAAGTTCTTGGAGATATTGTTGAATCCATTGCTGGTGCCATTTTTCTTGACAGTGGACGTGACACTGCAGTTGTCTGGAGGGTTTTTCAACCTCTGTTGCATCCCATGGTGACTCCAGAGACATTGCCGATGCATCCTGTCCGGGAACTACAAGAACGATGCCAGCAACAAGCTGAAGGCTTGGAATACAAAGCCAGTCGTAGTGGCAATTTGGCCACTGTGGAGGTTTTCATTGATGGGGTCCAGGTTGGAGTTGCTCAGAATCCCCAAAAGAAGATGGCACAGAAACTAGCTGCAAGGAATGCACTTGCTGTTCTGAAGGAGAAAGAAACAGCTGAAGCTAAGGAGAACTGTGAGGAGAATGGGAAAAAGAAGAAGAATGGCAACCAGACATTCACCAGGCAAACATTGAATGATATCTGCCTGCGTCGAAACTGGCCTATGCCCTTCTATCGGTGTGTGAATGAAGGGGGCCCTGCCCATGCAAAGAGATTTACTTTTGCTGTCAAGGTTAACACCACTGACAGGGGGTGGACCGATGAATGCATAGGTGAGCCAATGCCTAGTGTTAAGAAGGCCAAGGACTCGGCCGCTGTGCTTCTCTTGGAACTTTTAAACAAATGGTATTCA

**>Cotton_A_14097_GaDCL1_ protein sequence length= 2009aa**

MEEEGRVSGGNGSSYWLDACEDISCDLISDFVDFDAPIVQDSVDNTSNQDFFGGIDHILDSFKNGGGLPPVGSNGDSSAVNGNGIHDPVAGDGWSPNELSGVSKDLPDNSVPPSNGVEKKIGSKGQEKSCDDSNSSLFDYSNKDNGVHRDDKRSSESRDRGLDSEERCRKRARANGCKSDRQYSSRGQYYPRDRERCSSRKRVRDWDEIDRRDREHVRRREHYYGGNRRDGREREPRGYWERDRSGSNEMVFRLGTWEADRQREGKVAYDKTPECNGKMEKKVEQPKEKLLEEQARQYQLDVLEQAKRKNTIAFLETGAGKTLIAVLLMKSISDDLQKHTRKMLSVFLVPKVPLVYQQAEVIRERTGFQVGHYCGEMGQDFWDARRWQREFESKQVLVMTAQILLNILRHSIIKMESINLLILDECHHAVKKHPYSLVMSEFYHTTPKEKRPSVFGMTASPVNLKGVSSQVDCAIKIRNLESKLDSVVCTIKDRKELEKHVPMPSEVVVEYDKAASLWSLHEQIKQMEATVEEAAQSSSRRSKWQFMGARDAGAKEELHQVYGVSERTESDGAANLIQKLRAVNYALGELGQWCAYKVSYVAQSFLTALQNDERANYQLDVKFQESYLNKVVSLLQCQLSEGAVTEKDMNNAEAENCNAQDGTNTDEIEEGELPDSHVVSGGEHVDVIIGAAVADGKVTPKVQSLIKILLKYQHTEDFRAIIFVERVVAALVLPKVFAELPSLSFIRCASLIGHNNSQEMRTGQMQDTIAKFRDGRVTLLVATSVAEEGLDIRQCNVVIRFDLAKTVLAYIQSRGRARKPGSDYILMVERYLQANFHVVYRGNLSHATFLRNARNSEETLRKEAIERTDLSHLKDTSRLISVDMVPGTVYQVESTGAIVSLNSAVGLVHFYCSQLPSDRYSILRPEFIMKKHEKPGGPTEYSCKLQLPCNAPFEELEGPMCSSMRLAQQACYTAVCLAACKKLHEMGAFTDMLLPDKGSGEEAEKVDQNDEGDPLPGTARHREFYPEGVADILQGEWILSGRDCVGDSKIHRLYMYTIKCVNNGSSKDPFLTKVSDFAVLFGKELDAEVLSMSVDLFIVRAMITKASLVFRGSIDITESQMASLKNFHVRLMSIVLDVDVDPATTPWDPAKAYLFVPVVGDKFVDPIKEVDWDLVDNIITTNAWSNPLQRARPDVFLGTNERTLGGDRREYGFGKLRHGLAFGHKPHPTYGIRGAVAPFDVVKATGVVPSRDTIEVQGDWTKGNLIMADGVARAEDLVGRIITAAHSGKRFYVDTICYDMSAETSFPRKEGYLGPVEYSSYADYYKLKYEFDISYLNSYFLPYIKYAFCFFPHNKCIRLLSPLYIVSRYGVELSYKQQALIRGRGVSYCKNLLSPRFEHSEGESEEALDKTYYVFLPPELCFVHPLPGSLVRGAQRLPSIMRRVESMLLAIQLKHIIQFPVPASKILEALTAASCQETFCYERAELLGDAYLKWVVSRFLFLKYPQKHEGQLTRMRQQMVSNMVLYQYALNKGLQSYIQADRFAPSRWAAPGVLPVFDEDTKDGDMSLFDQEHATADVLPVKVLGDGFEDEDMEDGEIESDSSSYRVLSSKTLADVVEALIGVYYVEGGKHAANHLMKWIGIQVESDPDDMDSIVKPSNVPESILRSVNFEALEGALNIEFKNRALLVEAITHASRPSSGVSCYQRLEFVGDAVLDHLITRHLFFTYTNLPPGRLTDLRAAAVNNENFARVAVKHQLHVHLRHGSSALEKQIRDFVKEVQDELLKPGFNSFGLGDCKAPKVLGDIVESIAGAIFLDSGRDTAVVWRVFQPLLHPMVTPETLPMHPVRELQERCQQQAEGLEYKASRSGNLATVEVFIDGVQVGVAQNPQKKMAQKLAARNALAVLKEKETAEAKENCEENGKKKKNGNQTFTRQTLNDICLRRNWPMPFYRCVNEGGPAHAKRFTFAVKVNTTDRGWTDECIGEPMPSVKKAKDSAAVLLLELLNKWYS

**>Cotton_A_34031_BGI-A2_v1.0_ID=Cotton_A_34031_BGI-A2_v1.0_ *Gossypium arboreum*| mRNA|length=3533bp_DCL2a**

ATGGAGCCAGTTGACATGGAGGAGGACATTTCTCAACTACACCCTGCTGATCCTCTCCCTTTTGCTTTAACAGACAGTCTTGTTCCTGATATTGTTTTTGAAGAACATGATGTGGAAGAAATTGGTAATAATAATGCATGTCCTTATTTTCTTTATCTCAAATAGTTAATGCATGTGATTTCAAGATATATAAAGTTGTTAGAAGAAGTATCTGATCTTCGTTTATTTTATTTAATTATTTGATTCGTAG AAAAGGAGCCCTATAATGATGATCAACCTATTTTCTTTCCGCCTGAACTAGTGAATAAGGGTTCACTGGACTCCATGACAAAGTACTACTGCTACTTAATGGAGTTGAAGCAGAACTTTGATTATGAGGTTCCTGTTCAGAACATCATGCTTCTTGTCAGGAATCAGTTTGACATGGATGAAAAAAGTATGAATATTGAGTTAGAAGTTGACAGGGGCACATTAACAGTTAACATGAAATATATTGGACTAAAATGTCTTAATTCTGACCAGGTACTATATATTTTTTAAATTTTATTATTGTTCTAGGGTGATAAGAATAGAAAATATATTGTAATGCGTTCATATTTTAACATTTTACCCTCTGTGCGCTTTTTCCATTTTGGTAGGTTATCTTATGTAGAAGGTTTCAGCTTGCAGTTTTTCAAGTACTTATGGATCGTAAAGCTGAAAAGTTGGCAGAGGTGTTATGTGACCATACCTTGGGGAATAATTCTGAAATTGATTATCTACTCCTCCCATCAAATTATGTGGGCCAGAGTCCTCTGATTGATTGGTTGTCAGTTACTTCTGTCACATTTTCTTATGAAAAGGCCTGCAAGAATCATGTGAACTGCAATGCTGATATATTGATACAGATCAAAAGCGGTCTGGTGTGCACTTGCATGATCCAAAATTCGTTGGTCACTACTCCTCATAATGGTCATGCTTATATTATCAGTGGTCTTTTAACTAATATAAATGCAAATTCACTTTTGAGGTTGAGTGATGGACGCTTAATGACTTACAAGGAATATTATGAAAAACGGTAAGCATTTCTTTAGTAATTTTCCAAAGAATTAATGTGAATATGCGATGCTTATGTTTCTGGTTATTAACTGTATTCTCATGTGACATTACAGCCACGGTATCAATTTATGTTATAGTCAAGTCTCTTTCCTTGCTGGGCGACAGATTTTCCGGGTGCAAAATCACATTCAAAGGCGCAGAAAACAGAAAGAGAGAGGCATGTTGTCTCCTATCATCTTGCCTTTTTGAGATTAGTAAGGATAGCTTTGGATGATCTTCGCAATTGAATTCAACTGTTAAGAACAAATTTTCATATTGGAAGATTTTTGTAGAGAAACTAAGATTTTCGTAATATCTGGTACAAAATAGGTTTTGTCCAAGTTTTCATACCGGTTCAGTTTATTTTAATATTAAATTTTTTATTTATGTTGCAGAATCAAGTAACCCATTCGTAGAATTGCCTCCTGAGCTTTGTTGTGTAGTAATGTCTCCCATATCGATCAGCACATTTTATTCTTTCACATTTCTTCCGTTAATCATGCATCGACTCGAGTCTTTGCTCCTTGCTACCAGCTTGAAAAAGATGCATTTGAATCATTGCGTGCAAAATGT TGCGATTCCAACCATGAAGGTGAGCTAGCATACTTTTGAATACCACTTTTTAGAGCCACCTATGCTTTACAGAAACATGTATTACATCTTAAAATTATCTGCTGAAGTATTATTGAGAAAACAAACAGTATGTATTACATCCTTGTGATATGTACAGTTAAGTGTTGCATTTTATATATTTGTGTTTGACTTGGCTCAACTATGCTTGGTAGGTTTTGGAGGCAATTACTACCAAAAAGTGCCTAGAAAACTTTCATTTGGAATCACTAGAGACTCTTGGTGACTCTTTTTTGAAGTATGCTGTTTGTCAACAGCTTTTCAAAAAATATCAAAATCATCACGAGGGCCTTCTTAGTATTAGGAAGGAAAAATTTATTTCAAATACAGCTCTGTCCATGCTAGGATGTGACAAGAAACTTCCGGTAAGTTGTCTTCTAGATATTTATTTAATTTATCCAATTGTGTTGTTATATAGTCAGTTCTCTCTTTAATATGCTTTGGAAACTGCAGGGGTTTATCCGTGATGAGCCTTTCGATCCCAAAGACTGGATGATTCCTGGTTATAATTGTGGAAGTTACTCATTAAATGAGGAAACACTGTGTAATGCAAAAAAAATATATGTTAGAGGAACAAGAAAGCTGAAGTTTAAGAAGGTTGCTGATGTTGTTGAGGCACTTATTGGTGCATACCTTAGCACGGGAGGCGAAGCAGCTGGATTACTATTTTTGGATTGGATCGGTATAAGTATTAATTTCACAAATATACCATATGAAAGACATTTTAAAGTGCGGGCCGAGAAGTTTGTTAATGTCCAGCACTTTGAGTCCCTTCTACACTATTCATTCCAAGACCCTTCTTTGTTGGTGGAAGCATTAACCCATGGTTCTTATATGCTAGCTGAAATTCCAGGATGTTATCAGGTGATAATCTGCCATACGAATGACACATTAATGGTGCAATTTTTTTTTTCATCATATGTATCTTAATGTCTTATAATCATGGGTAGTTGGTTCATTTTTCTCTCTCAAGTTTAAATTGAACATAATTAATACTTTTTCCTTGCAGCGGCTGGAATTTCTAGGGGACTCCGTTTTAGATT ATCTTATTACTCTGCATTTATATAATAAATATCCTGGGCTCACGCCAGGATTATTAACGGATCTTAGGTCAGCATCTGTTAACAACAATTGCTATGCATTATCAGCTGTAAAGGCTGGATTTCATAAGCACATTCTCCAAAGTTCACAGAAGCTTTACAAGGAAATAAAAGAAACCGTCGAAAGTTTTCAGGAATTATCGTTGGAATACACTTTCGGCTGGGAATCTGAAAAATCTTTCCCCAAGGTTTGCATTAGATTACGATTACCACTCTTGATTATTTACATAGCAATGCTCGCTTATCAGAGTTTTCTCCTTATTTTGTGAAGCAGTGTAGAGTAATGGGTTCTTAAAAGCATTATTTTTATCACCATTGCTCTCGCTTAGAGAAAAAATAATTGTTGTGAATGTTTCTTAAATGATCTTATTATGACAGGTACTCGGTGACGTGATGGAGTCACTTGCTGGAGCTATTTTTGTTGATTCAGGATACAATAAGGAAATCGTATTCCAGAGTATAAGGCCACTGTTGGAGCCCATGATTACTCCGGAGACTCTGAAGGTTCACCCTGTCAAAGAGCTATATGAGCTCTGTCAAAAGCAGCATTATGAACTAAGAACACCCATTGTTTCACATGAGGATGGCATGTCTTCTATTACGATAGAGGTTGAAGCAAATGGGAAGGTATTCAAGCACACATCCACAGTTTGTGATAAGAAGATGGCCAAGAAAGTGGCTTCCAAAGAAGTT TTGAAGTCTCTAAAGGGAGCCAGTTGTAGCTAG

**>Cotton_A_34031_GaDCL2a-1_ protein sequence length=770aa**

MEPVDMEEDISQLHPADPLPFALTDSLVPDIVFEEHDVEEIEKEPYNDDQPIFFPPELVNKGSLDSMTKYYCYLMELKQNFDYEVPVQNIMLLVRNQFDMDEKSMNIELEVDRGTLTVNMKYIGLKCLNSDQVILCRRFQLAVFQVLMDRKAEKLAEVLCDHTLGNNSEIDYLLLPSNYVGQSPLIDWLSVTSVTFSYEKACKNHVNCNADILIQIKSGLVCTCMIQNSLVTTPHNGHAYIISGLLTNINANSLLRLSDGRLMTYKEYYEKRFHTGSVYFNIKFFIYVAESSNPFVELPPELCCVVMSPISISTFYSFTFLPLIMHRLESLLLATSLKKMHLNHCVQNVAIPTMKVLEAITTKKCLENFHLESLETLGDSFLKYAVCQQLFKKYQNHHEGLLSIRKEKFISNTALSMLGCDKKLPGFIRDEPFDPKDWMIPGYNCGSYSLNEETLCNAKKIYVRGTRKLKFKKVADVVEALIGAYLSTGGEAAGLLFLDWIGISINFTNIPYERHFKVRAEKFVNVQHFESLLHYSFQDPSLLVEALTHGSYMLAEIPGCYQRLEFLGDSVLDYLITLHLYNKYPGLTPGLLTDLRSASVNNNCYALSAVKAGFHKHILQSSQKLYKEIKETVESFQELSLEYTFGWESEKSFPKVLGDVMESLAGAIFVDSGYNKEIVFQSIRPLLEPMITPETLKVHPVKELYELCQKQHYELRTPIVSHEDGMSSITIEVEANGKVFKHTSTVCDKKMAKKVASKEVLKSLKGASCS

**>Cotton_A_34032_BGI-A2_v1.0_ID=Cotton_A_34032_BGI-A2_v1.0_ *Gossypium arboreum*| mRNA|length=3723bp_DCL2a**

ATGGAGCCAGTTGACATGGAGGAGGATATTTCTCAACTACTCCCTGCTGATCCTCTCCCTTTTGCCAGAAGCTACCAGCTTGAAGCATTAGAGAAAGCTATCAAGCAAAACACAATAACTTACTTGGAAACTGGATCTGGCAAGACAATGATCGCCATCATGCTTCTCCGCAGCTATGCTTATCTTATCCGCAAGCCCTCACCCTTTTTCGCTGTGTTCTTGGTTCCCAAAGTTGTTCTGCAAGCTGATGCTGTGGAAATGCATACGGACTTGAATGTTGGAAAGTATTGGGGAGATATGCAGGTCGACTTTTGGGATGGGGAGAAGTGGAAACAAGAAATAGATAAATATGAGGTGCTTGTGATGACACCTCAAATTTTACTTGATGGATTGAGGCATAGCTTCTTCAAGATAAACATGATAAAGGTTTTGATAATTGATGAATGCCATCATGCCCGAGGAAAGCATCCTTATGCCTCTATTATGAGAGAATTCTATCATCGCCCGTTAGAAGCTGGTGCATCTAATCTTCCTAGGATTTTTGGGATGACTGCTTCTCCTATAAATTCAAAAGGGGCAAACTCTGCTGATAGCTATTGGCAGAAGATCCATGAATTGGAGACTATTATGAACTCAAAGGTGTATACATGTGTAAGTGCATCAGTGCTTGCTCAGTTTGTTCCATTTGCGACTCCAAAGTTCAAGTTTTACCAACATATGGCAATTCCAAATGTTTTATATTCACACTTGGTAGAGGAATTGACTGTTTTGAAAGTAAAGCATGAATGTTCGTTGGATAATTTGGATCTTGAAGCTTCTGCAGCAGAATCTACAAGAAAGAAACTATCAAAGATACATTCAGCTTTAATACATTGTTTATATGAGCTTGGTGCTTGGTTGGCTCTGAAGGCTGCAGAGTGCTTTTCATGTTATGAAAGTGAGCTTCTTATGTGGGGGAAATTGGATGTCTTTGGCGAGAAAATTATTAGGAGCTACAGCGTGGATGCTTTTCGTGCAATTGAAACATGCATGCCATCTGGTCTGGATTGGACCATAGCTAATGATGTTAAAGGCAGTGTGGCTGCTGGGTTTCTTACCACAAAAGTTTTATGCCTTATTGAATCTTTATTCGAATACAGGGTGTTGAAGGACATAAGATGTATAATTTTTGTTGAGAGGGTTATAACAGCTGTTGTGCTACAATCGCTATTCAGTGAATTGCTTCCCAGGTACAGTAATTGGAAGACTAAGTACATTGCGGGAAATAACTCTGGATTGCAGAATCAGACAAGGAAAAAACAAAATGAAATTGTAGAAGAATTCCGCAAAGGCATGGTTAACATAATCGTTGCAACTTCAATTCTTGAAGAAGGCTTGGATGTTCAATCATGCAACCTAATTATCAGATTTGATCCTTCACCAACAATTTGTAGTTTCATACAGTCTCGAGGACGTGCTAGAATGCAGAACTCAGATTATCTATTAATGCTGAAGAGCGGGGATTTTTCTACACATTCTCGACTGAAGAACTATCTTACTAGTGGAGATGTAATGAGAAAGGAATCTTTATGCCATGCATCATATCCTTGTTCTGCTCTTTGTGAGGGCTTAGATGATGAAGAGTTTTACCATGTTGCGAGTACAGGGGCATGTATGACTCTTAGTTCTAGTGTTGGTCTAATGTACTTCTATTGCTCACGTCTCCCTGCAGATGGGTATTTTAAACCTATTCCGAGGTGTGTTATAGACAAGCAAATGGGGCTTTGCACCCTCCTTCTACCCAAGAGTTGCCCTATACAAACTTTTTGTGTTCAGGGTGATATAAAAAACATAAAGAAAAAAGCATGCTTTGAAGCATGCAAGAAACTCCATCAAATTGGTGCTTTAACAGACAATCTTGTTCCTGATATTGTTTTTGAAGAACATGATGTGGAAGAAATTGAAAAGGAGCCCTATAATGATGATCAACCTATTTTCTTTCCACCTGAACTAGTAAATAAGGGTTCACTGGACTCCATGACAAAGTACTACTGCTACTTAATGGAGTTGAAGCGGAACTTTGATTATGAGGTTCCTGTTCATAACATCATGCTTCTTGTCAGGAATCAGTTTGACATGGATGAAAAAAGTGTGAATATTGAATTAGAAGTTGACAGGGGCACATTAACAGTTAACATGAAATATATTGGACTAATACGTCTTAATTCTGACCAGGTTATCTTATGTAGAAGGTTTCAGCTTGCAGTTTTTCAAGTACTTATGGATCGTAAAGCTGAAAAGTTGGCAGAGGTGTTATGTGACCATACCTTGGGGAATAATTCTGAAATTGATTATCTACTCCTCCCATCAAATTATGTGGGCCAGAGTCCTCTGATTGATTGGTTGTCAGTTACTTCTGTCACATTTTCTTATGAAAAGGCCTGCAAGAATCATGTGAACTGCAATGCTGATATGATACAGATCAAAAGCGGTCTGGTTTTGGAGGCAATTACTACCAAAAAGTGCCTAGAAAACTTTCATTTGGAATCACTAGAGACTCTTGGTGACTCTTTCTTGAAGTATGCTGTTTGTCAACAGCTTTTCAAAAAATATCAAAATCATCACGAGGGCCTTCTTAGTATTAGGAAGGAAAAAATTATTTCAAATACAGCTCTGTCCATGCTAGGATGTGACAAGAAACTTCCGGGGTTTATCCATGATGAGCCTTTCGATCCCAAAGACTGGATGATTCCTGGATATAATTGTGGAAGTTACTCATTAAATGAGGAAACACTGTGTAATGCAAAAAAAATATATGTTAGAGGAAGAAGAAAGCTGAAGTTTAAGAAGGTTGCTGATGTTGTTGAGGCACTTATTGGTGCATACCTTAGCACGGGAGGCGAAGCAGCTGGATTACTATTTTTGGATTGGATCGGTATAAGTATTAATTTCACAAATATACCATATGAAAGACATTTTAAAGTGCGGGCCGAGAAGTTTGTTAATGTCCAGCACTTTGAGTCCCTTCTACACTATTCATTCCAAGACCCTTCTTTGTTGGTGGAAGCATTAACCCATGGTTCTTATATGCTTGCTGAAATTCCAGGATGTTATCAGCGGCTGGAATTTCTAGGGGACTCCGTTTTAGATTATCTTATTACTCTGCATTTATATAATAAATATCCTGGGATCACGCCAGGATTATTAACGGATCTTAGGTCAGCATCTGTTAACAACAATTGCTATGCATTATCAGCTGTAAAGGCTGGATTTCATAAGCACATTCTCCAAAGTTCACAGAAGCTTTACAAGGATATAAAAGAAATCGTTGAAAGTTTTCAGGAATTATCGTTGGAATACACTTTCGGCTGGGAATCTGAAAAATCTTTCCCCAAGGTACTCGGTGACGTGATGGAGTCACTTGCTGGAGCTATTTTTGTTGATTCAGGATACAATAAGGAAATCGTATTCCAGAGTATAAGGCCACTGTTGGAGCCCATGATTACTCCGGAGACTCTGACGGTTCACCCTGTCAAAGAGCTATATGAGCTGTGTCAAAAGGAGCATTATGAACTAAGAAAACCCATTGTTTCACATGAGGATGGTATTTCTTCTATTACGATAGAGGTTGAAGCAAATGGGAAGGTATTCAAGCGCACATCCACAGCTTGTGATAAGAAGATGGCCAAGAAACTGGCTTTCAAAGAAGTTTTGAAGTCTCTAAAGGGAGCAAATTTCAGCTAG

**>Cotton_A_34032_GaDCL2a-2_ protein sequence length= 1240aa**

MEPVDMEEDISQLLPADPLPFARSYQLEALEKAIKQNTITYLETGSGKTMIAIMLLRSYAYLIRKPSPFFAVFLVPKVVLQADAVEMHTDLNVGKYWGDMQVDFWDGEKWKQEIDKYEVLVMTPQILLDGLRHSFFKINMIKVLIIDECHHARGKHPYASIMREFYHRPLEAGASNLPRIFGMTASPINSKGANSADSYWQKIHELETIMNSKVYTCVSASVLAQFVPFATPKFKFYQHMAIPNVLYSHLVEELTVLKVKHECSLDNLDLEASAAESTRKKLSKIHSALIHCLYELGAWLALKAAECFSCYESELLMWGKLDVFGEKIIRSYSVDAFRAIETCMPSGLDWTIANDVKGSVAAGFLTTKVLCLIESLFEYRVLKDIRCIIFVERVITAVVLQSLFSELLPRYSNWKTKYIAGNNSGLQNQTRKKQNEIVEEFRKGMVNIIVATSILEEGLDVQSCNLIIRFDPSPTICSFIQSRGRARMQNSDYLLMLKSGDFSTHSRLKNYLTSGDVMRKESLCHASYPCSALCEGLDDEEFYHVASTGACMTLSSSVGLMYFYCSRLPADGYFKPIPRCVIDKQMGLCTLLLPKSCPIQTFCVQGDIKNIKKKACFEACKKLHQIGALTDNLVPDIVFEEHDVEEIEKEPYNDDQPIFFPPELVNKGSLDSMTKYYCYLMELKRNFDYEVPVHNIMLLVRNQFDMDEKSVNIELEVDRGTLTVNMKYIGLIRLNSDQVILCRRFQLAVFQVLMDRKAEKLAEVLCDHTLGNNSEIDYLLLPSNYVGQSPLIDWLSVTSVTFSYEKACKNHVNCNADMIQIKSGLVLEAITTKKCLENFHLESLETLGDSFLKYAVCQQLFKKYQNHHEGLLSIRKEKIISNTALSMLGCDKKLPGFIHDEPFDPKDWMIPGYNCGSYSLNEETLCNAKKIYVRGRRKLKFKKVADVVEALIGAYLSTGGEAAGLLFLDWIGISINFTNIPYERHFKVRAEKFVNVQHFESLLHYSFQDPSLLVEALTHGSYMLAEIPGCYQRLEFLGDSVLDYLITLHLYNKYPGITPGLLTDLRSASVNNNCYALSAVKAGFHKHILQSSQKLYKDIKEIVESFQELSLEYTFGWESEKSFPKVLGDVMESLAGAIFVDSGYNKEIVFQSIRPLLEPMITPETLTVHPVKELYELCQKEHYELRKPIVSHEDGISSITIEVEANGKVFKRTSTACDKKMAKKLAFKEVLKSLKGANFS

**>scaffold3086__BGI-A2_v1.0_ID=Cotton_A_34032_BGI-A2_v1.0_ scaffold3086:32023..41620_ *Gossypium arboreum* _ mRNA_ length=4207_ DCL2b**

ATGCTTGTGATAATTTATCAGAACATTTTACATATATATTTATGCATACATGGTATGGGAGATGCATTAGATATATATGATGGTTCCATCTAGCTAGTTTAGGAATCCTAGTTACCTACCATATGCTTGGGCTAAGAGGAATGAAAAATCGTACTCATGGAATATTATTTATCCTTCCGGGTGTCTGTTGAAATTTGAAGGCATGAATGTGATTCTTTCTCATACTTGGATATGCATTTGTTTTTACTATTAGGATAATGACAATAGATGTACTGGTTTTCAGCATGAATGTTCGTTGGACAATTTGGATCTTGAAGCTTCTGCAGCAGAATCTACGAGAAGGAAAATATCAAAGATGCATTCAGCTTTAATACATTGTTTGGACGAGCTTGGTGTCTGGTTGGCTTTAAAGGTACCTTTTTAGACAATAATTTCTGCCAAAGTGCCATATATGTATATCATTGAGTTTGTTGCTGTTGTAATAGGCTGCAGAGTACTTGTCATGTTATGAAAGTGAGTTTCTTATGTGGGGGAAATTGGATGTCTTTGGCGAGAAAATTGTTAGGAGTTACACTGTGGATGCTTTCCATGCCATTAGAACATGCATACCATCTGGTACTTGTATATTCCTCTAATGGCGAGTTAGTAATATGTTGCTCTATCATTTTTCTTAAATACTCTTTCTCTCTCTTGTTTCATCATATAGATCCAGATTGGACCATCGTTAATGATGTTAAAGCCAGTGTGGATGCTGGGTTTCTTACCACAAAAGTTTTATGCCTTATTGAATCTCTTTCTGAATACAGGTAACAGAGTGTTTACTAAGTGGTAAGATAATATTGTCTCCCTCGAAAGTTATCCTTCTGCATTCCAATTATTTGAGGTTTATTTTGGGACGATAGTCTGATGCATTAATGTCTTGAATACAGAAACGATTTAAGGAATGGTTTGTTGTGTTTTTTTTTTACTGTGGTTATCAAAAACATTTCGCTTGGATTTTATTTACTTTAAAACATAAATAACTTAATTTAATGAGGTCAATAATATAAATGTATAAAGGGTTTTAAAAACTGAATTTGAAAAGACTGATCTACAGCCCCATTCATTCCATCTCCCTCATTTTTGCTCTGTTTTGCGATGTTTCTTCTTGTTTCATTCTGTTATGTGCTACAATGCAGGGTGTTGAAAGACATAAGGTGTATAATTTTTGTGGAGAGAATTATAACAGCAGTTGTGCTTCAATCACTATTCAGTGAATTGCTTCCGAAGTACGGTAACTGGAAGACTAAATACATTGCAGGAAATAACTCTGGATTGCAGAACCGGACTAGGAAAAAACAAAATGAAATTGTAGAAGAATTCCGAAAAGGCATTGTAAACGTAATTGTTGCAACTTCGATTCTTGAGGAAGGCTTGGATGTTCAAGCATGCAACTTAATTATCAGATTTGATCCTTCGCCGACAGTTTGTAGTTTTATACAGTCTCGAGGACGTGCTAGAATGCAGAATTCAGATTATCTATTAATGGTGAAGACCGGGGATTTTTCTACTCATTCTCGACTGAAGAACTATCTTACTAGTGGAGATGTAATGAGAAAGGAATCTCTGTGCCATGCATCTGATCCTTGTTCTCCTCTAAGCAATGACTTATGTGATGAAGAGTTCTACCATGTTGCAAGTACAGGGGCATTTATGACTCCTAGTTCAAGTGTTGGTTTGATATACTTCTATTGCTCACGCCTCCCTACAGACAGGTTATTTTAAACCTACTCTGAGGTGTATCATAGACAAGCAAATGGGGATTTGCACCCTCCATCTACCCAAGAGTTCCCCTATACAAACTGTTTGTGTTAAGGGAAACTTCAAAAGCCTAAAGCAGAAGGCATGCTTTGAAGCATGCAAGCAACTCCATCAATTTGGTGCTTTGACAGATAATCTTGTGCCTGATATTGTTGTGGAAGAAAATGATGCTGAAGAAATTGGAAAGGAATCCTATAATGATGATCAACCGATTTTTTTTCCTCCTGAACTGGTGAATCAAGATTCACAGGAGTCCATGACAAAATACTACTGCTACTTAATGGAGTTGAAGCAGAACTTTGGTTATGAGATTCCTGTTCATAACATCATACTCCTTGTCAGGAGTCAGCTTGAAATGGAAGCAAAAAGTATTGAGTTAGAAGTTGATAGGGGCACTCTGACAGTTAACTTGAAATATATTGGGCTGATACGTCTTAGTTCTGACCAGGTTATCTTGTGTAGAAGGTTTCAGATTGCTCTTTTTCGAGTTCTTATGGATCATAAAGCAGAAAAGTTGACAGAGTTGTTATCTGACCTTACGTCAGGGAACAATTCTGAAATCGATTATCTTCTTCTCCCATCAAATTACATGGGCCAACCCGTGATTGATTGGTTGTCAATTTCTTCCATCTTATTTTCTTATGAGAAGGTCTGGAAGAATCATGTGAACTGCAATGCTGGCATGATACAGACCAAAAGTGGTCTGGTGTGCACTTGCATGATAAAAAATTCGTTGGTCTCTACTCCTCATAATGGTCACACATATATTATTGACGGTCTTTTAAATAATATAAATGCAAATTCACTTTTGACACTGAGTGATGGAGGAGTAATGACCTACAAGGAATATTATGAACAACGGCATGGTATCCATTTATGTTTTAGTCGAGTCTCTTTCCTTGCTGGGCGACACATTTTCCCCGTGCAAAATCACATTCAAAGGTGCAAAAAACAGAAAGAGAAAGAATCCAGTAACGCATTTGTAGAATTGCCTCCTGAGCTTTGTGATGTAATAATGTCTCCCATATCGATTAGCACATTTTATTCTTTCACATTTCTTCCATCAATCATGCATCGACTCGAGTCTTTACTCCTTGCTACCAGCTTGAAAAAGATGCTTCTCGATCATTGTGTCCATAATGTTGCTATTCCAACCATGAAGGTTTTGGAGGCAATTACCACAAAAAAGTGCCTGGAAAGTTTTCATTTGGAATCACTGGAGACTCTTGGTGATTCTTTTTTGAAGTATGCTGTTTGTCAACAGCTATTCAAAAAACATCAGAATCATCATGAGGGCCTTCTTAGTATTAGGAAGGACAAAATGATTTCAAATACAACCCTTTGCATGCTGGGATGTGACAAGAAACTTCCGGGATTTATCCGTGATGAGCCTTTCGATCCCAAAGGTTGGATGGTTCCTGGTTATAATTGTGGAAGATATACATTAGATGAGGAGACATTGAATACAAGAAAAATATATGTTAGTGGGAGAAGGAAGCTGAAGAGTAAGAAGGTTGCTGATGTTGTTGAGGCTCTTATCGGTGCATATCTTAGCATGGGAGGTGAAGCTGCTGCATTATTATTTTTGAATTGGATTGGTATAACTATTGATTTTAGAAATATACCGTACGAAAGACATTTCGAAGTGCAGGCTGAAAAGTTTGTTAATGTCCAACATTTAGAGTCTCTTCTTAACTATTCATTTCAAGACCCTTCTTTTTTAGTGGAAGCACTAACACATGGTTCTTACATGCTTGCTGAAATTCCAGGATGTTATCAGCGGCTGGAATTTCTAGGGGACTCCGTTTTAGATTATCTCATCACTCGGCATTTATATAATAATTATCCTGGGATCTCACCAGGATTATTAACGGATCTGAGGTCAGCATCTGTTAACAACAATTGCTATGCATTATCAGCTGTTAAGGCTGGATTGCATAAGCACATTCTCCAAAGTTCACAGAAGCTTTACAAGCATATAAAAGAAACTGTTGAAAGTTTTCGGGAATTATCGTTGGATTGCACTTTCGGTTGGGAATCTGAAAAATCTTTCCCCAAGGTACTGGGTGATGTGATGGAGTCACTTGCTGGGGCTATATTTGTTGATTCAGGATACAACAAGGAAACTGTATTCCGGAGTATCAGGCCACTGTTGGAGCCCCTGATTACTCTAGAGACTATGACGGTCCACCCCGTGAAGGAGTTGAATGAACTTTGTCAAAAGAAGCATTATGAACAAAGAAAACCCATTGTTTCACATGACAATGGGGTTTCTTCTGTTACCATAGAGGTTGAAGCAAATGGCGAGGTATTAAAGCACACATCAACAGCTTCTGATAGGAAGATGGCCAAGAAACTGGCTTCCAAAGAAATTTTGAAGTCCCTAAAGGGAGCCAATTTTTGCTAG

**>scaffold3086_DCL2b_protein sequence length= 1403aa**

MLVIIYQNILHIYLCIHGMGDALDIYDGSI*LV*ES*LPTICLG*EE*KIVLMEYYLSFRVSVEI*RHECDSFSYLDMHLFLLLG**Q*MYWFSA*MFVGQFGS*SFCSRIYEKENIKDAFSFNTLFGRAWCLVGFKGTFLDNNFCQSAIYVYH*VCCCCNRLQSTCHVMKVSFLCGGNWMSLARKLLGVTLWMLSMPLEHAYHLVLVYSSNGELVICCSIIFLKYSFSLLFHHIDPDWTIVNDVKASVDAGFLTTKVLCLIESLSEYR*QSVY*VVR*YCLPRKLSFCIPII*GLFWDDSLMH*CLEYRNDLRNGLLCFFFTVVIKNISLGFYLL*NINNLI**GQ*YKCIKGFKN*I*KD*STAPFIPSPSFLLCFAMFLLVSFCYVLQCRVLKDIRCIIFVERIITAVVLQSLFSELLPKYGNWKTKYIAGNNSGLQNRTRKKQNEIVEEFRKGIVNVIVATSILEEGLDVQACNLIIRFDPSPTVCSFIQSRGRARMQNSDYLLMVKTGDFSTHSRLKNYLTSGDVMRKESLCHASDPCSPLSNDLCDEEFYHVASTGAFMTPSSSVGLIYFYCSRLPTDRLF*TYSEVYHRQANGDLHPPSTQEFPYTNCLC*GKLQKPKAEGML*SMQATPSIWCFDR*SCA*YCCGRK*C*RNWKGIL***STDFFSS*TGESRFTGVHDKILLLLNGVEAELWL*DSCS*HHTPCQESA*NGSKKY*VRS**GHSDS*LEIYWADTS*F*PGYLV*KVSDCSFSSSYGS*SRKVDRVVI*PYVREQF*NRLSSSPIKLHGPTRD*LVVNFFHLIFL*EGLEESCELQCWHDTDQKWSGVHLHDKKFVGLYSS*WSHIYY*RSFK*YKCKFTFDTE*WRSNDLQGIL*TTAWYPFMF*SSLFPCWATHFPRAKSHSKVQKTERERIQ*RICRIAS*AL*CNNVSHID*HILFFHISSINHASTRVFTPCYQLEKDASRSLCP*CCYSNHEGFGGNYHKKVPGKFSFGITGDSW*FFFEVCCLSTAIQKTSESS*GPS*Y*EGQNDFKYNPLHAGM*QETSGIYP**AFRSQRLDGSWL*LWKIYIR*GDIEYKKNIC*WEKEAEE*EGC*CC*GSYRCIS*HGR*SCCIIIFELDWYNY*F*KYTVRKTFRSAG*KVC*CPTFRVSS*LFISRPFFFSGSTNTWFLHAC*NSRMLSAAGISRGLRFRLSHHSAFI**LSWDLTRIINGSEVSIC*QQLLCIISC*GWIA*AHSPKFTEALQAYKRNC*KFSGIIVGLHFRLGI*KIFPQGTG*CDGVTCWGYIC*FRIQQGNCIPEYQATVGAPDYSRDYDGPPREGVE*TLSKEAL*TKKTHCFT*QWGFFCYHRG*SKWRGIKAHINSF**EDGQETGFQRNFEVPKGSQFLLX

**>Cotton_A_31241_BGI-A2_v1.0_ID=Cotton_A_31241_BGI-A2_v1.0_ *Gossypium arboreum*| mRNA|length=4980bp_DCL3a**

ATGCATTCTCCAAAGGAGGAACAGGGTGAGCCTCTAAAACGAAGTTTCAGTGAGGCGAACTCAGACCCACCTGATTGGATGGTTCTAGGCGACAATGAAACTGCTAAGGAAGAAGACCCTTCTTCATCTTCAAAACCTAAGGACTTTAACCCCAGAGGGTATCAATTGCAGGTATATGAGGTGGCAAAGAGAAGAAACATAATAGCAGTGTTGGATACAGGTGTAGGGAAGACAATGATAGCTGTGATGCTCATAAAGGATTTTGGTCAAGCTATCGAGTCTACTGAAAGTAAAAAATTGATTATTTTCTTGGCTCCCACTGTTCATCTAGTCAATCAGCAATTTGAATATATAAAAGATCATACTGGTTTGGAAGTTGAACAGTATTATGGAGCTAAGGGGGTTGATGAATGGACCTTGGATTGTTGGGAGAAAGAAACAAAGGAGCATGATGTTCTGGTTATGACACCCCAAATTCTATTGGATGCCTTAAGGAAGGCATTCTTGAGTCTGGATATGGTGTCCTTAATGATAATAGACGAGTGCCATCGTGCTACTGGCAACCATCCATATGCCAAAATAATGAAGGAATTTTATCACAAATCTAATAACAAGCCAAAGATTTTTGGAATGACAGCATCACCTGTAGTTAGCAAAGGTGTGTTGTCCAGCAATAATTGCGACGGTCAAATGTCAGAACTTGAATGTGTTTTGGATTCCTTGATATACACTATTGAAGACAGGACGGAGATGGAAGCGTGTGTTCCTTCTGCTAAAGAAAGTTGTAGATTTTTTGATCCAGCACAATTTTCTAGCTTGGATTTGAAAGCAAAGGTAGAAGCCTCTTGGTTAAAGACAGATGAGTCATTGTCAAATTTGCAAAGCTCACTGCAAACTTCTCATAAAGACATGGATGATAAACTTAAGAATCTACGGAAGCGATTATCAAATGACCATGCTAAGGTTCTGCATTGCCTTGATAATCTTGGTCTCATATGTGCTTATGAGGCTGTTAACATTTGTCTAGAGAACATCCTTGACACCACAGAGGAATCTAAAGCATATAGAGAAAGTGTGTTGCAGTATAAAAATTTCCTTGAGGAAGTCCAATGTAGAATTGGGGAGTCTCTTCCCCTCGGTGATAAAAATTTTCTGAATTCTGGATTTGACTATTTAAAGGCAGTAGATCTTGGCTATATTTCTCCAAAACTGCATGAACTACTTCAACTGTTCCAATCATTTGGAAAAACTAGACAAGTATTGTGCCTGATTTTTGTTGAAAGAATTATTACTGCTAAAGTAATTGAAAGATTTGCAAAAAAAGTAAGCTGTTTATCACATTTCATGGTTTCATATATGACTGGAAGTAATACATCTGTTGATTCCCTGGCACCAAAAATACAAAAGGAAACCTTGGAGTCATTTCGATCTGGGAAGGTGAATCTATTGTTTACTACTGATGTGGTTGAGGAGGGAATTCATGTACCAAATTGCTGTTATGTGATACGTTTCGACCTGCCAAAGACAGTCCGGAGTTATGTACAATCTCGAGGAAGAGCTAGGCAGAACAATTCTGAATTCATTATGATGCTTGAGAGGGGAAATATGAAACAAAGAAATCAACTATATGATATAATCAGAAGTGAGTATTCAATGACAAATTTGGCAATTAAAAGAGATCCCGATTCTGATCCATGCCTTCTTAAAGATCATACATTTGAAGAAACAAATGTTTTTATTGTGGATGCTACTGGAGCTTCAGTTACTACAGATTCTGCTGTTAGCCTCATCCATAAATATTGTGGGAAGCTCCCCGGTGATAAGTATTACACACCAAAGCCAAATTTCCAGTTCATGTCTTCTGAAGGATTATATAAGTGTAAATTAACATTACCTGTTAATGCAGCCGTTCAAACAATAGTTGGTCCACCATCTAGGAATTCTCATTTAGCAAAGCAGCTTGTATGCTTAGAAGCATGTAAGCAGCTTCATCAAATGGGTGCCTTGGATGATCATCTCACTCCATCCATTGAAGAGCCTTCAGAAAATGCTTGTGTTTCTAAAGGAAAAGATTCAGGTGCAGGTGCAGGAACTACAAAACGGAAGGAGCTTCATGGAACAAGTTGCATACAGGCATTATGTGGAAGCTGGGGAGAAAAATCTGATGATGCTGTTTTCTTTGCCTATAAATTTGACTTCAAATGCAATATCATTACTGTGGTTTATTCTGGATTTGTTCTTCTAATTGAATCAAAGCTTGCGGATGATGTGGGGAATACTGAAATGGATCTTTTCTTGATTGGTAAGATGGTTAAGGCCAGTGTTTCTTCCTGTGGGCAAGTGCATTTGAATGCAGAACAGATGATGAAAGCGAAGCGCTTTCAGGAATTTTTCTTTAATGGCTTGTTTGGTAAGTTATTTGTTGGATCTAAATCATCTGGAGCACCAAGAGAATTTCTACTTCGGGATAAATCAAGCTCATTGTGGAGTCCATCCCGCATGTATTTGCTTTTGCCCCTCGAGGATAATTCAACTGATGAATTGAGAATACACTGGCCAGGGATAACAGCTTGCACATTAGCTGCAGAATTTTTAAATAAAAATTCTTTGTTAGGCACTGAGCAATCTGATGATGGTGGAAGCAATCCATCATTGAACAGTACTGGTTCACCTGTGACAGACTGCAAAGAAACCAATATAATCCGCTTTGCTAACAGTTCAGTTGATGCTAATAGTCTTAGAAATACGGTAGTATTGGCTATTCACACTGGAAGAATCTACTGCATCATTGAAGCTGTGAGTGATAAAACTGCTGAAAGTTCTTTCGCTGAAACTGTTGATACGGTCTCATCAGAGTTTGCTAACTTCTATGAATACTTCTACAAAAAGTACAATATTGTGCTGAAACATCCAGGACAGCCTTTGATGCTGTTAAAGCAGAGCCATAACCCGCACAACTTGCTTGTGAATTTTAATGATGAAGGTGTATCAGCTAAGGCATCACAAGCTGGCGTGGTTAATGAAAAGCCTCGGTTTCATGTCCACATGCCCCCTGAGCTTTTACTTGTCCTTGATGTCCCAGTGAGTGTTCTAAAATCATTGTACTTACTGCCATCATTGATGCATCGGCTGGAGTCCTTAATGTTGGCCAACCAACTCAGAGAAGAGATAAACTTTTGTTCTAGCAACATTGATATTCCAAGCTCAATGATCCTGGAAGCACTAACAACACTTAGATGCTGTGAAAGTTTTTCAATGGAAAGGCTGGAATTGCTTGGGGATTCAGTTCTGAAGTATGCTGTTAGCTGCCACCTCTTTCTTAGATATCCCAACAAACATGAGGGACAATTATCTGCCAGGCGTTCGTTGGCAGTTTGTAATTCAACCCTGCATAAGTTGGGAACTGACCACAAAATACAGGGATACATTAGGGACAGTGCTTTTGATCCCCGTCGTTGGGTAGCTCCAGGACAACGAGTGCTTCGCCCTGTTCCTTGCAAGTGTGGTGTGGACTCTCTAGAAGTTCCTTTAGATAAAAAATTTCAGACAGAAGACCCGAAAGTTAAGGTTGGTAAATCCTGTGATAGAGGCCACCGATGGTTCTGTTCAAAAACCATATCAGATTGTGTTGAAGCTCTCATAGGAGTATACTACTTAAGTGGTGGACTTGTTGCTGCCCTTCATGTGATGAAGTGGCTCGGGATTGATGCCGAAGTTGATCCCTCGGTGGTAGCTGAAGTCATTAATCAGGCATCCTTAAGAACTTATGTCCCCAATTACAAAATCCACATGATAGAGTCAAAAGTTGGCTACAATTTTTCTTTCAAGTTTTTCTTGCAGGAGGCATTGACACATGAATCTGTGCATGAGTCCTACTGTTACCAGAGGCTTGAATTTCTTGGTGATTCTGTGTTGGATTTGCTGATCACCCAGTATCTCTACAACCATCACACTGATATAGATCCGGGTGAGTTGACTGACCTGCGCTCTGCTTCGGTTAATAATGCAAATTTTGCTCAAGTTGCCGTGCGCCATGACCTTCATAAGCATCTTCAACATTGTTCAACTTTACTATCAAACCAAATAAGTGAATATGTGCAGTCCTTCACTGAATCTGATAACACCACCAGATTGGATCCCAGCATAAAAGGTCCCAAGGCTCTTGGAGACCTGGTTGAAAGTATTGTTGGGGCAATTCTGATTGATACAAACCTGAATCTTGGAAAAGTCTGGAGAATTGTTGAACCGTTGTTGTCTCCAATTGTAACCCCAGATAAGCTTGAGCTGCCTCCATTCCGGGAACTGAATGAATTATGCGACTCTCTTGGATATTTTATTAAGGAAAAATGTATAAATAAGGGGGAAGTGGTGCACGCCGAGCTTCTATTGCAGCTAGATCATGATTTGTTGGTAGGAGAGGGGTTTGATAGAAGTAGAAAAGTAGCAAAAGGAAAAGCCGCTTCTTGTCTGTTGAAGCACCTTGAGAACAGAGGAATCTCCCGGAAGAAAAGGAAACATGACTATGTGGATTCAAGTCAAACCATGGATGATGACTCGTTAGAACCAACAATCCCTAAGATGCAAAGGAGAGCCGAAATTCAGTTGCTTGATGAATCAAAGAAAGCATGCAGTGCTACGCCTGCCACCCCAGTTATTGTAACGGTAAAAACGAAGAAAGGAGGGCCGAGGACCACTCTTTTTGAGCTTTGCAAGAAACTGCTGTGGCCTATGCCTATGTTTCGAAGCCTCACTATTACTTTCCTTTGTAGTGCTCCCATGGAAATTGGTGAAGGTCCTGAGAGGAAAAAAGGGTTTATCAGCTTTGTGTCGAAAATCATCCTGAATGTCCCAGGATACGACATTATTGAATGCACTGGAGATGCAAAAGCTGACAAAAAAAGTTCTTCAGACTCAGCAGCACTGTTTATGCTTTATGAGCTTGAACAGCGCGGGAAGCTCATCATTGAGGAAACCTTATAA

**> Cotton_A_31241_GaDCL3a_ protein sequence length= 1659aa**

MHSPKEEQGEPLKRSFSEANSDPPDWMVLGDNETAKEEDPSSSSKPKDFNPRGYQLQVYEVAKRRNIIAVLDTGVGKTMIAVMLIKDFGQAIESTESKKLIIFLAPTVHLVNQQFEYIKDHTGLEVEQYYGAKGVDEWTLDCWEKETKEHDVLVMTPQILLDALRKAFLSLDMVSLMIIDECHRATGNHPYAKIMKEFYHKSNNKPKIFGMTASPVVSKGVLSSNNCDGQMSELECVLDSLIYTIEDRTEMEACVPSAKESCRFFDPAQFSSLDLKAKVEASWLKTDESLSNLQSSLQTSHKDMDDKLKNLRKRLSNDHAKVLHCLDNLGLICAYEAVNICLENILDTTEESKAYRESVLQYKNFLEEVQCRIGESLPLGDKNFLNSGFDYLKAVDLGYISPKLHELLQLFQSFGKTRQVLCLIFVERIITAKVIERFAKKVSCLSHFMVSYMTGSNTSVDSLAPKIQKETLESFRSGKVNLLFTTDVVEEGIHVPNCCYVIRFDLPKTVRSYVQSRGRARQNNSEFIMMLERGNMKQRNQLYDIIRSEYSMTNLAIKRDPDSDPCLLKDHTFEETNVFIVDATGASVTTDSAVSLIHKYCGKLPGDKYYTPKPNFQFMSSEGLYKCKLTLPVNAAVQTIVGPPSRNSHLAKQLVCLEACKQLHQMGALDDHLTPSIEEPSENACVSKGKDSGAGAGTTKRKELHGTSCIQALCGSWGEKSDDAVFFAYKFDFKCNIITVVYSGFVLLIESKLADDVGNTEMDLFLIGKMVKASVSSCGQVHLNAEQMMKAKRFQEFFFNGLFGKLFVGSKSSGAPREFLLRDKSSSLWSPSRMYLLLPLEDNSTDELRIHWPGITACTLAAEFLNKNSLLGTEQSDDGGSNPSLNSTGSPVTDCKETNIIRFANSSVDANSLRNTVVLAIHTGRIYCIIEAVSDKTAESSFAETVDTVSSEFANFYEYFYKKYNIVLKHPGQPLMLLKQSHNPHNLLVNFNDEGVSAKASQAGVVNEKPRFHVHMPPELLLVLDVPVSVLKSLYLLPSLMHRLESLMLANQLREEINFCSSNIDIPSSMILEALTTLRCCESFSMERLELLGDSVLKYAVSCHLFLRYPNKHEGQLSARRSLAVCNSTLHKLGTDHKIQGYIRDSAFDPRRWVAPGQRVLRPVPCKCGVDSLEVPLDKKFQTEDPKVKVGKSCDRGHRWFCSKTISDCVEALIGVYYLSGGLVAALHVMKWLGIDAEVDPSVVAEVINQASLRTYVPNYKIHMIESKVGYNFSFKFFLQEALTHESVHESYCYQRLEFLGDSVLDLLITQYLYNHHTDIDPGELTDLRSASVNNANFAQVAVRHDLHKHLQHCSTLLSNQISEYVQSFTESDNTTRLDPSIKGPKALGDLVESIVGAILIDTNLNLGKVWRIVEPLLSPIVTPDKLELPPFRELNELCDSLGYFIKEKCINKGEVVHAELLLQLDHDLLVGEGFDRSRKVAKGKAASCLLKHLENRGISRKKRKHDYVDSSQTMDDDSLEPTIPKMQRRAEIQLLDESKKACSATPATPVIVTVKTKKGGPRTTLFELCKKLLWPMPMFRSLTITFLCSAPMEIGEGPERKKGFISFVSKIILNVPGYDIIECTGDAKADKKSSSDSAALFMLYELEQRGKLIIEETL

**>Cotton_A_03190_BGI-A2_v1.0_ID=Cotton_A_03190_BGI-A2_v1.0_ *Gossypium arboreum*| mRNA|length=4941bp_DCL3b**

GAGCAGTGTAACCCTCTAAAAACAACCTTCAGTGAAACACACTTACACCCACCTCAGATTATGGTTTCAATCAATGACGATCATGCTAAGAACCACCACCCTTCTTCATCTTCAAATCCTGATCACTCTAGCCCAAGAGGGCATCAGCTGCAAGTATATGAAGTTGCAAAGAGAAGAAATATCATAGCAGTGCTAGATACAAGTGGAGGGAAGACAATGATAGCTGTAATGCTTATCAAAGATTTTGTTCAACCTATCAACTCCATTGATAAAAAAAAGTTGATTATTTTCTTGGCCCCCACTGTTCATCTTGTTAATCAGCAATTTGAATATATAAAGTTCCATACAAGTTTGGATGTTGAACAATATTATGGAGATAAAGGGGTTAATGAATGGAACTCAGAGTATTGGGAGAAAGACATAAAGGAGCATGATGTTCTTGTTATGACACCCCAAATTCTGTTGGATGCCTTGTGGAAAGCATTCTTGAGTTTAGAGATGGTGTCCTTAATGATAATCGACGAGTGCCACCATGCTACCGGTAACCATCCGTACGTGAAAATAATGAAGGAATTCTATCACAAATTGAACAACAAACCGAAGATTTTCGGGATGACGGCATCGCCCGTAGTTAGCAAAGGTGCATTATCCAACAATGATTATGAGGATCAAATTTCAGAACTTGAAAATGTTATGGATTGCACGATATTCATTATTATGACAGAGATGGAAACATATGTTCCTACAGCAAAAAAAAGTTGTAGGTTTTTCGATCCAACACGATTTTATAGTTCGGGTTTGAAAGCAATGATCGAAGCCTCATGGTTAAAGATGGATGCTTTATTGTCAACGTTGCAAGGCTCGATTCATACTTCTTATACAGATATGGATGATAAGTTTCAGGCCCTCCATAAACGGTCATCCAATGACCATGCTAAGATTCTGCATTGTTTCGACGATCTTGGTCTCATATGTGCTTATGAGGCTGTTAAAGTCTGTCTTGAGAACATCCCTGATACAAAAGAGGAGAATGAAGCACATAGAGTAGGTGTATTGCAGTGTAAAAGGTTCCTCAACGAAGTGCTACAGATAATCGGGGAATCCCTTCCCCTCGGTGATGAAAATTTTCTGAATTCCGGGTTTGACTACTTAAAGGCGGTAGATTCAGGCTATATATCTCCGAAACTGCATGAACTAATTGAACTTTTATTATCACTCGGAGAACCTAGTCAAGTATTGTGCCTCGTTTTTGTTGAAAGAATTGTTACCGCTAAAGTAATCGAAAGATTTGTGAAGAAAGTAAGTTGTTTATCGTGTTTTGTGGTTTCGTGTTTGACTGGAAGTAATACGTCAGTTGATTCCATGGCACCAAAGATCCAAAAGGAAACTTTGGAGTCATGTCAATCCGGGAAGGTGAATCTATTATTTGCTACCGATGTGGTCGACGAGGGAATTCATATACCAAACTGTTCATGTGTGATACGTTTTGACTTGCCGAAAACAGTGTGGAGTTATGTACAATCTCGAGGAAGAGCTAGGCAAAGTGGTTCCCAATTCATCATGATGCTAGAGAGGGGAAATGAGAAACAAAGAGATCAACTATATGATATAATTCGGAGTGAACATTCAGTGACGAATACAGCTATGAATAGAGATCCTGATATATCTCTCCCGGAAAACCGTACATTGGAAGAAGCAAATGTTTCTATCGTGGCTGCTACTGGAGCTTCTGTTACTGAAGCTTCCATTGAAGAAACTTCCAAAAATCGTTGTGCTTCTAAAGGAAAAGATCCCATTTTCGATTCAGGTCTAGGTATATATATAAACTTTCAAAGTCTAAAACTTGTGTTTAACGATAGAATATGGCATGTAATAAGTATTGTTGTCTTGAGTTCAAACCGGAACCATATGGCTCATTACTATGATCGATTTCCGTGTTCACTTGTTATTTCCGCATGTAAAATTGTGTCCAGGTCGCCTTACTTATTGCTTTTGCTTTTCATAATTAGCTTCATATTCCTTTCTTTTGTGTGGTTCAAAGGAACTACAAGACGGAAGGAGCTGCACGGAACAACTCAGATTCGTGCATTATCCGGGAGCTGGGGAGAAAAAGCCGATGCTTCGGTTTTCTTTGCCTATAAAATTGACTTTTCATGCAATGTTGTTAGCGATGTTTACTCCGAATCTGTTCTTCTAATTGAATCGAAGCTTGCTGATGATGTGGGGAACATTGAAGTAGATCTTTACTTGATCGGAAAGATAGCTAAGGCTCGTGTTTCTTTTTATGGGAAAGTACATTTGGATGCTGTACAGATTACGGCAGCAAAGCGTTTTCAGGAAATTTTCTTTAATGGCTTGTTCGGGAGGCTGTTTGTTGGATCATCCGGAACATCAAGGGAACTTTTATTTCATAGTAAAACGAGCTTGTTGTGGCATCCATCGAACATGTATTTGCTTCTGCCCCTAGAGGATAGTTTGAGTAATGAACTGAGAATAAACTGGCCTGGGATAACAGCTTGTACATTTGCTGTAGAGTTTTTAACTGCTGACGGTAACAGAGGTAATCCGTCATTGAACCAAACTGATTCATCCGTGACAGAACACAAGGAGACCAATGTAATCCACTTTGCTAACAGATCTATAGATGTTAACAATATCAGCAACATCGTAGTATTGGCAATTCATACCGGAAGAATCTATTCCACCATTGAATTAGTTCATGATACATCCGCCAAAAGTTCTTTCAATGACATTGCTGATTTGAACTCATCGAAGTTAGCGACCTTCTCCGAATATTACAACAGAAAGTACCGCATCGTGCTGAAACATCCAGGACAACCTTTATTGCTGTTAAAGCAGAGTCATAACCCGCACAACTTGCTGGTTGATTTTAACGATGAAGATTTTTCAGCACAAGCATTACAAGCAAGCTCGGTTAATGAAAAGTCATGGAATAATATCCACATGCCACCTGAGCTTTTGCTCGTCCTTGATGTCCCGATCTATGTTCTAAAAGCCTTTTACTTACTACCATCATTGATGCACCGGCTCGAGTCCTTAATGTTGGCTAGCCAACTTAGGGAAGAGATTGATTTTCATTCAAGCAACTTCGATATACCGAGCTCATTGATCCTGGAAGCGTTAACAACGCTTAGATGTTGTGAAAGCTTTTCGATGGAGAGGCTGGAATTGCTCGGGGATTCAGTTCTGAAGTATGCTGTGAGCTGCCACCTCTTTCTTAGAGATCCCGAGAAACATGAAGGAGAATTATCAATGATACGTTCATTGGCACTGAGTAACTCGACGCTGCATAAGCTGGGAACTGACCGCAAATTACAGGGTTACATTAGAGACAGTGCTTTTGACCCCCGACGTTGGGTAGCTCCAGGACAACTACGTCTTCGGTCTTTTCCTTGTAACTGCAGGGTGGACTCTCGAGAAGTTCCTTTAGATAAAAAGTTTCAGACTGAAGACCTGGAAATTAAGGTCAGTATATCTTGTGATAGAGGCCACCGATGGATGTGTTCGAAAACCATATCAGATTGTGTTGAAGCTCTCATAGGAGCATACTATGTAAGTGGTGGACTCATTGCTGCTCTTCATATGATGAAATGGCTCGGGATCGATGCTGAACTTGATCCCTCTTTGGTAGCTGAAGTCATTAATCGTGCATCCCTACAATCTTATGTCCCTAATGATGAAATCCAGATTATAGAATCGAAAATTGGATACAAATTTTCTATGAAGTTTTTCTTACAGGAGGCCTTGACACATGCATCGGCGAACGAGTTTTACTGTTACCAGAGGCTTGAATTTCTTGGTGATTCCGTGTTGGACTTGCTGATAACCCGATATCTCTACTGCAATCACACCGATATAGATTCAGGAGAATTGACTGACCTCCGATCGGCTTCAGTTAGTAACGAAACTTTTGCTCAAGTTGCTGTCCGTAATGACCTTCATAAGCATCTTCGACATTGTTCACCTTTACTATTGGACCAAATAAGTGAATATGCACGGTCCTTCCCTGAATCTTGGGACACTACACGATCAGTACCCGGCATAAAAGGTCCAAAGGCTCTTGGAGAACTTATTGAAAGTATCGTCACGGCAATCTTGATCGATACAAACCTGAACCTTGACGAAGTTTGGAGAATTGTTGAGCCATTGTTATCTCCGATCGTGACTCCCGATAAGCTCGAGTTGCCTCCACTTCAAGAACTGAATGAATTATGTGACTCTCTCGGGTATTTCATAAGGGAAAAGTGTGTTAATACGGGAGAAATGGTGCATGCCAAGCTTTGGTTACAGCTGGATGATATTTTGTTGGTCGGAGAGGCATTCAATCGAAGCAAAAAGGTAGCAAAAGGAAAAGCAGCTTTATATCTATTGAAGGAACTCGAGGATAGAGGGATCTCCCGAAAGAGAACGAAACAGGGTCATGCCGGTTCGAGTGGAACCACTGATGATGGTTTGTTAGAGCCAAAAATGTGTAAGAAGCAGAAGATAGCTGAGATTCCTTCTCCGACTAACGATTCTTCAAGGACAGCATATGGAGTTGAGATTCCTTCTCCGGCTAACGATTCTTCAAGGACAGCATATGGTTTTAGCTCCGACGCCCTAGTTATTGCAAGGATAAACACGAATAAAGGACAACCGAGGGCCACTCTTTACGAGACTTGTCGGAAACAGTTATGGCCTAGGCCTACATTCGAAACAACAGAAGAAAGATCACGTTCTCCCATGGAAATCGGCAAAGGTGCAGACCGAAAAATCGGGTTTAACAGCTTTGTAACGAAGATCACCTTGAATGTACCGAGTTCAGGCATTATTGAATGCACCGGAGATGTAAGAGCCGATAAAAAGAGCTCTTGGGACTCTGCAGCAATCCTTATGCTTTACAGCCTTAAAGAGCTAGGGAAGCTCATCATCCGCGAATCC

**>Cotton_A_03190_GaDCL3b_ protein sequence length= 1647aa**

MEQCNPLKTTFSETHLHPPQIMVSINDDHAKNHHPSSSSNPDHSSPRGHQLQVYEVAKRRNIIAVLDTSGGKTMIAVMLIKDFVQPINSIDKKKLIIFLAPTVHLVNQQFEYIKFHTSLDVEQYYGDKGVNEWNSEYWEKDIKEHDVLVMTPQILLDALWKAFLSLEMVSLMIIDECHHATGNHPYVKIMKEFYHKLNNKPKIFGMTASPVVSKGALSNNDYEDQISELENVMDCTIFIIMTEMETYVPTAKKSCRFFDPTRFYSSGLKAMIEASWLKMDALLSTLQGSIHTSYTDMDDKFQALHKRSSNDHAKILHCFDDLGLICAYEAVKVCLENIPDTKEENEAHRVGVLQCKRFLNEVLQIIGESLPLGDENFLNSGFDYLKAVDSGYISPKLHELIELLLSLGEPSQVLCLVFVERIVTAKVIERFVKKVSCLSCFVVSCLTGSNTSVDSMAPKIQKETLESCQSGKVNLLFATDVVDEGIHIPNCSCVIRFDLPKTVWSYVQSRGRARQSGSQFIMMLERGNEKQRDQLYDIIRSEHSVTNTAMNRDPDISLPENRTLEEANVSIVAATGASVTEASIEETSKNRCASKGKDPIFDSGLGIYINFQSLKLVFNDRIWHVISIVVLSSNRNHMAHYYDRFPCSLVISACKIVSRSPYLLLLLFIISFIFLSFVWFKGTTRRKELHGTTQIRALSGSWGEKADASVFFAYKIDFSCNVVSDVYSESVLLIESKLADDVGNIEVDLYLIGKIAKARVSFYGKVHLDAVQITAAKRFQEIFFNGLFGRLFVGSSGTSRELLFHSKTSLLWHPSNMYLLLPLEDSLSNELRINWPGITACTFAVEFLTADGNRGNPSLNQTDSSVTEHKETNVIHFANRSIDVNNISNIVVLAIHTGRIYSTIELVHDTSAKSSFNDIADLNSSKLATFSEYYNRKYRIVLKHPGQPLLLLKQSHNPHNLLVDFNDEDFSAQALQASSVNEKSWNNIHMPPELLLVLDVPIYVLKAFYLLPSLMHRLESLMLASQLREEIDFHSSNFDIPSSLILEALTTLRCCESFSMERLELLGDSVLKYAVSCHLFLRDPEKHEGELSMIRSLALSNSTLHKLGTDRKLQGYIRDSAFDPRRWVAPGQLRLRSFPCNCRVDSREVPLDKKFQTEDLEIKVSISCDRGHRWMCSKTISDCVEALIGAYYVSGGLIAALHMMKWLGIDAELDPSLVAEVINRASLQSYVPNDEIQIIESKIGYKFSMKFFLQEALTHASANEFYCYQRLEFLGDSVLDLLITRYLYCNHTDIDSGELTDLRSASVSNETFAQVAVRNDLHKHLRHCSPLLLDQISEYARSFPESWDTTRSVPGIKGPKALGELIESIVTAILIDTNLNLDEVWRIVEPLLSPIVTPDKLELPPLQELNELCDSLGYFIREKCVNTGEMVHAKLWLQLDDILLVGEAFNRSKKVAKGKAALYLLKELEDRGISRKRTKQGHAGSSGTTDDGLLEPKMCKKQKIAEIPSPTNDSSRTAYGVEIPSPANDSSRTAYGFSSDALVIARINTNKGQPRATLYETCRKQLWPRPTFETTEERSRSPMEIGKGADRKIGFNSFVTKITLNVPSSGIIECTGDVRADKKSSWDSAAILMLYSLKELGKLIIRES

**>Cotton_A_01271_BGI-A2_v1.0_ID=Cotton_A_01271_BGI-A2_v1.0_ *Gossypium arboreum*| mRNA|length=4842bp_DCL4**

ATGCCGGACGGCGAATTGTCCACCGACGGGACCGAGCTGTCCATGGCCTCTAAAGTTAAAGCCTTTTCTACTCCGTCGCCGATCGTTGAAATCAGCCGAAAAGATGAACCTATAATGGAAAAGAAAGAGAAGGATCCACGAAAAATTGCTAGAAAGTATCAACTGGAGTTATGCAAGAAAGCCATGGAAGAGAACATAATTGTTTATTTGGAGACTGGTTGTGGGAAGACCCACATTGCAGTTCTGCTTATTTATGAGCTTGGCCATTTGATACGGAAACCTCAGAATAGGGTATGCATTTTTCTTGCTCCCACAGTGGCTCTTGTTCAGCAGCAAGCTCGGGTTATAGAAGATTCTCTTGATTTCAAGGTTGGGACTTATTGTGGTAACTGCAGACACTTGAAGAACCATCATGACTGGGAAATAGAGATCAAAGAATACGAGGTTCTTGTTATGACTCCCCAAATACTACTTCGTAGCTTGTATCACTGCTTCATCAGGATGGACTTAATCGCTCTTCTGATATTTGATGAGTGTCATCATGCTCAAATTAAAAGCAACCATCCTTATGCAGAAATCATGAAAGTTTTTTATGATAAAGCTACTGCATCAATGCTTCCTCGTATATTTGGAATGACAGCGTCTCCCGTTGTTGGTAAAGATGCTTCCAGTCAAGTAAATTTACCTAAAAGCATCAACAGCCTCGAAAACTTGCTTGATGCTAAGGTATATTCAGTTGGAGATAAGGAGGAATTGGAAAGCTTTGTAGCATCTCCTGTGGTTAGAGTATATGATTATGGTCCTGTCAATTTTGGTTCTTCTTGTTCCACCATGATCTATTGTAGTAAACTTGAGGAGATTAAGCGCCAGTGCATACCTCTTGTCAGGAAGAATGGTGATATTCAATCTGCCAGGAACACAAAAAAGCTGCTCAACAGAATGCATGATAATATAATCTTTTGCTTGGAAAATCTGGGCATTTGGGGAGCATTGCAAGCTTGCCGACTTCTTTTAACTGGTAATAACTCTGAGAGGAATGAATTGATAGAAGATGAAGGGATTTTAAGTGACGACTCTGTATGTGATCGATATCTTGTTCATGCTGCTGATGTTTTTGCTTCAGATTGTACGAGAGATGGATCTGCAAACGATTTATCTGATGTAGAGATTTTGAAGGAACCATTTTTCTCGAAAAAGCTGCTACGTCTTGTTGGAATTCTTTCCACCTTCAGGTTACAGCCAAATATGAAATGCATAATTTTTGTGAATAGGATTGTTACTGCAAGATCCTTGTCATACATACTGCAAAACCTTAAGTTCCTATTATTTTGGAAGTGCCATTTTCTTGTGGGAGTCCACTCTGGACTTAAAAGTATGTCAAGGAAGACAATGAAAAACATTCTTGAGAAGTTCAGAACAGGAGAGTTGAATCTTTTGATTGCAACTAAAGTCGGTGAAGAAGGACTTGACATTCAGACATGCTGCCTTGTGATACGTTTTGACCTTCCAGAAACTGTTGCGAGCTTTATCCAATCAAGAGGACGTGCAAGAATGCCTCTCTCTGAGTATGCATTTCTGGTGAACAGTGGGAACGAGAAGGAACTAGATTTGATTAAAAATTTTAAGAAAGATGAAGATCGTATGAATGTGGAAATTTCTTTCAGAACATCTACTGAGGTTTCTATTGGTCTTGAGGAGAGAATCTATACGGTTGATTCATCTGGTGCTTCCATCAGTTCAGGATATAGTATCTCTCTACTTCATCATTACTGCTCAAAACTGCCCCATGATGAGTACTTTTTCCCCAAGCCAAGCTTTTATTATTTTGAAGATTCAGGAGGAACAATCTGCAATATAATATTACCTTCTAATGCTCCAATAAATCAAATTGCCAGTACACCTCAATCTTCAGTTAATGCTGCCAAAAAGGATGCTTGTCTAAAAGCAATTGAAGAATTGCATAAATTGGGAGCCTTGACTGACCATCTCTTGCCACTGCAAAACAGTGTTCTTGAGGAGGAAACATTGCTGGTGTCTTCTGATTCTGGGAGCTCTGAAGCAGATGAGGATTCACGAGGTGAACTACATGAAATGCTTGTTCCTGCTGTGCTTAAAGAATCATGGACTAACTTAGAGAACTGTGTCCTCCTTTATGCTTACTATATAAAATTTATTCCGAATCCTGAGGACAGGAGCTATAAAGAGTTTGCTCTCTTTGTCAAGTCTCCTCTCCCTAAGGAGGCTGAGAGAATGGAGCTGGATCTTCACCTATCTCGCCGTAGGTCGGTGATGACAAAGCTTACCCCATCAGGAGTTGCAGAATTTAAGAGAGAAGAGATCATGCAGGCACAGCATTTTCAAGAAATGTTCTTCAAGGTCATCCTTGATAGATCAGAACTCCTTTCTGAATTTGTTACTTTAGGAAATGTCTTTCTTTCATCAAGCTCCTCAACATTCTATTTACTGCTTCCTGTTATTCTCTCCAATTGTGAGAATAAAGTGACAGTGGATTGGGGGATTGTGCAAAGATGTTTGTCGTCTCCACTTTTTAAGCCTCCTGTTGCAGCTGCAAAGATTGAAAATTTCCCTTCAGATGTTTGCTTGCACCTTGTAAATGGTTGTAGGAGTATAAGAGACATTGAGAATAGTTTGGTGTCTGGCATTCATCTTAAGTACCCTGAACAACCTCTTCTGCATGCAAAACCTCTTTTTAAGTTGCACAATTTACTCCACAACCGAAAGCCGGAAGATTCAGAATCAAATGAACTGGAAGAATACTTCATTGATTTGCCTCCTGAGCTTTGTCAGCTGAAAATAATAGGCTTCTCCAAAGAGATAGGGAGTTCGCTTTCTTTGTTACCATCAATTATGCATCGCCTGGAGAACTTGCTTGTGGCTATTGAACTGAAGCATGTTTTTTCTGCTTCATTTGCCGAGGGAGCTGAAGTTACAGCCCTTAGGGTTCTAGAAGCTCTTACTACAGAGAAGTGTCAAGAGCGTTTTTCTCTTGAAAGGCTTGAATCACTTGGTGATTCCTTCCTCAAGTTTGCTGTGGCCCGCCATCTTTTTCTTTTACATGATGCACTCGATGAAGGGGAATTGACTAGGAGAAGGTCAAATGTTGTAAATAATTCTAATTTATTCAAGCTGGCAACAAGGCGAAATTTACAGGTTTACATACGTGATCAACCTTTTGATCCTTATCAGTTCTTTCCCTTGGGCCATCCCTGCCCAGTAATTTGTACAAAAGAAACTAAAGGAACTGTTCATCCACAATCTAGTTGTCAAGTAGATCATGCAAAGAGTGAAGTCAGATGTAGTAGAAATCACCATTGGCTACAAAAGAAAACAATTTCTGATGTGGTTGAGGCTCTTGTAGGAGCATTTATAGTTGATAGGGGCTTCCAAGCGGCAACAGCATTTCTTAGATGGATAGGCATACGAGTGGACTTCCAAGGTTCTCAACTTAATAGTATTTGCGCTGCAAGCAAGAGATTTATGCCACTTTCCTCCCTATTGGATATTGGAGATCTTGAAAATTTATTGGGTTATCAGTTTCTTCATAAGGGTCTCCTCCTTCAGGCGATTGTGCATCCTTCTTTTAATAGGCATGGTGGAGGCTGCTACCAGAGATTGGAGTTTCTTGGAGATGCTGTCTTGGATTATTTAATTACATCATATCTGTTTTCACTGTATCCAAAGTTGAAACCTGGTCAGTTGACTGATTTGAGATCAGTGTCAGTGAACAACAAGTCCTTTGCCAATGTTGCAGTGGACAGACGCTTACACAAATTTCTTATGTGTGACTCTTGTCACCTCAACGAGGCCATAGAAAAATATGTTGACTTCATCACATCATCACCAGACAGGGGATTATTTGAAGGGCCAAAATGTCCGAAGGCTCTTGGTGACTTGGTAGAGTCTTGTTTTGGTGCTATTCTCCTTGATACAGGGTTCAACTTGAACCGTGTTTGGAAGATAATGTTATCCATTCTGGATCCAATCAAGAGCCTTTCAAGTGTGCAGCTCAATCCTATCAGGGAAGTACAGGAACTTTCCCAGTGTTATAACTGGGATTTGCAGTTTCTAGTTGCAAAAGTTGGTAGAAATTTTTCAGTGGATGCAAAGGTAAATGCAGGAGATGTTCCTCTATGTGTTTGTTCCAGCAACATCAATAGAAAAGAAGCTATCAGAACTACTGCACATCAACTATATGTAAAGTTAAAGGCTCTAGGATATGCACCCAAATCAAAGTCCTTGGACGAAGTTTTGAAGGGAAGCCCCAAGAATGAAGCCAAGCTTATTGGATACGATGAAACACCCATTGATGTCACTGTTCCTGACATAATTGGATTTGAAAACATGAAATTGCAAGAATCTCTTGTGAATGATTTCAACCCCAAAACCCGTTCTAGTAAAAAGACAACTTCTAGCGGTGTATCTTGCATCTCGCCTGGTAGCAGACCTTTTGAAGTAAAAGCAGCAAGGGGTTCAGCCTCTGGTATTGAACCCAAAGGCACACCCCCCAATTGCTCCATTGTGGATCCTAGTTGTGGGATTGACTCGCCATCCAAAGGTGAATCACATAGTCGTACTGCTAGATCACAGTTATACGAAATCTGTACTATCAATTGCTGGAAACCCCCTTTGTTTGAATGTTGCAAAGAGGAAGGACCAAGTCATTTGAGATCATTCACTTACAGGGTAATAGTTGAAATCGAAGAAGCTCCAGATATGATATTGGAGTGCTTCAGCAGTCCTCGGACCACAAAGAAGGCAGCAGCCGAGCATGCAGCGGAAGGTGCGCTCTGGTATTTAAAACATGAAGGATACTTACAATAA

**>Cotton_A_01271_GaDCL4_ protein sequence length= 1613aa**

MPDGELSTDGTELSMASKVKAFSTPSPIVEISRKDEPIMEKKEKDPRKIARKYQLELCKKAMEENIIVYLETGCGKTHIAVLLIYELGHLIRKPQNRVCIFLAPTVALVQQQARVIEDSLDFKVGTYCGNCRHLKNHHDWEIEIKEYEVLVMTPQILLRSLYHCFIRMDLIALLIFDECHHAQIKSNHPYAEIMKVFYDKATASMLPRIFGMTASPVVGKDASSQVNLPKSINSLENLLDAKVYSVGDKEELESFVASPVVRVYDYGPVNFGSSCSTMIYCSKLEEIKRQCIPLVRKNGDIQSARNTKKLLNRMHDNIIFCLENLGIWGALQACRLLLTGNNSERNELIEDEGILSDDSVCDRYLVHAADVFASDCTRDGSANDLSDVEILKEPFFSKKLLRLVGILSTFRLQPNMKCIIFVNRIVTARSLSYILQNLKFLLFWKCHFLVGVHSGLKSMSRKTMKNILEKFRTGELNLLIATKVGEEGLDIQTCCLVIRFDLPETVASFIQSRGRARMPLSEYAFLVNSGNEKELDLIKNFKKDEDRMNVEISFRTSTEVSIGLEERIYTVDSSGASISSGYSISLLHHYCSKLPHDEYFFPKPSFYYFEDSGGTICNIILPSNAPINQIASTPQSSVNAAKKDACLKAIEELHKLGALTDHLLPLQNSVLEEETLLVSSDSGSSEADEDSRGELHEMLVPAVLKESWTNLENCVLLYAYYIKFIPNPEDRSYKEFALFVKSPLPKEAERMELDLHLSRRRSVMTKLTPSGVAEFKREEIMQAQHFQEMFFKVILDRSELLSEFVTLGNVFLSSSSSTFYLLLPVILSNCENKVTVDWGIVQRCLSSPLFKPPVAAAKIENFPSDVCLHLVNGCRSIRDIENSLVSGIHLKYPEQPLLHAKPLFKLHNLLHNRKPEDSESNELEEYFIDLPPELCQLKIIGFSKEIGSSLSLLPSIMHRLENLLVAIELKHVFSASFAEGAEVTALRVLEALTTEKCQERFSLERLESLGDSFLKFAVARHLFLLHDALDEGELTRRRSNVVNNSNLFKLATRRNLQVYIRDQPFDPYQFFPLGHPCPVICTKETKGTVHPQSSCQVDHAKSEVRCSRNHHWLQKKTISDVVEALVGAFIVDRGFQAATAFLRWIGIRVDFQGSQLNSICAASKRFMPLSSLLDIGDLENLLGYQFLHKGLLLQAIVHPSFNRHGGGCYQRLEFLGDAVLDYLITSYLFSLYPKLKPGQLTDLRSVSVNNKSFANVAVDRRLHKFLMCDSCHLNEAIEKYVDFITSSPDRGLFEGPKCPKALGDLVESCFGAILLDTGFNLNRVWKIMLSILDPIKSLSSVQLNPIREVQELSQCYNWDLQFLVAKVGRNFSVDAKVNAGDVPLCVCSSNINRKEAIRTTAHQLYVKLKALGYAPKSKSLDEVLKGSPKNEAKLIGYDETPIDVTVPDIIGFENMKLQESLVNDFNPKTRSSKKTTSSGVSCISPGSRPFEVKAARGSASGIEPKGTPPNCSIVDPSCGIDSPSKGESHSRTARSQLYEICTINCWKPPLFECCKEEGPSHLRSFTYRVIVEIEEAPDMILECFSSPRTTKKAAAEHAAEGALWYLKHEGYLQ

***Gossypium raimondii***

**>Gorai.001G150400.1-JGI_221_v2.1_ID=Gorai.001G150400.1-JGI_221_v2.1_*Gossypium raimondii*| CDS|length=5853bp_DCL1**

ATGGAGGAAGAAGGTAGGGTTTCTGGTGGCAATGGGTCATCTTACTGGCTGGATGCTTGTGAGGACATATCATGCGACTTGATTAGCGATTTTGTTGATTTCGATGCTCCTATAGTTCAAGACTCCGTTGACAATACTTCCAATCAAGATTTCTTTGGAGGAATTGATCACATTCTCGATAGCTTCAAGAACGGCGTGGGACTTCCTCCGGTGGGAAGTAACAGTGATAGCTCTGTCGTCAATGGAAATGGAATTCACGACCCCGTTGCTGGAGATGGATGGTCTCCCAATGAGCTCTCTGGGGTTTCCAAGGATTTACCTGACAACTCAGTTCCGCCATCTAATGGAGTTGAAAAAAAGATTGGAAGCAAAGGGCAGGAAAAAAGCTGCGATGATAGTAATTCCAGTTTATTTGATTATTCTAATAAGGATAATGGAGTTCATCGAGACGATAAAAGGTCATCCGAGTCGAGAGATAGGGGTTTAGATAGTGAAGAGAGGTGTAACAAGAGGGCTCGTGCGAATGGCTGCAAGAGTGATAGGCAGTATTCTAGTAGAGGTCAATATTATCCCCGGGATAGGGAGAGATGTTTTGCTAGGAAAAGGGTTCGAGATTGGGATGAAATTGATCGGAGAGATAGAGAGCATGTTAGGAGAAGAGAACATTATTATGGTGGCAATAGGAGGGATGGGAGAGAGAGAGAACCAAGGGGTTATTGGGAGAGGGAACGGTCAGGGTCCAATGAGATGGTTTTTAGGCTGGGTACTTGGGAAGCAGATCGGCAAAGAGAAGGGAAGGTGGCTAATGACAAAACTCCCGAGTGCAATGGAAAGATGGAGAAGAAAGTTGAGCAGCCGAAGGAAAAACTCTTGGAGGAGCAAGCTCGTCAATATCAATTGGATGTTCTTGAGCAGGCAAAAAGGAAAAACACAATTGCATTCCTTGAAACCGGGGCAGGGAAGACCCTCATTGCTGTTCTCCTCATGAAAAGTATTTCTGATGATTTACAAAAGCACAACAGAAAAATGCTCTCTGTCTTTTTGGTTCCTAAAGTTCCACTAGTTTATCAGCAAGCTGAAGTTATTCGCGAGAGAACTGGTTTCCAAGTCGGTCATTATTGTGGGGAGATGGGTCAAGATTTTTGGGATGCTCGTAGGTGGCTGCGCGAGTTTGAATCAAAACAGGTTTTGGTTATGACAGCTCAAATTCTGCTGAATATTTTGAGACACAGCATAATTAAAATGGAATCAATCAATCTCCTTATCCTGGACGAGTGTCATCATGCTGTGAAGAAACATCCGTATTCACTGGTTATGTCTGAATTCTATCATACAACACCAAAGGAGAAGAGACCTTCTGTTTTTGGAATGACCGCTTCTCCTGTTAACTTGAAGGGTGTTTCAAGTCAAGTTGATTGTGCTATAAAAATTCGTAATCTAGAAAGCAAACTGGATTCTGTAGTCTGTACCATCAAAGACCGCAAGGAACTCGAGAAACATGTGCCAATGCCTTCAGAAGTTGTGGTAGAGTATGACAAAGCAGCCAGTTTATGGTCCCTCCATGAACAAATAAAACAAATGGAAGCAACAGTTGAAGAAGCTGCACAGTCAAGCTCTAGAAGAAGTAAATGGCAGTTCATGGGAGCTAGAGATGCAGGAGCCAAGGAAGAGCTTCACCAAGTTTATGGTGTATCTGAAAGAACAGAAAGTGATGGAGCTGCTAATTTGATACAAAAGTTGAGGGCTGTTAATTATGCACTGGGTGAACTGGGCCAATGGTGTGCTTATAAGGTTGCACAATCTTTTCTGACAGCTTTGCAAAATGATGAGAGGGCAAACTACCAGCTTGATGTCAAGTTTCAAGAATCTTACCTAAACAAAGTTGTTTCCCTCTTACAATGCCAATTATCAGAGGGAGCTGTTACTGAAAAAGACATGAACAATGCAGAAGCAGAGAACTGTAATGCTCAAGATGGGACCAATACTGATGAGATTGAGGAAGGAGAGCTCCCTGACAGTCATGTTGTCTCTGGTGGAGAGCATGTGGATGTGATCATTGGAGCGGCTGTAGCAGATGGAAAAGTGACCCCGAAAGTACAGTCATTGATTAAAATACTTCTGAAGTATCAGCACACAGAGGATTTTCGAGCAATCATCTTTGTTGAGCGAGTTGTGGCTGCTTTAGTTCTTCCTAAGGTTTTTGCAGAGCTTCCGTCTCTGAGTTTCATCAGGTGTGCAAGTTTAATTGGGCACAACAATAGTCAAGAAATGCGGACCGGACAAATGCAGGATACAATTGCTAAATTCCGTGATGGTCGTGTGACATTGTTAGTTGCAACTAGTGTTGCTGAGGAAGGATTGGATATTCGGCAATGCAATGTTGTCATTCGTTTTGATCTTGCAAAAACTGTTTTGGCATACATTCAGTCTAGAGGTCGTGCAAGGAAGCCTGGGTCAGATTACATCTTGATGGTTGAGAGAGGAAATTTATCGCATGCTACATTCCTAAGGAATGCGAGGAATAGTGAGGAGACCTTGCGGAAAGAAGCAATTGAGAGAACCGACCTTAGTCATTTGAAGGATACTTCGAGGTTGATTTCGGTGGATATGGTACCAGGTACTGTGTACCAGGTTGAATCAACTGGTGCCATTGTTAGCTTGAATTCTGCTGTTGGACTAGTCCATTTTTACTGCTCTCAGCTTCCTAGTGACAGATATTCAATACTTCGTCCTGAGTTTATTATGAAGAAGCATGAGAAGCCAGGGGGTCCAACTGAATATTCTTGCAAGCTTCAGCTCCCCTGTAATGCACCATTTGAAGAGCTCGAGGGTCCCATGTGCAGTTCTATGCGTCTTGCCCAGCAGGCTGTATGTTTGGCTGCTTGCAAGAAGCTCCATGAGATGGGAGCATTTACTGATATGCTCTTGCCCGACAAAGGAAGCGGGGAAGAAGCAGAGAAGGTTGACCAGAATGATGAAGGAGACCCACTTCCTGGAACTGCTAGGCATAGAGAATTCTATCCAGAAGGTGTAGCAGATATACTCCAGGGAGAATGGATTTTATCTGGAAGAGATGGTGTTGATGACTCCAAAATACATCGTCTGTACATGTATACTATCAAATGTGTAAATAATGGCTCTTCAAAAGATCCATTCTTAACTAAAGTCTCAGATTTTGCAGTACTTTTTGGCAAAGAGCTGGATGCAGAGGTGTTATCGATGTCGGTGGATCTATTTATCGTTCGAGCCATGATAACAAAGGCATCTCTTGTCTTCAGGGGATCAATAGATATAACTGAAAGTCAGATGGCATCCCTTAAAAATTTTCACGTAAGAATGATGAGCATTGTATTGGATGTGGATGTTGATCCTGCCACTACTCCTTGGGATCCTGCCAAGGCATATTTGTTTGTCCCTGTGGTTGGCGATAAGTTTGTAGATCCTATAAAAGAAGTTGATTGGGATTTGGTAGATAATATAATCACTACAAATGCGTGGAGCAATCCCCTTCAGAGAGCTAGGCCAGATGTTTTCCTTGGGACAAATGAGAGGACACTTGGTGGTGACAGAAGGGAGTACGGATTTGGGAAATTGCGTCATGGCCTGGCTTTTGGGCACAAACCTCATCCTACTTATGGTATCAGAGGAGCCGTAGCCCCATTTGATGTTGTTAAAGCTACCGGGGTGGTTCCTAGTCGTGATACGATCGAGGTACAAGGGGATTGGACCAAAGGCAAATTGATAATGGCTGATGGTGTTGCACGTGCAGAAGATCTTGTTGGAAGAATAATAACAGCCGCTCATTCGGGGAAGAGGTTTTATGTAGATACAATATGCTATGACATGTCAGCAGAGACCTCCTTTCCGAGGAAAGAGGGCTATCTTGGTCCTGTCGAGTACAGCTCATATGCCGATTACTATAAGCTGAAGTATGGTGTTGAGTTGAGCTGCAAGCAACAAGCTTTGATAAGAGGTCGTGGTGTTTCATACTGCAAGAATCTCTTATCTCCTCGATTTGAGCACTCAGAAGGTGAATCGGAGGAGGCACTTGACAAAACATACTACGTGTTTCTTCCGCCTGAGCTATGTTTTGTTCATCCACTTCCTGGATCACTTGTTAGAGGTGCCCAGAGGTTGCCCTCTATTATGAGGAGGGTTGAGAGCATGCTTCTTGCAATTCAACTTAAGCACATAATACAATTTCCGGTCCCTGCTTCAAAGATTTTGGAAGCTTTGACTGCTGCTTCTTGTCAGGAGACATTTTGCTATGAAAGGGCTGAGCTTCTTGGGGATGCTTACTTGAAATGGGTAGTCAGTCGTTTTCTGTTTCTTAAATATCCCCAGAAACATGAAGGTCAACTGACCAGGATGAGACAACAAATGGTGAGTAACATGGTATTGTATCAATATGCATTAAATAAGGGACTTCAATCATACATCCAAGCAGATCGCTTTGCACCATCTAGATGGGCTGCTCCTGGGGTGTTGCCAGTCTTTGACGAGGATACAAAAGATGGTGACACGTCCTTATTTGATCAAGAACATGCAACTGCTGATGTTTTACCAGTAAAAGTACATGGTAATGGGTTTGAAGATGAAGATATGGAAGATGGTGAGATTGAGAGTGACTCAAGTTCTTATAGAGTCCTCTCTAGCAAGACCTTAGCAGATGTGGTTGAAGCACTGATTGGAGTTTATTATGTTGAAGGTGGCAAGCATGCAGCTAACCACCTCATGAAATGGATTGGGATCCAGGTGGAGTCTGATCCTGATGAGATGGACTCTATAGTGAAGCCATCTAATGTTCCAGAAAGCATACTCAGGAGTGTAAATTTTGAGGCCTTAGAAGGTGCATTGAAAATCGAGTTTAAAAATAGGGCCTTGTTGGTGGAAGCTATTACTCATGCTTCACGCCCATCATCGGGAGTATCCTGCTACCAGCGTTTGGAGTTTGTTGGTGATGCAGTCTTGGATCATCTTATCACAAGACATTTGTTTTTTACATACACCAATTTGCCTCCAGGTCGCTTGACTGATTTGCGTGCTGCTGCCGTAAACAATGAAAACTTTGCACGTGTTGCAGTAAAGCACCTGTTGCATGTGCACCTTCGGCATGGATCAAGTGCCCTTGAGAAACAGATTCGGGACTTCGTGAAGGAAGTTCAGGATGAGTTATTAAAGCCGGGTTTCAACTCTTTTGGATTGGGAGATTGCAAAGCACCAAAAGTTCTTGGAGATATTGTTGAATCCATTGCTGGTGCCATTTTTCTCGACAGTGGACGTGACACTGCAGTTGTCTGGAGGGTTTTTCAACCTCTGTTGCATCCCATGGTGACTCCAGAGACATTGCCGATGCATCCTGTCCGGGAACTACAAGAACGATGCCAACAACAAGCTGAAGGCTTGGAATACAAAGCCAGTCGAAGTGGCAATTTGGCCACTGTGGAGGTTTTCATTGATGGGGTCCAGGTTGGAGTTGCTCAGAATCCCCAAAAGAAGATGGCACAGAAACTAGCTGCAAGGAATGCACTTGCTGTTCTGAAGGAGAAAGAAACAGCTGAAGCTAAGGAGAAATGTGAGGAGAATGGGAAAAAGAAGAATGGCAACCAGACATTTACCAGACAAACATTGAATGATATCTGCCTGCGTCGAAACTGGCCTATGCCCTTCTATTGGTGTGTGAATGAAGGGGGCCCTGCCCATGCAAAGAGATTTACTTTTGCTGTCAAGGTTAACACCACTGACAGGGGGTGGACCGATGAATGCATAGGTGAGCCAATGCCTAGTGTTAAGAAGGCCAAGGACTCAGCCGCCGTGCTTCTCTTGGAACTTTTAAACAAATGGTATTCATGA

**>Gorai.001G150400.1_GrDCL1_ protein sequence length= 1950aa**

MEEEGRVSGGNGSSYWLDACEDISCDLISDFVDFDAPIVQDSVDNTSNQDFFGGIDHILDSFKNGVGLPPVGSNSDSSVVNGNGIHDPVAGDGWSPNELSGVSKDLPDNSVPPSNGVEKKIGSKGQEKSCDDSNSSLFDYSNKDNGVHRDDKRSSESRDRGLDSEERCNKRARANGCKSDRQYSSRGQYYPRDRERCFARKRVRDWDEIDRRDREHVRRREHYYGGNRRDGREREPRGYWERERSGSNEMVFRLGTWEADRQREGKVANDKTPECNGKMEKKVEQPKEKLLEEQARQYQLDVLEQAKRKNTIAFLETGAGKTLIAVLLMKSISDDLQKHNRKMLSVFLVPKVPLVYQQAEVIRERTGFQVGHYCGEMGQDFWDARRWLREFESKQVLVMTAQILLNILRHSIIKMESINLLILDECHHAVKKHPYSLVMSEFYHTTPKEKRPSVFGMTASPVNLKGVSSQVDCAIKIRNLESKLDSVVCTIKDRKELEKHVPMPSEVVVEYDKAASLWSLHEQIKQMEATVEEAAQSSSRRSKWQFMGARDAGAKEELHQVYGVSERTESDGAANLIQKLRAVNYALGELGQWCAYKVAQSFLTALQNDERANYQLDVKFQESYLNKVVSLLQCQLSEGAVTEKDMNNAEAENCNAQDGTNTDEIEEGELPDSHVVSGGEHVDVIIGAAVADGKVTPKVQSLIKILLKYQHTEDFRAIIFVERVVAALVLPKVFAELPSLSFIRCASLIGHNNSQEMRTGQMQDTIAKFRDGRVTLLVATSVAEEGLDIRQCNVVIRFDLAKTVLAYIQSRGRARKPGSDYILMVERGNLSHATFLRNARNSEETLRKEAIERTDLSHLKDTSRLISVDMVPGTVYQVESTGAIVSLNSAVGLVHFYCSQLPSDRYSILRPEFIMKKHEKPGGPTEYSCKLQLPCNAPFEELEGPMCSSMRLAQQAVCLAACKKLHEMGAFTDMLLPDKGSGEEAEKVDQNDEGDPLPGTARHREFYPEGVADILQGEWILSGRDGVDDSKIHRLYMYTIKCVNNGSSKDPFLTKVSDFAVLFGKELDAEVLSMSVDLFIVRAMITKASLVFRGSIDITESQMASLKNFHVRMMSIVLDVDVDPATTPWDPAKAYLFVPVVGDKFVDPIKEVDWDLVDNIITTNAWSNPLQRARPDVFLGTNERTLGGDRREYGFGKLRHGLAFGHKPHPTYGIRGAVAPFDVVKATGVVPSRDTIEVQGDWTKGKLIMADGVARAEDLVGRIITAAHSGKRFYVDTICYDMSAETSFPRKEGYLGPVEYSSYADYYKLKYGVELSCKQQALIRGRGVSYCKNLLSPRFEHSEGESEEALDKTYYVFLPPELCFVHPLPGSLVRGAQRLPSIMRRVESMLLAIQLKHIIQFPVPASKILEALTAASCQETFCYERAELLGDAYLKWVVSRFLFLKYPQKHEGQLTRMRQQMVSNMVLYQYALNKGLQSYIQADRFAPSRWAAPGVLPVFDEDTKDGDTSLFDQEHATADVLPVKVHGNGFEDEDMEDGEIESDSSSYRVLSSKTLADVVEALIGVYYVEGGKHAANHLMKWIGIQVESDPDEMDSIVKPSNVPESILRSVNFEALEGALKIEFKNRALLVEAITHASRPSSGVSCYQRLEFVGDAVLDHLITRHLFFTYTNLPPGRLTDLRAAAVNNENFARVAVKHLLHVHLRHGSSALEKQIRDFVKEVQDELLKPGFNSFGLGDCKAPKVLGDIVESIAGAIFLDSGRDTAVVWRVFQPLLHPMVTPETLPMHPVRELQERCQQQAEGLEYKASRSGNLATVEVFIDGVQVGVAQNPQKKMAQKLAARNALAVLKEKETAEAKEKCEENGKKKNGNQTFTRQTLNDICLRRNWPMPFYW

**>Gorai.009G374900.1-JGI_221_v2.1_ID=Gorai.009G374900.1-JGI_221_v2.1_*Gossypium raimondii*| CDS|length=4188bp_DCL2a**

ATGGAGCCAGTTGACATGGAGAAGGACATTTCTCAACTACACCCTGCTGATCCTCTCCCTTTTGCCAGAAGCTATCAGCTTGAAGCATTAGAGAAAGCTATCAAGCAAAACACAATAACTTACTTGGAAACTGGATCTGGCAAGACAATGATCGCCATCATGCTTCTCCGCAGCTATGCCCATCTTATTCGCAAGCCCTCACCTTTTTTTGCTGTCTTCTTGGTTCCCAAAGTTGTCCTGGTTAAACAACAAGCTGATGCTGTGGAAATGCATACGGACTTGAATGTTGGAAAGTATTGGGGAGATATGCAGGTCGACTTTTGGGACGGGGAGAAGTGGAAGCAAGAACTAGATAAATATGAGGTGCTTGTGATGACACCTCAGATTTTACTTGATGGATTGAGGCATAGCTTCTTCAAGATAAACATGATAAAGGTTTTGATAATTGATGAATGCCACCATGCCCGAGGAAATCACCCTTATGCCTCTATTATGAGAGAATTCTATCATCGCCATTTAGAAGCTGGTGCATCTAATCTTCCTAGGATTTTTGGGATGACTGCTTCTCCTATAAATTCAAAAGGGGCAAACTCTGCTGATAGCTATTGGCAGAAGATCCATGAATTGGAGACGATTATGAACTCAAAGGTGTATACATGTGAAAGTGAATCAGTGCTTGCTCAGTTTGTTCCATTTTCTACTCCAAAGTTCAAGTTTTACCAACATATGGAAATTCCAAATGTTCTATATGCACACTTGGTAGAGGAATTGACTGTTTTGAAAGTAAAGCATGAATGTTCGTTGGATAATTTGGATCTTGAAGCTTCTGCAGCAGAATCTACAAGAAAGAAACTATCAAAGATACATTCAGCTTTAATACATTGTCTACATGAGCTTGGTGTTTGGTTGGCTTTAAAGGCTGCAGAGTGCTTTTCATGTTATGAAAGTGAGCATCTTATGTGGGGGAATTTGGATGTCTTTGGCGAGAAAATTATTAGGAGCTACAGTGTGGATGCTTTTCATGCAATTGAAACATGCATGCCATCTGGTCTGGATTGGACCATAGCTAATGATGTTAAAGGCAGTGTGGCTGCTGGGTTTCTTACCACAAAAGTTTTATGCCTTATTGAATCTCTATTTGAATACAGGGTGTTGAAGGACATAAGATGTATAATTTTTGTTGAGAGGGTTATAACAGCAGTTGTGCTTCAATCGCTATTTAGTGAATTACTTCCCAGGTACAGTAATTGGAAGACTAATTACATTGCGGGAAATAACTCTGGATTGCAGAATCAGACAAGGAAAAAACAAAACGAAATTGTAGAAGAATTCCGCAAAGGCATGGTTAACATAATCGTTGCAACTTCAATTCTTGAAGAAGGCTTGGATGTTCAATCATGCAACCTAATTATCAGATTTGATCCTTCACCAACAATTTGTAGTTTCATACAGTCTCGAGGACGTGCTAGAATGCAGAACTCAGATTATCTATTAATGCTGAAGAGTGGGGACTTTTCTACACATTCTCGACTGAAGAACTATCTTACTAGTGGAGATGTAATGAGAAAGGAATCTTTACGCCATGCATCCAATCCTTGTTCTCCTCTTAGTAAGGGCTTAGATGATGAAGAGTTTTACCAAGTCGCGAGTACAGGGGCATGTATGACTCTTAGTTCTAGCGTTGGTCTAATGTACTTCTATTGCTCACGTCTCCCTGCAGATGGGTATTTTAAACCTATTCCGAGGTGTGTTATTGACAAGCAAATGGGGCTTTGCACCCTCCTCCTACCCAAGAGTTGCCCTATACAAACTGTTTGTGTTCAGGGTAATATAAAAAACCTAAAGAAAATAGCATGCTTTGAAGCATGCAAGAAACTCCATCAAATTGGTGCTTTAACAGACAATCTTGTTCCTGATATTGTTTTTGAAGAAAATGATGTGGAAGAATTTGAAAAGGAGCCCTATAATGATGATCAACCTATTTTCTTTCCACCTGAACTAGTGAATAAGGGTTCACTGGACTCCATGACAAAGTACTACTGCTATTTAATGGAGTTGAAGCAGAACTTTGATTATGAGGTTCCTGTTCATAACATCATGCTTCTTGTCAGGAATCAGTTTGACATGGATGAAAAAAGTGTGAATATTGAGTTAGAAGTTGACAGGGGCACATTAACAGTTAACATGAAATATATTGGACTAATACGTCTTAATTCTGACCAGGTTATCTTATGTAGAAGGTTTCAGCTTGCAGTTTTTCAAGTACTTATGGATCGTAAAGCCGAAAAGTTTGCAGAGGTGTTATGTGACCATACCTTTGGGAATAATTCCGAAATTGATTATCTACTCCTCCCATCAAATTATGTGGGCCAGAGTCCTCTGATTGATTGGTTGTCAGTTACTTCTGTCACATTTTCTTATGAAAAGGCCTGGAAGAATCATGTGAACTGCAATGCTGGTATGATACAGACCAAAAGCGGTCTGGTGTGCACTTGCATGGTCCAAAATTCGTTGGTCTCTACTCCTCATAATGGTCATGCTTATATTATCAGTGGTCTTTTAACTAATATAAATGCAAATTCACTTTTGAGGTTGAGTGATGGACGCTTAATGACTTACAAGGAGTATTATGAACAACGCCACGGTATCAATTTCTGTTATGGTCAAGTCTCTTTCCTTGCAGGGCGACATATTTTCCCCGTGCAAAATCACATTCAAAGGTTCAGAAAACAAAAAGAGAAAGAATCAAGTAATGCATTGGTAGAATTGCCTCCTGAGCTTTGTTGTGTAGTAATGTCTCCCATATCGGTTAGCACATTTTATTCATTCACATTTCTTCCGTCAATCATGCATCGACTCGAGTCTTTGCTCCTTGCTACCAGCTTGAAAAAGATGCATTTGGATCATTGCGTGCAAAATATTGCGATTCCAACCATGAAGGTTTTGGAGGCAATTACAACCAAAAAGTGCCTAGAAAACTTTCATTTGGAATCACTAGAGACTCTTGGTGACTCTTTTTTGAAGTATGCTGTTTGTCAACAGCTTTTCAAAAAATATCAAAATCATCATGAGGGCCTTCTTAGTATTAGGAAGGACAAAATTATTTCAAATACAGCTCTGTCCATGCTAGGATGTGACAAGAAACTTCCGGGGTTTATCCGTGATGAGCCTTTTGATCCCAAAGATTGGATGATTCCTGGTTATAATTGTGGAAATTACTCATTAAATGAGGAAACACTGTGTAATGCAAAAAAAATATATGTTAGAGGGAGAAGAAAGGTAAAGTGTAAGAAGGTTGCTGATGTTGTTGAGGCACTTATTGGCGCATACCTTAGCACGGGAGGCGAAGCAGCTGGATTACTATTTTTGGATTGGATCGGTATAAGTATTGATTTCACAAATATACCATATGAAAGACATTTTAAAGTGCGGGCCGAGAAGTTTGTTAATGTCCAGCACTTTGAGTCCCTTCTACACTATTCATTCCAAGACCCTTCTTTGTTGGTGGAAGCATTAACCCATGGTTCTTATATGCTTGCTGAAATTCCAGGTTGTTATCAGCGGCTGGAATTTCTAGGGGACTCCGTTTTAGATTATCTTATTACTCTGCATTTATATAATAAATATCCTGGGATCACGCCAGGATTATTAACGGATCTTAGGTCAGCATCTGTTAACAACAATTGCTACGCATTATCAGCTGTAAAGGCTGGATTTCATAAGCACATTCTCCAAAGTTCACAGAAGCTTTACAAGGATATAAAAGAAACCGTTGAAAGTTTTCAGGAATTATCGTTGGAATACACTTTCGGCTGGGAATCTGAAAAATCTTTCCCCAAGGTACTCGGTGACGTGATGGAGTCACTTGCTGGAGCTATTTTTGTTGATTCAGGATACAAGAAGGAAATCGTATTCCAGAGTATAAGGCCACTGTTGGAGCCCATGATTACTCCAGAGACTATGACGGTTCACCCTGTCAAAGAGCTATATGAGCTCTGTCAAAAGGAGCATTATGAACTAAGAAAACCCATTGTTTCACATGAGGACGGCATTTCTTCTATTACGATAGAGGTTGAAGCAAATGGGAAGGTATTCAAGCACACATCCACAGCTTGTGATAAGAAGATGGCCAAGAAACTGGCTTCCAAAGAAGTTTTGAAGTCTCTAAAGGGAGCCAATTTTAGCTAG

**>Gorai.009G374900.1_GrDCL2a_ protein sequence length= 1395aa**

MEPVDMEKDISQLHPADPLPFARSYQLEALEKAIKQNTITYLETGSGKTMIAIMLLRSYAHLIRKPSPFFAVFLVPKVVLVKQQADAVEMHTDLNVGKYWGDMQVDFWDGEKWKQELDKYEVLVMTPQILLDGLRHSFFKINMIKVLIIDECHHARGNHPYASIMREFYHRHLEAGASNLPRIFGMTASPINSKGANSADSYWQKIHELETIMNSKVYTCESESVLAQFVPFSTPKFKFYQHMEIPNVLYAHLVEELTVLKVKHECSLDNLDLEASAAESTRKKLSKIHSALIHCLHELGVWLALKAAECFSCYESEHLMWGNLDVFGEKIIRSYSVDAFHAIETCMPSGLDWTIANDVKGSVAAGFLTTKVLCLIESLFEYRVLKDIRCIIFVERVITAVVLQSLFSELLPRYSNWKTNYIAGNNSGLQNQTRKKQNEIVEEFRKGMVNIIVATSILEEGLDVQSCNLIIRFDPSPTICSFIQSRGRARMQNSDYLLMLKSGDFSTHSRLKNYLTSGDVMRKESLRHASNPCSPLSKGLDDEEFYQVASTGACMTLSSSVGLMYFYCSRLPADGYFKPIPRCVIDKQMGLCTLLLPKSCPIQTVCVQGNIKNLKKIACFEACKKLHQIGALTDNLVPDIVFEENDVEEFEKEPYNDDQPIFFPPELVNKGSLDSMTKYYCYLMELKQNFDYEVPVHNIMLLVRNQFDMDEKSVNIELEVDRGTLTVNMKYIGLIRLNSDQVILCRRFQLAVFQVLMDRKAEKFAEVLCDHTFGNNSEIDYLLLPSNYVGQSPLIDWLSVTSVTFSYEKAWKNHVNCNAGMIQTKSGLVCTCMVQNSLVSTPHNGHAYIISGLLTNINANSLLRLSDGRLMTYKEYYEQRHGINFCYGQVSFLAGRHIFPVQNHIQRFRKQKEKESSNALVELPPELCCVVMSPISVSTFYSFTFLPSIMHRLESLLLATSLKKMHLDHCVQNIAIPTMKVLEAITTKKCLENFHLESLETLGDSFLKYAVCQQLFKKYQNHHEGLLSIRKDKIISNTALSMLGCDKKLPGFIRDEPFDPKDWMIPGYNCGNYSLNEETLCNAKKIYVRGRRKVKCKKVADVVEALIGAYLSTGGEAAGLLFLDWIGISIDFTNIPYERHFKVRAEKFVNVQHFESLLHYSFQDPSLLVEALTHGSYMLAEIPGCYQRLEFLGDSVLDYLITLHLYNKYPGITPGLLTDLRSASVNNNCYALSAVKAGFHKHILQSSQKLYKDIKETVESFQELSLEYTFGWESEKSFPKVLGDVMESLAGAIFVDSGYKKEIVFQSIRPLLEPMITPETMTVHPVKELYELCQKEHYELRKPIVSHEDGISSITIEVEANGKVFKHTSTACDKKMAKKLASKEVLKSLKGANFS

**>Gorai.002G143000.1-JGI_221_v2.1_ID=Gorai.002G143000.1-JGI_221_v2.1_*Gossypium raimondii*| CDS|length=4182bp_DCL2b**

ATGGAGCCAGTTGGCATGGAGATGAACAATTCCCAACAACACTCTTCTGATCCTCTCCCATTTGCCAGAAGCTATCAGCTTGAAGCACTAGAGAAAGCTATCAAGCAAAACACAATAGCTTACTTGGAAACTGGCTCTGGCAAGACACTGATTGCCATCATGCTTCTCCGCAGATATGCCTACCTTATTCGGAAGCCTTCACCTTTTAATGCTGTCTTCTTGGTTCCCCAAGTTGTCCTGGTTGAACAACAAGCTGATGCTGTGGAAATGCATACGGACTTGAATGTGGGAAAGTATTGGGGAGATATGGAGGTTGACTTTTGGGATGATGCGAAATGGAAGCAAGAAATTGATAAATATGAGGTGCTTGTGATGACACCTCAAATTTTACTCAATGGATTGAGGCATGGCTTCTTCAAGATAAACATGATTAAGGTTTTGATAATTGATGAATGCCATCATGCCCGAGGAAAGCACCCTTATGCTTCCATTATGACAGAATTCTATCATCGGCAATTAGAAGCTGGTGTATCTGATCTTCCTAGGATTTTCGGGATGACTGCTTCCCCTATAAACACAAAAGGGGCAAACTCGGCTGATAGCTATTGGCAGAAGATCCATGAACTAGAGACGATTATGAATTCAAAGGTGTATACATGTGTAAGCGAATCAGTGCTTGCTCAGTTCGTTCCATTTTCGACTCCAAAGTTCAAGTTTTACCAAGACATGGAAATTCCATATGTTTTATATGCACGCTTGGTACAGGAATTGAATGTTTTGAAAGTAAAGCATGAATGTTCGTTGGACAATTTGGATCTTGAAGCTTCTGCAGCAGAATCTACGAGACGGAAAATATCGAAGATACATTCAGCTTTAATACATTGTTTAGATGAGCTTGGTGTCTGGTTGGCTTTAAAGGCTGCAGAGTACTTGTCATGTTATGAAAGTGAGTTTCTTATGTGGGGGAAATTGGATGTCTTTGGTGACAAAATTGTTAGGAGTTACAGTGTGGATGCTTTCCATGCCATTGAAACATGCATACCATCTGATCCAGATTGGACCATCGTTAATGATGTTAAAGCCAGTGTGGATGCTGGGTTTCTTACCACAAAAGTTTTATGCCTTATTGAATCTCTTTCTGAATACAGGGTGTTGAGAGACATAAGGTGTATAATTTTTGTGGAGAGAATTATAACAGCAGTTGTGCTTCAATCACTATTCAGTGAATTGCTTCCGAGGTACGGTAACTGGAAGACTAAATACATTGCAGGAAATAACTCTGGATTGCAGAACCAGACTAGGAAAAAACAAAATGAAATTGTAGAAGAATTCCGAAAAGGCATGGTAAACATAATTGTTGCAACTTCGATTCTTGAGGAAGGCTTGGATGTTCAAGCATGCAACTTAATTATCAGATTTGATCCTTCACCAACAGTTTGTAGTTTTATACAGTCTCGAGGACGTGCTAGAATGCAGAATTCGGATTATCTATTAATGGTGAAGAGTGGGGATTTTTCTACACATTCTCGACTGAAGAACTATCTTACTAGTGGAGATGTAATGAGAAAGGAATCTTTGTGCCATGCATCTGATCCTTGTTCTCCTCTAAGCAATGACTTATGTGATGAAGAGTTCTACCATGTTGCAAGTACAGGGGCATTTATGACTCTTAGTTCTAGTGTTGGTTTGATATACTTCTATTGCTCACGCCTCCCTGCAGACGGGTATTTTAAATGTACTCCGAGGTGTATCATAGACAAGCAAATGGGGGTTTGCACCCTCCATCTACCCAAGAGTTCCCCTATACGAACTGTTTGTGTTAAGGGTAACTTTAAAAGCCTAAAGCAGAAGGCATGCTTTGAAGCATGCAAGCAACTCCATCAAATTGGTGCTTTGACAGATAATCTTGTGCCTGATATTGTTGTGGAAGAAAATGATGCTGAAGAAATTGGAAAGGAATCCTATAATGATGATCAACCGATTTTTTTTCCACCTGAACTGGTGAATCAAGATTCACAGGAGTCCATGACAAAATACTACTGCTACTTAATGGAGTTGAAGCAGAACTTTGGTTATGAGTTTCCTGTTCAAAACATCATACTCCTTGTCAGGAGTCAGCTTGAAATGGAAGCAAAAAGTGTGGGTATTGAGTTAGAAGTTGATAGGGGCACTCTGACAGTTAACTTGAAATATATTGGACTGATACGTCTTAGTTCTGACCAGGTTATCTTGTGTAGAAGGTTTCAGATTGCTCTTTTTCGAGTTCTTATGGATCATAAAGCAGAAAAGTTGACAGAGTTATCTGACCTTACGTCAGGGAACAATTCTGAAATCGATTATCTTCTTCTCCCATCAAATTACATGGGCCAAAATCCCGTGATTGATTGGTTGTCAATTTCTTCCATCTTATTTTCTTATGAGAAGGTCTGGAAGAATCATGTGAACTGCAATGCTGGCATGATACAGACCAAAAGTGGTCTGGTGTGCACTTGCATGATAAAAAATTCGTTGGTCTCTACTCCTCATAATGGTCGCACATATATTATTGACGGTCTTTTAAATAATATAAATGCAAATTCACTTTTGACACTGAGTGATGGAGGAGTAATGACCTACAAGGAATATTATGAACAGCGACATGGTATCCATTTATGTTTTAGTCGAGTCTCTTTCCTTGCTGGGCGACACATTTTCCCCGTGCAAAAGCACATTCAAAGGTGCAAAAAACAGAAAGAGAAAGAATCCAGTAACGCATTTGTAGAATTGCCTCCTGAGCTTTGTGATGTAATAATGTCTCCCATATCGATTAGCACATTTTATTCTTTCACATTTCTTCCATCAATCATGCATCGACTCGAATCTTTACTCCTTGCTACCATCTTGAAAAAGATGCTTCTCGATCATTGTGTGCATGATGTTGCTATTCCAACCATGAAGGTTTTGGAGGCAATTACCACAAAAAAGTGCCTGGAAAGTTTTCATTTAGAATCACTGGAGACTCTTGGTGATTCTTTTTTGAAGTATGCTGTTTGTCAACAGCTATTCAAAAAACATCAGAATCATCATGAGGGCCTTCTTAGTATTAGGAAGGACAAAATGATTTCAAATACAACCCTTTGCATGCTGGGATGTGACAAGAAACTTCCGGGATTTATCCGTGATGAGCCTTTCGATCCCAAAGGTTGGATGGTTCCTGGTTATAATTGTGGAAGACATACATTAAATGAGGAGAAATTAAATACAAGAAAAATATACGTTAGTGGGAGAAGGAAGCTGAAGAGTAAGAAGGTTGCTGATGTTGTTGAGGCTCTTATTGGTGCATACCTTAGCACGGGAGGTGAAGTAGCTGCATTATTATTTATGAATTGGATTGGTATAACTATTGATTTTAGAAATATACCATACGAAAGACATTTCGAAGTGCAGGCTGAAAAGATTGTTAATGTCCAACATTTAGAGTCTCTTCTTAACTATTCATTCCAGGACCCTTCTTTGTTAGTGGAAGCACTAACACATGGTTCTTACATGCTTGCTGAAATTCCAGGATGTTATCAGCGGCTGGAATTTCTAGGGGACTCCGTTTTAGATTATCTTATCACTCGGCATTTATATAATAAATATCCTGGGATCTCACCAGGATTATTAACGGATCTGAGGTCAGCATCTGTTAACAACAATTGCTATGCATTATCAGCTGTCAAGGCTGGATTGCATAAGCACATTCTCCAAAGTTCACAGAAGCTTTACAAGCATATAAAAGAAACTGTTGAAAGTTTTCAGGAATTATCGTTGGATTGCACTTTCGGTTGGGAATCTGAAAAATCTTTCCCCAAGGTACTGGGTGATGTGATGGAGTCAATTGCTGGGGCTATATTTGTTGATTCAGGATACAACAAGGAAACTGTATTCCGGAGTATCAGGCCACTGTTGGAGCCCCTGATTACTCTAGAGACTATGACGGTTCACCCCGTGAAGGAGTTGAATGAACTCTGTCAAAAGAAGCATTATGAACAAAGAAAACCCATTGTTTCACATGGCAATGGGGTTTCTTCTGTTACCATAGAGGTTGAAGCAAATGGCGAGGTATTAAAGCACACATCAACAGCTTGTGATAAGAAGATGGCCAAGAAACTGGCTTCCAAAGAAATTTTGAAGTCCCTAAAGCTAGCCAATTTTTGCTAG

**>Gorai.002G143000.1_GrDCL2b_ protein sequence length= 1393aa**

MEPVGMEMNNSQQHSSDPLPFARSYQLEALEKAIKQNTIAYLETGSGKTLIAIMLLRRYAYLIRKPSPFNAVFLVPQVVLVEQQADAVEMHTDLNVGKYWGDMEVDFWDDAKWKQEIDKYEVLVMTPQILLNGLRHGFFKINMIKVLIIDECHHARGKHPYASIMTEFYHRQLEAGVSDLPRIFGMTASPINTKGANSADSYWQKIHELETIMNSKVYTCVSESVLAQFVPFSTPKFKFYQDMEIPYVLYARLVQELNVLKVKHECSLDNLDLEASAAESTRRKISKIHSALIHCLDELGVWLALKAAEYLSCYESEFLMWGKLDVFGDKIVRSYSVDAFHAIETCIPSDPDWTIVNDVKASVDAGFLTTKVLCLIESLSEYRVLRDIRCIIFVERIITAVVLQSLFSELLPRYGNWKTKYIAGNNSGLQNQTRKKQNEIVEEFRKGMVNIIVATSILEEGLDVQACNLIIRFDPSPTVCSFIQSRGRARMQNSDYLLMVKSGDFSTHSRLKNYLTSGDVMRKESLCHASDPCSPLSNDLCDEEFYHVASTGAFMTLSSSVGLIYFYCSRLPADGYFKCTPRCIIDKQMGVCTLHLPKSSPIRTVCVKGNFKSLKQKACFEACKQLHQIGALTDNLVPDIVVEENDAEEIGKESYNDDQPIFFPPELVNQDSQESMTKYYCYLMELKQNFGYEFPVQNIILLVRSQLEMEAKSVGIELEVDRGTLTVNLKYIGLIRLSSDQVILCRRFQIALFRVLMDHKAEKLTELSDLTSGNNSEIDYLLLPSNYMGQNPVIDWLSISSILFSYEKVWKNHVNCNAGMIQTKSGLVCTCMIKNSLVSTPHNGRTYIIDGLLNNINANSLLTLSDGGVMTYKEYYEQRHGIHLCFSRVSFLAGRHIFPVQKHIQRCKKQKEKESSNAFVELPPELCDVIMSPISISTFYSFTFLPSIMHRLESLLLATILKKMLLDHCVHDVAIPTMKVLEAITTKKCLESFHLESLETLGDSFLKYAVCQQLFKKHQNHHEGLLSIRKDKMISNTTLCMLGCDKKLPGFIRDEPFDPKGWMVPGYNCGRHTLNEEKLNTRKIYVSGRRKLKSKKVADVVEALIGAYLSTGGEVAALLFMNWIGITIDFRNIPYERHFEVQAEKIVNVQHLESLLNYSFQDPSLLVEALTHGSYMLAEIPGCYQRLEFLGDSVLDYLITRHLYNKYPGISPGLLTDLRSASVNNNCYALSAVKAGLHKHILQSSQKLYKHIKETVESFQELSLDCTFGWESEKSFPKVLGDVMESIAGAIFVDSGYNKETVFRSIRPLLEPLITLETMTVHPVKELNELCQKKHYEQRKPIVSHGNGVSSVTIEVEANGEVLKHTSTACDKKMAKKLASKEILKSLKLANFC

**>Gorai.010G093100.1-JGI_221_v2.1_ID=Gorai.010G093100.1-JGI_221_v2.1_*Gossypium raimondii*| CDS|length=5028bp_DCL3a**

ATGTCTTTGCTGAAGTCCCATTCAACACCCCAATTTTCTCCGGTTATCTTAACCAAACTAATCGAGGAACAGGGTGAGCCTCTAAAACGAAGTTTCAGTGAGGCGAACTCAGACCCACCTGATTGGATGGTTCTAGACGACAATGAAACTGCTAAGGAAGAAGACCCTTCTTCATCTTCAAAACCTAAGGACTTTAACCCCAGAGGGTATCAATTGCAGGTATATGAGGTGGCAAAGGGAAGAAACATAATAGCAGTGTTGGATACAGGTGTAGGGAAGACAATGATAGCTGTGATGCTCATAAAAGATTTTGTTCAAGCTATCGAGTCTACTGAAAGTAAAAAATTGATTATTTTCTTGGCTCCCACTGTTCATCTAGTCAATCAGCAATTTGAATATGTAAAAGATCATACTAGTTTGGAAGTTGAACAGTATTATGGAGCTAAGGGGGTTGATGAATGGACCTTGGATTGTTGGGAGAAAGAAACAAAGGAGCATGATGTTCTGGTTATGACACCCCAAATTCTATTGGATGCCTTAAGGAAGGCATTCTTGAGTCTGGAGATGGTGTCCTTAATGATAATAGACGAGTGCCACCGTGCTACTGGCAACCATCCATATGCCAAAATAATGAAGGAATTTTATCACAAATCTAATAACAAGCCAAAGATTTTTGGAATGACAGCATCACCTGTGGTTAGCAAAGGTGTGTTGTCCAGCAATAATTGCGATGGTCAAATGTCAGAACTTGAATGTGTTTTGGATTCCTTGATATACACTATTGAAGACAGGACAGAGATGGAAGCGTGTGTTCCTTCTGCTAAAGAAAGTTGTAGATTTTTTGATCCAGCACAATTTTCTAGCTTGGATTTGAAAGCAAAGGTAGAAGCCTCTTGGTTAAAGACAGATGCGTCATTGTCAAATTTGCAAAGCTCACTGCAAACTTCTTATAAAGGCATGGATGATAAACTTAAGAATCTACGGAAGCGATTATCAAATGACCATGCTAAGGTTCTGCATTGCCTTGATAATCTTGGTCTCATATGTGCTTATGAGGCTGTTAACATTTGTCTAGAGAACATCCTTGACACCACAGAGGAATCTAAAGCATATAGAGAAAGTGTGTTGCAGTATAAAAATTTCCTTGAGGAAGTCCAATGTAGAATTGGGGAGTCTCTTCCCCTCGGTGATAAAAATTTTCTGAATACTGGATTTGACTATTTGAAGGCAGTAGATCTTGGCTATATTTCTCCAAAACTGCATGAACTACTTCAACTGTTCCAATCATTTGGAGAAACTAGACAAGTATTGTGCCTGATTTTTGTTGAAAGAATTATTACTGCTAAAGTAATTGAAAGATTTGCGAAGAAAGTAAGCTGTTTATCACATTTCATGGTTTCATATATGACTGGAAGTAATACATCTGTTGATTCCCTGGCACCAAAAATGCAAAAGGAAACCTTGGAGTCATTTCGATCTGGGAAGGTGAATCTATTGTTTACTACTGATGTGGTTGAGGAGGGAATTCATGTACCAAATTGCTGTTATGTGATACGTTTCGACCTGCCAAAGACAGTCCGGAGTTATGTACAATCTCGAGGAAGAGCTAGGCAGAACAATTCTGAATTCATTATGATGCTTGAGAGGGGAAATGTGAAACAAAGAAATCAACTATATGATATAATCAGGAGTGAGTATTCAATGACAAATTCGGCAATTAAAAGAGATCCCGATTCTGATCCATGCCTTCTTAAAGATCATACATTTGAAGAAACAAATGTTTTTATTGTGGATGCTACTGGAGCTTCAGTTACTGCAGATTCTGCTGTTAGCCTCATCCATAAATATTGTGGGAAGCTCCCCGGTGATAAGTATTACACACCAAAACCAAATTTCCAGTTCACGTCTTCTGAAGGATTATATAAGTGTAAATTAACATTACCTGTTAATGCAGCCGTTCAAACAATAGTTGGTCCACTGTCTAGGAATTCTCATTTAGCAAAGCAGCTTGTATGCTTAGAAGCATGTAAGCAGCTTCATCAAATGGGTGCCTTGGATGATCATCTCACTCCATCCATTGAAGAGCCTTCAGAAAATGCTTGTATTTCTAAAGGAAAAGATTCAGGTGCAGGTGCAGGTGCAGGAACTACAAAACGGAAGGAGCTTCATGGAACAACTTGCATACAGGCATTATGTGGAAGCTGGGGAGAAAAATCTGATGATGCTGTTTTCTTTGCCTATAAATTTGACTTCAAATGCAATATCATTACTGTGGTTTATTCTGGATTTGTTCTTCTAATTGAATCAAAGCTTGCGGATGATGTGGGGAATACTGAAATGGATCTTTTCTTGATTGGTAAGATGGTTAAGGCTAGCGTTTCTTCCTGTGGGCAAGTGTATTTGAATGCAGAACAGATGGTGAAAGCGAAGCGCTTTCAGGAATTTTTCTTTAATGGCTTGTTTGGGAAGTTATTTGTTGGATCTAAATCATCTGGAGCACCAAGAGAATTTCTACTTCGGGATAAAACAAGCTCCTTGTGGAGTCCATCCCACATGTATTTGCTTTTGCCCCTCGAGGATAATTCAACTGATGAATTGAGAATACACTGGCCAGGGATAACAGCTTGCACATTAGCTGCGGAATTTTTAAATAAAAATTCTTTGTTGGGCACTGAGCAATCTGATGATGATGGAAGCAATCCATCATTGAACAGTACTGGTTCACCTGTGACATACTGCAAAGAAACCAATATAATCCGCTTTGCTAACAGTTCAGTTGATGCTAATAGTCTTAGAAATACGGTAGTATTGGCTATTCACACTGGAAGAATCTACTGCATCATTGAAGCTGTGAGTGATAAAACTGCTGAAAGTTCTTTCGCTGAAACTGTTGATACGGTCTCATCAGAGTTTGCTACCTTCTATGAATACTTCTACAAAAAGTACAATATTGTGCTGAAACATCCAGGACAGCCTTTGATGCTGTTAAAGCAGAGCCATAACCCGCACAACTTGCTTGTGAATTTTAATGATGAAGGTGTATCAGCTAAGGCATCACAAGCTGGCGTGGTTAATGAAAAGCCTCGGTTTCATGTCCACATGCCCCCTGAGCTTTTACTTGTCCTTGATGTCCCAGTGAGTGTTCTAAAATCATTGTACTTACTGCCATCATTGATGCATCGGCTGGAGTCCTTAATGTTGGCCAACCAACTCAGAGAAGAGATAAACTTTTGTTCTAGCAACATTGATATTCCAAGCTCAATGATCCTGGAAGCATTAACAACACTTAGATGCTGTGAAAGTTTTTCAATGGAAAGGCTGGAATTGCTTGGGGATTCAGTTCTGAAGTATGCTGTTAGCTGCCACCTCTTTCTTAGATATCCCAACAAACATGAGGGACAATTATCTGCCAGGCGTTCGTTGGCAGTTTGTAATTCAACCCTGCATAAGTTGGGAACTGACCACAAAATACAGGGATACATTAGGGACAGTGCTTTTGATCCCCGTCGTTGGGTAGCTCCAGGACAACGAGTGCTTCGCCCTGTTCCTTGCAAGTGTGGTGTGGACTCTCTAGAAGTTCCTTTAGATAAAAAATTTCAGACAGAAGACCCTAAAGTTAAGGTTGGTAAATCCTGTGATAGAGGCCACCGATGGTTGTGTTCAAAAACCATATCAGATTGTGTTGAAGCTCTCATAGGAGCATACTACTTAAGTGGTGGACTTGTTGCTGCCCTTCATGTGATGAAGTGGCTCGGGATTGATGCCGAACTTGATCCCTTGGTGGTAGCTGAGGTCATTAATCAGGCATCCTTAAGAACTTATGTCCCCAATTACGAAATCCACATGATAGAGTCAAAAGTTGGCTACAATTTTTCTGTCAAGTTTTTCTTGCAGGAGGCATTGACACATGAATCTTTGCACGAGTCCTACTGTTACCAGAGACTTGAATTTCTTGGTGATTCTGTGTTGGACTTGCTGATCACCCAGTATCTCTACAACCATCACACTGATATAGATCCGGGTGAGTTGACTGACCTGCGCTCCGCTTCGGTTAATAATGAAAATTTTGCTCAAGTTGCCGTGCGCCATGACCTTCATAAGCATCTTCAACATTGTTCAACTTTACTATCAAACCAAATAAGTGAATATGTGCGGTCCTTCACTGAATCTGATAACACCACCAGATTAGATCCCAGCATAAAAGGTCCCAAGGCTCTTGGAGACCTGGTTGAAAGTATTGTTGGGGCAATTCTGATTGATACAAACCTGAATCTTGACAAAGTCTGGAGAATTGTTGAACCGTTGTTGTCTCCAATTGTAACCCCAGATAAGCTTGAGCTGCCTCCATTCCGGGAACTGAATGAATTATGCGACTCTCTTGGATATTTTATTAAGGAAAAATGTAAAAATAAGGGGGAAGTAGTGCACGCCGAGCTTCTATTGCAGCTAGATCATGATTTGTTGGTAGGAGAGGGGTTTGATAGAAGTAGAAAAGTAGCAAAAGGAAAAGCCGCTTCTTGTCTGTTGAAGGACCTTGAGAACAGAGGAATCTCCAGGAAGAAAAGGAAACATGACTGTGTGGATTCAAGTCAAACCATGGAAGATGACTCGTTAGAACCAACAATCCCTAAGAGGCAAAGGAGCGCCGAAATTCAGTTGCTTGATGAATCAAAGAAAGCATGCAGTGCTACGCCTGCCACCCCAGTTATTGTAACAGTAAAAACGAAGAAAGGAGGGCCGAGGACCACTCTTTTTGAGCTTTGCAAGAAACTGCTGTGGCCTATGCCTAGTATCAAAGCAACAGAACACAAATCAAGTGCTCCCATGGAAATTGGTGAAGGTCCCGAGAGGAAAAAAGGGTTTATCAGCTTTGTGTCGAAAATCATCTTGAATGTCCCAGGATACGACATTATTGAATGCACTGGAGATGCAAAAGCTGACAAAAAAAGTTCTTCAGACTCAGCAGCACTGTTTATGCTTTATGAGCTTGAACAGCGTGGGAAGCTCATCATCGAGGAAACCTTATAA

**>Gorai.010G093100.1_GrDCL3a_ protein sequence length= 1675aa**

MSLLKSHSTPQFSPVILTKLIEEQGEPLKRSFSEANSDPPDWMVLDDNETAKEEDPSSSSKPKDFNPRGYQLQVYEVAKGRNIIAVLDTGVGKTMIAVMLIKDFVQAIESTESKKLIIFLAPTVHLVNQQFEYVKDHTSLEVEQYYGAKGVDEWTLDCWEKETKEHDVLVMTPQILLDALRKAFLSLEMVSLMIIDECHRATGNHPYAKIMKEFYHKSNNKPKIFGMTASPVVSKGVLSSNNCDGQMSELECVLDSLIYTIEDRTEMEACVPSAKESCRFFDPAQFSSLDLKAKVEASWLKTDASLSNLQSSLQTSYKGMDDKLKNLRKRLSNDHAKVLHCLDNLGLICAYEAVNICLENILDTTEESKAYRESVLQYKNFLEEVQCRIGESLPLGDKNFLNTGFDYLKAVDLGYISPKLHELLQLFQSFGETRQVLCLIFVERIITAKVIERFAKKVSCLSHFMVSYMTGSNTSVDSLAPKMQKETLESFRSGKVNLLFTTDVVEEGIHVPNCCYVIRFDLPKTVRSYVQSRGRARQNNSEFIMMLERGNVKQRNQLYDIIRSEYSMTNSAIKRDPDSDPCLLKDHTFEETNVFIVDATGASVTADSAVSLIHKYCGKLPGDKYYTPKPNFQFTSSEGLYKCKLTLPVNAAVQTIVGPLSRNSHLAKQLVCLEACKQLHQMGALDDHLTPSIEEPSENACISKGKDSGAGAGAGTTKRKELHGTTCIQALCGSWGEKSDDAVFFAYKFDFKCNIITVVYSGFVLLIESKLADDVGNTEMDLFLIGKMVKASVSSCGQVYLNAEQMVKAKRFQEFFFNGLFGKLFVGSKSSGAPREFLLRDKTSSLWSPSHMYLLLPLEDNSTDELRIHWPGITACTLAAEFLNKNSLLGTEQSDDDGSNPSLNSTGSPVTYCKETNIIRFANSSVDANSLRNTVVLAIHTGRIYCIIEAVSDKTAESSFAETVDTVSSEFATFYEYFYKKYNIVLKHPGQPLMLLKQSHNPHNLLVNFNDEGVSAKASQAGVVNEKPRFHVHMPPELLLVLDVPVSVLKSLYLLPSLMHRLESLMLANQLREEINFCSSNIDIPSSMILEALTTLRCCESFSMERLELLGDSVLKYAVSCHLFLRYPNKHEGQLSARRSLAVCNSTLHKLGTDHKIQGYIRDSAFDPRRWVAPGQRVLRPVPCKCGVDSLEVPLDKKFQTEDPKVKVGKSCDRGHRWLCSKTISDCVEALIGAYYLSGGLVAALHVMKWLGIDAELDPLVVAEVINQASLRTYVPNYEIHMIESKVGYNFSVKFFLQEALTHESLHESYCYQRLEFLGDSVLDLLITQYLYNHHTDIDPGELTDLRSASVNNENFAQVAVRHDLHKHLQHCSTLLSNQISEYVRSFTESDNTTRLDPSIKGPKALGDLVESIVGAILIDTNLNLDKVWRIVEPLLSPIVTPDKLELPPFRELNELCDSLGYFIKEKCKNKGEVVHAELLLQLDHDLLVGEGFDRSRKVAKGKAASCLLKDLENRGISRKKRKHDCVDSSQTMEDDSLEPTIPKRQRSAEIQLLDESKKACSATPATPVIVTVKTKKGGPRTTLFELCKKLLWPMPSIKATEHKSSAPMEIGEGPERKKGFISFVSKIILNVPGYDIIECTGDAKADKKSSSDSAALFMLYELEQRGKLIIEETL

**>Gorai.013G221400.1-JGI_221_v2.1 ID=Gorai.013G221400.1-JGI_221_v2.1_ *Gossypium raimondii*| CDS|length=4389bp_DCL3b**

ATGCATTCTCCCATGGAAGAAGAGCAGTGTTACCCTCTAAAAACAACCTTCAGTGAAACACACTTACACCCATCTCAGATTATGGTTTCAATCAATGACGATCATGCTAAGAACCACCACCCTTCTTCATCTTCAAATCCTGATCACTCTAGCCCTAGAGGGCATCAGCTGCAGGTTGTTAGATACAAAATGATAGCTGTAATGCTTATCAAAGATTTTGTTCAACCTATCAACTCCATTGATAAAAAAAAGTTGATTATTTTCTTGGCCCCCACTGTTCATCTTGTTAATCAGCAATTTGAATATATAAAGTTCCATACAAGTTTGGATGTTGAACAATATTATGGAGATAAAGGGGTTAATGAATGGAACTCAGGGTATTGGGAGAAAGACATAAAGGAGCATGATGTTCTTGTTATGACACCCCAAATTCTGTTGGATGCCTTGTGGAAAGCATTCTTGAGTTTAGAGATGGTGTCCTTAATGATAATCGACGAGTGCCACCATGCTACCGGTAACCATCCGTATGCGAAAATAATGAAGGAATTCTATCACAAATCGAACAACAAACCGAAGATTTTCGGGATGACGGCATCGCCCTTAGTTAGCAAAGGTGCATTATCCAACAATGATTGTGAGGATCAACTTTCAGAACTTGAAAATGTTATGGATTGCATGATATATGCAATTGAAGACATGACAGAGATGGAAACATATGTTCCTACAGCAAAAGAAAGTTGTAGGTTTTTCGATCCAACACGATTTTGTAGTTCGGGTTTGAAAGCAATGATCGAAGCCTCGTGGTTAAAGATAGATGCTTCATTATCAACGTTGCAAGGCTCGATTCAAACTTCTTATACAGACATGGATGATAAGTTTAAGGCCCTCCATAAACGGTCATCCAATGACCATGCTAAGATTCTGCATTGTCTCGACGATCTTGGTCTCATATGTGCTTATGAGGCTGTTAAAGTCTGTCTTGAGAACATCCCTGATACAAAAGAGGAGTGTGAAGCATATAAAGAAGGTGTATTGCAGTGCAAAAGGTTCCTCAACGAAGTGCTACAGATAATCAGGGAATCCCTTCCCCTGGGTGATGAAAATTTTCTGAATTCCGGGTTTGACTACTTAAAGGCGGTAGATTCGGGCTATATATCTCCGAAACTGCATGAACTAATTGAATTTTTCTTATCACTCGGAGAACCTAGTCAAGTATTGTGCCTCGTTTTTGTTGAAAGAATTGTTACCGCTAAAGTAATTGAAAGATTTGTGAAGAAAGTAAGTTGTTTATCGTGTTTTGTGGTTTCGTGTTTGACTGGAAGTAATACGTCAGTTGATTCCATGGCACCAAAGATTCAAAAGGAAACTTTGGAATATTTTATGGTCAATGATTGTGGAGAGGGAATTCATATACCAAACTGCTCATATGTGATACGTTTTGACTTGCCGAAAACAGTGTTGAGTTATGTACAATCTCGAGGAAGAGCTAGGCAGAGTGGTTCCCAATTCATCATGATTCTAGAGAGGGGAAATGAGAAACAAAGAGATCAACTATACGATCTAATTCGGAGTGAACATTCAATGACGAATACAGCTATGAATAGAGATCCTGATCTATCTCTTCCGGAAAACCGTACATTGGAAGAAACAAATGTTTCTATCGTGGCTGCTACTGGAGCTTCCATTGAAGAAACTTCCAAAAATCGTTGCGCTTCTAAAGGAAAAGATCCCATTTTCGATTCAGGAACTACAAGATGGAAGGAGCTGCACGGAACAACTCAGATTCGTGCATTTTCCGGGAGCTGGGGAGAAAAAGCCGATGCTTCAGTTTTCTTTGCCTATAAAATTGACTTTTCATGCAATGTTGTTAGCGTTGTTTACTCTGGATTTCTTACTGATGATGTGGGGAACATCGAAGTAGATCTTTACTTGATCGGAAAGATAGCTAAGGCTCGTGTTTCTTTTTATGGGAAAGTACATTTGGATGCTGTACAGATTACCGCAGCAAAGCGTTTTCAGGAAATTTTCTTTAATGGCTTGTTCGGGAGGCTGTTTGTTGGATCATCCAGAACATCGAGGGAACTTTTATTTCATAGTAAAACGAGCTTGTTGTGGCATCCATTTAACATGTATTTGCTTCTGCCCCTAGAGGATAGTTTGAGTAATGAACTGAGAATAAACTGGCCTGGGATAACAGCTTGTACATTAGCTGTAGAGTTTTTAACTGCTGACGGTAACAGAGGTAATCCGTCATTGAACCAAACTGATTCATCCGTGACAGAACACAAGGAGACCAATGTAATCCACTTTGCTAACAGATCTATAGATGTTAACAATATCAGTAACATCGTAGTATTGGCAATTCATACCGGAAGAATCTATTCCACCATTGAATTAGTTCATGATACATCCGCCAAAAGTTCTTTCAATGACATTGTTGATATGAACTCATTGAAGTTCGCGACCTTCTCCGAATACTACAACAGAAAGTACTGCATCGTGCTGAAACATCCAGGACAACCTTTATTGCTGTTAAAGCAGAGTCATAACCCGCACAACTTGCTGGTTGATTTTAACGATGAAGGTTGCAGATTTTCAGCACAAGCATTACAAGCAAGCTCGGTTAATGAAAAGTCATGGAATACTATCCACATGCCACCCAAGCTTTTGCTCATCCTTGATGTCCCGGTCTATGTTCTAAAAGCCTTTTACTTACTACCATCACTGATGCACCGGCTCGAGTCCTTAATGTTGGCTAGCCAACTTAGGGAAGAGATCGATTTTCGTTCAAGCAACTTCGATATACCGAGCTCAATGATCCTGGAAGCGTTAACAACGCTTAGATGTTGTGAAAGCTTTTCGATGGAGAGGCTGGAATTTCTCGGTGATTCAGTTCTGAAGTATGCTGGTTACATTAGAGACAGTGCTTTCGACCCCCGACGTTGGGTAGCTCCAGGACAACTACGTCTTCGGTCTTTTCCTTGTAACTGCAGGGTGGACTCTCGAGAAGTTCCTTTAGATAAAAAGTTTCAGACTGAAGATCTGGAAATTAAGGTCAGTATATCTTGTGATAGAGGCCACCGATGGATGTGTTCGAAAACCATATCGGATTGTGTTGAAGCTCTCATAGGAGCATACTATGTAAGTGGTGGACTCATTGCTGCTCTTCATATGATGAAATGGCTTGGGATCGATGCCGAACTTGATCCCTCTTTGGTAGCTGAAGCCATTAATCGTGCATCCCTACAATCTTATAAATTTTCTATGAAGTTTTTCTTACAGGAGGCCTTGACACATGCATCGGCAAACGAGTTTTACTGTTACCAGAGGCTTGAATTTCTTGGTGATTCCGTGTTGGACTTGCTGATAACCCGATATCTCTACTGCAATCACACCGATATAGATTCAGGAGAACTGACTGACCTCCGATCGGCTTCAGTTAGTAATGAAACTTTTGCTCAAGTTGCTGTCCGTAATGACCTTCATAAGCATCTTCGACATTGTTCAACTTTACTATTGGACCAAATAAGTGAATATGCACAGTCCCTCCCTGAATCTTGGGACACTACACGATCAGTACCCGGCATAAAAGGTCCAAAGATATGTTCAAACCTTATTGAAAGTATCGTCGCGGCAATCTTGATCGATACAAACCTGAACCTTGACGAAGTTTGGAGAATTGTTGAGCCATTGTTATCTCTGATCGTGACTCCCGATAAGCTCGAGGAAAAGTGCGTTAATACGGGAGAAATGGTGCATGCCAAGCTTTGGTTACAGCTGGATGATATTTTGTTGGTCGGAGAGGCATTCAATCGAAGCAAAAAGGTAGCAAAAGGAAAAGCAGCTTTATATCTATTGAAGGAACTCGAGGATAGAGGGATCTCCCGAAAGAGAACGAAACAGGATCATGCCGGTTCGAGTGGAACCACTGATGATGATTTGTTAGAGCCAAAAATGTGCAAGAAGCAGAGGATAGCTGAGATTCCTTCTCCGACTAACGACTCTTCAAGGACAGCATATGGAGCTGAGATTCCTTCTCCGGCTAACGCTTCTTCAAGGACAGCATATGTTATTGCAAGGATAAACACAAATAAAGGACGACCGAGGGCCACTCTTTACGAGATTTGTCGGAAACAGTTATGGCCTAGGCCTACATTCGAAACAACAGAAGGAAGATCACGTTCTCCTATGGAAATCGGCAAAGGTGCAGACCGAAAAATCGGGTTTAACAGCTTTGTAACGAAGATCACCTTGAATGGACCGAGTTCAGGCATTATTGAATGCAGCGGAGATGTAAGAGCCGACAAAAAGAGCTCTTGTGACTCTGCAGCAATCCTTATGCTTTACAGGCTTAAAGAGCTAGGGAAGCTCATCATCCGCGAATCCTAG

**>Gorai.013G221400.1_GrDCL3b_ protein sequence length= 1462aa**

MHSPMEEEQCYPLKTTFSETHLHPSQIMVSINDDHAKNHHPSSSSNPDHSSPRGHQLQVVRYKMIAVMLIKDFVQPINSIDKKKLIIFLAPTVHLVNQQFEYIKFHTSLDVEQYYGDKGVNEWNSGYWEKDIKEHDVLVMTPQILLDALWKAFLSLEMVSLMIIDECHHATGNHPYAKIMKEFYHKSNNKPKIFGMTASPLVSKGALSNNDCEDQLSELENVMDCMIYAIEDMTEMETYVPTAKESCRFFDPTRFCSSGLKAMIEASWLKIDASLSTLQGSIQTSYTDMDDKFKALHKRSSNDHAKILHCLDDLGLICAYEAVKVCLENIPDTKEECEAYKEGVLQCKRFLNEVLQIIRESLPLGDENFLNSGFDYLKAVDSGYISPKLHELIEFFLSLGEPSQVLCLVFVERIVTAKVIERFVKKVSCLSCFVVSCLTGSNTSVDSMAPKIQKETLEYFMVNDCGEGIHIPNCSYVIRFDLPKTVLSYVQSRGRARQSGSQFIMILERGNEKQRDQLYDLIRSEHSMTNTAMNRDPDLSLPENRTLEETNVSIVAATGASIEETSKNRCASKGKDPIFDSGTTRWKELHGTTQIRAFSGSWGEKADASVFFAYKIDFSCNVVSVVYSGFLTDDVGNIEVDLYLIGKIAKARVSFYGKVHLDAVQITAAKRFQEIFFNGLFGRLFVGSSRTSRELLFHSKTSLLWHPFNMYLLLPLEDSLSNELRINWPGITACTLAVEFLTADGNRGNPSLNQTDSSVTEHKETNVIHFANRSIDVNNISNIVVLAIHTGRIYSTIELVHDTSAKSSFNDIVDMNSLKFATFSEYYNRKYCIVLKHPGQPLLLLKQSHNPHNLLVDFNDEGCRFSAQALQASSVNEKSWNTIHMPPKLLLILDVPVYVLKAFYLLPSLMHRLESLMLASQLREEIDFRSSNFDIPSSMILEALTTLRCCESFSMERLEFLGDSVLKYAGYIRDSAFDPRRWVAPGQLRLRSFPCNCRVDSREVPLDKKFQTEDLEIKVSISCDRGHRWMCSKTISDCVEALIGAYYVSGGLIAALHMMKWLGIDAELDPSLVAEAINRASLQSYKFSMKFFLQEALTHASANEFYCYQRLEFLGDSVLDLLITRYLYCNHTDIDSGELTDLRSASVSNETFAQVAVRNDLHKHLRHCSTLLLDQISEYAQSLPESWDTTRSVPGIKGPKICSNLIESIVAAILIDTNLNLDEVWRIVEPLLSLIVTPDKLEEKCVNTGEMVHAKLWLQLDDILLVGEAFNRSKKVAKGKAALYLLKELEDRGISRKRTKQDHAGSSGTTDDDLLEPKMCKKQRIAEIPSPTNDSSRTAYGAEIPSPANASSRTAYVIARINTNKGRPRATLYEICRKQLWPRPTFETTEGRSRSPMEIGKGADRKIGFNSFVTKITLNGPSSGIIECSGDVRADKKSSCDSAAILMLYRLKELGKLIIRES

**>Gorai.009G053300.1-JGI_221_v2.1 ID=Gorai.009G053300.1-JGI_221_v2.1_ *Gossypium raimondii*| CDS|length=4965bp_DCL4**

ATGCCGGGCGGCGAATTGTCCACCGACGGGACCGAGCCGTTCATGGCCTCTAAAGTTAAAGCCTTTTCTACTCCGTCGCCGATCGTTGAAATCAGCCGAAAAGATGAACCTATAATGGAAAAGAAAGAGAAGGATCCACGAAAAATTGCTAGAAAGTATCAACTGGAGTTATGCAAGAAAGCCATGGAAGAGAACATAATTGTTTATTTGGAGACTGGTTGTGGGAAGACCCACATCGCAGTTCTGCTTATTTATGAGCTTGGCCATTTGATACGGAAACCTCAGAATAGGGTATGCATTTTTCTTGCTCCCACAGTGGCTCTTGTTCAGCAGCAAGCCCGGGTTATAGAAGATTCTCTTGATTTCAAGGTTGGGACTTATTGTGGTAACTGCAGACACTTGAAGAACCATCATGACTGGGAAATAGAGATCAAAGAATATGAGGTTCTTGTTATGACTCCCCAAATACTACTTCGTAGCTTATATCACTGCTTCATCAGGATGGACTTAATCGCTCTTCTGATATTTGATGAGTGTCATCATGCTCAAATAAAAAGCAACCATCCTTATGCAGAAATCATGAAAGTTTTTTATGATAAAGCTACTGCATCAATGCTTCCTCGTATATTTGGAATGACAGCATCTCCCGTTGTTGGTAAAGATGCTTCCAGTCAAGTAAATTTACCTAAAAGCATCAACAGCCTCGAAAACTTGCTTGATGCTAAGGTATATTCAGTTGGAGATAAGGAGGAATTGGAAAGCTTTGTAGCATCTCCTGTGGTTAGAGTATATGATTATGGTCCTGTCAATTTTGGTTCTTCTTGTTCCACCATTATCTATTGTAGTAAACTTGAGGAGATTAAGCGCCAGTGCATACCTTTTGTCAGGAAGAATGGTGATATTCAATCTGCCAGGAACACAAAAAAGCTGCTCAACAGAATGCATGATAATATAATCTTTTGCTTGGAAAATTTGGGCATTTGGGGAGCATTGCAAGCTTGCCGACTTCTTTTAACTGGTAATAACTCTGAGAGGAATGAATTGATAGAAGATGAAGGGATTTTAAGTGACGACTCTGTATGTGATCGATATCTTGTTCATGCTGCTGATGTTTTTGCTTCAGATTGTACGAGAGATGGATCTGCAAACGATTTATCTGATGTAGAGATTTTGAAGGAACCATTTTTCTCGAAAAAGCTGCTACGTCTTGTTGGAATTCTTTCCACCTTCAGGTTACAGCCAAATATGAAATGCATAATTTTTGTGAATAGGATTGTTACTGCAAGATCCTTGTCATACATACTGCAAAACCTTAAGTTCCTATTATTTTGGAAGTGCCATTTTCTTGTGGGAGTCCACTCTGGACTTAAAAGTATGTCAAGAAAGACAATGAAAAACATTCTTGAGAAGTTCAGAACAGGAGAGTTGAATCTTTTGATTGCAACTAAAGTCGGTGAAGAAGGACTTGACATTCAGACATGCTGCCTTGTGATACGTTTTGACCTTCCAGAAACTGTTGCGAGCTTTATCCAATCAAGAGGACGTGCAAGAATGCCTCTCTCTGAGTATGCATTTCTGGTGAACAGTGGGAACGAGAAGGAACTAGATTTGATTAAAAATTTTAAGAAAGATGAAGATCGTATGAATGTGGAAATTTCTTTCAGAACATCTACTGAGGTTTCTATTGGTCTTGAGGAGAGAATCTATATGGTTGATTCATCTGGTGCTTCCATCAGTTCAGGATATAGTATCTCTCTACTTCATCATTACTGCTCAAAACTGCCCCATGATGAGTACTTTTTCCCCAAGCCAAGCTTTTATTATTTTGAAGATTCAGGAGGAACAATCTGCAATATAATATTACCTTCGAATGCTCCAATAAATCAAATTGCCAGTACACCTCAATCTTCAGTTAATGCTGCCAAAAAAGATGCTTGTCTAAAAGCAATTGAAGAATTGCATAAATTGGGAGCCTTGACTGACCATCTCTTGCCACTGCAAAACAGTGTTCTTGAGGAGGAAACATTGCTGGTGTCTTCTGATTCTGGGAGCTCTGAAGATGAGGATTCACGAGGTGAACTACATGAGATGCTTGTTCCTGCTGTGCTTAAAGAATCATGGACTAACTTAGAGAACTGTGTCCTCCTTTATGCTTACTATATAAAATTTAATCCGAATCCTAAGGACAGGAGCTATAAAGAGTTTGCTCTCTTTGTCAAGTCTCCTCTCCCTAAGGAGGCTGAGAGAATGGAGCTGGATCTTCACCTATCTCGGCGTAGGTCGGTGATGACAAAGCTTACCCCATCTGGAGTTGCAGAATTTAAGAGAGAAGAGATCATGCAGGCACAGCATTTTCAAGAAATGTTCTTCAAGGTCATCCTTGATAGATCAGAACTCCTTTCTGAATTTATTACTTTAGGAAATGTCTTTCTTTCATCAAGCTCCTCAACATTCTATTTATTGCTTCCTGTTATTCTCTCCAATTGTGAGAATAAAGTGACAGTGGATTGGGGGATTGTCCAAAGATGTTTGTCGTCTCCACTTTTTAAGCCTCCTGTTGCAGCTGCAAAGATTGAAAATTTCCCTTCAGATGTTTGCTTGCACCTTGTAAATGGTTGTAGGAGTATAAGAGACATTGAGAATAGTTTGGTGTATGCCACACACAAGAGGGCCTTTTACTTTATTACTAGCATTGTTGGTGAAAAAAATGGTTACAGCCCATATAGAGATTCAGGCACTTTAAATCACGTGGAGCACTTAAACATGTCTGGCATTCATCTTAAGTACCCTGAACAACCTCTTCTGCATGCAAAACCACTTTTTAAGTTGCACAATTTGCTCCACAACCGAAAGCCGGAGGATTCAGAAGCAAATGAACTGGAAGAATACTTCATTGATTTGCCTCCTGAGCTTTGTCAGCTGAAAATAATAGGCTTCTCCAAAGAGATAGGGAGTTCGCTTTCTTTGTTACCATCAATTATGCATCGCCTGGAGAACTTGCTTGTGGCTATTGAACTGAAGCATGTTTTTTCTGCTTCATTTGCCGAGGGAGCTGAAGTTACTGCCCTTAGGGTCCTAGAAGCACTTACTACAGAGAAGTGTCAAGAGCGTTTTTCTCTTGAAAGGCTTGAGACACTTGGTGATTCCTTCCTCAAGTTTGCTGTGGCCCGCCATCTTTTTCTTTTACATGATGCACTTGATGAAGGGGAATTGACTAGGAGAAGGTCTAATGTTGTAAATAATTCTAATTTATTCAAGCTGGCAACAAGGCGAAATTTACAGGTTTACATACGTGATCAACCTTTTGATCCTTATCAGTTCTTTCCCTTGGGCCATCCCTGCCCAGTAATTTGTACAAAAGAAACTAAAGGAACTGTTCACCCACAATCTAGTTGTCAAGTAGATCATACAAAGAGTGAAGTCAAATGTAGTAGAAATCACCATTGGCTACATAAGGACACAATTTCTGATGTGGTTGAGGCTCTTGTAGGAGCATTTATAGTTGATAGGGGCTTCCAAGCGGCAACAGCATTTCTTAGATGGATAGGCATACGAGTGGACTTCCAAGGTTCTCAACTTAATAGTATTTGCGCTGCAAGCAAGAGATTTATGCCACTTTCCTCCCTATTGGATATTGGAGATCTTGAAAATTTGTTGGGTTACCAGTTTCTTCATAAGGGTCTCCTCCTTCAGGCGATTGTGCATCCTTCTTTTAATAGGCATGGGGGAGGCTGCTACCAGAGATTGGAGTTTCTTGGAGATGCTGTCTTAGATTATTTAATTACATCATATCTGTTTTCACTGTATCCAAAGTTGAAACCTGGTCAGTTGACTGATTTGAGATCAGTGTCAGTGAACAACAAGTCCTTTGCCAATGTTGCAGTGGACAGACGCTTACACAAATTTCTTATGTGTGACTCTTGTCACCTCAACGAGGCCATAGAAAAATATGTTGACTTCATCACATCATCATCACCAGACAGGGGATTGTTTGAAGGGCCAAAATGTCCGAAGGCTCTTGGTGACTTGGTAGAGTCTTGTTTCGGTGCTATTCTCCTTGATACAGGGTTCAACTTGAACCGTGTTTGGAAGATAATGCTATCCATTCTGGATCCAATCAAGAGCCTTTCAAGTGTGCAGCTCAATCCTATCAGGGAAGTACAGGAGCTTTCCCAGTGTTATAACTGGGATTTGCAGTTTCTAGTTGCAAAAGTTGGTAGAAATTTTTCAGTGGATGCAAAGGTAAATGCAGGAGATGTACCTCTATGTGTTTCTTACAGCAACATTAATAGAAAAGAAGCTATCAGAACTACTGCACATCAACTATATGTAAAGTTAAAGGCTCTAGGATATGCACCCAAATCAAAGTCCTTGGAAGAAGTTTTGAAGGGAAGCCCCAAGAATGAAGCCAAGCTGATTGGATACGATGAAACACCCATTGATGTCAGTGTTACTGACATAGTTGGATTTGAAAACATGAAATTGCAAGAATCTCTTGTGAATGATTTCAACCCCAAAACTCGTTCTATTAAAAGGACAACTTCTAGCGGTGTATCTTGCATCTCACCTGGTAGCAGACCTCCACCTTCTTTTGAAGTAAAAGCAGCAAGGGGTTCAGCCTCTGGTATTGAAGCCAAAGGCAAACCCCCCAATTGCTCCATTGTGGATCCTAGTTGTGGGATTGACTCGCCATCCAAAGGTGAATCACATGGTCGTACTGCTAGATCACAGTTATACGAAATCTGTGCTATCAATTGCTGGAAACCCCCTTTGTTTGAATGTTGCAAAGAGGAAGGACCAAGTCATTTGAGATCATTCACTTACAGGGTAATAGTTGAAATCGAAGAAGCTCCAGATATGATATTGGAGTGCTTCAGCAGTCCTCGGACCACAAAGAAGGCAGCAGCCGAGCATGCAGCGGAAGGTGCACTCTGGTATTTAAAACATGGAGGATACTTACAATAG

**>Gorai.009G053300.1_GrDCL4_ protein sequence length= 1654aa**

MPGGELSTDGTEPFMASKVKAFSTPSPIVEISRKDEPIMEKKEKDPRKIARKYQLELCKKAMEENIIVYLETGCGKTHIAVLLIYELGHLIRKPQNRVCIFLAPTVALVQQQARVIEDSLDFKVGTYCGNCRHLKNHHDWEIEIKEYEVLVMTPQILLRSLYHCFIRMDLIALLIFDECHHAQIKSNHPYAEIMKVFYDKATASMLPRIFGMTASPVVGKDASSQVNLPKSINSLENLLDAKVYSVGDKEELESFVASPVVRVYDYGPVNFGSSCSTIIYCSKLEEIKRQCIPFVRKNGDIQSARNTKKLLNRMHDNIIFCLENLGIWGALQACRLLLTGNNSERNELIEDEGILSDDSVCDRYLVHAADVFASDCTRDGSANDLSDVEILKEPFFSKKLLRLVGILSTFRLQPNMKCIIFVNRIVTARSLSYILQNLKFLLFWKCHFLVGVHSGLKSMSRKTMKNILEKFRTGELNLLIATKVGEEGLDIQTCCLVIRFDLPETVASFIQSRGRARMPLSEYAFLVNSGNEKELDLIKNFKKDEDRMNVEISFRTSTEVSIGLEERIYMVDSSGASISSGYSISLLHHYCSKLPHDEYFFPKPSFYYFEDSGGTICNIILPSNAPINQIASTPQSSVNAAKKDACLKAIEELHKLGALTDHLLPLQNSVLEEETLLVSSDSGSSEDEDSRGELHEMLVPAVLKESWTNLENCVLLYAYYIKFNPNPKDRSYKEFALFVKSPLPKEAERMELDLHLSRRRSVMTKLTPSGVAEFKREEIMQAQHFQEMFFKVILDRSELLSEFITLGNVFLSSSSSTFYLLLPVILSNCENKVTVDWGIVQRCLSSPLFKPPVAAAKIENFPSDVCLHLVNGCRSIRDIENSLVYATHKRAFYFITSIVGEKNGYSPYRDSGTLNHVEHLNMSGIHLKYPEQPLLHAKPLFKLHNLLHNRKPEDSEANELEEYFIDLPPELCQLKIIGFSKEIGSSLSLLPSIMHRLENLLVAIELKHVFSASFAEGAEVTALRVLEALTTEKCQERFSLERLETLGDSFLKFAVARHLFLLHDALDEGELTRRRSNVVNNSNLFKLATRRNLQVYIRDQPFDPYQFFPLGHPCPVICTKETKGTVHPQSSCQVDHTKSEVKCSRNHHWLHKDTISDVVEALVGAFIVDRGFQAATAFLRWIGIRVDFQGSQLNSICAASKRFMPLSSLLDIGDLENLLGYQFLHKGLLLQAIVHPSFNRHGGGCYQRLEFLGDAVLDYLITSYLFSLYPKLKPGQLTDLRSVSVNNKSFANVAVDRRLHKFLMCDSCHLNEAIEKYVDFITSSSPDRGLFEGPKCPKALGDLVESCFGAILLDTGFNLNRVWKIMLSILDPIKSLSSVQLNPIREVQELSQCYNWDLQFLVAKVGRNFSVDAKVNAGDVPLCVSYSNINRKEAIRTTAHQLYVKLKALGYAPKSKSLEEVLKGSPKNEAKLIGYDETPIDVSVTDIVGFENMKLQESLVNDFNPKTRSIKRTTSSGVSCISPGSRPPPSFEVKAARGSASGIEAKGKPPNCSIVDPSCGIDSPSKGESHGRTARSQLYEICAINCWKPPLFECCKEEGPSHLRSFTYRVIVEIEEAPDMILECFSSPRTTKKAAAEHAAEGALWYLKHGGYLQ
