# Supplementary material for: Genome-wide identification of the Dicer-like family in cotton and analysis of the DCL expression modulation in response to biotic stress in two contrasting commercial cultivars
Source: BMC Plant Biol. 2019 Nov 15;19:503. doi: 10.1186/s12870-019-2112-4 (PMC6858778; doi:10.1186/s12870-019-2112-4)
Supplement: Supplementary file 2 — Additional file 2: Table S1. Gene name and gene ID of DCLs in Arabidopsis, Medicago, rice, Populus, Physcomitrella and grapevine. Table S2. List of primers and amplicon characteristics of DCL genes. Table S3. DCL expression in organs. Figure S1. DCL amplification melting curves. Figure S2. Expression pattern of G. hirsutum acc. FM DCL genes based on transcriptome sequencing data. [file 12870_2019_2112_MOESM2_ESM.docx]

**Additional File 2**

**Table S1: Gene name and gene ID of DCLs from *Arabidopsis*, *Medicago*, rice, *Populus*, *Physcomitrella* and grapevine.**

| **Gene name*** | **Gene ID** | **Gene name*** | **Gene ID** |
| --- | --- | --- | --- |
| AtDCL1 | At1g01040 | PtDCL1 | Potri.002G181400.1 |
| AtDCL2 | At3g03300 | PtDCL2a | Potri.010G181400.1 |
| AtDCL3 | At3g43920 | PtDCL2b | Potri.008G075900.1 |
| AtDCL4 | At5g20320 | PtDCL3 | Potri.018G047500.1 |
| MtDCL1 | Medtr7g118350 | PtDCL4 | Potri.006G188800.1 |
| MtDCL2 | Medtr2g030490 | PpDCL1a | LOC112278919 |
| MtDCL3 | Medtr3g105390 | PpDCL1b | LOC112284512 |
| OsDCL1 | Os03g02970 | PpDCL3 | LOC112275623 |
| OsDCL2a | Os03g38740 | PpDCL4 | LOC112277957 |
| OsDCL2b | Os09g14610 | VvDCL1 | VIT_00027460001 |
| OsDCL3a | Os01g68120 | VvDCL2 | VIT_00019052001 |
| OsDCL3b | Os10g34430 | VvDCL3 | VIT_00035494001 |
| OsDCL4 | Os04g43050 | VvDCL4 | VIT_00001045001 |

*At, Mt, Os, Pt, Pp and Vv indicated *A. thaliana, M. truncatula, O. sativa,* *P. trichocarpa*, *P. patens* and *V. vinífera* respectively.

Table S2: List of primers and amplicon characteristics of *DCL* genes

| Gene | Forward | Reverse | Amplicon size (bp) | Efficiency ± SD* | R2 |
| --- | --- | --- | --- | --- | --- |
| DCL1 | TTCCGAGGAAAGAGGGCTAT | TTGCAGCTCAACTCAACACC | 105 | 0.928647 ±  0.0283724 | 0.9859 |
| DCL2a | ACTTCCGGGGTTTATCCGTG | TTTGCATTACACAGTGTTTCCTCA | 108 | 0.98622 ±  0.00353329 | 0.9466 |
| DCL2b | GCTGTCTTCTTGGTTCCCCA | AGTCAACCTCCATATCTCCCCA | 102 | 0.997162 ±  0.0091986 | 0.9908 |
| DCL3a | TCTGCTGTTAGCCTCATCCA | GAACGGCTGCATTAACAGGT | 139 | 0.992603 ±  0.0061324 | 0.9875 |
| DCL3b | CGCTTCTTCAAGGACAGCAT | GGCCATAACTGTTTCCGACA | 99 | 0.964982 ± 0.0028187 | 0.9754 |
| DCL4 | TTATTCAAGCTGGCAACAAGG | CAAATTACTGGGCAGGGATG | 104 | 0.976748 ±  0.0088974 | 0.9989 |

*Efficiency ± standard deviation (SD) generated by the Miners software.

References genes (miR390 and PP2A) were used according Fausto et al., 2017.

**Table S3 - DCL expression in organs**

| Sample/ gene | **DCL1** | **DCL2a** | **DCL2b** | **DCL3a** | **DCL3b** | **DCL4** |
| --- | --- | --- | --- | --- | --- | --- |
| **FM_Leaf ± SD** | 0.198 ± 0.058 | 1.538 ± 0.033 | 3.010 ± 0.140 | 1.883 ± 0.082 | 0.0335 ± 0.007 | 1.803 ± 0.101 |
| **DO_Leaf ± SD** | 1.031 ± 0.150 | 1.428 ± 0.124 | 0.007 ± 0.007 | 1.466 ± 0.475 | 0.002 ± 0.006 | 1.054 ± 0.571 |
| **FM_Flower ± SD** | 2.252 ± 0.013 | 1.005 ± 0.034 | 0.073 ± 0.003 | 1.629 ± 0.087 | 1.321 ± 0.066 | 0.190 ± 0.054 |
| **DO_Flower ± SD** | 1.261 ± 0.063 | 0.353 ± 0.086 | 0.974 ± 0.067 | 1.232 ± 0.106 | 1.998 ± 0.054 | 2.286 ± 0.006 |
| **FM_Root ± SD** | 6.955 ± 0.249 | 1.899 ± 0.013 | 2.778 ± 0.047 | 1.365 ± 0.043 | 0.0641 ± 0.012 | 2.437 ± 0.029 |
| **DO_Root ± SD** | 3.922 ± 0.212 | 1.374 ± 0.035 | 1.710 ± 0.118 | 1.329 ± 0.072 | 7.337 ± 0.0323 | 0.152 ± 0.004 |
| **FM_Stem ± SD** | 2.839 ± 0.080 | 1.331 ± 0.048 | 0.183 ± 0.003 | 0.673 ± 0.034 | 0.041 ± 0.005 | 1.139 ± 0.059 |

FM- Fibermax plants and DO - Delta Opal plants

**Figure S1 - DCL amplification melting curves**

**
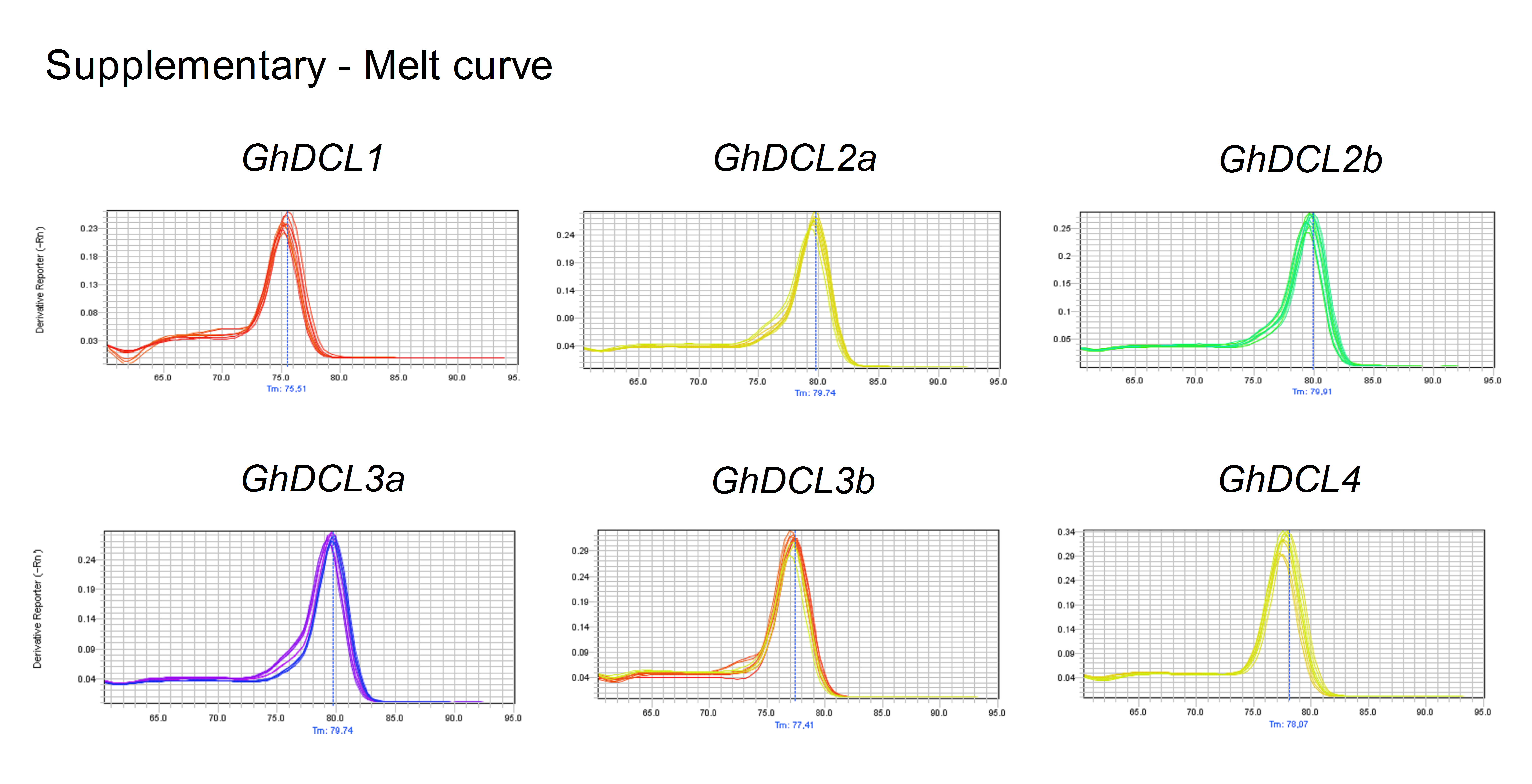
**

**Figure S2: Expression pattern of *G. hirsutum* acc. FM DCL genes based on transcriptome sequencing data**


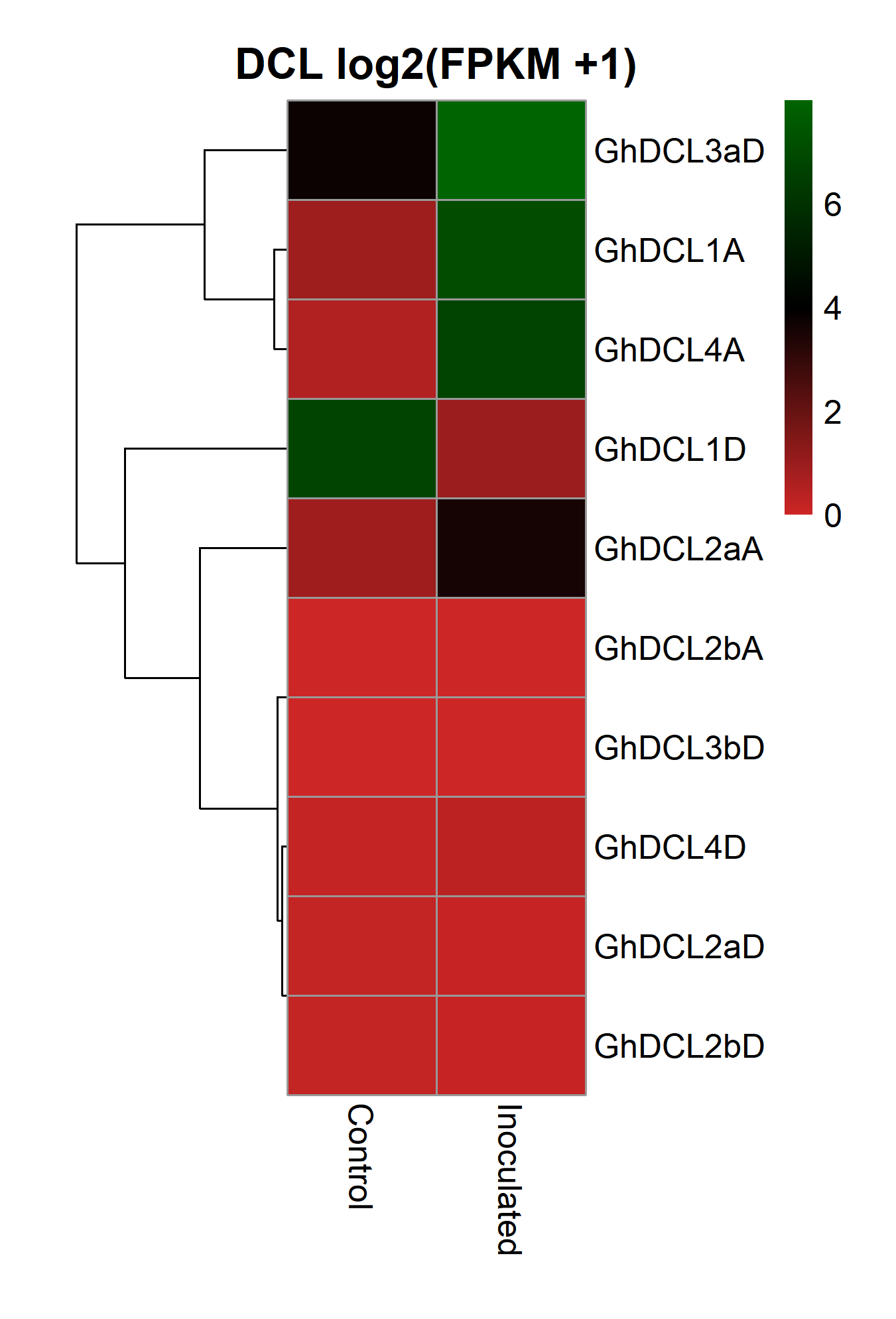


Expression pattern of the DCLs in uninfected (control) and 5 dpi CLRDV infected leaves were obtained as log of FPKM +1 values and calculated by Z-Score. A and D after DCL names represent the subgenome where the gene is found. For transcriptome analysis, plants were grown in greenhouse conditions and infected with CLRDV 60 days after germination as described in methods.
