# Supplementary material for: Genome-wide identification of the Dicer-like family in cotton and analysis of the DCL expression modulation in response to biotic stress in two contrasting commercial cultivars
Source: BMC Plant Biol. 2019 Nov 15;19:503. doi: 10.1186/s12870-019-2112-4 (PMC6858778; doi:10.1186/s12870-019-2112-4)
Supplement: Supplementary file 3 — Additional file 3: Figure S3. Cis-elements distribution in DCL promoters with enhancer elements. Figure S4. Promoter sequences of Gossypium hirsutum DCLs. [file 12870_2019_2112_MOESM3_ESM.docx]

**Additional file 3**

**Figure S3** - *Cis*-elements distribution in DCL promoters with enhancer elements

**
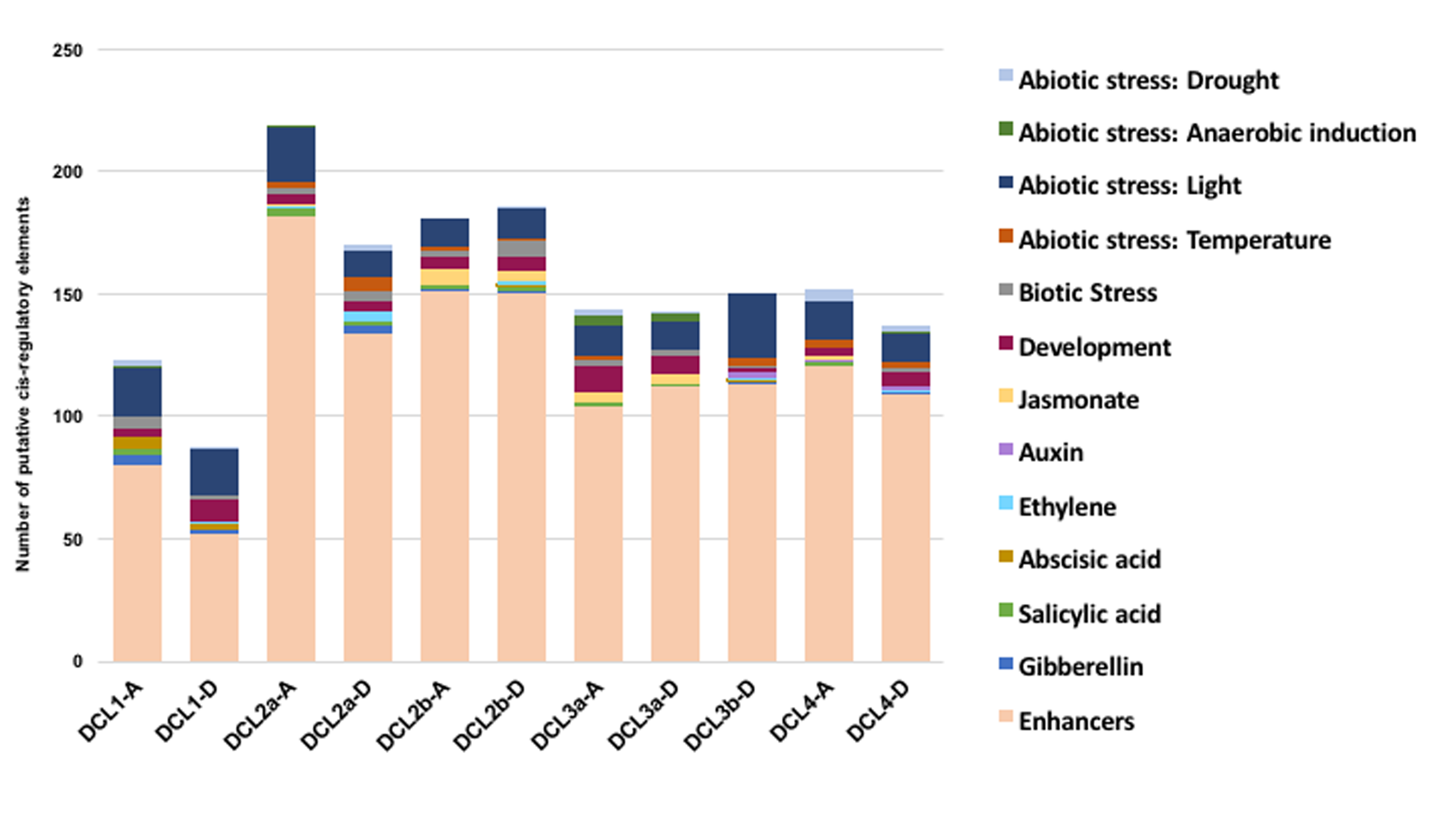
**

**Figure S4: Promoter sequences of *Gossypium hirsutum* DCL**

**Sequences 1500 bases upstream to start codon.**

**>Gh_A07G1222_NBI_AD1_v1.1ID=Gh_A07G1222_*Gossypium hirsutum*|promoter| length=1500_DCL1**

TATTTTTATTCCATTTTGATTCATGCTTTTATTGATATTTAATAAGTTTCAATTTAAAACAATCTCTTATTTTATTCTTTAAATCACAAACTTTTTTATATAACAAAAACAATTTTATTAAAATCTTGTTTTTGGTATGTAAACTAATTAGTATTAATTTAATATATAATCAATAAATATAAAAAAAATTGATTAATAAATCTAACAGAGGAACATATTCCTATAAATTATAACAAAAAAAAATGGAACTGTTAATAGAAATAGAATGCCCAAATAACCTTAACATTCCTATCCTCAAATGTAAGATATTTTTGTTTCTCCCAAAATCACAAGATTTTCTCATACGGAATGTGTGAGAAATAAATGCAAACATTTAATTGTAATGAGAAGAAGAAAAAGTGGAGCCACCCAAAACAAAATTCACCCAATCACTACACCAAAGGCCAATTGGTTTTCACTGTATCCAACCCACCAAAGGGAAAGTTTCTGGTCTCATAGGATAAAAAAATATTCAGCCTGATCTAAGGGGAATAAACTTTTATAATTATTTTTTCTTTATGAAGAATAAATCAAACGGCTGAGAATGTTCTATCATCTTTGACCTTTGAATAAGCTTTCGTTTTTGCCGGGATTCTACCAGTGGACTCCAACTGTTTGACACGTGTCTGTACTTTTCCATAAGCGTTGCTGTTCTCATGTCCTTGGTAGCAAAAAAGAGAACACTTAACATCTCCCCGCTCCCTACGGTAACGCAACTGAACAGTGAAGAGCGTAGTTCCAATAAAACCCTATCCTACCATCCACTCACAAATCACCACGTAATCCGGAACCCTTGTTGGGTCAAACGTGGCGTTATCGTATTGGTTGAAGCACTGTTTCTCTGTACTTTCGAGTTCGGAAAGTTAAAGAAAAACAGAACCGCCTCAAGTCACAGAAGTTTCTTTGTAAATTGTAAGGAAAAAAACATTGCGTGAAGACCAAAAAGAAAAAGGGGAAAACCCGTAGTTTTGGGTTTTATTATTTTAATTTTGTTTCTCCATCTCTCTTTCGTAGAGAAAACAGAGCTAAAACCCAGAAGAACCAAAGAAAGAGTGGGGGAGGAAAGCAAAAAACTGAGAAAGGCGCACGCTTGAAGGGAGTCAAATAAAAGAGAAACGGAAGATAACAGAATAGGCGTTGATAGACCGTAGAATCTAAGAATCACACACAAAAGTTTTGGTGGGGTTTTATGTTTTTTTCCCCCTTCAATTTTCTGTTTCACTTTTTTAAATGAATCCCCTTCCTTAACTACACTTCTTGCGTGTTTCCTGCGGTCTATCTACTCTTAATCTGTGTCTTCACTCACTCTTTTTTCTTCTCTCTGTTTTTTTCTGTATCTTTATAAAAACTATTGATTTCTTTCTCTTCTTTCTGGGTTTTTTTTATAATTCTTGTTGCTCTTGGTTCTTTTCCCCTTTTTTTTTTTGAAGTGGGAGTATCCGAGTTCTGGGCAATTGATTG

**>Gh_D07G1326_NBI_AD1_v1.1_ID=Gh_D07G1326_ *Gossypium hirsutum*|promoter| length=1500_DCL1**

ATAAGATTCGCTCATTTGATGTCTTTCTTGCAGGTTTTGGTTATGACAGCTCAAATTCTGCTGAATATTTTGAGACACAGCATAATTAAAATGGAATCAATCAATCTCCTTATCCTGGACGAGTGTCATCATGCTGTGAAGAAACATCCGTATTCACTGGTTATGTCTGAATTCTATCATACAACACCAAAGGAGAAGAGACCTTCTGTTTTTGGAATGACCGCTTCTCCTGTTAACTTGAAGGGTATATTTTTGTTTTCTGGTAGTTGTCTTCTTCAGATTTATCTCTGTTTGAGACCAAGGGCGGTCTCTCTTTCAAAATTTTCTTTCACTTGATTATAAGAAAGTTTCCCCCTTTAACTCATATTTTGAAATGTTATATTCAGGTGTTTCAAGTCAAGTTGATTGTGCTATAAAAATTCGTAATCTAGAAAGCAAACTGGATTCTGTAGTCTGTACCATCAAAGACCGCAAGGAACTCGAGAAACATGTGCCAATGCCTTCAGAAGTTGTGGTAGAGTATGACAAAGCAGCCAGTTTATGGTCCCTCCATGAACAAATAAAACAAATGGAAGCAACAGTTGAAGAAGCTGCACAATCAAGCTCTAGAAGAAGTAAATGGCAGTTCATGGGAGCTAGAGATGCAGGAGCCAAGGAAGAGCTTCACCAAGTTTATGGTGTATCTGAAAGAACAGAAAGTGATGGAGCTGCTAATTTGATACAAAAGTTGAGGGCTGTTAATTATGCACTGGGTGAACTGGGCCAATGGTGTGCTTATAAGGTATCATATGTGTTAAGTAATTAAGGTTACCACAACTTTTTCTTATTTTCTTGGTTGTGCAGAAATGATCNACCAAGTTTATGGTGTATCTGAAAGAACAGAAAGTGATGGAGCTGCTAATTTGATACAAAAGTTGAGGGCTGTTAATTATGCACTGGGTGAACTGGGCCAATGGTGTGCTTATAAGGTATCATATGTGTTAAGTAATTAAGGTTACCACAACTTTTTCTTATTTTCTTGGTTGTGCAGAAATGATCTTGGTTGTTTCCTTTCATTTCCTTTCTTTTTCAGGTTGCACAATCTTTTCTGACAGCTTTGCAAAATGATGAGAGGGCAAACTACCAGCTTGATGTCAAGTTTCAAGAATCTTACCTANCAATGCAGAAGCAGAGAACTGTAATGCTCAAGATGGGACCAATACTGATGAGATTGAGGAAGGAGAGCTCCCTGACAGTCATGGTGTGTCTTTTATTTAATTTTCTACCTTTCTTTTACTTCTTTTTTTTTTTGCCAGCACATTTTGAGACTGACATATAATTGAAAAGGATATCCTCAGGTTTTGAATGTATACGTGTGATAACGTTAAGTAGAACAAAACATGCATTGTTTACTTGCTTGTGTTGCGTTTCTTGTCATCCATTTTAAGGAAAATTCTTATCATTGTAGATGTTTCTAAACAAAAGATATTTTAAAAACATTTTGGTGTGTATTCTCAACTC

**>Gh_D05G3343_NBI_AD1_v1.1_ID=Gh_D05G3343_*Gossypium hirsutum*|promoter| length=1500_DCL2a**

ATCTTATAAATAAAAAACAAATATTTAATTAGGTACTTCAATATTAAGTAACCTCAAATGGGTATTTTACTAAAATAATAAAATAAAATAAAAAAATACTAAAATAGTATATTTTTTATTTACTAAAATGGTACAAAAAAACAGTTAAAAAAATAAAAAAAGTAGAAATATGGGCCTGCCACAGGTGGCACCAAAGGTGCATGTGGCAGAGGCAGCACCAACATGTTCGTATTTCGGCCTTTTATTCGTATTTTTTTCCCCGAATTTTATTACTTTAAAAAATATACTATTTTCTAATTTTATTATTTTACATTATTTTAGTACATTATGTTTGAAATGTATTCATTTAGTGTGTTTTTTATTAAAAGTAATAAAATAAGGGCCAAAATAAGGATTTTTTATTTTATTTTAAAAACACACTAAATGAATACATTTCAAACATAATGTACTAAAATAATATAAAATAATAAAATTAGAAAATAGTATATTTTTTTAAAGTAATAAAATTCGGGGAAAAAATACGAACAAAAGGCCGAAATAAGGTTTTTTTTAAAAATTTATTTAAAAAAATAGTAAATTAATACATTTCAAACATAATGTACTAAAATAATGTAAAATAATAAATACGAAAATAATATATTTTTATTGAAAGTAATAAAATCAGAAAAAAAATACGAACAACTGGCCAAAATTAGGGTTTTCTTCCAATTTATTTAAAAAACACACTAAATGAATACATTTCAAATATAATGTACTAAAATAATGTAAAATGATAAAATTAGAAAATAGTATATTTTTGAAAGTAATAAAATTCGAGGAAAAAATATGAATAAAGAGCCGAAATACGAATATATTGATACTGCCTCTGTCGCTGGCACCTTTGATGCTGCATGTGGCAAGCCCATATTTTTGCTTTTTTTTTTTATTTTTTAACTCTGTTTTATTTTGGTAAATAAAAAAATGTGTACTATTTTGGTTTTTTTAATTTTTTTTATTATTTTAATAAAATACCCAACCTCAAATTCTTTTTAAAAAAAGTAATTTTAAAATTACTTATTAAAAATATTATTAAAAAAATATATTTGAAAATTGTCACCTCCTTGGGAAAGAAGCAGCTATCAGAACTTCTCATTAGTCAGGCATTCTCTTCTCCCTCTCTGTTTCAGAAAAGTATCATTACAAAAGCAACGACAAAGAGATCATCCAACATTTTACACCTTCATTTCCTCATAGCCATTTTTCAGTAGTACTCCTTTACCAGAGACATATAAGTTGGATTTCCTTGATAATATGTTTTTGGGTTCTTGGCGGTGAGTTGGGAGAAAGGGTTTCTCTGATTTTATATTTATGTCAGCTAATTTTTTTAAAAAAAAGTGTCAGAGAATACTAAAAACAGTAGAACGTTAAAGAATGTCATGTGTAACCCAGAATGGTTGGGATTCTTTGTTATTGTAATGTGTTTACTTATACAGGCAGAGAAGTAACATAACTTTGTGAGCC

**>Gh_A01G1100_NBI_AD1_v1.1_ID=Gh_A01G1100_ *Gossypium hirsutum*|promoter| length=1500_DCL2b**

TTTTAATTATTTGTTTTATACAAGAGAATTTTTAATTGTTAAATATATTAATATATACGATATTTTAAATTAATATGATACAAATATTGCCTAGTCAAAATTAGATAATTATATTTTTACCCTTTAAAATATATAAAATTAATAAATGAGTAATGATAAAATTATACTTTAATCCTAAAAATGTTAAAATTTTAATTTACTCTTTGAAAAAATTATAAAAATATTAGCTAATACAATGGTGAAATTACATTTTAATTCTTATCAATGTTTATAACTTCCTATTCCCACAAAAAATTTTATAGTTCCGTCCCTAAAATATTAGATCGATTTAAAATTTTACATGTATAATAAATATAATTATACTAATAAATTCAATATCAATAATTAGAGTTTTAAAATATCATTGATAAAATTACCAAACGATATAAAAGCGTATTAAAATTAGCGAATGATATAAAATCGTATTAGTTTTACATATAAGTGATTATTTTCTATTTATTTATTTGTTTTTTACAATATTTATTTATTTTTATTCTACATCGTATACATAATACATTCTACGTATATATGTAAACATTATACAAGTCAAAGTATACTATATACACTCAAAACCTTCGTCACTAAGTTGTACTCCTTTCTCAGCACTGAACAACTTTGTTGTCTTTTTTGTTGCTATAAATTCAGATGTTCTACTCCCTTCCGTCTATCGTTACAAAAGCAGGAAAAAACCGAGCATCCTCCATTTACACTTTCAGTTTCTTATTGCCTTTTTCAAGTCCTTTACAAGTTGGCATTTTCAACATTGCTTCAATCTCGCGTATGTTCTTGATTCTTTGGCTCTCTTTTTGGGTTGTTCTTAGATTCTTTCTTTTTCTTTTTATCATCACATCGGTATTTGATTTAAGGAGAGGAAATATAAGTTGGGTTTTCTTGTGATAATAATTTTTGGGTTCTTGGCGGTGGGTTGCGAGAACTAGCCGGAGAAAGGGCCCGCGAGATCTCTCTATCATAGATTGCAGTCGAAGAAGTGTGGAGGTGGGACTCTGGACTTGTTCTGATATAAGTCACCCAGGTTTTATATTTATGTCAGCCAAAATATATATATTTTAAAAAAAAGGTAGTGTCAGAGAATAATAAAATACTAGAATGCTAAAGAATGTGATTTAGGATTTTGTGTTTTAACTTCAGACTTATCATGTTTAACCGAGAATGGTTGGGGTTGTATTTTTTTTTTATTGTCATATTGAATCGGCGGCTGAATCCTGTCTTATACTTCACTCAAGTGAATACTGGGCATAAGTTTGATTTCCTTTTGAACGTCATGTTGTTAGAGAAAAATAAGAAATGGGTTTTCTTGATCTTCTCTATATCACACATTGTTTTCACCTTTATCCGTGTTACAATTCTGAACATGTTTTTTCTTTGCATTGACGGACATGAGTGAATTCTTTGTTATGGTAATGTGTTTGCTTTTACAGGCAGAAAATATTAAATTGATAA

**>Gh_A04G0311_NBI-AD1_v1.1 ID=Gh_A04G0311_ *Gossypium hirsutum*|promoter| length=1500_DCL2a**

TTATCATTTAAGCCATTTATAATTTATAAAATTTTAAATTAATAATGATAAGATTGCACATTAATCCCAAAAATGATAAAAATCTGATTTAATTATTTAAAAATTATAAATATATAAACTATAAAAATATACAATTTAATCCTAGTCACAAAAATTTTCTTAGCTCTTAGCCTTCCTAATAAGTAAAAAGTAAATTTTGGTACTAGAAATAGATTCATTAGCTAGCGGTGGAGCAAGAGGTGACTTGGCAAATGTCTCGACCTCCTAAAATAAATTTTTTTATTTAAATATTTTATAATTTATAAAATTTTAAGTTAATAATAATAAAATTACATTTTAACTCTAGAAAATGATAAAAAATTAATTTAATCTTTTAAAAATTATAAATATATAAACTATTAAAATAATAAAATTATATTTTTATTATCGTAAAAATATACAATTAAATTCTACCCTCCAAAATCAATAAAAATTTGCATAGTATAAATAGATGCATCAATCTCCAAAGAATCCTACTTTTTGCATGGTTACTCATTTGCATTTTACCCTTTTATGAATGAAAATTCTCAACGTATGGATATTAAATTATCTTTTATTATGTGATAATAACAAAAATAACGTATAGTAATAAATAAATAAATAAATAAATTAAGAGTTTTCTTGAAAAATAACATTATAAATTAACCTTAAAATATCTCACTAATTAATAAAAAAACATTTGGATAAATAATAATTAAAAAAATACATTTTCATCGAAATTGCATCTAATGAAATACCAAAAAAAAATCTTAATATAGTAGTATTAATAATCAATAAAAATAGATTTGTTTAAGGTTAAAATATATTATCTTATAAATTAAAATAAATATTTAATTAGGTACTTCAATATGAAGTAACCTCAATTTCTTTTTAAAAAATAATTTTAAAATTTAATTAGGTACTTCAAGATTAAGTAACCTCAATTTCTTTTTCAAAAAATAATTTTAAAATTACTAAATCACTTATCGAATATTTATTAATTTATAAAAATTCTTTTAATATATGGTTTTAATATAAAATATTATTAAAAAATATATTTAGAACTTGTTTGAAATAATTTATAAGAGAAGAAAAATTTCCCACCCAAAAAAAAAAAAAACAAGGAAAATTTCAATCGTCACTTGCACACTATTATCATTATATAATTCAGGCATTCTCTTCTCTCTCTTTTGTCTCAGAAAAGTATCATTATTACACCTTCACTTCCTCATACCCATTTTTCAGTAGTATTCCTTTACCAGAGACATATAAGTTGGATTTTCTTGATGATTGTTTTTGGGTTCTTGGCGGTGAGTTGGGAGAAAGGGTTTCTCTGATTTTATATTTATGTCAGCTAAAATCTAAAAAAAAGTGTCAGAGAATAATAAAAACAGTAGAACGTTAACCCAGAATGGTTGGGATTCTTTGTTATGGTAATGTGTTTACTTTTACAGGCAGAGAAGTGATATAACTTTGTGAGCC

**>Gh_D01G1133_NBI-AD1_v1.1 ID=Gh_D01G1133_ *Gossypium hirsutum*|promoter| length=1500_DCL2b**

AATTTTTTTATTATTTGTTTTATATAAAAGAATTTTCAATTGTTAAATATATTAATATATACAATATTTTAAATTAATATGATACAAATATTGCCCGGTCAAAATTAGATAATTATATTTTACCCTTTAAAATATATAAAATTATAAATTAGTAATGATAAAATTATACTTTGACCCCAAAAATGTTAAGATTTTAATTTACTCTTTTAAAAAATTATAAAAATATTAGCTAATACAATGGTGAAATTGCATTTTAATTCTTATCAATATTTATAACTTTCAATTTCCACAAAAATATTTTTAGCTTCGTCCCTAAAATATTAGATCGATTTGAAATTTTACATGCATAATAAATAGAATTATATTGATAAATTTAATATCAATTATTAGAGTGTTAAAATATCATTGACAAAAAAATTACGAAAGGATATAAAATGGTATTAAAATTAGGGAATGATATAAAATCCTATTAGTTTTACACGTAAAGTGATTATTTTCTATTTATTTATTTATTTATTACAATATTTATTTATTTTTATTCTACATCGTATACATAATACATTCTACGTATATATGTAAAAATTATATACAGTCAAATATACTATATACACTCAAAACCTTCGCAACTAAGTTGTACTCCTTTCTCAGCACTGAACAACTTTGTTGTCTTTTACGTTACTATAAATTCAGATGTTCTATCTCTTCCGTCTATCGTTACAAAAGCAGGAAAAAACCGAGCATCCTCCATTTACACTTTCAGTTTCTCATTGCCTTTTTCAAGTCCTTTACAAGTTGGCATTTTCAAGATTGCTTCAATCTCGCGTATGTTCTTGATTCTTTGGCTCTCTTTCTGGGTTGTTCGTATATTCTTTCTTTTTCTTTTTATCATCACATCGGTATTTGATTTAAGGAGAGAAATATAAGTTGGGTTTTCTTGTGATAATAATTTTTGGGTTCTTGGCGGTGGGTTGCGAAAACTAGCCGGAGAAAGGGCCCGCAAGATCTCTCTATCATAGATTGCAGTCAAAGAAGTGTGGAAGTGGGACTCTGGACTTGTTCTGATATTTAAGTCACCCAGGTTTTATATTTATGTCAGCCAAAATATTTTTTTTAAAAAAAAGGTAGTGTCAGAGAATAATAAAATACTAGAATGCTAAAGAATGTGATTTAGGATTTTGTGTTTTAACTTCAGACTTATCATGTTTAACCGAGAATGGTTGGGATTGTATGTTTTTTTTATTGTCATATTTAATCGGCGGCTGAATCCTGTCATACTTCACTCAAGTGAATACTGGGCATATGTTTGATTTCCTTTTGAACGTCATGTTGTTAGAGAAAAATAAGAAATGGGTTTTCTTGATCTTCTCTATATCACATTGTTTTCACCTTTATCCGTGTTATAATTCTGAACATGTTTTTTCTTTGCATTGACGGACATGAGTGAATTCTTTGTTATGGTAATGTGTTTGCTTTTACAGGCAGAAAATATTAAATTGATAA

**>Gh_D06G0845_NBI-AD1_v1.1 ID=Gh_D06G0845_ *Gossypium hirsutum*|promoter| length=1500_DCL3a**

AAATGGTTTTTTCGCTAGCTCATAAATAATTGGAAGTTCAGTCATGATATTATAGTGATTTAATGTTATTATAGATGTATTTATGAAAAGAATATCGTCACGACACCATTAAATGTTGGAAAAAATGGATATTTTGGAAGCTTTGAGGCATATTATCGATTAGTAAATGTGGATATTCGAATCATAAAACTATGGTTTTATATTTTACTCCATGAGAACTTTACAATGGTATATCATATGCTCAAAATAGTTGAGTGATGAATGAGAAATTGATACTCAAAGTAAGTCACTTGTGAATTATGTTGAGGAAAGTAGAAAGCTAGTCACTTGTGAATTATGTTGAGAAAGTAGAAAGCTTAGTCCCACATTTGTTAGATATCAAGAGTGAAATATATTTATATATGTGAACCAACTTGGTGGTTATTGAATGACTAAATTAATACTATCTCTCATGCGTAGGGGGCACAAATCTAAACCTGTTAGGGTTGAGGGCACACCCACACGATGTGGGTAACGGGAAATATTTGTATTGGTTTGAGCATTTTTTAGCCCCTATGAATATTTAAGTAATCTGACACGGAAGATACACACTGTGATAGAAGCCATCATATTGTTGTATCCATCTTCCAGTATAATATTTTAATTTTCTATTTTCCAAATTATATATTATTGTTCTTATATATAAATATTTTATAATACTGTCATTTTACTAACATTTTTGTGCAACATTAAAAGCAACCACGGTATCAAATCCACTCTCATTAATATTTCCAAATTTTTAAGCTCACCACACAAGTAATCGCGGGAGAAGGGCTCACTCCAAGAAGAGAGACCACACTTTGCTTAAGTGTAACCTCAACTCCCTCAAATTATCACTTCGAGTATATAACTTAATTATTAAACAAAGAATATGTTAAAAAATGTTGCAAAATCACAATATGCAACATAACACAAGTATGAACTACGTCAACTGTCGAACTGTTATCGTTTCCAACTCATAAGCCAAACCCTCTTACCGCGCATTACATGTCCAATAAAAGAGGGAGGTTTGCATTGCGTGTCCAATAAACATTGGGCTGTCATTGCACATTCCCCATAACGCGCGATAACCAAACAGCTTGGGTTCAAATCATCATTGGGCTGTCATTGCACACTCCCCCTAACGCGCGTTAACCAAAACAGCTTGGGTCCAAATCATCTGATAGTTTCAACCTGTTTCTCTCTGCAGACTCGGCACTGCTACTGTTTTCGAGTTTCAATGAGTTTAAATAAAATCTAACAAAACCCCATCACCAAATTTCATTTTATACTTGTATCATACATTAAGTGCCTTTAGCGAGTCAGTGTTCTTATAGCTTCTGGTAGTTGGCTAGTTAATTGCGACCCGAGGTTTCATTGTAAACCGTTCGGTGTTGGGGCAGGGTTCTTCCTTTTTCTCCATAAAAATAGCCAAAATGAGTTAATTGGAAAATTCCCAGAACAGTACATTTTATATAATCC

**>Gh_D13G2027_NBI-AD1_v1.1 ID=Gh_D13G2027_ *Gossypium hirsutum*|promoter| length=1500_DCL3b**

AAGAAAAAAAGAACAGAAGTAGCAGAAGTGTATGATTAATGTTTAAAAAGGTTAGATGAGAATAGCAAATGCATTTTGGTAGAGCATCAGGGGAGGGCGAGGCTGAGTCTAGCCGATAACTTGTTTTTCTCCGTTTTGTTAATTTTGTATCTTAAATTTATACTTTGTTAAATTACTTCTTGAATTACATCATGATTGTATGATTCATGAAAATTAAACTATTGTGCTAAATGCTGAAAACTTTTCATTATTACTTTTTAGGTCTATATAGCTGTATAGTATTCAAGTTCAAGCTCAACTCGCCATTCAAAGTTCATCTTGAGCTTGGTTGAGTTTCGGTTTTAAAATTCGAGTTTGATTTAAGATAAAAAATTATATTACTTTTGAGTCTATTAATTTAATCTGCTTTATACGGGATTATAAACTTGGTTATTTGTGTTTGAACTTAAATTCGATTTAAATCCATCATTAGAACACTTAGAATTTAATTTATCCAATAGTTCGAGTTTGGCTTCGATAATAGCAGGGAAAATTACAATAGAGGTCTTTATATTAAGAATTAGATTATATTGTATTCTTTTTTTTATAAAATGAGTAAATTAGTCCTTATACTTTAGATAAAGAAGTAAATTGGTCATTTTGTTAAAAATTTTATCTATTTCTACTATTAAAAATTAGTTCTCATACATCATTTTGAAGTACATGAGGCACACTATGAGTTACTATCTGATTATTTTATCAATCATGTTAGTTTTTAATAGTACAAATAGATAATTTTTTTACAAAAATAATTAATTTACTCTTTAATCTAATATATAAAAATTTAATTATCTATTTTTTCAGTAAAAAACAAAATGCAAAACCTTCTTAGTTTTACGATAATAGTTTAAAATACATCTGTTTCACTCTATGCATAGGGCATTATAACGTGTCAACTCATGATTGGGGCGTTTATGTGCTTCTCCCGATGGCGCGTAAATCAAACAACTTTCGTCCGAATCTTTTAAACAGCTTCGTCTGAATATGGAATTTTTTTCTTTTTATGGTAGTAAACCTGGAACTTTCTAATACAACAAAAAATAAGTTATTATATTTTTCATAAATTTAAAATTTAATTTTTGTATTTTTATTTTCAAAATTTTAATCTCTTTATTTTTAAAATTTTAAAATTCATAAACACTAATAAAATTATTTAATTAAATTCAAAGTTTATTACAAGATTAATTTTTTAATTGAATTACTACTAAATAAATATTTTTTATTTCAAAATGTTTTTTACCATAAAAATGGTGTGAATTTAATGGAATCAATGTCGTTGCATTGCACAGTCTGCTTCGCACCCCCATTTTCTCAGTTTTCTCACTGAAATATTCGTTCTTTTAGAACTTCACGTATATGGTAATTTTCATCTTTATCCTTTCTAGTCGTTTTCATTTCAATGATAAAATTTTAATGTTTGTCTTTTTGTCTAAATACTCAGCATCCAACATGCATTCTCCC

**>A06:22197055-22207732_NBI-AD1_v1.1_*Gossypium hirsutum*|Promoter| length=1500bp_DCL3a**

AAAGTGGAATGAAAGCATCGGTCATTTGTTTTGTTAAATCGCAAGCTCATTGGAAAAAAGTTTTTAAATATAGCTTTTGTAATTATAGTGGTTTGACTCTATGACTTAATAACAATGTAATTAATAGACGAAAAATCAAAACTTAATTACATGTTATTTTAGTCTACATTTTATATGTTTAAATGGTTTTTTCGCTAGCTCATAAATAATTGGAAGTTCAGTCATGATATTATAGTGGTTTGATGTTATTATAGATGTATTTATGAAAAGAATATCGTCACGACGCCATTAAATATTAGAAAAAATGGATATCTTAGAAGCTTTGAGGCATATTATCGATTAGTAAATATGGAGATTTGAATTATAAAAGTATGGTTTTATATTCTACTCCATGAGATCTTTACAACAATATATCATATACTCAAAATGGTTGAGTGATGGATGAGAAATTGATACTCAAAGTAAGCCACTTGTGAGAAATTGATACTCAAAATAAGCTACTTGTGAATTATGTTGAGGAAAATAAAAACCTTTGTCCTACATTTGTTAGATATCAAGAGTGAAATATTTTTATATATGTGAACCAACTAAGTGGTTATTGAATGACTAAATTAATACTTTCCCTTGTGCGCAAGGGGTACAAATCCAAACCTATTAAGATTGTGCTCCTACAATATAGTGATGCAAAATGTTTGGATCAGTTGGACCTTTTTAACCCTGTAGATATTTACTCATTAATATTTCCAAATTTTTAAGCTTACCACATAAGTAATCGCGGGAGAAGGGCTCACTCCAAGAAGCGAGACCACACTTTACTTAAGTGTAGCCTCAACTCCCTCAAATTATCACTTCGAGTATATAACTTAATTATTGAATAAGAATAATTTATTAAAAAAAAGAATATGTTAAAAAATGTTGCAAAATCACAATATGCAACATAACACAAGTATGAACTACGTCAACTGTCGAACTGTTATCGTTTGCGCTCATAAGCCAGACCCCCTTAACGCGCATTACATGTCCATAAAAGAGGGAGGTTTGCATTGCGTGTCCGATAAACATTGGACTGTCATTGCACATTCCCCATAACGCTCGTTAACCAAACCGCTTGGGTCCAAATCATCATTGGGCTGTCATTGCACACTCCCCCTAACGCGCGTTAACCAAAACAGCTTGGGTCCAAATCATCTGATAGTTTCAACCTGTTTCTCTCTGCAGACTCAGCACTGCTACTGTTTTCGAGTTTCAACGAGTTTAAGTAAAATCTAACAAAACCCCATCCCCAAAATTTCATTTTATACTTGTATCATACATTAAGTGCCTTTAGCGACTCAGTGTTCTTATAGCTTCTGGTAGTTGGCTAGTTAATTGCGTCCCGAGCTTTCATTGTAAACCGTTCGGTGTTGGGGGCAGGGTTCTTCCTTTTTCTCCATAAAAATAGCCAAAATGAGTTAATTAGAAAATTCCCCGAACAGTACATTTTAATTTTTATATAATCC

**>Gh_D05G0516_NBI-AD1_v1.1 ID=Gh_D05G0516_*Gossypium hirsutum*|promoter| length=1500_DCL4**

CTCCTAAGTATGGAAATGTGAATTTAAGATATTAGGAGCATCAAATTCACTAATTTACATTATTAATCATCCAAAAATGCATTAAAATGAGATTAAATATGTTACTTTTATAGCTTATCAATTTCTCTAAGTCCTTGGTGATTTATGGTTCATAGAGTTTCTACAAAAACTCGATTTGTTTGGAGCTCTTTTGCATCCGTTGTTTCAAGTAAGAGATGTTTCCACTACCACATGAGTAATGATTTGTATGGAGATTTGCTACATCGTGATTTTTATTGATGGTTGAGAGTGTCGTTTGGCTGAAAGAGTTGTGATAACATGCCAGCGTCTTTAATTATTTATGATTTAAATAAGGAGGCCAAATTCAAACAAAGAAAGCTCGTTTTATTCCTCATAAATTTTAAGTTATTTTTCCTTTTGGGGCTATGTTTAGTTCGAGACCGAAATCTTCGACTACTTTCTCCGTTTTATATAACTAGATTTTTGACTTTGCTGGCCATTATGTATACGATGGCAAAGTTTTCTAACTCCACAAATAAAAAAACTGCTACTTGTCTTATTTAATTAGTTTTTATTTTTTAAAGTATAGTTTTTATTTTTTAAAGTATCATAAAATTATCCATAAAAAATATTTAAAAGTAAAGTAACTTTAGGAGTTGTTTAAATTACAAATTAAGTAATTGATTTTTAGGTTAAATTTTGCTATTAATCCTCGTATTTTGTAAAAGTTATGGATTGAGTTCTGATACTTTAATTTGATCATTTTTAATTTTTGTACTTTTTAAAATTTAAAATTGATGATAACTGTTAAATTCATTAAGTTAATTTTTATTATTTCTAAAATTTAATGCGCTAAACATATTATCACATGTGTAATGTCATGTCAGCTTGTTATTTTCACATATTGTTTACTAAAAATCCAGTTAATGGATTAACAATTGTCATTTGGGTCAAGATTGAAATTTTAAAATTTGCGATTTAGAATGATTCAATTGAAAAATATGAATTAAATCTATAATTGTACAAGACTAATAATTAAATTGAAACAAATAAATATAATGGTTATTATTTAATTTAGACTAAAATTTCAAAATTAAAGAAAAAACAGCGGAATTTAATATATTGTTTATTAATTTGTTTTTGAGAATAAAAGAAACCATTCTCCACTGTTTAATTTTGATTTTATTTATGAAATTTCGGATTTCCGAGTGGGAGACGAGATTCGGCAGTTCGACTCATTAATAAGCGAACAAATGTAATGATAAAAAATTGTTGGAAGAGGAGTTACTAGATTTGACTTTCCATTGACTGAGCACAATTCATTACAAGAAATCCTAGGAGACCGCTCTGCTTAGTAAACCGAATGGGATTTCGACATCTATTTGTCCATTTGATCAAAGCAAACAATAATCTTTTTTTCTTTTTGCTTAGCGGTTGAGACTTGGAAAATAAGCAATTTTTACCAGTCTTCTCACCGCTGTGGCTATAATTCCCTCGTTG

**>Gh_A05G0400_NBI-AD1_v1.1 ID=Gh_A05G0400_*Gossypium hirsutum*|promoter| length=1500_DCL4**

TTCTTTTTAAAAAGCGAGAGGACAAATCATCTTAATAATATATTTTTTGTTGGCCGGTTTCAATCATGATTTAGTGCGATGTGGTAATTGTTGTGACACTCGAGTCTTGTTGTGCCTGGGTTTCTTTAAGTTTTTGGTGACTTATGGTCCATTGAGTTTCTATAAAAACTCAATTTGTTTGGGGTTCTTTTGCATCCGTTGTTTCAAGTAAGAGATGTTTCTACTACCACATGAGCAATGATTTGTATGAAAATTTGCTACATCATGATTCTTATTGATGGTTGAGAGTGTTGTTTGGCTGAAAGGATTGTGATAACATGCTGACGTCTTTAATTATTCTCGATTTAAGTAATGAGGCGAAATTCAAACAAAGAAAACTCGTTTTATTCCTCATAATTTGTAAGTTATTTTACCTTTTAGCGCTATGTTTAGTTCTGGGCCGAAATTTTCAACTGCTTTCTCCGTTTTATATAACTAGATTTTTTACTTTGCTAGCCATTGTGTATACATGATGTCAAAGTTTATGGAAAAGAAAAAACTGCTACTTATCTTATTTAATTAGTTTTTATTTTTTAAGGTATCATATAATTATCCATAAAAAATATTTAAAAGTAAAGTAACTTTAGGAGTTGTTTATATTACAAATTAACTTAAGTAATTAATTTTTAGGTTAAATTTTGCTATTAATCCTCCTATTTTGTAAAAGTAATGGATTGAGTCCTAATACTTTAATTTGATCATTTTTAATTTTTGTACTTTTCAAAATTTAAAATTGATGATAACTGCTAAATTGATTAAGTTAAGTTTTATTATTTCTAAAATTTAATGCACTAAATATATTATCACATGTGTAATGTCATGCAAGCTTGTTATTTTCACATATTATTTACTAAAAATCCCGTTAATAGATTAACAACTGTCATTTGAGTCGAGATTAAAATTTTAAAATTTACGAGTTAGAATAATTCAATTAAAAAATATAAATTAAATCTATAATTGTACAAAACAATAATTGAATTGAAACAAATAAATATAATGGTTATTATTTAATTTAGACTAAAATTTGAAAAAGAAAAAAGAAAAAAAAGACTAAAATTGGCTCAGACAGCGGAATTTAATATATTGTTTATTCATTTGTTTTTGAGAATAAAAGGAACTATTCTCAACTGTTTAATTTTGATTTTTTTTATGAAATTTCGGATTTCCGAGTGAGAGACGAGATTCGGCAGTTCGACTCATTAATAAGCGAACAAATGTAATGATAAAAAATTGTTAGAAGAGGAGTTACTAGATTTGACTTTCCATTGACTGAGCACAATTCATAACAAGAAATCCTAGGAGACCGCTCTGCTTAGTAAACCGAAGGGGATTTCGACATCTATTTGTCCATTTGATCAAAGCAAACAAGAATCTTTTGTTCTTTTTGCTTAGCAGTTAAGACTTGGAAAATAAACAATTTTTACCAGCCTTCTCACCGCCGTGGCTATAATTCCCTCATTG
